# Tracing global flows of bioactive compounds from farm to fork in Nutrient Balance Sheets can help guide intervention towards healthier food supplies

**DOI:** 10.1038/s43016-022-00585-w

**Published:** 2022-09-19

**Authors:** Keith Lividini, William A Masters

**Affiliations:** 1 Friedman School of Nutrition Science and Policy, Tufts University, Boston MA; 2 International Food Policy Research Institute (IFPRI), Washington DC

## Abstract

Adequate supplies of healthy foods available in each country are a necessary but not sufficient condition for adequate intake by each individual. This study provides complete Nutrient Balance Sheets that account for all plant-based and animal-sourced food flows from farm production through trade to non-food uses and waste in 173 countries from 1961 to 2018. We track 36 bioactive compounds in all farm commodities recorded by the Food and Agriculture Organization of the United Nations, accounting for nutrient-specific losses in processing and cooking as well as bioavailability. We compare supply to requirements given each country’s age-sex distribution and find that the adequacy of food supplies has increased but often remains below total needs, with even faster rise in energy levels and lower density of some nutrients per calorie. We use this nutrient accounting to show how gaps could be filled, either from food production and trade or from selected biofortification, fortification and supplementation scenarios for nutrients of concern such as vitamin A, iron and zinc.

A first step in monitoring global food systems is complete accounting of farm production, trade and distribution of each commodity, as in Food Balance Sheets (FBS) maintained by the Food and Agriculture Organization of the United Nations (FAO) since 1949.^1,2^ These are used primarily to guide agricultural policy, although the FBS are also increasingly used to track supplies of more nutritious foods^3^. The FAO itself uses the FBS to track total dietary energy and grams of protein or fat, while others have transformed FBS data into availability of selected micronutrients^4–18^.

This study provides the first complete global accounting of global micronutrient flows, building on a number of recent studies that use aggregate FBS categories ^19,20^ or the more disaggregated Supply and Utilization Accounts (SUAs).^21^ Those previous studies focus on nutrient composition of nutrients available for final human consumption only, and often use statistical procedures to smooth observations and impute missing data such as the Global Nutrient Database (GND) that uses the FBS as a Bayesian prior to infer availability of 156 nutrients with spatiotemporal regression.^21^ Bayesian imputation and smoothing is common in the health sciences, for example in the Global Burden of Disease (GBD) study which uses FBS data as a Bayesian prior to estimate levels of 15 dietary risk factors^22^, and the Global Dietary Database (GDD) which uses survey observations as its Bayesian priors which are updated with FBS and other data as covariates^23,24^.

Unlike previous work, our Nutrient Balance Sheets (NBS) is designed for complete accounting of all nutrient flows from farm to fork, in a fully transparent and replicable manner designed to be updated with new FBS data each year, and to be expanded as new opportunities become available to further disaggregate commodities, decompose product flows or track additional bioactive compounds in each food. Complete accounting is particularly important for monitoring and intervention in global food systems, to take account of nutritional linkages from agricultural production through trade and transformation prior to consumption, whereas previous studies have focused on single countries^25–27^ or a limited set of years^28,29^, or did not account for food system outflows, food loss and waste, or the distribution of nutrient availability relative to requirements^30,31^.

We develop and apply the NBS in this study to achieve three aims. First, we demonstrate the feasibility of complete accounting for nutrient production and flows in food commodities recorded in global statistics, using nutrient composition data and nutrient losses in cereal processing and meal preparation as well as bioavailability data for iron and zinc. Second, we compare the resulting nutrient availability to total requirements in each country every year, accounting for differences in demographic composition by age and sex. Finally, we identify the magnitude of change needed to provide adequate supplies of key nutrients by rebalancing agricultural production towards nutrient-dense commodities, or through biofortification of locally grown crops, fortification of packaged foods sold on local markets, or delivery of supplements to specific sub-populations such as vitamin A for children.

## Results

Food balance information was initially available from the FAO for 186 current and former countries and territories, covering 97.6% of the global population in 2018, the latest year of available FBS data as of this study (see Supplementary Figure 1A and 1B). We identified countries and years with implausible reporting based on the inter-quartile range (IQR) of total dietary energy per capita, leading to dropped data for Antigua and Barbuda in 1961-1964, and Comoros and Seychelles which had been available for only 2014-2018. One nutrient, choline, was dropped from the analysis due to missing values in food composition data for some cereals and meats. Food composition data was also missing for vitamins D and E in pig meat, and vitamin K in goat meat, and those were set at their lower bound of zero.

### Nutrient composition, food classification and uncertainty

The nutrients and other compounds included in this study are shown in Supplementary Table 1, and the classification system used to quantify food flows from farm production to household consumption is shown in Supplementary Table 2. Our classification system starts with FBS data, which tracks production and utilization in 21 food subgroups divided into 98 food categories, which we further disaggregate using SUA and other information as described in the methods section and detailed in the Supplementary Information. For reporting purposes only, we then aggregate flows into four groups: starchy staples; nutrient-dense vegetal foods; animal source foods; and other foods.

All FAO data is reported in terms of total weight. Of the 98 food categories used in the FBS, a total of 14 categories are used to account for other products within their subgroup that are not elsewhere classified. These 14 “other foods” categories account for 12%-24% of total national food production by weight, with roughly 27% of countries having more than 20% of their total weight that are classified in the FBS only by their subgroup. The subgroups with the highest share of total weight in these “other” categories include vegetables, fruits and pulses, each of which has over 40% of total weight in their highly diverse category of not-elsewhere-classified items. To address uncertainty about the composition of these and other FBS categories such as tree nuts that contain a diversity of items with different food composition, we conducted a systematic sensitivity analysis using Monte Carlo methods to sample many possible combinations of items within each category. The resulting range of plausible nutrient values is greater than +/- 5% for total global availability of only a few nutrients, notably vitamins B12, D, E, and K, and trans fatty acids (TFA). A principal goal of the NBS is to enable more accurate measurement of nutrient flows over time, as more precise estimates of food quantities matched to item descriptions of known nutrient composition becomes available from the FAO and other sources.

### Comparison of NBS apparent intake to FAO and GDD estimates

To benchmark our work, we compare nutrient flows measured in the NBS to the FAO’s own estimates of macronutrient availability per capita shown in Table 1. NBS results are closely correlated with FAO estimates. Pearson correlation coefficients equal 0.94, 0.97, and 0.95, for energy, protein, and fat, respectively, and all are statistically significant. However, regression lines from country-level scatterplots (not shown) are systematically higher than the lines of equality indicating that NBS estimates are generally higher than FAOSTAT estimates. For this comparison we use NBS data that corresponds to the FAO analysis which includes cereal processing but not the additional accounting for food loss and waste (FLW), cooking retention and bioavailability. When FLW and nutrient retention from cooking are additionally accounted for in the final best NBS estimate, regression lines are generally lower than the line of equality (not shown), indicating systematically lower values from the NBS compared with FAOSTAT estimates due to FLW and cooking losses. The overall correlations are still generally high between the NBS and FAOSTAT (Pearson’s *r* = 0.90, 0.96, and 0.95 for dietary energy, protein, and fat, respectively) and highly statistically significant.

Fixed effects panel regression models (not shown) were used to explore the reasons for the differences between NBS and FAOSTAT estimates for per capita energy, protein, and fat. For each macronutrient, differences arise due to specific post hoc adjustments that FAOSTAT makes for available food supply quantities as well as differences in how the food composition tables are created and matched to food products. For dietary energy, the most important food groups for which these differences apply are for starchy staples followed by the catchall ‘other’ categories, cereal grains, roots, and groundnuts, as well as sugar, tomatoes, citrus, plant oils, animal fats, wine and other miscellaneous foods. For protein, the categories with important differences in availability between the NBS and FAOSTAT are cereals such as wheat and millet, sugar beet, pulses, fruits and vegetables, and pig meat. For fat, categories with important differences are maize, vegetables oils, alcoholic beverages, dairy, animal fats and shellfish.

Another useful benchmark for the NBS is comparing total estimated availability per person to each country’s mean dietary intake estimated from survey data by the GDD^32^ (Table 1). Across the twenty nutrients compared, five nutrients had either a Pearson or Spearman correlation greater than 0.5; ten nutrients had either type ranging between 0.2 and 0.5; and five nutrients had correlations less than 0.2. The nutrients with the strongest correlations between the NBS and GDD were carbohydrate (*r*_P_ = 0.77; *r*_S_ = 0.77), potassium (*r*_P_ = 0.62; *r*_S_ = 0.70), riboflavin (B2) (*r*_P_ = 0.67; *r*_S_ = 0.68), and calcium (*r*_P_ = 0.61; *r*_S_ = 0.64), and all were highly statistically significant. The nutrients with the weakest correlations between the NBS and GDD were niacin (*r*_P_ = 0.07; *r*_S_ = 0.06), vitamin B6 (*r*_P_ = -0.01; *r*_S_ = 0.05), selenium (*r*_P_ = -0.04; *r*_S_ = -0.03), and vitamin E (*r*_P_ = -0.16; *r*_S_ = -0.16). Values for vitamin B6 and selenium were not statistically significant.

### Variation in nutrient production and intake, 1961-2018

**Figure 1:**
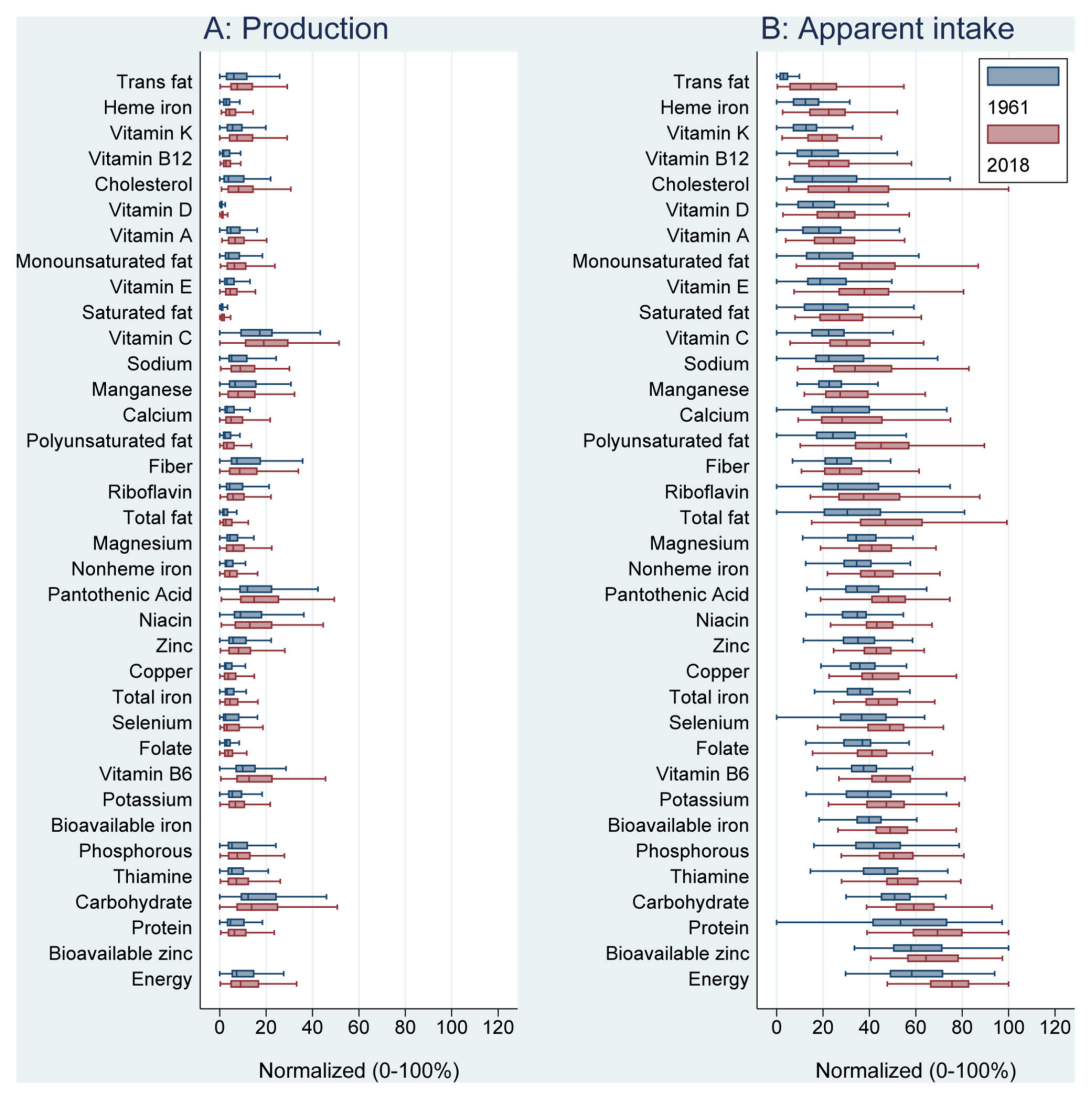
Production and apparent intake of nutrients per capita, 1961 and 2018 Annual per capita nutrients from production and apparent intake were calculated for each country, and then normalized for each nutrient as percentages from zero to 100 of the global minimum and maximum observed in 1961 and 2018 combined, and for production and apparent intake separately: (value - min)/(max - min) * 100%. Values outside box whiskers are removed. Boxes are sorted by 1961 median normalized apparent intake. Bioavailable iron and zinc were not calculated for production.

Figure 1 shows the range across countries for production and apparent intake per person of each nutrient for 1961 and 2018 without accounting for additional nutrients from fortification, supplementation, or other sources. To account for differences in units and scales, each nutrient’s production and apparent intake values are separately normalized across 1961 and 2018 values and presented as values between 0 and 100% of the maximum observed for each nutrient. Nutrients are sorted by 1961 median apparent intake. Figure 1 shows that median global production and apparent intake of all nutrients increased from 1961 to 2018. Within each boxplot, the distribution of nutrient production is notably more skewed than its apparent intake, indicating the high concentration of agricultural output per capita in particular countries, and the role of trade and other factors in helping to equalize apparent intake across countries.

**Figure 2:**
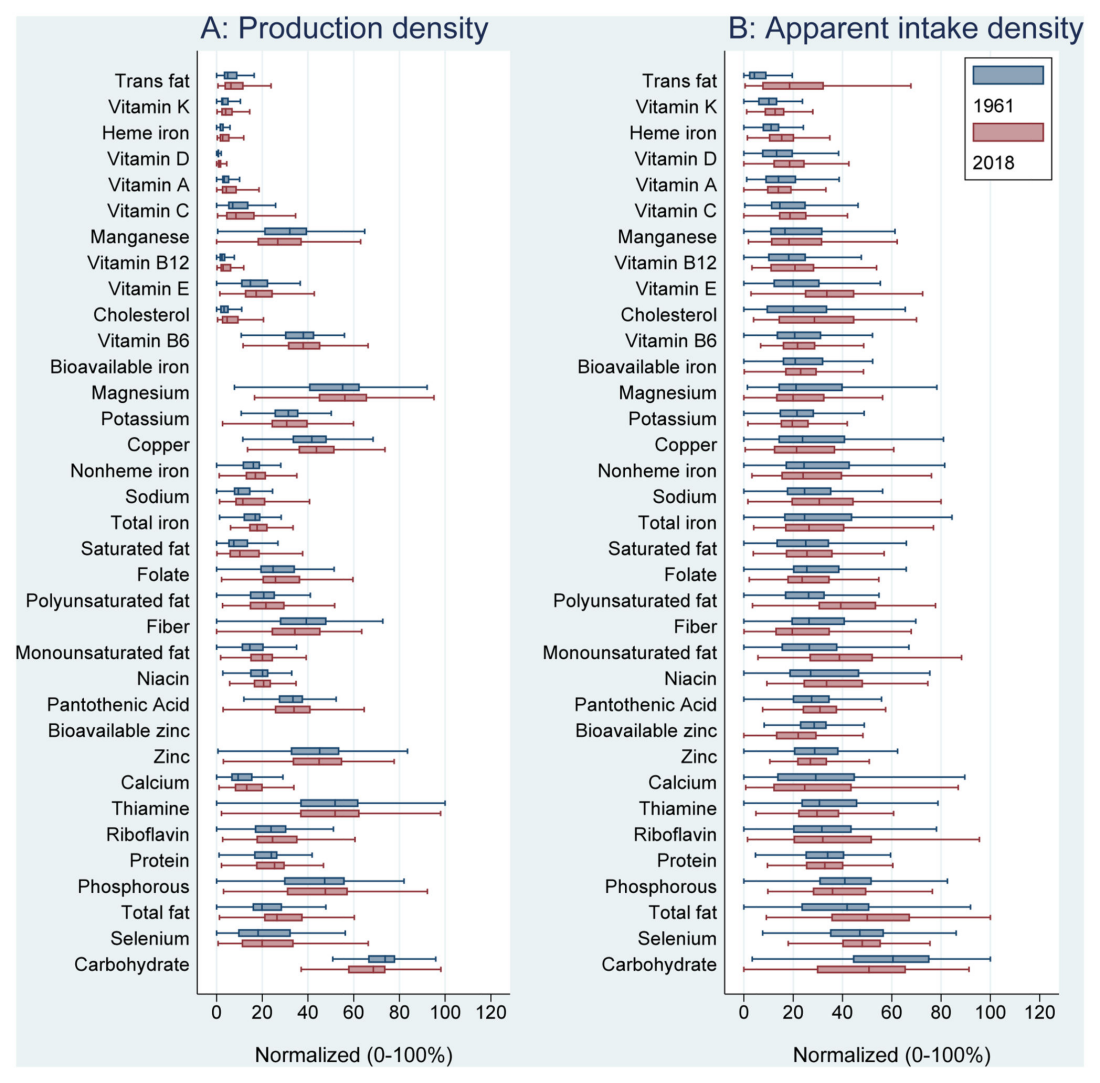
Nutrient density of dietary energy produced and apparent intake, 1961 and 2018 Annual per capita nutrients from production and apparent intake were calculated for each country, then expressed as nutrient densities per unit of dietary energy (nutrients/kcal) and normalized as percentages from 0 to 100 of the global minimum and maximum observed in 1961 and 2018 combined, and for production and apparent intake densities separately: (value - min)/(max - min) * 100%. Values outside box whiskers are removed. Boxes are sorted by 1961 median normalized apparent intake density. Bioavailable iron and zinc were not calculated for production.

Figure 2 shows the density of each nutrient per unit of dietary energy, for agricultural production density and apparent intake in 1961 and 2018. Values are normalized as above, showing each observation as a percentage of each nutrient’s maximum value. While the nutrient density of production supply has increased for most nutrients, the median has declined for several nutrients including manganese, potassium, and fiber while rising significantly for total, saturated and monounsaturated fats. Higher energy has led to lower median nutrient density of apparent intake for many essential nutrients including magnesium, potassium, copper, nonheme iron, folate, fiber, zinc, calcium, thiamine, phosphorous, protein and carbohydrate.

### Production and intake relative to requirements

**Figure 3:**
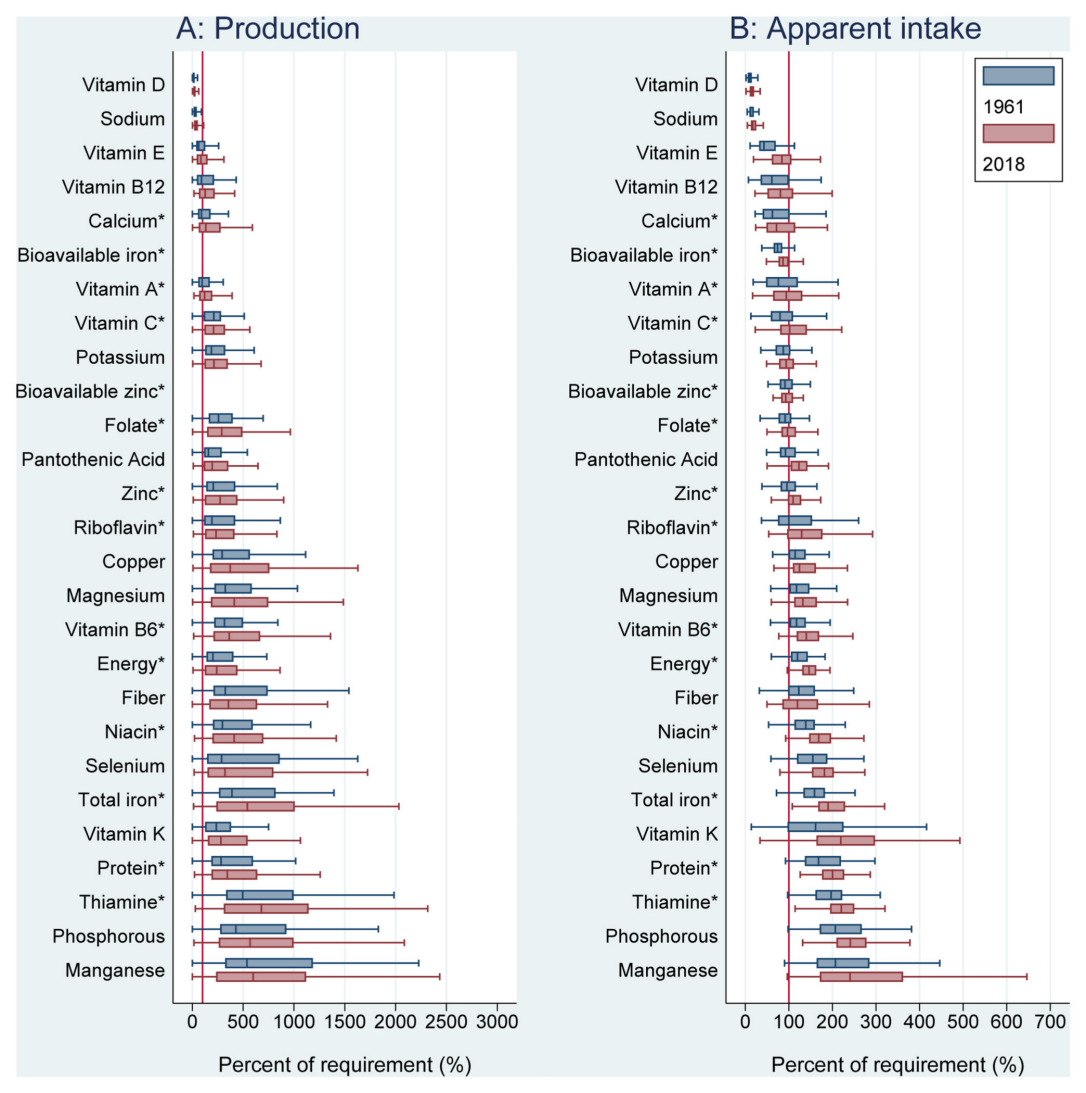
Nutrient adequacy of food production and apparent intake, 1961 and 2018 Annual per capita nutrients from production and apparent intake were calculated for each country, then compared to total national requirements based on EFSA Dietary Reference Values (DRVs) for the country’s population in each 1-year age and sex group using annual country demographic composition data available from UN DESA World Population Prospects, 2019 revision, with additional calculations for each country’s fraction of women who were pregnant or lactating. Annual values were then expressed as percentages of weighted per capita requirements: (pcNBS value/pc DRV) * 100%. Values outside box whiskers are removed. Boxes are sorted by 1961 median apparent intake as a % of requirements. Bioavailable iron and zinc were not calculated for production. Asterisks (*) denote needs based on average requirement (AR).

Figure 3 shows per capita production and apparent intake of nutrients expressed as percentages of per capita requirements. A solid red line indicates 100% of per capita European Food Safety Authority (EFSA) requirements (either Average Requirement (AR) or Adequate Intake (AI) if no AR exists), weighted by national population demographics. Agricultural production for many nutrients is geographically concentrated, reaching 3000% (30x) of national requirements for some nutrients, and then international trade as well as losses and non-food uses lead to more equal apparent intake for which the highest value is around 700% (7x) of requirements. With the exception of dietary fiber, apparent intake relative to requirements has increased for all nutrients since 1961; however, the median percent of requirements remains lower than 100% for several essential nutrients including vitamins D, E, A, B12, and C, and sodium, calcium, iron, potassium, zinc and folate.

**Figure 4:**
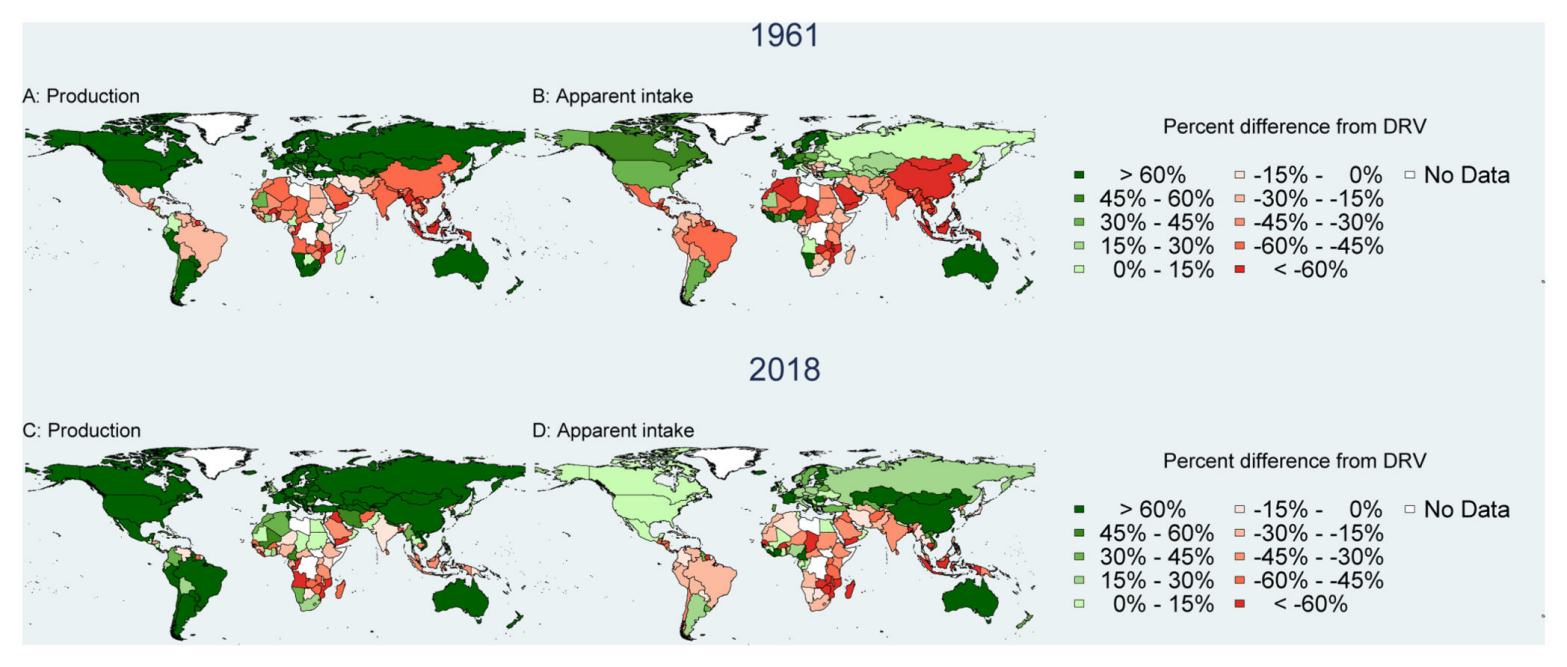
Vitamin A adequacy of national food production and apparent intake, 1961 and 2018 Annual per capita quantity of vitamin A from food production and apparent intake was calculated for each country, then compared to total national average requirements (AR) based on EFSA Dietary Reference Values (DRVs) for the country’s population in each 1-year age and sex group using annual country demographic composition data available from UN DESA World Population Prospects, 2019 revision, with additional calculations for each country’s fraction of women who were pregnant or lactating. Annual values were then expressed as percentages of weighted per capita requirements in each year: ((pcNBS value - pc AR)/pc AR) * 100%.

**Figure 5:**
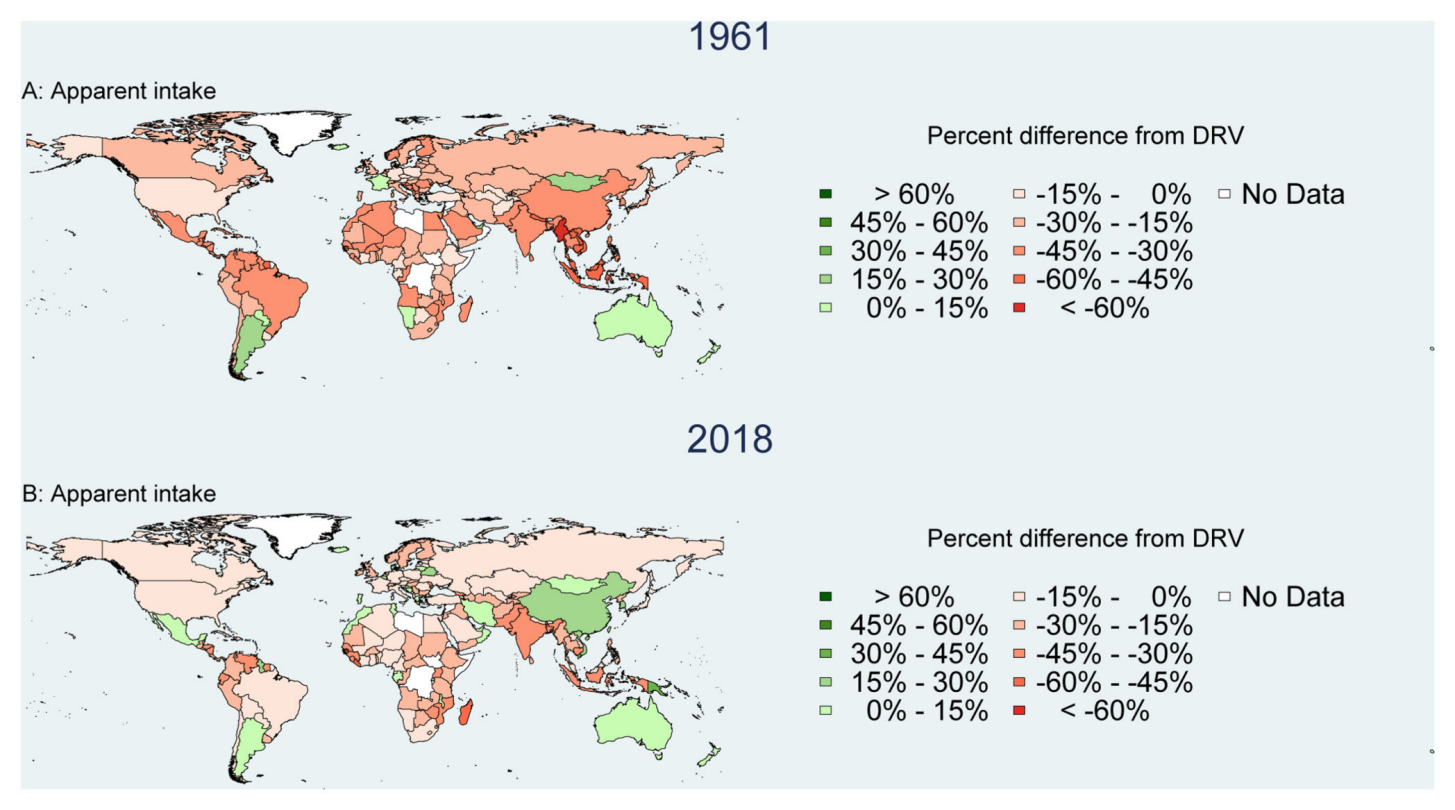
Bioavailable iron adequacy of national food production and apparent intake, 1961 and 2018 Annual per capita apparent intake of iron was calculated for each country, with bioavailability calculated using the algorithm of Rickard et al. (2009) and compared to physiological requirements based on EFSA Dietary Reference Values (DRVs) of the country’s population in each 1-year age and sex group. Demographic composition is from UN DESA World Population Prospects, 2019 revision, with additional calculations for each country’s fraction of women who were pregnant or lactating in each year. Apparent intake from the NBS is then expressed as a percent of weighted per capita requirements in each year: ((pcNBS value - pc DRV)/pc DRV) * 100%.

**Figure 6:**
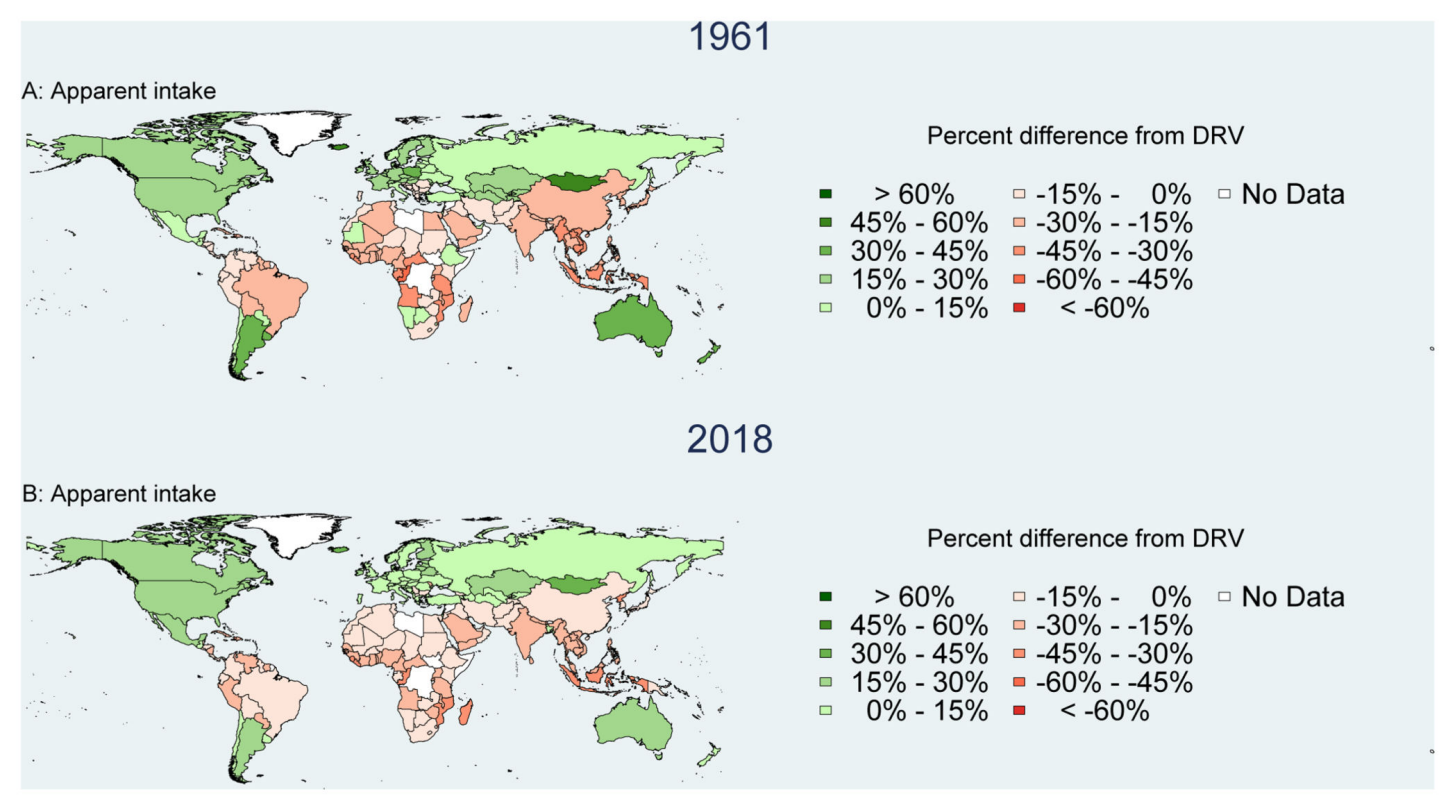
Absorbed zinc adequacy of national food production and apparent intake, 1961 and 2018 Annual per capita apparent intake of zinc was calculated for each country, with absorption calculated using the algorithm of Hambidge et al. (2010), then compared to physiological requirements based on EFSA Dietary Reference Values (DRVs) of the country’s population by 1-year age and sex group. Demographic composition is from UN DESA World Population Prospects, 2019 revision, with additional calculations for each country’s fraction of women who were pregnant or lactating in each year. Apparent intake from the NBS is then expressed as a percent of weighted per capita requirements in each year: ((pcNBS value - pc DRV)/pc DRV) * 100%.

There is particular interest in vitamin A, iron and zinc, for which deficiencies are known to be direct causes and risk factors of illnesses such as anemia, measles, lower respiratory infections and diarrhea that impose significant disease burdens especially for children.^33^ Figures 4-6 show NBS results for the gap in percent of requirements for agricultural production and apparent intake in 1961 and 2018 for these three nutrients, after adjusting iron and zinc for bioavailability. Countries where total production or apparent intake is below total requirements are shown in shades of red, while countries where production or apparent intake exceeds requirements are shown in shades of green. For vitamin A, we see that production exceeds requirements to a greater degree than apparent intake, due primarily to food loss and waste (Figure 4). Both production and apparent intake relative to requirements have increased from 1961 to 2018, particularly in Central and South America, and East Asia. Gains have also been made in Africa and South Asia, but many countries still have total production and apparent intake below total requirements.

Figure 5 shows a very different picture for apparent intake of iron, which is adjusted for bioavailability using the Rickard et al.^34^ algorithm. Figure 5 shows that apparent iron intake is below total physiological requirements for absorbed intake for even more countries than the Vitamin A deficits shown in Figure 4. As before apparent intake relative to requirements has improved in almost all countries, notably in China which shifted from apparent intake 37% below to 24% above its total requirements. Supplemental Figure 2 shows the importance of adjusting for bioavailability, as total iron often exceeds requirements but much of this is from sources that are not bioavailable.

Figure 6 shows that zinc deficits are less widespread than the iron deficits shown in Figure 5 but are more geographically concentrated especially in 2018. As for iron, adjusting for bioavailability^35^ and comparing with physiological requirements for absorbed intake is important as shown also in Supplemental Figure 2. Finally, results for other nutrients indicate that significant gaps remain for some nutrients supplied by animal source foods including calcium, riboflavin (B2), pantothenic acid (B5), and B12, but also for folate (B9), vitamin C, potassium and vitamin E (see Supplemental Figures 2-9).

### Can existing interventions fill nutrient intake gaps?

Adequacy of national nutrient supplies as measured by the NBS is a necessary but not sufficient condition for adequacy of individual intakes. Increasing micronutrient availability to meet or exceed requirements without exceeding dietary energy needs would require rebalancing production and trade towards more nutrient-dense foods, as well as loss and waste reduction for those foods and also biofortification to increase nutrient density of staples. The NBS is designed so that researchers and policy analysts can readily monitor and simulate that kind of change, tracing the flows of all nutrients produced and delivered through crop and livestock products^36^. The NBS also allows calculation of how much additional nutrients from non-food sources would be needed to fill remaining gaps, such as vitamin A, iron or zinc which are commonly added as fortificants during food processing or distributed to individuals as supplements.^37–39^

To illustrate how the NBS can be used to compute the magnitude of intervention needed to fill gaps in supply, Table 2 shows the number of vitamin A supplementation capsules required to prevent vitamin A deficiency in children 6-59 months (C6-59) living in countries where total per capita apparent intake was less than per capita vitamin A requirements. Calculations are based on either (A) the total number of 100K IU (30,000 mcg) and 200K IU (60,000 mcg) capsules required to meet annual C6-59 requirements or (B) the protocol that specifies that infants 6-11 months receive one 100K IU capsule annually and that children 1-4 years receive two 200K IU capsules annually, i.e., one every 6 months^40^. In 1961, between 381 (A) and 523 (B) million vitamin A supplements were required for 274 million C6-59, while in 2018, between 489 (A) and 654 (B) million supplements were required for 346 million C6-59 among deficit countries.

Table 2 also shows the amount and percent of wheat flour (WF) fortification required to prevent risks of inadequate vitamin A, iron and zinc intake in deficit countries. Whereas in 1961, fortifying 100% of domestic WF supplies with 3 mg/kg retinol would not have been sufficient to close the gap in vitamin A intake requirements in deficit countries, in 2018 fortifying approximately 40% of domestic WF supplies among deficit countries was required. For iron, in 1961, fortification of 100% of WF supplies using sodium-iron EDTA (NaFeEDTA) with an assumed bioavailability of 10% would have been required to close the iron intake gap, while in 2018, 50% of WF supplies were required. While not shown, use of a less bioavailable form of iron such as ferrous fumarate (5% bioavailability) would require fortifying 100% of supplies. Finally, fortifying 100% of WF supplies with 30 mg/kg zinc oxide would not have been sufficient to close the zinc intake gap in 1961, whereas in 2018 fortification of 40% of WF supplies was required.

## Discussion

The NBS provides complete accounting of nutrient flows in the world food system, applied in this study to track farm production from crops and livestock through transport and processing to the end-use of each nutrient, compare quantities produced and consumed to physiological requirements of each population by age and sex; and quantify changes needed to provide adequate total supplies for distribution to populations at risk of deficiency.

While median per capita production and apparent intake of all nutrients have increased since 1961, significant global inequalities remain as shown earlier for selected nutrients by Bell et al.^41^ Tracking all nutrient flows from production to apparent intake reveals the extent to which food production, trade and non-food uses allows human requirements to be met in each country, revealing the magnitude of need and opportunities to equitably address differences in national food supplies.^42^

For example, the NBS reveals how global median dietary heme iron density has increased since 1961, as a result of rising use of animal source foods which can be desirable for many reasons but also raises important concerns regarding climate change and the environment as well as animal welfare^43,44,45^. Using the NBS could help decisionmakers address these tradeoffs, with policies that help farm families use livestock in ways that improve food and nutrition security and reduce poverty^46^, while also limiting environmental harms and disease burdens from excess consumption^33^. The NBS also reveals how policies and programs might improve access to specific nutrients most cost-effectively through changes at each stage of supply, complementing local production of micronutrient-rich foods with international trade and changes in food composition such as biofortification^47^.

While most micronutrient needs are or can be met through a balanced diet using locally available items from diverse food groups, important gaps remain and can be filled using nutrient-specific interventions such as fortification and supplementation. For example, the NBS reveals how the total supply of vitamin A available from apparent intake of all foods is insufficient to meet per-capita requirements in 90 countries of the world, where there were 346 million children aged 6 to 59 months in 2018. Meeting their requirements would require up to 654 million capsules, while an estimated 500 million vitamin A capsules were actually distributed in 2007^48^. UNICEF and its partners prioritized 82 countries for national vitamin A supplementation programs from 2000-2017 with coverage peaking in 2009 at 78% and 290 million children,^49^ but coverage has since slipped to 41% among 64 priority countries^49,50^.

In some cases the micronutrient density of food can be increased, using either biofortication on the farm or industrial fortification at the point of processing. For example, wheat as well as vegetable oil, rice and maize can be industrially fortified to fill deficits in several micronutrients, but many countries do not yet have mandated standards that could improve outcomes. Of the countries identified in the NBS as having deficits in their food supply of vitamin A (90 countries), iron (129), and zinc (111), a total of 16, 28, and 25 countries respectively do not currently have wheat flour fortication programs that could help meet those requirements^51^.

The NBS is also useful to reveal changes over time and disparities across countries in the nutrient density of their overall food supplies and national dietary patterns. Most importantly, we showed that for 12 distinct micronutrients, global production and apparent intake has risen less quickly than total dietary energy per capita. Reduction in the nutrient density of global food supplies for those 12 micronutrients is in large part due to the rising share of dietary energy provided by vegetable oils, including palm oil, which has both environmental^52^ and dietary^53^ implications. Accounting for nutrient flows as in the NBS could help future policies address the tradeoffs raised by this and other changes associated with the dietary transition towards more packaged food and food away from home^54,55^.

The principal strength of our approach is in its replicability with explicit equations so that NBS results can readily be updated and expanded to account for additional sources and losses of nutrients as new data become available. By accounting for all nutrient flows implied by existing data, the NBS reveals the implications of those data for nutrient adequacy, and points to the additional disaggregation or improved data collection that would be most helpful. Complete accounting is also a necessary foundation to quantify the magnitude of policy or program changes needed to meet human development goals, including simulation models that would track behavioral responses within environmental constraints.

The limitations of NBS accounting derive from both its structural specification and its data sources. The initial NBS introduced in this study aims to account for naturally occurring nutrients in the food supply, omitting non-food sources that would be addressed in future studies such as sodium from table salt, vitamin D synthesis from sunlight, and calcium or other minerals absorbed from drinking water, in addition to the industrial fortification and supplementation strategies discussed above. Similarly, the initial NBS used here does not account for all processing and cooking losses and only accounts for bioavailability of iron and zinc. Additional equations could readily be added as additional data becomes available, for example regarding differences in nutrition composition of foods at the point of production, changes in nutrient composition caused by food processing (including fermentation), and additional bioactive compounds beyond the 37 variables included here. Future versions of the NBS could also disaggregate apparent intake by population group, using survey data to quantify disparities within countries in addition to the cross-country inequities addressed in this study.

A fundamental constraint for monitoring nutrient balances is the accuracy of data on food composition^56^. Food composition data remains limited for many reasons, including the high cost and technical difficulty of measurement using gold standard laboratory techniques. Use of the NBS and other efforts to improve the nutrient density of available foods could raise demand for lower-cost techniques, and support more frequent measurement of food composition in a wider range of settings. More testing would reveal the degree of naturally occurring or industrially-caused variation in the nutrient composition of each food, as well as differences among the thousands of differentiated products reported in dietary surveys.

In this study we compare national average nutrient levels using the best available data against two widely used benchmarks: FAO’s own accounting of total macronutrients per capita, and the GDD’s estimates of mean micronutrient intake. Our NBS results are highly correlated with variation in the FAO data, but systematically lower due to the NBS having accounted for a variety of food losses not included in FAO estimates. Comparing to GDD estimates, we find low correlation: 15 of the 20 nutrients compared had correlation coefficients below 0.5, and five had correlation coefficients below 0.2. This finding is partly due to the fact that GDD estimates are smoothed across countries and over time, replacing survey observations and missing data with Bayesian inference, and partly due to limitations of the surveys themselves. Comparing these data sources underscores their different purposes, as survey data and the GDD is particularly useful for disaggregation within countries, while the FAO and the NBS aim for complete accounting of aggregate flows to and from each country.

A central purpose of the NBS, beyond the FAO’s own food balance sheets, is to track nutrients lost in food transformation and meal preparation. Recent estimates suggest that roughly 14% of food production is lost post-harvest prior to the retail stage^57^ and that an additional 17% of food is wasted at the retail and household levels^58^. In this study regional FLW was applied to food and nutrient intake^59^. More initiatives are currently under way to better assess FLW such as FAO’s food loss index and food waste index that quantify FLW using various country-level surveys^60^. Given FLW has important implications for both human and environmental health, future versions of the NBS should be updated as new estimations for FLW become available.

In summary, using the NBS to track national total nutrient flows is an important complement to survey data such as food frequency questionnaires, 24HR quantitative dietary recall surveys, Demographic and Health Surveys (DHS) focused on maternal and child wellbeing, or Household Consumption and Expenditure Surveys (HCES) focused on livelihoods and food acquisition^61,62^, as well as micronutrient surveys and health examination surveys that include biomarkers and other clinical measures of nutritional status.^24^ Each individual kind of survey and every data collection method has its own strengths and limitations, with differences in the degree to which they provide accurate estimates of national totals, within-country differences, or other statistics of interest for each study.^63^ The highest quality surveys will remain costly and scarce relative to lower-quality surveys and aggregate data such as the FBS, calling for continued efforts at cross-validation between different sources of information to inform national policymaking and intervention.^64^

## Methods

### Nutrients of interest

The nutrients selected for inclusion in the NBS are shown in Supplementary Table 1. Data from the Nutrient Database for Standard Reference Legacy database (NDB) available at Food Data Central were used to inform the nutrient composition of foods^65^. From the available nutrients in the NDB, primarily essential macro and micronutrients for which there are Dietary Reference Values (DRV) and/or Reference Intake Ranges (RIs) were selected, while simple sugars, sugar alcohols, alternative forms of vitamins A, D, E and K, provitamins A, amino acids, polyphenols, and specific fatty acid chains were excluded. These nutrients could be added in future iterations following these methods. Two nutrients, commonly referred to as antinutrients because of their inhibitory effect on micronutrient bioavailability, were also included (Supplementary Table 1). Phytate data were obtained from PhyFoodComp1.0^66^. Polyphenol values for tea were obtained from Table A1 from Hallberg and Hulthén^67^.

### Country definitions and population data

FBS data were obtained from FAOSTAT^68^. Daily per capita quantities of each FBS element (i.e., production, imports, etc.) were created using the population data supplied in the FBS download (see Supplementary Formula 1). Per capita quantities from former countries such as the Soviet Union were also applied to their current counterparts for applicable past years, and corresponding population estimates for those regions were imported from UN DESA World Population Prospects (2019 revision) for the applicable former years^69^. International Organization for Standardization (ISO) ISO2 and ISO3 codes and United Nations standard country and area codes for statistical use were applied to each country. Countries were then grouped into seven regions based on World Bank regional definitions^70^: Sub-Saharan Africa, South Asia, East Asia and the Pacific, Middle East and North Africa, Latin America and the Caribbean, Europe and Central Asia, and North America. Countries were also grouped based on the World Bank income classification system, updated for June 2020^70^. Per capita GNI, calculated using the World Bank Atlas method and updated May 2021^71^ was also merged into the database for each year available.

### Food categories and nutrient composition

#### Food Classification

FAO’s FBS consists of 98 food categories constructed from 456 Supply and Utilization Accounts (SUAs) for individual food products^72^. Each SUA has a Central Product Classification (CPC) system code, updated for CPC v2.1. The five-level CPC consists of internationally agreed-upon definitions and is used for data and statistics related to agricultural production, international trade, payment balances and prices important for analysis and policymaking^73^.

CPC v2.1 contains revisions necessary for maintaining consistency with other economic accounting systems^74^. One such system is the Classification of Individual Consumption According to Purpose (COICOP) 2018^75^. COICOP 2018 is part of the System of National Accounts (SNA) and relates the SNA to household expenditure through budget surveys and is important for assessments of living standards and calculation of various social economic statistics^75^. COICOP 2018’s 4- and 5-level structures for up to 269 food products are closely related to CPC v2.1 in that expenditures on products form the basis of the COICOP classes^75^. Ultimately these standardized systems make it possible to produce comparable food and nutrient availability estimates across countries.

To construct the FBS, FAO transforms food product quantities from the 456 SUAs back into primary equivalent commodity forms using conversion factors^2^. At the time of this study, SUA data on individual food products were not available through FAOSTAT and currently, SUA data are available only for years 2014-2018. Only the list of food products making up the 98 food categories is publicly available.

Supplementary Table 2 shows the NBS food categories and relationships to FBS categories and food products. SUAs, here called Food Products (FPs) are organized into 98 FBS categories, simply referred to as Food Categories (FCs). FCs are organized into 21 FBS food groups, here referred to as Food Subgroups (FSGs). One FC within each of 14 FSGs deemed ‘Other’ (Food Category Other or FCO) is devoted to additional FPs that are not covered among the other FCs within each FSG. Four overarching groups were developed for the NBS, called Food Groups or FGs. To construct the FGs, each of the 98 FCs was classified into one of four FGs based on collapsing the Minimum Dietary Diversity-Women (MDD-W)^76^ index into four groups: Starchy Staples; Nutrient-dense Vegetal Foods; Animal Source Foods; and Other Foods. Dummy variables were then created to additionally identify fruits and vegetables (FV) and sources of heme iron (HSI)^77^.

For each of the variables FV, HSI, FG, FSG, and FC in Supplementary Table 2, the NBS and/or FAO code is provided. For each FP, the CPC v2.1 code is provided as well as the authors’ assumed relationship to a COICOP 2018 code. Within each FC in Table 2, the FP (or FPs) that is used as the primary commodity is shown in bold/italicized font- this is the food form requiring the food composition table (FCT) match. Within each FSG, the FCO is shown in italics. The FCO typically contains multiple primary commodities. In addition, within each FCO is an FP deemed ‘Not Elsewhere Specified’ (NES) additionally shown in Supplementary Table 2 with an asterisk. NES are a set of additional FPs^78^.

FAOSTAT does not provide FP SUA data or definitions for fish and seafood which were informed using the FAO/Coordinating Working Party on Fishery Statistics (CWP) Handbook of Fishery Statistical Standards, Annex S.II: International Standard Statistical Classification of Aquatic Animals and Plants^79^. Lists of fish species within these groups were obtained from the FAO software FishStatJ^80^. FishStatJ provides information on FAO’s Fisheries and Aquaculture statistics and provides data sets on production, trade, and consumption.

#### Nutrient Composition

Schematic development of the NBS is shown in Supplementary Figure 1. To construct the FCT for the NBS, NDB food item matches were selected for the primary commodity FPs and organized by FC (Supplementary Figure 1F). Nutrients were expressed in terms of 100g of edible portion of the food. Several food items required additional consideration. The West Africa Food Composition Table 2019^81^ was used for palm kernels and to distinguish pale flesh sweet potato varieties commonly consumed in developing countries from orange flesh sweet potato varieties that are generally consumed in high income countries^82^. Next, in Africa palm oil is often consumed as unrefined, red palm oil. In Nigeria roughly 70% of palm oil produced is consumed without refining^83^. Therefore, we assumed that 70% of palm oil production in Sub-Saharan African countries of the tropical rain belt that is retained for domestic consumption is consumed as red palm oil, with vitamin A levels from the WAFCT 2019. All other palm oil, including exports from African tropical rain belt countries, production from other countries, and all other imports and exports are assumed to be as refined palm oil. For Bangladesh, NDB food item matches for rice were limited specifically to parboiled, polished white rice (the form primarily consumed in Bangladesh). For all other countries, additional matches for rice (e.g., brown and/or non-parboiled) were incorporated into the FCT.

For phytate, food items were selected from PhyFoodComp1.0^66^ that matched the primary commodity FPs for each FC and the phytate value from the primary assay specified in PhyFoodComp1.0 was used. If no assay was specified, the phytate value was prioritized in order of inositol hexaphosphate (IP6), IP6 + inositol pentaphosphate (IP5) or back-calculation using the value of phytate phosphorous and its conversion factor. PhyFoodComp1.0 yielded phytate values for 13 NBS food categories. Phytate for palm kernels was informed by Wessels et al.^17^ All other categories were assumed to have phytate values of zero. Polyphenol values for tea obtained from Hallberg and Hulthén^67^ were specified as tannic acid equivalent per 100g of dry matter and taken as the average of green and black tea. Finally, an additional FCT was created to match wheat, maize, millet, and sorghum flours to their corresponding FBS categories.

#### Uncertainty in the composition of unclassified foods

FCOs contain more potential FCT matches including NES foods than do other FCs, thus creating the potential for greater uncertainty in the estimation of nutrient supplies from the FCOs. To understand how this might impact NBS estimates, the quantity and proportion of foods reported in FCO categories were calculated and sensitivity analysis using Monte Carlo methods were used to assess the uncertainty of nutrient estimation within the NBS.

#### Refuse factors

Refuse values were accessed from the USDA National Nutrient Database for Standard Reference, Release 28^84^ (Supplementary Figure 1G). FAOSTAT specifies the dressed carcass which is ready for cutting into individual ‘bone-in’ retail cuts such as chuck, rib, loin, etc., in the case of beef as the primary commodity for meat. We therefore selected ‘bone-in’ retail cuts and missing values for beef (33.3%) veal (31.4%) lamb, mutton and goat (34.3%) and pork (27.1%) were obtained from the USDA ERS agricultural handbook^85^. The average refuse value obtained from the values for beef, pork, veal, lamb/mutton, and poultry was then used for the FBS categories ‘Aquatic animals, others’ and ‘Aquatic mammals.’ Refuse values for fish and seafood were obtained from the FAO/CWP Handbook of Fishery Statistics, Annex 1.1, for fish species (using ‘skin off’ conversion factors from the table titled ‘Fillets’) as well as crustaceans, cephalopods, and mollusks (from the table titles ‘Crustaceans and Molluscs’)^86^.

#### Nutrient retention in processing

Processing of several foods was accounted for. The quantity of cereal grain processed into flour in each country was calculated for wheat, maize, sorghum, and millet (Supplementary Figure 1D and 1E) based on regional proportions compiled by Wessels et al.^17^ Extraction rates were used to determine the quantity of flour available for consumption: 72% for straight-grade wheat flour (the primary product of most flour mills); 100% for whole wheat flour^87^; 70% for refined maize flour^88^; 74% for refined millet^89^; 79% for refined sorghum^89^; and 100% for whole maize, millet and sorghum flours (personal communication: Victor Taleon, HarvestPlus/IFPRI). The total quantity of cereal consumption of wheat, maize, sorghum, and millet was taken as the resulting quantities of refined and whole grain flours.

Several additional commodities required processing retention rates to convert from primary commodity forms to processed forms for which there is FCT information (Supplementary Figure 1G). These include coffee (12.92)^90,91^, tea (119.05)^92^, cocoa (0.82)^93^, and sugar cane (0.11) and beat sugar (0.15)^2^.

#### Food loss and waste

Food loss and food waste (FLW) percentages by food group and region were informed by the Swedish Institute for Food and Biotechnology (SIK) (Supplementary Figure 1H and 1I)^94,95^. Percentage post-harvest loss (for fish and seafood only), retail waste and consumption waste were included^95^. The SIK excluded the following FBS food groups: Sugar Crops, Sugar and Sweeteners, Pulses, Treenuts, Vegetable Oils, Stimulants, Spices, Alcoholic Beverages, Animal Fats and Infant Foods.

#### Nutrient retention in cooking

A nutrient retention FCT for cooking was created by categorizing the USDA nutrient retention factors^96^ by FBS food category (Supplementary Figure 1H and 1I). The matches included various types of preparation, such as boiling, steaming, roasting, etc. For FBS categories with no resulting nutrient retention matches or for nutrients not identified in the database, values of 100% were assumed.

### Creation of standardized nutrient variables

#### Calculation of per capita nutrient values

The FCT was first collapsed by FC using the mean value of category matches and then merged into the NBS by FC. Per capita nutrient values of all FBS elements (i.e., production, imports, etc.) were calculated for all observations (Supplementary Figure 1J) by multiplying the daily per capita quantity of each element (in grams) by the edible portion (1 - minus the refuse proportion), processing retention rate and nutrient composition per 100g for each nutrient separately and by FC, country, and year (see Supplementary formulas 2-5b).

#### Calculation of variables that account for nutrient losses

Three additional variables were calculated that account for nutrient losses (Supplementary Figure 1J). The first variable accounted for FLW and nutrient retention from cooking only. The second variable accounted for the nutrient retention from cereal processing only. The third variable, deemed the final best estimate of apparent intake, accounted for all three additional nutrient losses. See Supplementary formulas 6a-8c for specific calculation of these variables.

Macronutrient estimates from the second and third additional variables described above were compared with FAO’s macronutrient availability estimates. Per capita energy, protein and fat estimates for each variable were aggregated to the country level, and scatterplots were created and compared at the overall country level and by country. Pearson correlations were calculated for the country-level comparisons. The percent differences between the final best NBS estimates and FAOSTAT values were also calculated.

Fixed effects regression methods were used to explore the reasons for the differences between NBS and FAOSTAT estimates for energy, protein, and fat using the closest comparison of availability. For each macronutrient outcome variable, the percent difference was calculated as (NBS – FBS)/FBS*100%. Each regression explores whether differences were associated with relative size or income levels of countries, differences in geographic region, quantities of particular food groups, specific food categories, reporting of imported foods, differences between the per capita food variable calculated for the NBS and the post hoc calculation of per capita food reported separately to the FBS, or differences in the food composition values between the NBS and FBS.

### Calculation of iron and zinc bioavailability

The amount of dietary iron absorbed from food depends on an individual’s level of iron depletion^97^, the amount of bioavailable iron in food from different sources, and the presence of other dietary factors that enhance or inhibit absorption^98^. For nutrient accounting purposes, the NBS disaggregates total iron into heme and non-heme sources, counting 40% of all iron in heme-containing foods as heme iron^77,99^.

Because consensus has not yet been achieved on one suitable iron bioavailability algorithm, iron availability was determined by adapting each of three of the latest algorithms: a food group-based algorithm developed using a regression equation by Conway et al.^100^; an algorithm based on test meal data that also accounts for the non-linear nature of absorption, developed by Rickard et al.^34^; and an algorithm based on complete diets developed by Armah et al.^97^.

Using each algorithm, the percent non-heme iron bioavailability was determined. Twenty-five percent heme iron bioavailability was assumed^34^. Bioavailable heme and non-heme iron were then determined algebraically, and total bioavailable iron was calculated as the sum of bioavailable heme and non-heme iron. The Armah algorithm requires information on serum ferritin; a constant value for serum ferritin corresponding to a reference dose absorption of 40% (21.7 mcg/L of serum ferritin) was used for this application as in other studies^67,100^. See Supplementary Figure 1K.

Zinc absorption from national food supplies was also calculated (Supplementary Figure 1K). The updated Miller algorithm^35,101^ was used to calculate the total absorbed zinc (TAZ) in national diets for each year. Finally, estimates of the total percent bioavailability of each country’s national available iron and zinc supplies were determined based on the ratio of total per capita bioavailable supplies to total per capita supplies. See Supplementary formulas 9-17 for specific calculations.

### Country demographics and nutrient requirements

A database of country-wide nutrient requirements called the National Nutrient Requirement Database (or NNRD) was created to link nutrient requirements by demographic groups to the number of persons in each demographic category (Supplementary Figure 1L). Population data were obtained from the UN DESA World Population Prospects, 2019 revision for all years from 1950-2019^69^. Additionally, the number of pregnant women was calculated by adapting the method defined by the CDC^102^ and including data on the number of induced abortions^103^ and miscarriages^104^. The number of lactating women was calculated by multiplying the number of live births by the average length of time for which breastmilk production remains relatively constant, 6 months^105^. The number of infants ages 0-5 months was assumed to be equal to the number of lactating women that are exclusively breastfeeding.

EFSA Dietary Reference Values (DRVs) for nutrients were used to inform the nutrient requirements of each age, sex, pregnancy, and lactation category^106^. Dietary energy and other nutrient requirements were calculated at the minimum physical activity level (PAL) for ages 4 and older. Where available, the Average Requirement (AR) was used as the standard for macro and micronutrients. Additionally, for iron and zinc, the median physiological requirements for absorbed intake were included and used as the standards where bioavailability adjustments for iron and zinc are incorporated. Where an AR did not exist, the Adequate Intake (AI) was used. For protein, EFSA specifies requirements in units of g/kg of body weight. The reference body weights defined in Table 18 of the EFSA summary report on DRVs for nutrients were therefore used^106^. EFSA Reference Intake ranges (RIs) were used for carbohydrates and fats which do not have ARs or AIs. See Supplementary formulas 18-22 for the calculation of nutrient requirements.

### Applications

#### Comparison to the Global Dietary Database

Country-level daily per capita macro and micronutrient estimates from the NBS were compared to results for daily averages across all demographics from the Global Dietary Database (GDD) model; data were provided by the GDD team through direct download^107^. Pearson correlations were calculated to determine the magnitude and significance of the linear relationship between the NBS and GDD while Spearman correlations were calculated to determine the correlation and significance of the relative ranking of estimates between the two databases.

#### Examination of nutrient production and intake

To examine differences between per capita nutrient production and apparent intake between 1961 and 2018, annual per capita nutrient values from all FCs were summed by country and year. To avoid double counting nutrients from production, FCs representing processed foods – vegetable oils, alcohol, butter, cream and fish oils – were excluded from the summation of production. Horizontal boxplots separately showing the distributions of per capita production and apparent intake of each nutrient across countries were then created. Since nutrients differ in their reported units and scales, to improve visibility, each nutrient’s values for 1961 and 2018 were normalized on the same scale separately for production and apparent intake and presented as values between 0 and 100%. Resulting boxplots were then sorted by 1961 median apparent intake.

To examine differences in dietary density between 1961 and 2018, national annual per capita production and apparent intake values were then expressed per 2000 kcal of dietary energy either produced or apparently consumed. Values were normalized as above and again displayed as horizontal boxplots.

#### Comparison of nutrient availability to requirements

Horizontal boxplots of each nutrient relative to requirements were also created. Weighted per capita requirements (using the AR or an AI if no AR exists) were developed for each country and year using data compiled in the NNRD. Annual per capita production and apparent intake values were then expressed as the percent of weighted per capita requirements (pc NBS value/pc DRV) x 100%). Horizontal boxplots were developed as above using a solid red line to indicate 100% of national requirements.

To better show how production and apparent intake of nutrients relative to national requirements have varied among countries and between 1961 and 2018, the percent differences between production and national requirements (and apparent intake and national requirements) were calculated as (pc NBS value – pc DRV / pc DRV x 100%). Resulting percent differences were then grouped into 10 categories each separated by 15 percentage points and displayed as chloropleth maps containing each country of the world using the spmap command in Stata and base maps and shape files obtained from ArcGIS Hub^108^.

#### Examination of vitamin A supplementation and fortification

We examined potential interventions to address vitamin A, iron and zinc gaps in countries with apparent intake deficits relative to requirements. First, the number of vitamin A supplementation capsules required to prevent vitamin A deficiency in children 6-59 months (C6-59) was calculated. The number of children from deficit countries ages 6-11 months, 1-3 years, and 4 years of age were tabulated and their total annual requirements were calculated. Vitamin A supplementation protocol specifies that infants 6-11 months receive one 100K IU (30,000 mcg) capsule annually while children 1-4 years receive two 200K IU (60,000 mcg) capsules annually^40^. The number of supplements required was then calculated, first by dividing the total annual requirements among infants 6-11 mo and children 1-4 years by the number of micrograms of vitamin A supplied in 100K IU and 200K IU capsules, respectively, and second by calculating the number of capsules needed based on the delivery protocol.

Next, the quantity of fortified wheat flour required to fill apparent dietary intake gaps of vitamin A, bioavailable iron (calculated using the Rickard 2009 algorithm) and bioavailable zinc were calculated. For each nutrient separately, among deficit countries, annual deficits were calculated. Next, the quantity of fortified wheat flour expressed in 1000 metric tons (MT) required to fill each gap was determined based on the following fortificants: 3mg/kg retinol for vitamin A; 35 mg/kg sodium-iron EDTA for iron; and 30 mg/kg zinc oxide for zinc. The bioavailability of sodium iron EDTA was assumed to be 10%^109^ while the bioavailability of zinc oxide was assumed to be 30% based on differences between EFSA ARs and physiological requirements for zinc^110^ The resulting values were expressed as percentages of aggregate wheat flour supplies across the deficit countries.

## Supplementary Material

Supplementary Figure 1

Supplementary Figure 2

Supplementary Figure 3

Supplementary Figure 4

Supplementary Figure 5

Supplementary Figure 6

Supplementary Figure 7

Supplementary Figure 8

Supplementary Figure 9

Supplementary information

Supplementary Table 1

Supplementary Table 2

Supplementary Table 3

Supplementary Table 4

## Figures and Tables

**Table 1:** Correlation of NBS with FAO and GDD estimates Total national per capita nutrient values from the NBS were compared with FAO estimates, which are based on Food Balance Sheets and Supply and Utilization Accounts for each country, and compared with GDD estimates of average national nutrient intake, which are based on Bayesian inference from survey data. Pearson correlations (*r_p_*) were calculated to determine the magnitude and significance of each correlation, while Spearman correlations (*r_s_*) were calculated for the correlation and significance of relative rankings between the NBS and GDD estimates.

**Comparison with FAO**				
**Nutrient**	***r*_P_**	**p-value**	***r*_S_**	**p-value**
***Without NBS accounting for loss and waste, cooking or bioavailability***				
Dietary energy	0.94	0.00	-	-
Protein	0.97	0.00	-	-
Fat	0.95	0.00	-	-
***Final best NBS estimate***				
Dietary energy	0.90	0.00	-	-
Protein	0.96	0.00	-	-
Fat	0.95	0.00	-	-
**Comparison with GDD**				
**Nutrient**	***r*_P_**	**p-value**	***r*_S_**	**p-value**
***Final best NBS estimate***				
Carbohydrate	0.77	0.00	0.77	0.00
Riboflavin	0.67	0.00	0.68	0.00
Potassium	0.62	0.00	0.70	0.00
Calcium	0.61	0.00	0.64	0.00
Saturated fat	0.58	0.00	0.55	0.00
Folate	0.43	0.00	0.44	0.00
Protein	0.41	0.00	0.48	0.00
Vitamin D	0.40	0.00	0.49	0.00
Zinc	0.39	0.00	0.46	0.00
Magnesium	0.36	0.00	0.40	0.00
Sodium	0.34	0.00	0.49	0.00
Vitamin C	0.24	0.00	0.34	0.00
Fiber	0.23	0.00	0.20	0.00
Thiamin	0.19	0.00	0.24	0.00
Monounsaturated fat	0.17	0.00	0.21	0.00
Iron	0.07	0.017	-0.0098	0.74
Niacin	0.07	0.028	0.059	0.028
Vitamin B6	-0.010	0.73	0.053	0.077
Selenium	-0.038	0.20	-0.033	0.27
Vitamin E	-0.16	0.00	-0.16	0.00

**Table 2:** Magnitude of deficits and example interventions to fill nutrient intake gaps The number of countries in 1961 and 2018 with apparent vitamin A, iron, and zinc intakes less than requirements are shown and annual aggregate net deficits are displayed. The number of vitamin A supplements required for children ages 6-59 months were then calculated based on either: 1) the total number of 100K IU and 200K IU capsules required to meet annual requirements in children ages 6-59 months; or 2) a delivery protocol where infants ages 6-11 months receive one 100K IU capsule and children ages 12-59 months receive two 200K IU capsules annually. For fortification, the amount of wheat flour fortified with either 3 mg/kg retinol, 35 mg/kg NaFeEDTA (10% bioavailability), or 30 mg/kg zinc oxide (30% bioavailability), required to fill apparent intake gaps was also calculated for vitamin A, iron and zinc, respectively.

Vitamin A (mcg RAE)			
Deficit	Units	1961	2018
Countries	no.	105	90
Apparent intake	mcg (billions/day)	382	1,221
Requirement	mcg (billions/day)	895	1,840
Annual deficit	mcg (billions/yr)	187,200	225,870
**Supplementation**			
Population C6-59 mo	millions	274	346
Total annual VA requirements among C6-59 mo	mcg (billions/yr)	21,237	26,769
Total capsules required annually to meet C6-59 mo requirements	millions/yr	381	489
Total capsules required annually based on delivery regimen	millions/yr	523	654
**Fortification**			
Fortified wheat flour required @ 3 mg/kg retinol	1000 MT/yr	62,400	75,290
Annual wheat flour consumed	1000 MT/yr	46,902	185,211
Percent of wheat flour consumption required to fill deficit	%	133	41
**Bioavailable iron (mg)**			
**Deficit**			
Countries	no.	156	129
Apparent intake	mg (millions/day)	1,947	4,136
Requirement	mg (millions/day)	2,820	5,317
Annual deficit	mg (millions/yr)	318,588	431,072
**Fortification**			
Fortified wheat flour required at 35mg/kg NaFeEDTA & 10% BV	1000 MT/yr	91,025	123,163
Annual wheat flour consumed	1000 MT/yr	94,931	251,657
Percent of wheat flour consumption required to fill deficit	%	96	49
**Bioavailable zinc (mg)**			
**Deficit**			
Countries	no.	115	111
Apparent intake	mg (millions/day)	4,113	13,256
Requirement	mg (millions/day)	5,374	15,679
Annual deficit	mg (millions/yr)	460,101	884,438
**Fortification**			
Fortified wheat flour required at 30mg/kg Zinc Oxide & 30% BV	1000 MT/yr	51,122	98,271
Annual wheat flour consumed	1000 MT/yr	47,484	253,586
Percent of wheat flour consumption required to fill deficit	%	108	39

## Data Availability

All data used in this study are publicly available from open sources as indicated in the references in the Methods section. The NBS and NNRD will be made available at: https://sites.tufts.edu/willmasters/. Inquiries related to the NBS should be made to Keith Lividini, keith.lividini@tufts.edu.

## Code Availability

All data analysis was carried out in Stata SE 15-17. All figures were produced in Stata. The code used for data analysis and figure production is available from the corresponding author upon request and will be made available at https://sites.tufts.edu/willmasters/.
