# Supplementary figures and images for: Tracing global flows of bioactive compounds from farm to fork in Nutrient Balance Sheets can help guide intervention towards healthier food supplies
Source: Nat Food. Author manuscript; Available in PMC 2022 Oct 11. (PMC7613697; doi:10.1038/s43016-022-00585-w)

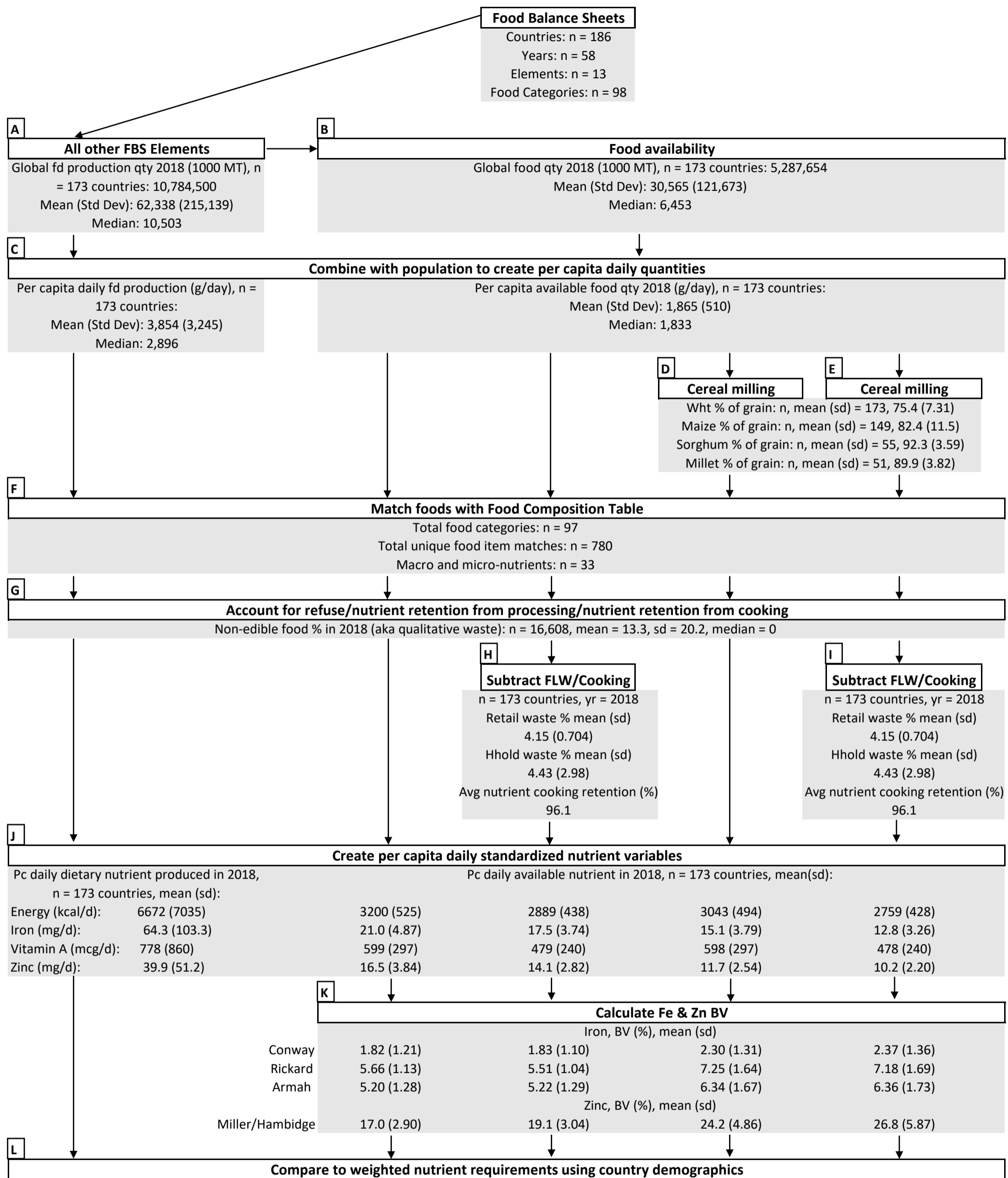

Supplement: Supplementary Figure 1 [file EMS153904-supplement-Supplementary_Figure_1.pdf]

Iron (Fe)

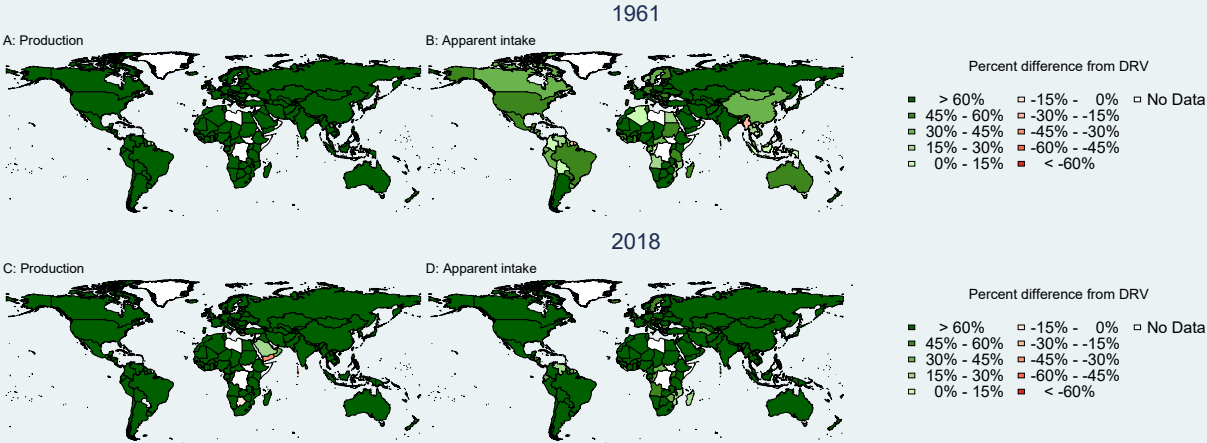

Zinc (Zn)

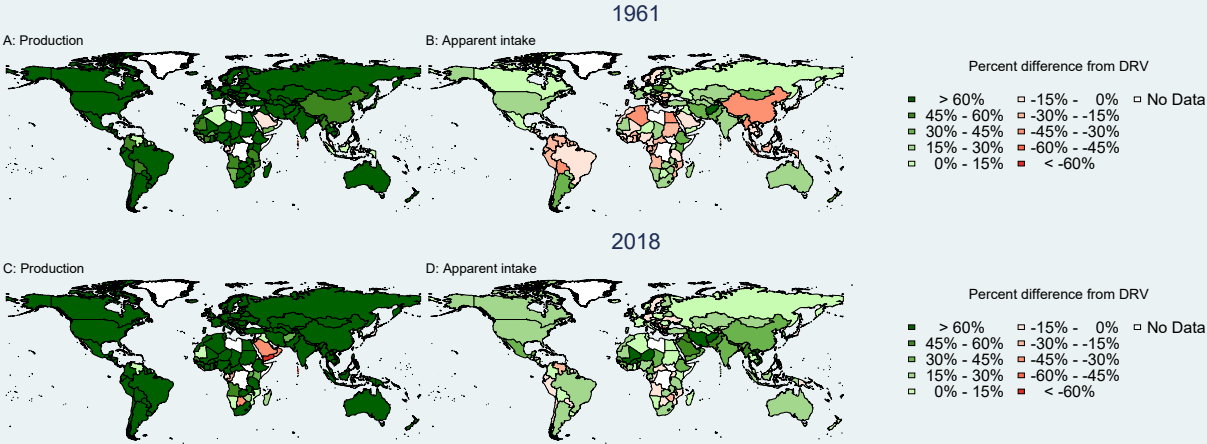

Calcium (Ca)

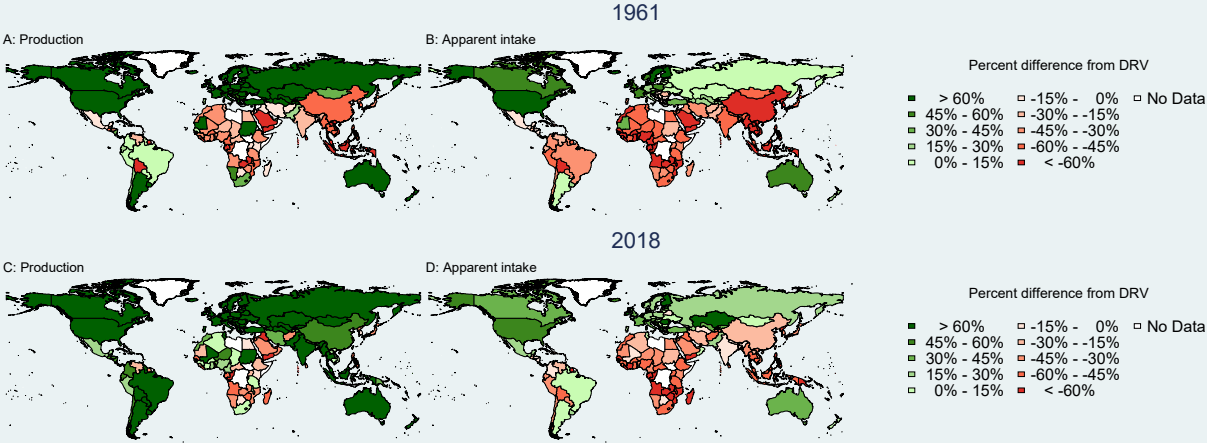

Supplement: Supplementary Figure 2 [file EMS153904-supplement-Supplementary_Figure_2.pdf]

Dietary energy

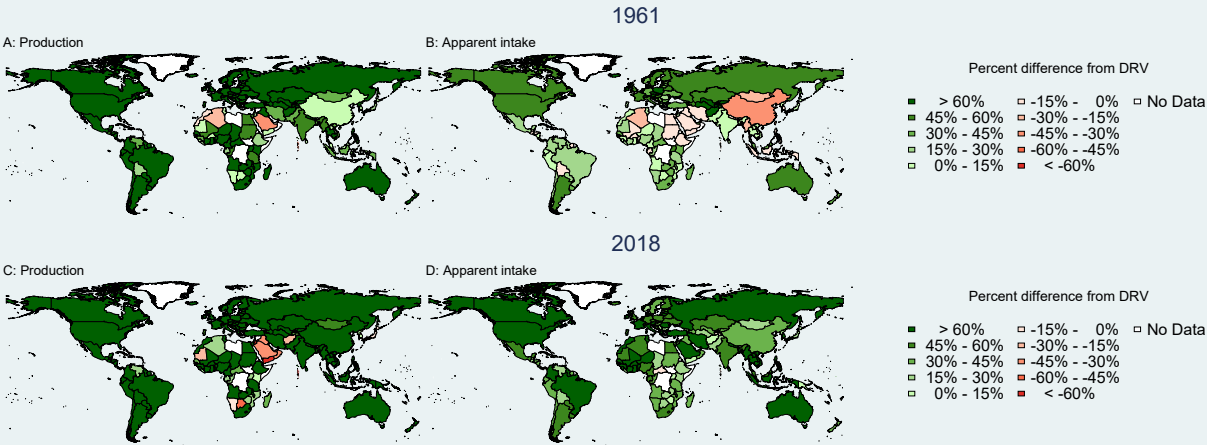

Protein

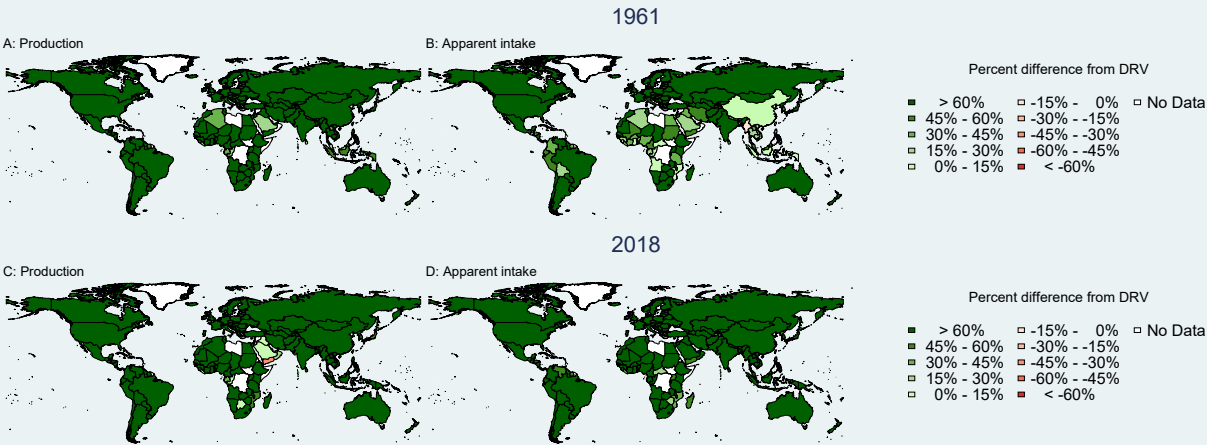

Fiber

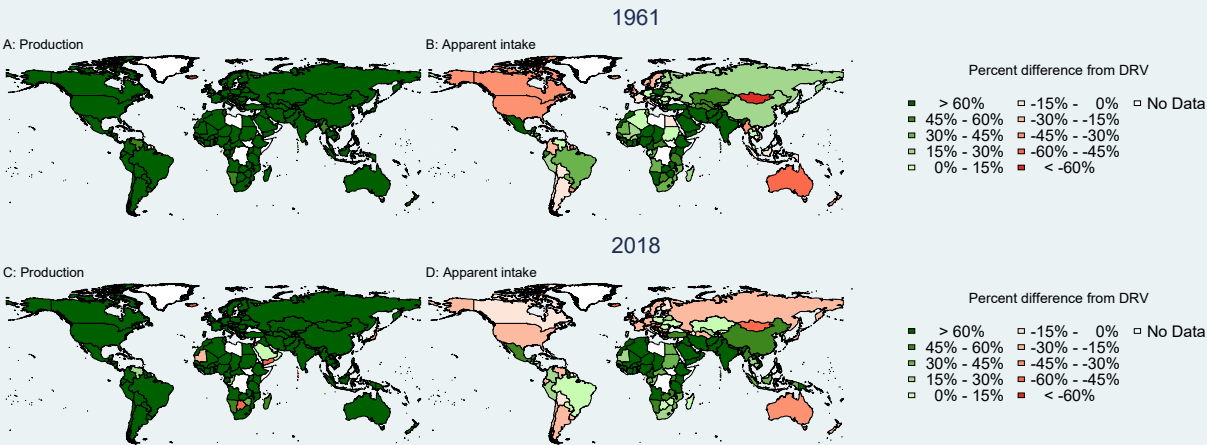

Supplement: Supplementary Figure 3 [file EMS153904-supplement-Supplementary_Figure_3.pdf]

Thiamin (B1)

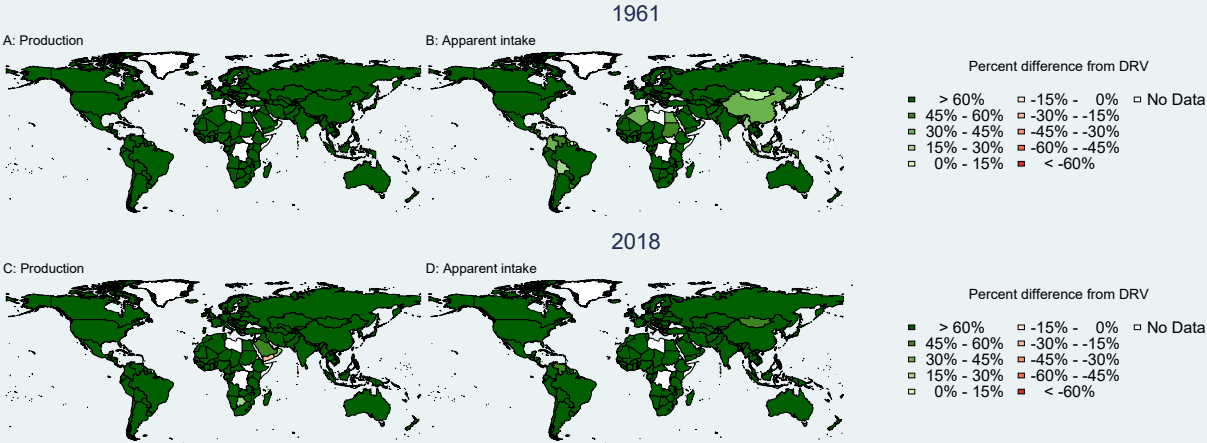

Niacin (B3)

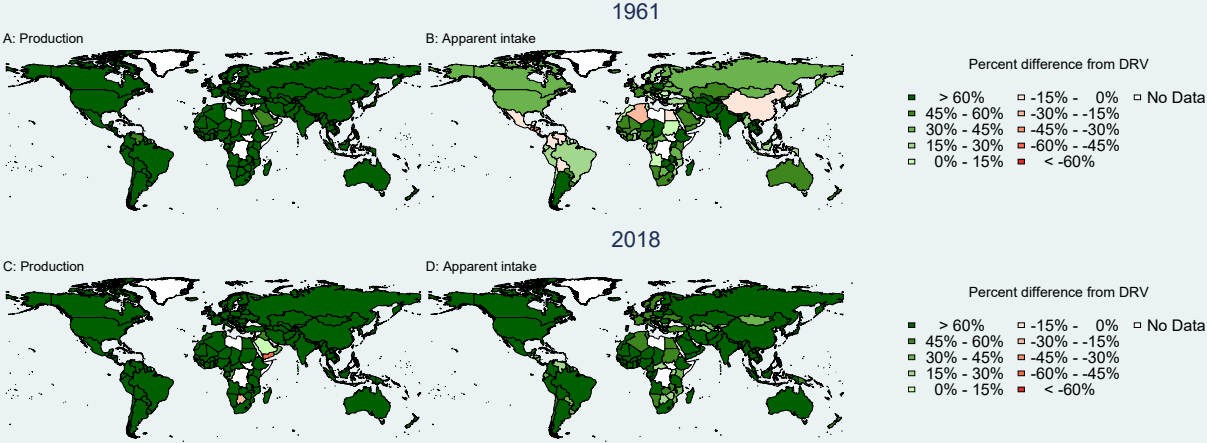

Vitamin B6

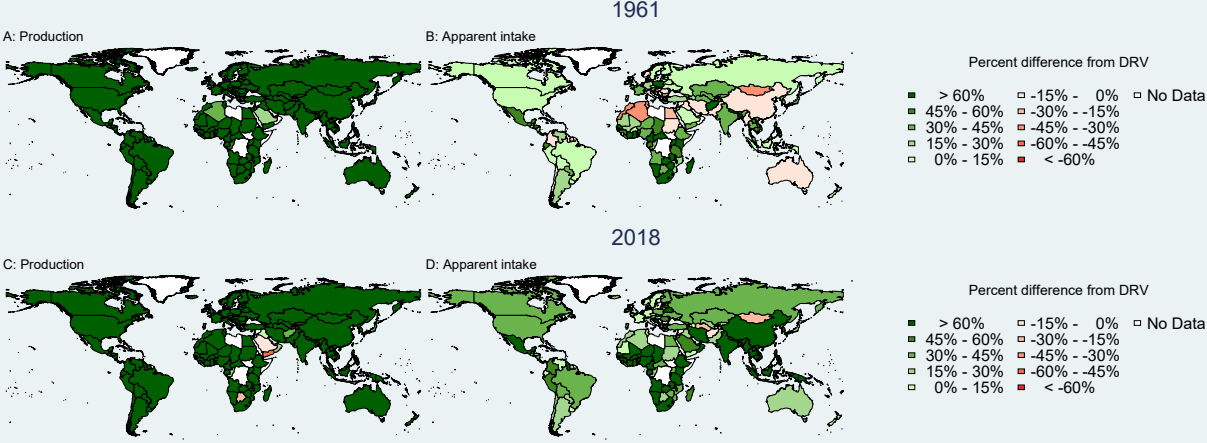

Supplement: Supplementary Figure 4 [file EMS153904-supplement-Supplementary_Figure_4.pdf]

Riboflavin (B2)

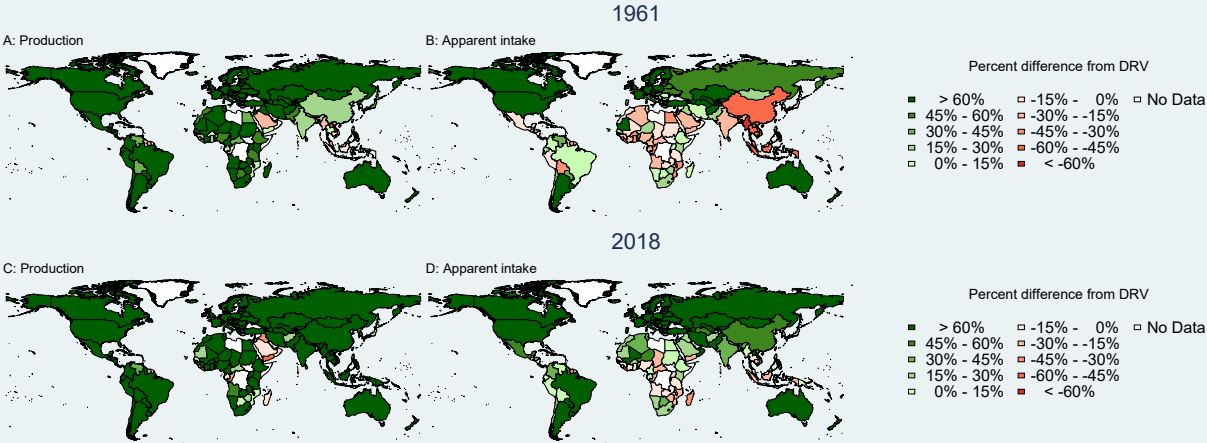

Pantothenic acid (B5)

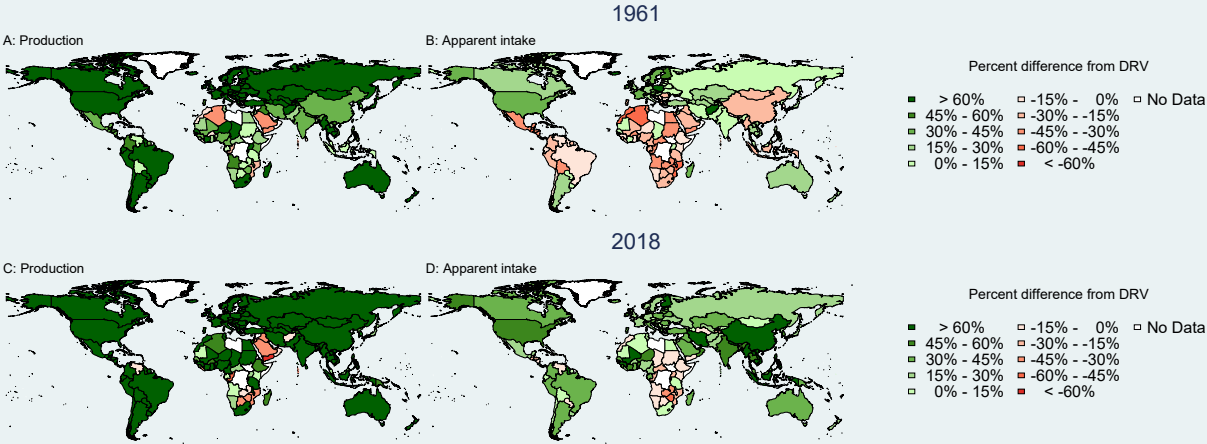

Vitamin B12

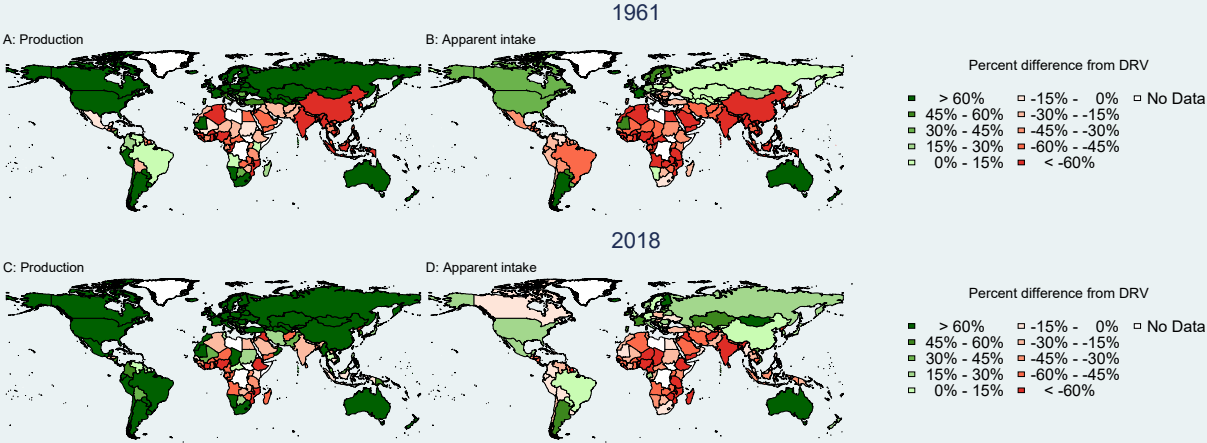

Supplement: Supplementary Figure 5 [file EMS153904-supplement-Supplementary_Figure_5.pdf]

Manganese (Mn)

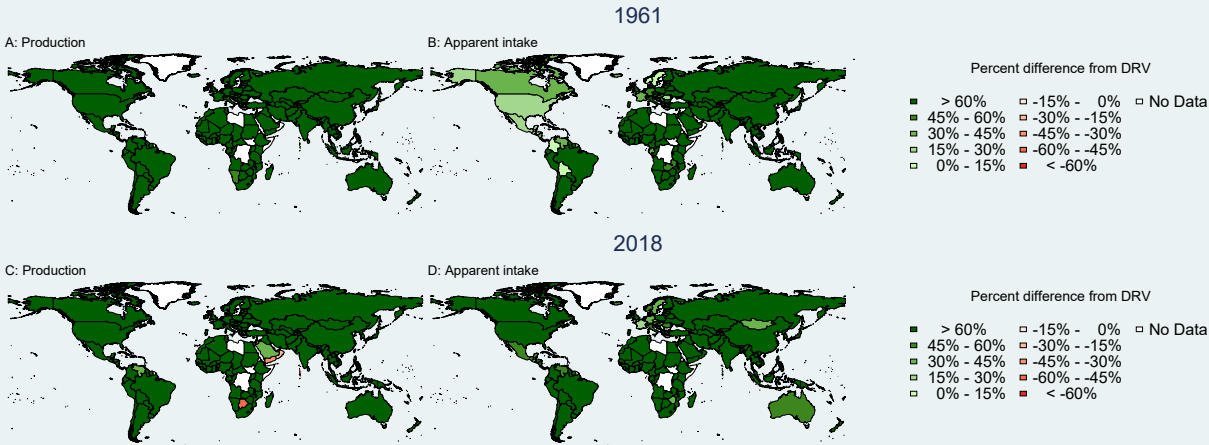

Folate (B9)

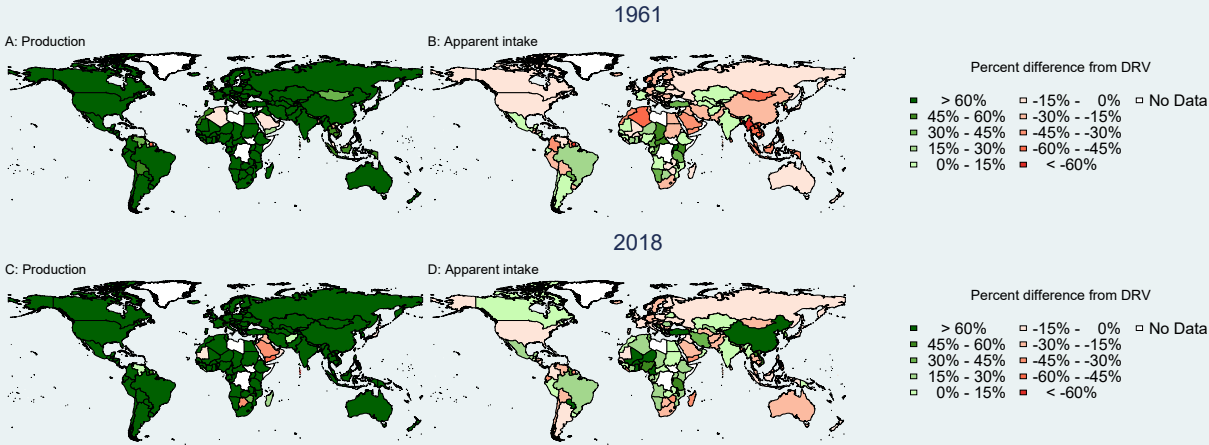

Vitamin C

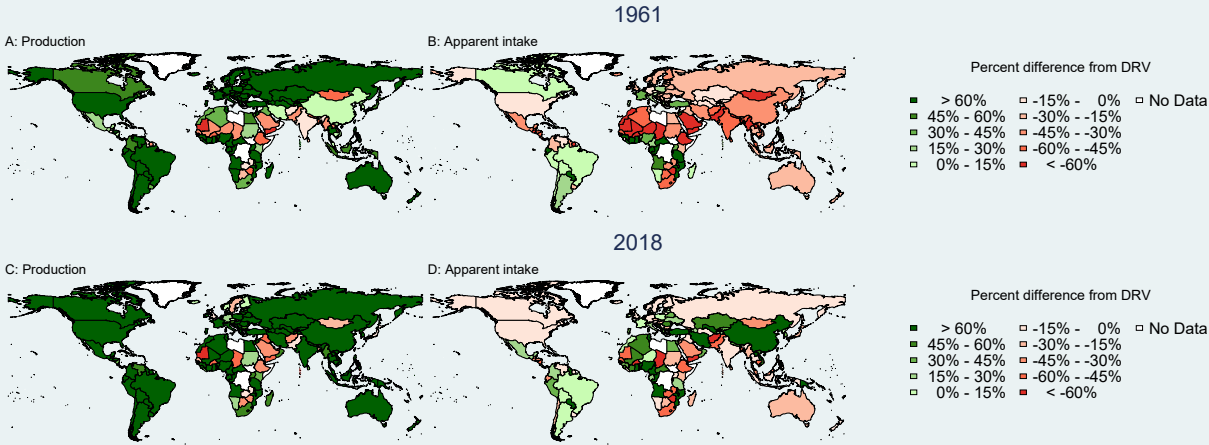

Supplement: Supplementary Figure 6 [file EMS153904-supplement-Supplementary_Figure_6.pdf]

Copper (Cu)

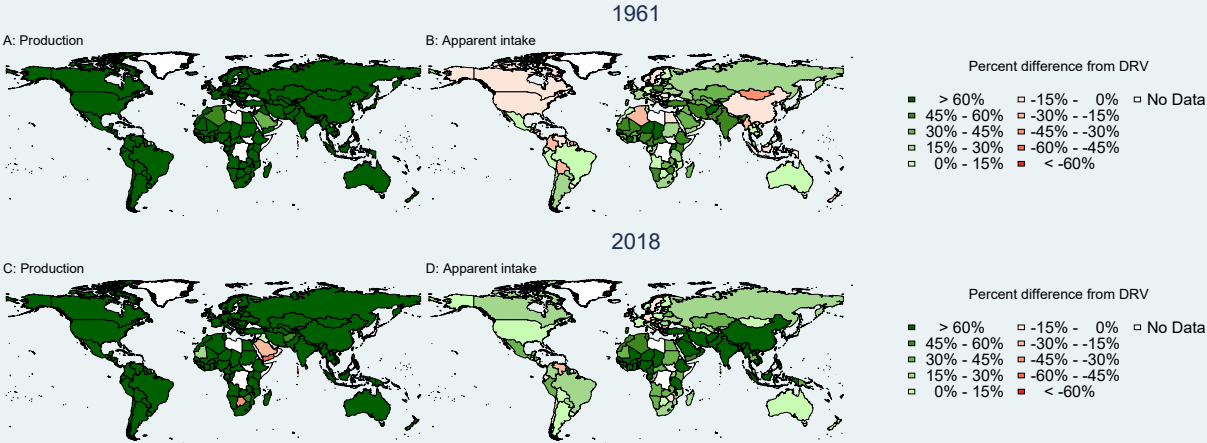

Magnesium (Mg)

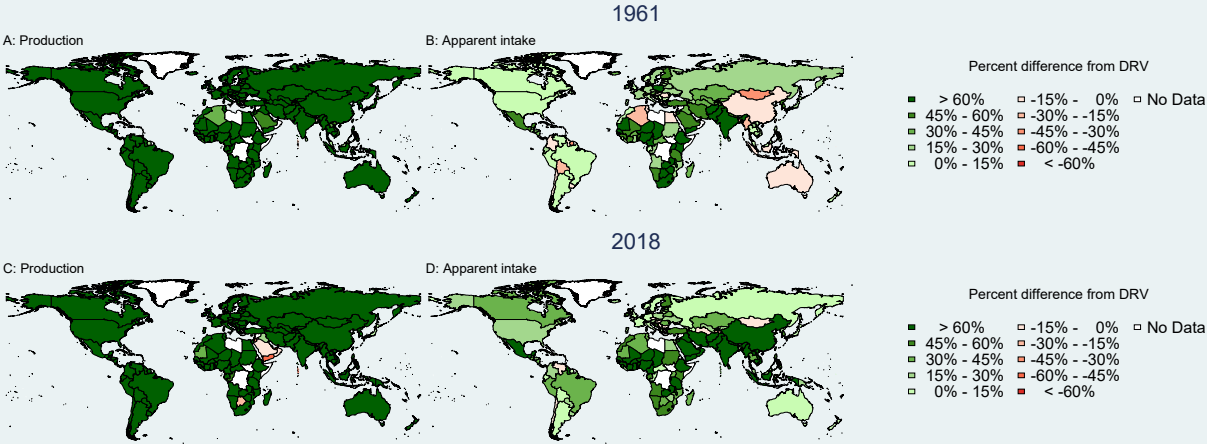

Selenium (Se)

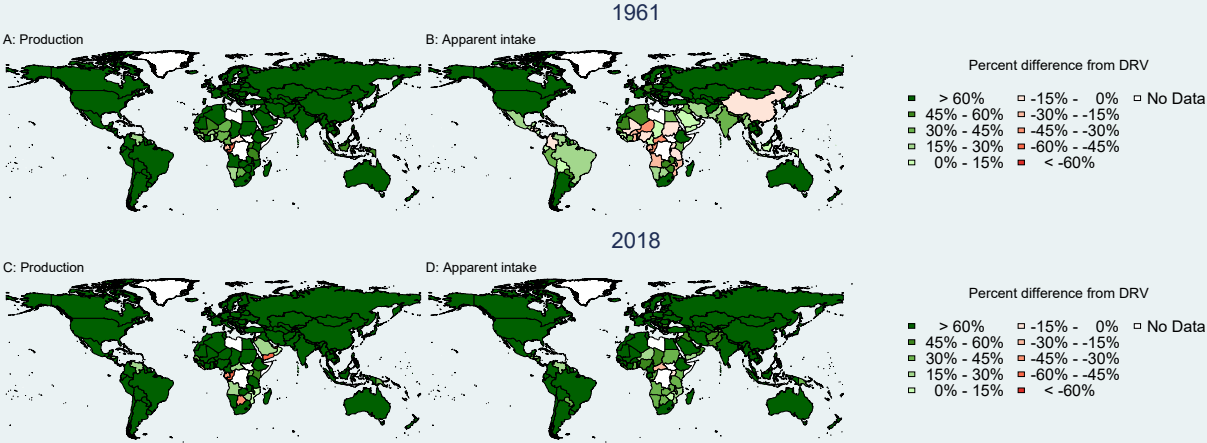

Supplement: Supplementary Figure 7 [file EMS153904-supplement-Supplementary_Figure_7.pdf]

Sodium (Na)

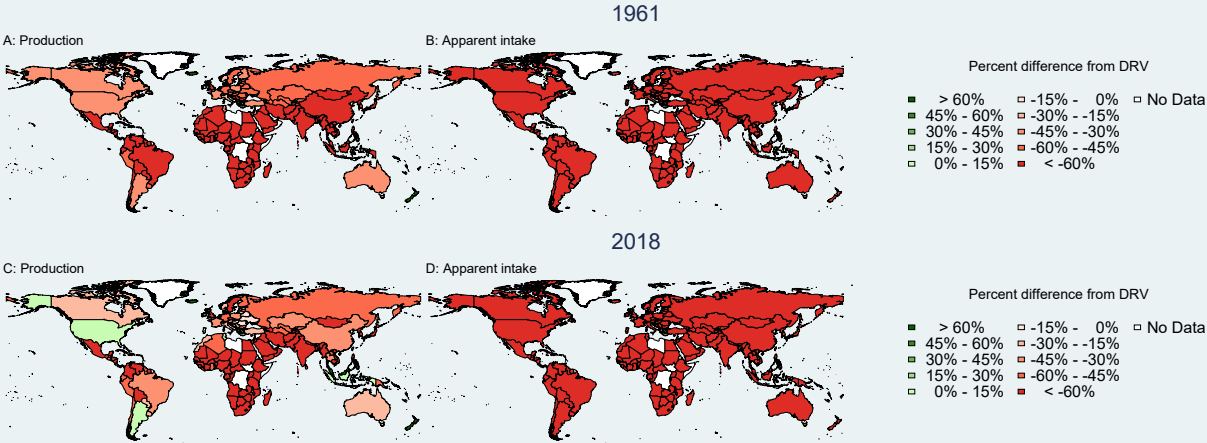

Potassium (K)

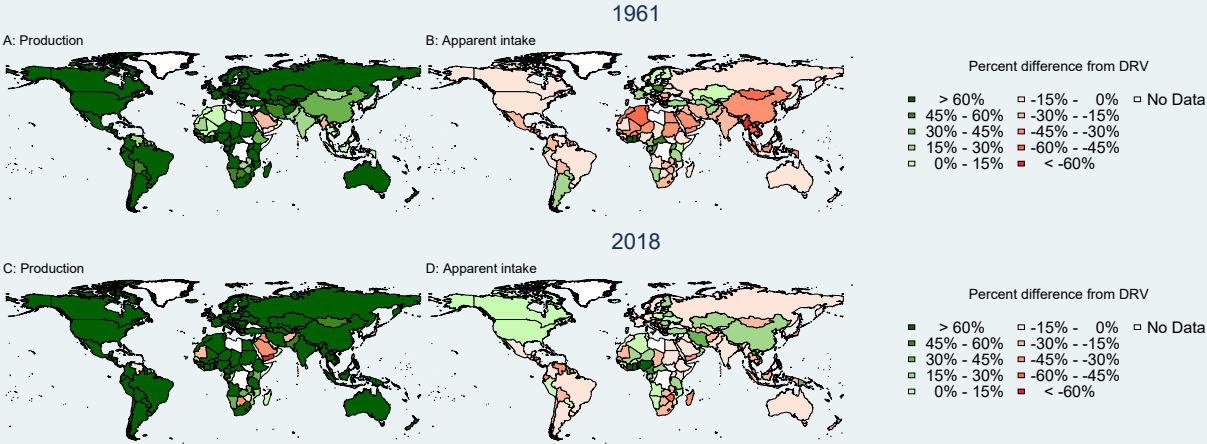

Phosphorous (P)

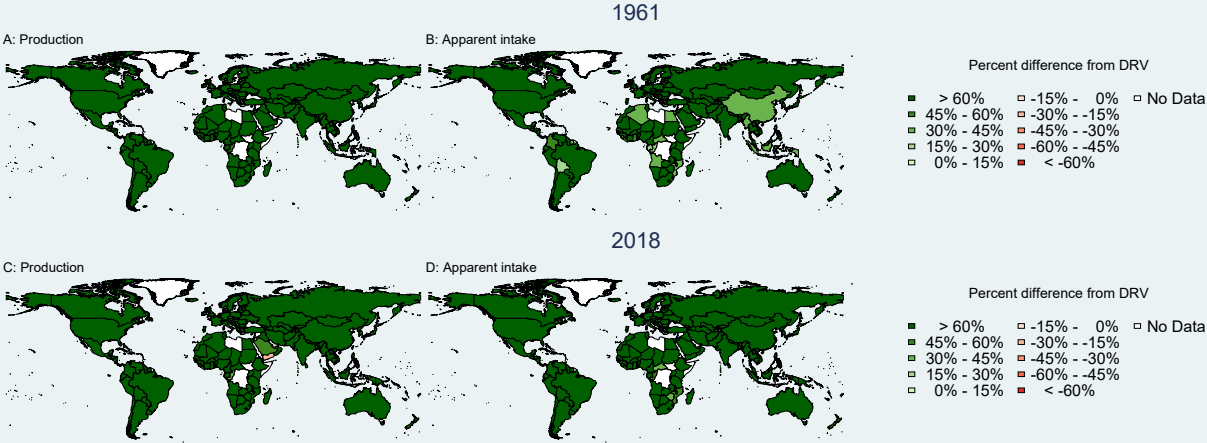

Supplement: Supplementary Figure 8 [file EMS153904-supplement-Supplementary_Figure_8.pdf]

Vitamin D

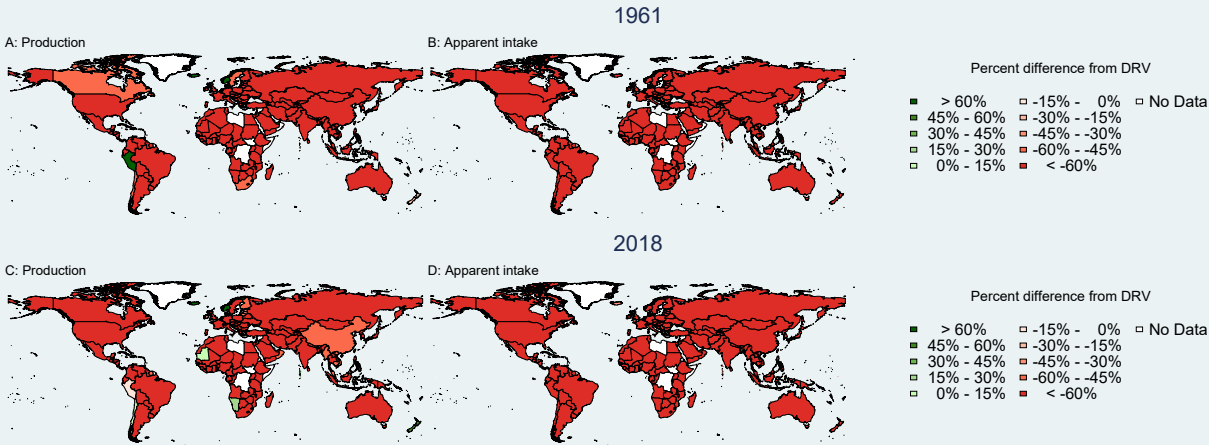

Vitamin E

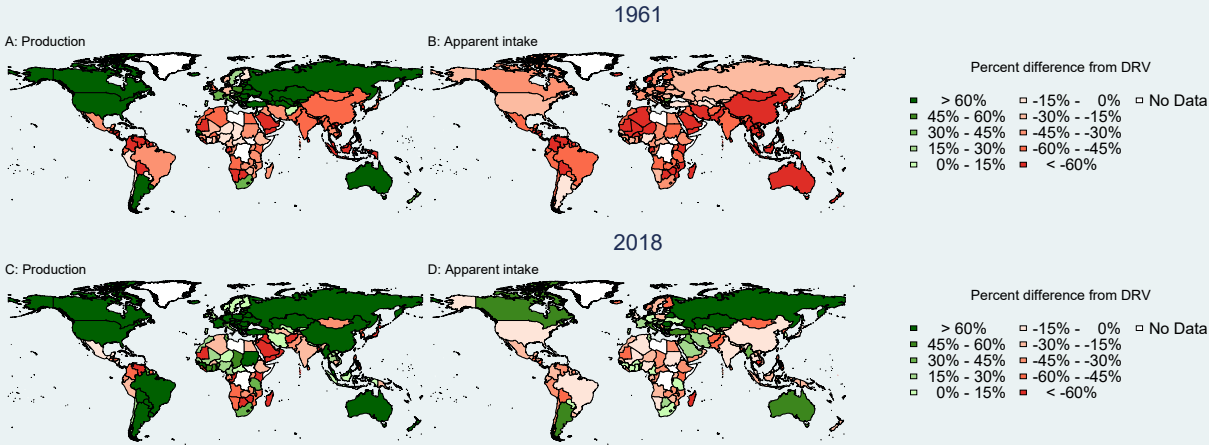

Vitamin K

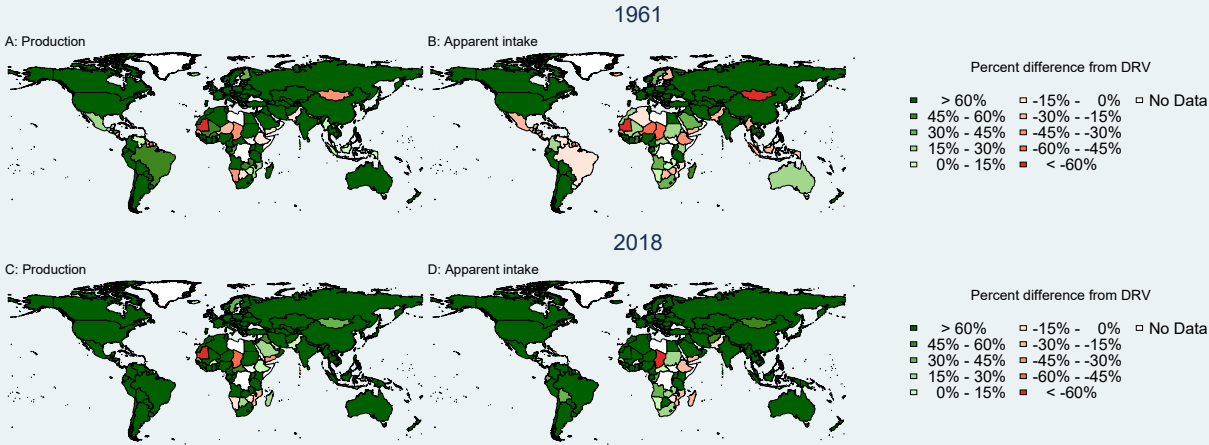

Supplement: Supplementary Figure 9 [file EMS153904-supplement-Supplementary_Figure_9.pdf]
