# Supplementary material for: Tracing global flows of bioactive compounds from farm to fork in Nutrient Balance Sheets can help guide intervention towards healthier food supplies
Source: Nat Food. Author manuscript; Available in PMC 2022 Oct 11. (PMC7613697; doi:10.1038/s43016-022-00585-w)
Supplement: Supplementary information [file EMS153904-supplement-Supplementary_information.pdf]

Supplemental figures, tables and formulas for:

Tracing global flows of bioactive compounds from farm to fork in Nutrient Balance Sheets can help guide intervention towards healthier food supplies

Keith Lividini, MS/MPH, and William A Masters, PhD

Supplementary Table 1: List of nutrients included in NBS
Supplementary Table 2: NBS food categories and relationships to food products
Supplementary Table 3: NBS food composition matches
Supplementary Table 4: NBS food matches for phytate
Supplementary Figure 1: Schematic development of the Nutrient Balance Sheets
Supplementary Figure 2: Iron (Fe), zinc (Zn) and calcium (Ca) production and intake relative to requirements
Supplementary Figure 3: Energy, protein and fiber production and intake relative to requirements
Supplementary Figure 4: Thiamin (B1), niacin (B3) and B6 production and intake relative to requirements
Supplementary Figure 5: Riboflavin (B2), pantothenic acid (B5) and B12 production and intake relative to requirements
Supplementary Figure 6: Manganese (Mn), folate (B9) and vitamin C production and intake relative to requirements
Supplementary Figure 7: Copper (Cu), magnesium (Mg) and selenium (Se) production and intake relative to requirements
Supplementary Figure 8: Sodium (Na), Potassium (K) and phosphorous (P) production and intake relative to requirements
Supplementary Figure 9: Vitamin D, vitamin E and vitamin K production and intake relative to requirements
Supplementary Formula 1: Expression of FBS elements as daily per capita quantities
Supplementary Formula 2: Per capita nutrient quantities of FBS elements
Supplementary Formula 3: Import ratio
Supplementary Formula 4: Production ratio
Supplementary Formula 5a: Vitamin A from palm oil production in SSA
Supplementary Formula 5b: Vitamin A from palm oil for non-production variables in SSA
Supplementary Formula 6a: Nutrients net of FLW and cooking loss
Supplementary Formula 6b: Nutrients net of FLW and cooking loss for seafood
Supplementary Formula 6c: Vitamin A from palm oil in SSA net of FLW and cooking
Supplementary Formula 7a: Nutrients from wheat and wheat flour
Supplementary Formula 7b: Nutrients from maize, millet, sorghum and flours
Supplementary Formula 7c: Nutrients from maize and masa harina
Supplementary Formula 8a: Nutrients from wheat and flour net of FLW and cooking loss
Supplementary Formula 8b: Nutrients from maize, millet, sorghum and flours net of FLW and cooking loss
Supplementary Formula 8c: Nutrients from maize and masa harina net of FLW and cooking loss
Supplementary Formula 9: Per capita heme iron
Supplementary Formula 10: Per capita nonheme iron
Supplementary Formula 11: Log percent nonheme iron availability using Conway algorithm
Supplementary Formula 12: Per capita bioavailable iron based on Conway algorithm
Supplementary Formula 13: Percent nonheme iron availability using Rickard (2009) algorithm
Supplementary Formula 14: Per capita bioavailable iron based on Rickard (2009) algorithm
Supplementary Formula 15: Log percent nonheme iron availability using Armah (2013) algorithm
Supplementary Formula 16: Per capita iron absorption based on Armah (2013) algorithm
Supplementary Formula 17: Total per capita absorbed zinc using Hambidge (2010) algorithm
Supplementary Formula 18: Number of pregnant women
Supplementary Formula 19: Number of lactating women
Supplementary Formula 20: Total daily nutrient requirements by demographic stratum
Supplementary Formula 21: Total daily national nutrient requirements
Supplementary Formula 22: Total daily national per capita nutrient requirements

Supplementary Table 1: List of nutrients included in NBS Macro and micronutrients were selected from the list of available nutrients in the Nutrient Database for Standard Reference Legacy Database (NDB) available at Food Data Central, focusing on essential nutrients for which there are Dietary Reference Values (DRVs) and/or Reference Intake Ranges (RIs). Simple sugars, sugar alcohols, alternative forms of vitamins A, D, E and K, provitamins A, amino acids, most polyphenols, and specific fatty acid chains are excluded. Two "antinutrients" are included to estimate iron and zinc bioavailability: 1) phytate data were obtained from PhyFoodComp1.0 available at INFOODS; and 2) polyphenol values for tea only were obtained from Table A1 from Hallberg and Hulthén (2000).

Num	Nutrient	Unit	Num	Nutrient	Unit
1	Energy	kcal	20	Vitamin D (D2+D3)	mcg
2	Protein	g	21	Vitamin C, total ascorbic acid	mg
3	Total lipid or fat	g	22	Thiamin	mg
4	Carbohydrate by difference	g	23	Riboflavin	mg
5	Total dietary fiber	g	24	Niacin	mg
6	Calcium, Ca	mg	25	Pantothenic acid	mg
7	Iron, Fe	mg	26	Vitamin B6	mg
8	Iron, heme, HFe	mg	27	Vitamin B12	mcg
9	Iron, non-heme, NHFe	mg	28	Choline, total	mg
10	Magnesium, Mg	mg	29	Vitamin K (phylloquinone)	mcg
11	Phosphorous, P	mg	30	Folate, food	mcg
12	Potassium, K	mg	31	Cholesterol	mg
13	Sodium, Na	mg	32	Fatty acids, total trans	g
14	Zinc, Zn	mg	33	Fatty acids, total saturated	g
15	Copper, Cu	mg	34	Fatty acids, total monounsaturated	g
16	Manganese, Mn	mg	35	Fatty acids, total polyunsaturated	g
17	Selenium, Se	mcg		Antinutrients	
18	Vitamin A, RAE	mcg	36	Phytate	mg
19	Vitamin E (alpha-tocopherol)	mg	37	Polyphenols (tannic acid equivalents)	mg

Supplementary Table 2: NBS food categories and relationships to food products The NBS is categorized into variables for fruits and vegetables (FV), heme-source iron (HIS), food group (FG), food subgroup (FSG) and food category (FC). FCs are constructed from 456 Supply and Utilization Accounts (SUAs), here called food products (FPs). For each variable, the NBS and/or FAO code is provided. For each FP, the CPC v2.1 code is provided and the authors' assumed relationship to a COICOP 2018 code. Within each FC, FPs used as primary commodities (used for FCT match) are shown in bold/italicized font. Within each FSG, food category other (FCO) is shown in italics. The FCO contains multiple primary commodities and an FP deemed 'Not Elsewhere Specified' (NES). NES are sets of additional FPs. Fish and seafood categories were informed using the FAO/Coordinating Working Party on Fishery Statistics (CWP) Handbook of Fishery Statistical Standards, Annex S.II: International Standard Statistical Classification of Aquatic Animals and Plants (ISSCAAP). Lists of fish species within these groups were obtained from the FAO software FishStatJ. (*) = NES foods; (**) = Only in 2014-2018 data. FPs that do not have any data in the 2014-2018 SUA data download are shown in gray highlight.

Fruit and Veg (FV)	Heme Source Iron (HIS)	Food Group (FG)	Food Subgroup (FSG)	Food Category (FC)	Food Product (FP)							
		NBS food group	FAO/FBS food group name	FAO/FBS food category	SUA item				SUA item			
					COICOP 2018 code	FAO/FBS item code	CPC 2.1 code	SUA item name	COICOP 2018	FAO/FBS item code	CPC 2.1 code	SUA item name
0: No	0: No	1: Starchy Staples	2905: Cereals - Excluding Beer	2511: Wheat and products	01.1.1.1.1	15	111	***Wheat***	01.1.1.3.9	22	F0022	Pastry
					01.1.1.2.1	16	23110	Flour, wheat	01.1.1.9.0	23	23220.01	Starch, wheat
					01.1.1.9.0	17	39120.01	Bran, wheat	01.1.1.9.0	24	23220.02	Gluten, wheat
					01.1.1.5.0	18	23710	Macaroni	01.1.1.4.0	41	23140.03	Cereals, breakfast
					01.1.1.9.0	19	23140.01	Germ, wheat	01.1.1.9.0	110	23490.01	Wafers
					01.1.1.3.1	20	F0020	Bread	01.1.1.9.0	114	23180	Mixes and doughs
					01.1.1.4.0	21	23140.02	Bulgur	01.1.1.9.0	115	23999.02	Food preparations, flour, malt extract
				2805: Rice (Milled Equivalent)	01.1.1.1.2	27	113	Rice, paddy	01.1.1.1.2	32	23161.03	Rice, broken
					01.1.1.1.2	28	23162	Rice, husked	01.1.1.9.0	33	39130.01	Gluten, rice
					01.1.1.1.2	29	23161.01	Rice, milled/husked	01.1.1.9.0	34	23220.03	Starch, rice
					01.1.1.1.2	31	23161.02	***Rice, milled***	01.1.1.9.0	35	39120.02	Bran, rice
									01.1.1.2.2	38	23120.01	Flour, rice
				2513: Barley and products	01.1.1.1.4	44	115	***Barley***	01.1.1.9.0	47	39120.03	Bran, barley
					01.1.1.9.0	45	23140.04	Barley, pot	01.1.1.2.4	48	23120.02	Flour, barley and grits
					01.1.1.9.0	46	23140.05	Barley, pearled	01.1.1.9.0	49	24320	Malt
									01.1.1.9.0	50	23999.01	Malt extract
				2514: Maize and products	01.1.1.1.6	56	112	***Maize***	01.1.1.9.0	59	39120.04	Bran, maize
					01.1.1.9.0	57	23140.06	Germ, maize	01.1.1.9.0	63	39130.02	Gluten, maize
					01.1.1.2.6	58	23120.03	Flour, maize	01.1.1.9.0	64	23220.04	Starch, maize
									01.1.1.9.0	846	39130.04	Feed and meal, gluten
				2515: Rye and products	01.1.1.1.9	71	116	***Rye***	01.1.1.2.9	72	23120.04	Flour, rye
									01.1.1.9.0	73	39120.05	Bran, rye
				2516: Oats	01.1.1.1.9	75	117	***Oats***	01.1.1.9.0	76	23140.07	Oats rolled
									01.1.1.9.0	77	39120.06	Bran, oats
				2517: Millet and products	01.1.1.1.5	79	118	***Millet***	01.1.1.2.5	80	23120.05	Flour, millet
									01.1.1.9.0	81	39120.07	Bran, millet
				2518: Sorghum and products	01.1.1.1.3	83	114	***Sorghum***	01.1.1.2.3	84	23120.06	Flour, sorghum
									01.1.1.9.0	85	39120.08	Bran, sorghum
				2520: Cereals, Other	01.1.1.9.0	68	N/A	Popcorn	01.1.1.2.9	98	23120.09	Flour, triticale
					01.1.1.1.9	89	1192	***Buckwheat***	01.1.1.9.0	99	39120.11	Bran, triticale
					01.1.1.2.9	90	23120.07	Flour, buckwheat	01.1.1.1.9	101	1195	**Canary seed**
					01.1.1.9.0	91	39120.09	Bran, buckwheat	01.1.1.1.9	103	1199.02	**Grain, mixed**
					01.1.1.1.7	92	1194	**Quinoa**	01.1.1.2.9	104	23120.1	Flour, mixed grain
					01.1.1.1.9	94	1193	**Fonio**	01.1.1.9.0	105	39120.12	Bran, mixed grains
					01.1.1.2.9	95	23120.08	Flour, fonio	01.1.1.1.9	108	1199.9	**Cereals, nes***
					01.1.1.9.0	96	39120.1	Bran, fonio	01.1.1.2.9	111	23120.9	Flour, cereals
					01.1.1.1.9	97	1191	**Triticale**	01.1.1.9.0	112	39120.13	Bran, cereals nes
									01.1.1.4.0	113	23140.08	Cereal preparations, nes
			2907: Starchy Roots	2532: Cassava and products	01.1.7.5.3	125	1520.01	**Cassava**	01.1.7.9.9	127	23230.02	Tapioca, cassava
					01.1.7.9.1	126	23170.01	Flour, cassava	01.1.7.7.0	128	1520.02	Cassava dried
									01.1.7.9.9	129	23220.06	Starch, cassava
				2531: Potatoes and products		116	1510	**Potatoes**	01.1.7.8.0	118	21313	Potatoes, frozen
					01.1.7.9.1	117	21392	Flour, potatoes	01.1.7.9.9	119	23220.05	Starch, potatoes
									01.1.7.9.9	121	23230.01	Tapioca, potatoes
				2533: Sweet potatoes	01.1.7.5.2	122	1530	**Sweet potatoes**				
				2533: Yams	01.1.7.5.4	137	1540	**Yams**				
				2534: Roots, other	01.1.7.5.6	135	1591	**Yautia (cocoyam)**	01.1.7.5.9	149	1599.1	**Roots and tubers, nes***
					01.1.7.5.5	136	1550	**Taro (cocoyam)**	01.1.7.9.1	150	23170.02	Flour, roots and tubers nes
									01.1.7.7.0	151	1599.2	Roots and tubers dried
		2: MN-Dense Vegetals	2911: Pulses	2546: Beans				Phaseolus spp.:	01.1.7.6.1	176	1701	**black gram, urd (Ph. mungo)**
					01.1.7.6.1	176	1701	**kidney, haricot bean (Ph. vulgaris)**	01.1.7.6.1	176	1701	**scarlet runner bean (Ph. coccineus)**
					01.1.7.6.1	176	1701	**lima, butter bean (Ph. lunatus)**	01.1.7.6.1	176	1701	**rice bean (Ph. calcaratus)**
					01.1.7.6.1	176	1701	**adzuki bean (Ph. angularis)**	01.1.7.6.1	176	1701	**moth bean (Ph. aconitifolius)**
					01.1.7.6.1	176	1701	**mungo bean, golden, green gram (Ph. aureus)**	01.1.7.6.1	176	1701	**tepary bean (Ph. acutifolius)**
				2547: Peas	01.1.7.6.5	187	1705	**Garden pea (Pisum sativum)**	01.1.7.6.5	187	1705	**field pea (P. arvense)**
				2549: Pulses, Other and products	01.1.7.6.2	181	1702	**Broad beans, horse beans, dry**	01.1.7.6.8	203	1708	**Bambara beans**
					01.1.7.6.3	191	1703	**Chick peas**	01.1.7.6.9	205	1709.01	**Vetches**
					01.1.7.6.6	195	1706	**Cow peas, dry**	01.1.7.6.9	210	1709.02	**Lupins**
					01.1.7.6.7	197	1707	**Pigeon peas**	01.1.7.6.9	211	1709.9	**Pulses, nes***
					01.1.7.6.4	201	1704	**Lentils**	01.1.7.9.1	212	23170.03	Flour, pulses
									01.1.7.9	213	39120.14	Bran, pulses
			2912: Treenuts	2551: Nuts and products	01.1.6.8.7	216	1377	**Brazil nuts, with shell**	01.1.6.8.9	226	1379.01	**Areca nuts**
					01.1.6.8.2	217	1372	**Cashew nuts, with shell**	01.1.6.8.7	229	21429.01	Brazil nuts, shelled
					01.1.6.8.3	220	1373	**Chestnut**	01.1.6.8.2	230	21424	Cashew nuts, shelled
					01.1.6.8.1	221	1371	**Almonds, with shell**	01.1.6.8.1	231	21422	Almonds shelled
					01.1.6.8.6	222	1376	**Walnuts, with shell**	01.1.6.8.6	232	21429.02	Walnuts, shelled
					01.1.6.8.5	223	1375	**Pistachios**	01.1.6.8.4	233	21423	Hazelnuts, shelled
					01.1.6.8.9	224	1379.02	**Kola nuts**	01.1.6.8.9	234	1379.9	**Nuts, nes***
					01.1.6.8.4	225	1374	**Hazelnuts, with shell**	01.1.6.9.4	235	F0235	Nuts, prepared (exc. groundnuts)
			2913: Oilcrops	2555: Soyabeans	01.1.7.3.5	236	141	**Soybeans**	01.1.9.3.9	240	23995.02	Soya paste
					01.1.9.3.9	239	23995.01	**Soya sauce**	01.1.7.9.5	241	23999.03	Soya curd
				2556: Groundnuts (Shelled Eq)	01.1.6.8.8	242	142	**Groundnuts, with shell**	01.1.6.9.4	246	21495.01	Groundnuts, prepared
					01.1.6.8.8	243	21421	**Groundnuts, shelled**	01.1.8.4.0	247	21495.02	Peanut butter
				2557: Sunflower seed	01.1.6.9.4	267	1445	**Sunflower seed**				
				2558: Rape and Mustardseed	01.1.6.9.4	270	1443	**Rapeseed**	01.1.6.9.4	292	1442	**Mustard seed**
									01.1.6.9.1	295	23995.03	Flour, mustard
				2559: Cottonseed	01.1.6.9.4	329	143	**Cottonseed**				
				2560: Coconuts - Incl Copra	01.1.6.1.8	249	1460	**Coconuts**	01.1.6.7.9	250	21429.07	Coconuts, desiccated
									01.1.6.7.9	251	1492	Copra

Supplementary Table 2 (continued)

Fruit and Veg (FV)	Heme Source Iron (HIS)	Food Group (FG)	Food Subgroup (FSG)	Food Category (FC)	Food Product (FP)							
		NBS food group	FAO/FBS food group name	FAO/FBS food category	SUA item				SUA item			
					COICOP 2018 code	FAO/FBS item code	CPC 2.1 code	SUA item name	COICOP 2018	FAO/FBS item code	CPC 2.1 code	SUA item name
0: No	0: No	2: MN-Dense Vegetals	2913: Oilcrops	2561: Sesame seed	01.1.9.4.0	289	1444	**Sesame seed**				
				2562: Palm kernels	01.1.5.1.9	254	1491.01	Oil, palm fruit	01.1.6.8.9	256	1491.02	**Palm kernels**
				2563: Olives (incl. pres.)	01.1.7.4.7	260	1450	**Olives**	01.1.7.9.3	262	F0262	Olives preserved
				2570: Oilcrops, Other	01.1.6.8.9	263	1499.01	**Karite nuts (sheanuts)**	01.1.6.8.9	305	1499.04	**Tallowtree seed**
					01.1.6.8.9	265	1447	**Castor oil seed**	01.1.6.8.9	310	1499.05	**Kapok fruit**
					01.1.6.8.9	275	1499.02	**Tung nuts**	01.1.6.8.9	311	1499.06	**Kapokseed in shell**
					01.1.6.8.9	277	1499.03	**Jojoba seed**	01.1.6.8.9	312	1499.07	Kapokseed shelled
					01.1.6.8.9	280	1446	**Safflower seed**	01.1.6.8.9	333	1441	**Linseed**
					01.1.9.4.0	296	1448	**Poppy seed**	01.1.6.8.9	336	1449.02	**Hempseed**
					01.1.6.8.9	299	1449.01	**Melonseed**	01.1.6.8.9	339	1449.9	**Oilseeds nes***
									01.1.6.9.1	343	21920	Flour, oilseeds
1: Yes			2918: Vegetables	2601: Tomatoes and products	01.1.7.2.4	388	1234	**Tomatoes**	01.2.1.0.0	390	21321	Juice, tomato
					01.2.1.0.0	389	N/A	Juice, tomato, concentrated	01.1.7.9.2	391	21399.01	Tomatoes, paste
									01.1.7.9.2	392	21399.02	Tomatoes, peeled
				2602: Onions	01.1.7.4.3	403	1253.02	**Onions, dry**				
1: Yes			2918: Vegetables	*2605: Vegetables, Other*	01.1.7.1.2	358	1212	**Cabbages and other brassicas**	01.1.7.8.0	447	21319.01	Sweet corn frozen
					01.1.7.1.6	366	1216	**Artichokes**	01.1.7.9.2	448	21399.03	Sweet corn prep or preserved
					01.1.7.1.1	367	1211	**Asparagus**	01.1.7.4.5	449	1270	**Mushrooms and truffles**
					01.1.7.1.4	372	1214	**Lettuce and chicory**	01.1.7.7.0	450	21393.01	Mushrooms, dried
					01.1.7.1.5	373	1215	**Spinach**	01.1.7.9.2	451	21397.01	Mushrooms, canned
					01.1.7.1.7	378	1219.01	**Cassava leaves**	01.1.7.1.4	459	1691	**Chicory roots**
					01.1.7.1.3	393	1213	**Cauliflowers and broccoli**	01.1.7.3.9	461	1356	**Carobs**
					01.1.7.2.5	394	1235	**Pumpkins, squash and gourds**	01.1.7.4.9	463	1290.9	**Vegetables, fresh nes***
					01.1.7.2.2	397	1232	**Cucumbers and gherkins**	01.1.7.7.0	464	N/A	Vegetables, dried nes*
					01.1.7.2.3	399	1233	**Eggplants (aubergines)**	01.1.7.9.2	465	N/A	Vegetables, canned nes*
					01.1.7.2.1	401	1231	**Chillies and peppers, green**	01.2.1.0.0	466	21329	Juice, vegetables nes*
					01.1.7.4.3	402	1253.01	**Onions, shallots, green**	01.1.7.7.0	469	21393.9	Vegetables, dehydrated
					01.1.7.4.2	406	1252	**Garlic**	01.1.7.9.2	471	21340	Vegetables in vinegar
					01.1.7.4.4	407	1254	**Leeks, other alliaceous vegetables**	01.1.7.9.2	472	F0472	Vegetables, preserved nes*
					01.1.7.3.1	414	1243	**Beans, green**	01.1.7.8.0	473	F0473	Vegetables, frozen
					01.1.7.3.3	417	1242	**Peas, green**	01.1.7.9.2	474	21330.9	Vegetables, temp. pres.
					01.1.7.3.9	420	1241.9	**Vegetables, leguminous nes***	01.1.7.8.0	475	F0475	Vegetables, preserved, frozen
					01.1.7.3.2	423	1241.01	**String beans**	01.1.7.9.9	476	23991.02	Vegetables, homog. preps
					01.1.7.4.1	426	1251	**Carrots and turnips**	01.1.6.5.4	567	1221	**Watermelons**
					01.1.7.2.6	430	1239.01	**Okra**	01.1.6.5.3	568	1229	**Melons, other (inc.cantlpes)**
					01.1.7.4.8	446	1290.01	**Maize, green**	01.2.2.0.2	658	23912.01	Coffee, subs. containing coffee
			2919: Fruits - Excluding Wine	2611: Oranges, Mandarines	01.1.6.2.3	490	1323	**Oranges**	01.2.1.0.0	492	21431.02	Juice, orange, concentrated
					01.2.1.0.0	491	21431.01	Juice, orange, single strength	01.1.6.2.4	495	1324	**Tangrns, mndrins, clmntines, satsumas**
									01.2.1.0.0	496	21439.01	Juice, tangerine
				2612: Lemons, Limes and products	01.1.6.2.2	497	1322	**Lemons and limes**	01.2.1.0.0	498	21439.02	Juice, lemon, single strength
									01.2.1.0.0	499	21439.03	Juice, lemon, concentrated
				2613: Grapefruit and products	01.1.6.2.1	507	1321	**Grapefruit (inc. pomelos)**	01.2.1.0.0	509	21432	Juice, grapefruit
									01.2.1.0.0	510	21432.01	Juice, grapefruit, concentrated
				2614: Citrus, Other	01.1.6.2.9	512	1329	**Fruit, citrus nes***	01.2.1.0.0	513	21439.04	Juice, citrus, single strength
									01.2.1.0.0	514	21439.05	Juice, citrus, concentrated
				2615: Bananas	01.1.6.1.2	486	1312	**Bananas**				
				2616: Plantains	01.1.7.5.7	489	1313	**Plantains**				
				2617: Apples and products	01.1.6.3.1	515	1341	**Apples**	01.2.1.0.0	518	21435.01	Juice, apple, single strength
									01.2.1.0.0	519	21435.02	Juice, apple, concentrated
				2618: Pineapples and products	01.1.6.1.7	574	1318	**Pineapples**	01.2.1.0.0	576	21433	Juice, pineapple
						575	21491	Pineapples canned	01.2.1.0.0	580	21433.01	Juice, pineapple, concentrated
				2619: Dates	01.1.6.1.3	577	1314	**Dates**				
				2620: Grapes and products (excl wine)	01.1.6.5.1	560	1330	**Grapes**	01.2.1.0.0	562	21434	Juice, grape
					01.1.6.7.1	561	21411	**Raisins**	01.2.1.0.0	563	24212.01	Grapes, must
				2625: Fruits, Other	01.1.6.3.2	521	1342.01	**Pears**	01.1.6.4.9	558	1355.9	**Berries nes***
					01.1.6.3.2	523	1342.02	**Quinces**	01.1.6.1.4	569	1315	**Figs**
					01.2.6.3.3	526	1343	**Apricots**	01.1.6.7.9	570	21419.02	Figs dried
					01.1.6.7.9	527	21419.01	Apricots, dry	01.1.6.1.5	571	1316	**Mangoes, mangosteens, guavas**
					01.1.6.3.4	530	1344.01	**Cherries, sour**	01.1.6.1.1	572	1311	**Avocados**
					01.1.6.3.4	531	1344.02	**Cherries**	01.2.1.0.0	583	21439.08	Juice, mango
					01.1.6.3.5	534	1345	**Peaches and nectarines**	01.1.6.5.5	587	1359.01	**Persimmons**
					01.1.6.3.6	536	1346	**Plums and sloes**	01.1.6.5.6	591	1359.02	**Cashewapple**
					01.1.6.7.9	537	21412	Plums dried (prunes)	01.1.6.5.2	592	1352	**Kiwi fruit**
					01.2.1.0.0	538	21439.06	Juice, plum, single strength	01.1.6.1.6	600	1317	**Papayas**
					01.2.1.0.0	539	21439.07	Juice, plum, concentrated	01.1.6.1.9	603	1319	**Fruit, tropical fresh nes***
					01.1.6.3.9	541	1349.2	**Fruit, stone nes***	01.1.6.7.9	604	21419.91	Fruit, tropical dried nes*
					01.1.6.3.9	542	1349.1	**Fruit, pome nes***	01.1.6.5.9	619	1359.9	**Fruit, fresh nes***
					01.1.6.4.5	544	1354	**Strawberries**	01.1.6.7.9	620	21419.99	Fruit, dried nes*
					01.1.6.4.3	547	1353.01	**Raspberries**	01.2.1.0.0	622	21439.9	Juice, fruit nes*
					01.1.6.4.2	549	1351.02	**Gooseberries**	01.1.6.9.9	623	F0623	Fruit, prepared nes*
					01.1.6.4.1	550	1351.01	**Currants**	01.1.6.9.1	624	23170.04	Flour, fruit
					01.1.6.4.6	552	1355.01	**Blueberries**	01.1.6.9.2	625	23670.02	Fruits, nuts, peel, sugar pres.
					01.1.6.4.7	554	1355.02	**Cranberries**	01.1.6.9.3	626	23991.03	Fruit, cooked, homog. preps.
0: No			2923: Spices	2640: Pepper	01.1.9.4.0	687	1651	**Pepper (piper spp.)**				
				2641: Pimento	01.1.9.4.0	689	1652	**Chillies and peppers, dry**				
				2642: Cloves	01.1.9.4.0	698	1656	**Cloves**				
				2645: Spices, Other	01.1.9.4.0	692	1658	**Vanilla**	01.1.9.4.0	711	1654	**Anise, badian, fennel, coriander**
					01.1.9.4.0	693	1655	**Cinnamon (canella)**	01.1.9.4.0	720	1657	**Ginger**
					01.1.9.4.0	702	1653	**Nutmeg, mace and cardamoms**	01.1.9.4.0	723	1699	**Spices, nes***
1: Yes			2961: Aquatic Products, Other	2775: Aquatic Plants	01.1.7.4.6	1594	N/A	Aquatic plants, fresh	01.1.7.7.0	1595	N/A	Aquatic plants, dried
									01.1.7.9.9	1596	N/A	Aquatic plants, other preps
0: No	1: Yes	3: Animal Source Foods	2943: Meat	2731: Bovine Meat	01.1.2.2.1	867	21111.01	**Meat, cattle**	01.1.2.5.1	874	21184.01	Meat, beef and veal sausages
					01.1.2.2.1	870	21111.02	Meat, cattle, boneless (beef & veal)	01.1.2.5.9	875	F0875	Meat, beef, preparations
					01.1.2.3.1	872	21182	Meat, beef, dried, salted, smoked	01.1.2.5.2	876	N/A	Meat, beef, canned
					01.1.2.5.9	873	21185	Meat, extracts	01.1.2.5.9	877	23991.04	Meat, homog. preps.
									01.1.2.2.1	947	21112	**Meat, buffalo**
				2732: Mutton & Goat Meat	01.1.2.2.3	977	21115	**Meat, sheep**	01.1.2.2.3	1017	21116	**Meat, goat**
				2733: Pigmeat	01.1.2.2.2	1035	21113.01	**Meat, pig**	01.1.2.3.2	1039	21181	Bacon and ham
					01.1.2.2.2	1038	21113.02	Meat, pork	01.1.2.5.1	1041	21184.02	Meat, pig sausages
									01.1.2.5.9	1042	F1042	Meat, pig, preparations
				2734: Poultry Meat	01.1.2.2.4	1058	21121	**Meat, chicken**	01.1.2.2.4	1069	21122	**Meat, duck**
					01.1.2.5.3	1060	21189.02	Fat, liver prepared (foie gras)	01.1.2.2.4	1073	21123	**Meat, goose and guinea fowl**
					01.1.2.5.2	1061	F1061	Meat, chicken, canned	01.1.2.2.4	1080	21124	**Meat, turkey**

Supplementary Table 2 (continued)

Fruit and Veg (FV)	Heme Source Iron (HIS)	Food Group (FG)	Food Subgroup (FSG)	Food Category (FC)	Food Product (FP)							
					SUA item				SUA item			
		NBS food group	FAO/FBS food group name	FAO/FBS food category	COICOP 2018 code	FAO/FBS item code	CPC 2.1 code	SUA item name	COICOP 2018	FAO/FBS item code	CPC 2.1 code	SUA item name
0: No	1: Yes	3: Animal Source Foods	2943: Meat	*2735: Meat, Other*	01.1.2.2.4	1089	21170.01	**Meat, bird nes***	01.1.2.2.9	1163	21170.02	**Meat, game**
					01.1.2.2.6	1097	21118.01	**Meat, horse**	01.1.2.2.9	1151	21119.01	**Meat, other rodents**
					01.1.2.2.6	1108	21118.02	**Meat, ass**	01.1.2.2.7	1158	21117.02	**Meat, other camelids**
					01.1.2.2.6	1111	21118.03	**Meat, mule**	01.1.2.3.9	1164	21183	**Meat, dried nes***
					01.1.2.2.7	1127	21117.01	**Meat, camel**	01.1.2.2.9	1166	21170.92	**Meat, nes***
					01.1.2.2.5	1141	21114	**Meat, rabbit**	01.1.2.5.9	1172	F1172	Meat, nes, preparations
									01.1.2.2.9	1176	2920	**Snails, not sea**
			2945: Offals	2736: Offals, Edible	01.1.2.4.0	868	21151	**Offals, edible, cattle**	01.1.2.4.0	1074	21160.02	**Offals, liver geese**
					01.1.2.5.3	878	21189.01	Liver prep.	01.1.2.4.0	1075	21160.03	**Offals, liver duck**
					01.1.2.4.0	948	21152	**Offals, edible, buffaloes**	01.1.2.4.0	1081	21160.04	**Offals, liver turkeys**
					01.1.2.4.0	978	21155	**Offals, sheep,edible**	01.1.2.4.0	1098	21159.01	**Offals, horses**
					01.1.2.4.0	1018	21156	**Offals, edible, goats**	01.1.2.4.0	1128	21159.02	**Offals, edible, camels**
					01.1.2.4.0	1036	21153	**Offals, pigs, edible**	01.1.2.4.0	1159	N/A	Offals, other camelids
					01.1.2.4.0	1059	21160.01	**Offals, liver chicken**	01.1.2.4.0	1167	21170.93	**Offals, nes***
	0: No		2946: Animal fats	2740: Butter, Ghee	01.1.5.2.1	886	22241.01	**Butter, cow milk**	01.1.5.2.9	953	22242.02	Ghee, of buffalo milk
					01.1.5.2.9	887	22241.02	Ghee, butteroil of cow milk	01.1.5.2.1	983	22249.01	**Butter and ghee, sheep milk**
					01.1.5.2.1	952	22242.01	**Butter, buffalo milk**	01.1.5.2.1	1022	22249.02	**Butter of goat mlk**
			2946: Animal fats	2743: Cream	01.1.4.3.3	885	22120	**Cream fresh**				
				2737: Fats, Animals, Raw	01.1.5.9.2	869	21512	**Fat, cattle**	01.1.5.9.2	1065	21511.03	**Fat, poultry**
					01.1.5.9.2	871	21512.01	**Fat, cattle butcher**	01.1.5.9.2	1066	21522	Fat, poultry, rendered
					01.1.5.9.2	949	21513	**Fat, buffaloes**	01.1.5.9.9	1129	21519.02	**Fat, camels**
					01.1.5.9.2	979	21514	**Fat, sheep**	01.1.5.9.9	1160	21519.03	**Fat, other camelids**
					01.1.5.9.2	994	F0994	Grease incl. lanolin wool	01.1.5.9.9	1168	21529.03	**Oils, fats of animal nes***
					01.1.5.9.2	1019	21515	**Fat, goats**	01.1.5.9.1	1221	21529.02	Lard stearine oil
					01.1.5.9.1	1037	21511.01	**Fat, pigs**	01.1.3.3.3	1222	21932.01	Degras
					01.1.5.9.1	1040	21511.02	**Fat, pig butcher**	01.1.5.9.2	1225	21523	**Tallow**
					01.1.5.9.1	1043	21521	**Lard**	01.1.5.9.9	1243	F1243	Fat, nes, prepared*
				2781: Fish, Body Oil	01.1.5.9.3	1509	N/A	**Frwt Bdy Oil**	01.1.5.9.4	1535	N/A	**Pelg Bdy Oil**
					01.1.5.9.4	1522	N/A	**Dmrs Bdy Oil**	01.1.5.9.4	1548	N/A	**Marn Bdy Oil**
									01.1.5.9.9	1582	N/A	**Aq M Oils**
				2782: Fish, Liver Oil	01.1.5.9.4	1510	N/A	**Frwt Lvr Oil**	01.1.5.9.4	1536	N/A	**Pelg Lvr Oil**
					01.1.5.9.4	1523	N/A	**Demersal Liver Oils**	01.1.5.9.4	1549	N/A	**Marine nes Liver Oils**
			2949: Eggs	2744: Eggs	01.1.4.8.9	916	23993.01	Egg albumine	01.1.4.8.9	1063	23993.02	Eggs, liquid
					01.1.4.8.1	1062	231	**Eggs, hen, in shell**	01.1.4.8.9	1064	23993.03	Eggs, dried
									01.1.4.8.2	1091	232	**Eggs, other bird, in shell**
			2948: Milk - Excluding Butter	2848: Milk - Excluding Butter	01.1.4.1.1	882	2211	**Milk, whole fresh cow**	01.1.4.5.0	904	22251.02	Cheese, skimmed cow milk
					01.1.4.2.0	888	22110.02	Milk, skimmed cow	01.1.4.9.0	905	22251.03	Whey, cheese
					01.1.4.3.1	889	22222.01	Milk, whole condensed	01.1.4.5.0	907	22251.04	Cheese, processed
					01.1.4.9.0	890	22130.03	Whey, condensed	01.1.4.3.9	908	22110.03	Milk, reconstituted
					01.1.4.6.0	891	22230.01	Yoghurt	01.1.4.7.0	909	22290	Milk, products of natural constituents nes
					01.1.4.6.0	892	22230.02	Yoghurt, concentrated or not	01.1.4.7.0	910	22270	Ice cream and edible ice
					01.1.4.6.0	893	22230.03	Buttermilk, curdled, acidified milk	01.1.4.9.0	917	22260	Casein
					01.1.4.3.1	894	22221.01	Milk, whole evaporated	01.1.4.1.2	951	2212	**Milk, whole fresh buffalo**
					01.1.4.3.1	895	22221.02	Milk, skimmed evaporated	01.1.4.2.0	954	22110.04	Milk, skimmed buffalo
					01.1.4.3.1	896	22222.02	Milk, skimmed condensed	01.1.4.5.0	955	22252	Cheese, buffalo milk
					01.1.4.3.2	897	22211	Milk, whole dried	01.1.4.1.3	982	2291	**Milk, whole fresh sheep**
					01.1.4.3.2	898	22212	Milk, skimmed dried	01.1.4.5.0	984	22253	Cheese, sheep milk
					01.1.4.6.0	899	22230.04	Milk, dry buttermilk	01.1.4.2.0	985	22110.05	Milk, skimmed sheep
					01.1.4.9.0	900	22130.02	Whey, dry	01.1.4.1.3	1020	2292	**Milk, whole fresh goat**
					01.1.4.5.0	901	22251.01	Cheese, whole cow milk	01.1.4.5.0	1021	22254	Cheese of goat mlk
					01.1.4.9.0	903	22130.01	Whey, fresh	01.1.4.2.0	1023	22110.06	Milk, skimmed goat
									01.1.4.1.4	1130	2293	**Milk, whole fresh camel**
	1: Yes		2960: Fish, Seafood	2761: Freshwater Fish	01.1.3.1.1	1501	N/A	**Frwtr Diad F**	01.1.3.2.9	1505	N/A	Frwtr Cured
					01.1.3.1.1	1502	N/A	Frwtr Fz Whl	01.1.3.3	1506	N/A	Frwtr Canned
					01.1.3.1.1	1503	N/A	Frwtr Fillet	01.1.3.3	1507	N/A	Frwtr Pr nes
					01.1.3.1.1	1504	N/A	Frwtr Fz Flt	01.1.3.3	1508	N/A	Frwtr Meals
				2762: Demersal Fish	01.1.3.1.3/4	1514	N/A	**Dmrsl Fresh**	01.1.3.2.2	1518	N/A	Dmrsl Cured
					01.1.3.1.3/4	1515	N/A	Dmrsl Fz Whl	01.1.3.3	1519	N/A	Dmrsl Canned
					01.1.3.1.3/4	1516	N/A	Dmrsl Fillet	01.1.3.3	1520	N/A	Dmrsl Pr nes
					01.1.3.1.3/4	1517	N/A	Dmrsl Fz Flt	01.1.3.3	1521	N/A	Dmrsl Meals
				2763: Pelagic Fish	01.1.3.1.2/5/6	1527	N/A	**Pelagic Frsh**	01.1.3.2.1	1531	N/A	Pelgc Cured
					01.1.3.1.2/5/6	1528	N/A	Pelgc Fz Whl	01.1.3.3.1/2	1532	N/A	Pelgc Canned
					01.1.3.1.2/5/6	1529	N/A	Pelgc Fillet	01.1.3.3.1/2	1533	N/A	Pelgc Pr nes
					01.1.3.1.2/5/6	1530	N/A	Pelgc Fz Flt	01.1.3.3.1/2	1534	N/A	Pelgc Meals
				2764: Marine Fish, Other	01.1.3.1.9	1540	N/A	**Marine nes F**	01.1.3.2.9	1544	N/A	Marin Cured
					01.1.3.1.9	1541	N/A	Marin Fz Whl	01.1.3.3	1545	N/A	Marin Canned
					01.1.3.1.9	1542	N/A	Marin Fillet	01.1.3.3	1546	N/A	Marin Pr nes
					01.1.3.1.9	1543	N/A	Marin Fz Flt	01.1.3.3	1547	N/A	Marin Meals
				2765: Crustaceans	01.1.3.4.1/2	1553	N/A	**Crstaceans F**	01.1.3.6.1/2	1556	N/A	Crstc Canned
					01.1.3.4.1/2	1554	N/A	Crstc Frozen	01.1.3.6.1/2	1557	N/A	Crstc Pr nes
					01.1.3.4.1/2	1555	N/A	Crstc Cured	01.1.3.6.1/2	1558	N/A	Crstc Meals
				2766: Cephalopods	01.1.3.4.3/4	1570	N/A	**Cephlp Fresh**	01.1.3.6.3/4	1573	N/A	Cphlp Canned
					01.1.3.4.3/4	1571	N/A	Cphlp Frozen	01.1.3.6.3/4	1574	N/A	Cphlp Pr nes
					01.1.3.4.3/4	1572	N/A	Cphlp Cured	01.1.3.6.3/4	1575	N/A	Cphlp Meals
				2767: Molluscs, Other	01.1.3.4.5	1562	N/A	**Mlluscs Frsh**	01.1.3.6.5	1564	N/A	Molsc Cured
					01.1.3.4.5	1563	N/A	Molsc Frozen	01.1.3.6.5	1565	N/A	Molsc Canned
									01.1.3.6.5	1566	N/A	Molsc Meals
			2961: Aquatic Products, Other	2768: Meat, Aquatic Mammals	01.1.3.4.9	1580	N/A	Aq M Meat	01.1.3.6.9	1583	N/A	Aq M Prep Ns
				2769: Aquatic Animals, Others	01.1.2.2.9	1587	N/A	Aqutc Anim F	01.1.2.5.1	1589	N/A	Aquatic Animals Meals
					01.1.2.3.9	1588	N/A	Aq A Cured	01.1.2.5.1	1590	N/A	Aq A Prep Ns
	0: No	4: Other	2908: Sugar Crops	2236: Sugar cane	01.1.8.1.1	156	1802	**Sugar cane**				
				2537: Sugar beet	01.1.8.1.2	157	1801	**Sugar beet**				
			2909: Sugar & Sweeteners	2541: Sugar non-centrifugal	01.1.8.1.1	163	23511.02	Sugar non-centrifugal				
				2542: Sugar (Raw Equivalent)	01.1.8.1.1	158	23511.01	Sugar, cane, raw, centrifugal	01.1.8.1.1	164	23520	Sugar refined
					01.1.8.1.2	159	23512	Sugar, beet, raw, centrifugal	01.1.8.1.1	168	23670.01	Sugar confectionery
					01.1.8.1	162	2351F	**Sugar Raw Centrifugal**	01.1.8.2.0	171	23530	Sugar flavoured
				2745: Honey	01.1.8.3.1	1182	2910	**Honey, natural**				
				2543: Sweeteners, Other	01.1.8.2.0	154	23210.01	Fructose chemically pure	01.1.8.2.0	166	23210.03	**Fructose and syrup, other**
					01.1.8.2.0	155	23210.02	Maltose chemically pure	01.1.8.2.0	167	23210.04	**Sugar, nes***
					01.1.8.2.0	160	23530	**Maple sugar and syrups**	01.1.8.2.0	172	23210.05	Glucose and dextrose
					01.1.8.2.0	161	1809	**Sugar crops, nes***	01.1.8.2.0	173	23210.06	Lactose
					01.1.8.2.0	165	23540	**Molasses**	01.1.8.2.0	175	23210.08	Isoglucose
									01.2.4.0.0	633	24490	Beverages, non alcoholic

Supplementary Table 2 (continued)

Fruit and Veg (FV)	Heme Source Iron (HIS)	Food Group (FG)	Food Subgroup (FSG)	Food Category (FC)	Food Product (FP)							
					SUA item				SUA item			
		NBS food group	FAO/FBS food group name	FAO/FBS food category	COICOP 2018 code	FAO/FBS item code	CPC 2.1 code	SUA item name	COICOP 2018	FAO/FBS item code	CPC 2.1 code	SUA item name
0: No	0: No	4: Other	2914: Vegetable Oils	2571: Soyabean Oil	01.1.5.1.4	237	2161	**Oil, soybean**				
				2572: Groundnut Oil	01.1.5.1.5	244	2162	**Oil, groundnut**				
				2573: Sunflowerseed Oil	01.1.5.1.1	268	21631.01	**Oil, sunflower**				
				2574: Rape and Mustard Oil	01.1.5.1.9	271	21641.01	**Oil, rapeseed**	01.1.5.1.9	293	21641.02	**Oil, mustard**
				2575: Cottonseed Oil	01.1.5.1.9	331	2168	**Oil, cottonseed**				
				2576: Palmkernel Oil	01.1.5.1.9	258	21691.14	**Oil, palm kernel**				
				2577: Palm Oil	01.1.5.1.2	257	2165	**Oil, palm**	01.1.5.1.9	1276	34120	Fatty acids
									01.1.5.1.9	1277	21932.02	Fatty substance residues
				2578: Coconut Oil	01.1.5.1.6	252	2166	**Oil, coconut (copra)**				
				2579: Sesameseed Oil	01.1.5.1.9	290	21691.07	**Oil, sesame**				
				2580: Olive Oil	01.1.5.1.3	261	2167	**Oil, olive, virgin**	01.1.5.1.9	274	21673	Oil, olive residues
				2581: Ricebran Oil	01.1.5.1.9	36	21691.01	**Oil, rice bran**				
				2582: Maize Germ Oil	01.1.5.1.7	60	21691.02	**Oil, maize**				
				2586: Oilcrops Oil, Other	01.1.5.1.9	264	21691.03	**Butter of karite nuts**	01.1.5.1.9	334	21691.12	**Oil, linseed**
					01.1.5.1.9	266	21691.04	**Oil, castor beans**	01.1.5.1.9	337	21691.13	**Oil, hempseed**
					01.1.5.1.9	276	21691.05	**Oil, tung nuts**	01.1.5.1.9	340	21691.9	**Oil, vegetable origin nes***
					01.1.5.1.9	278	21691.06	**Oil, jojoba**	01.1.8.5.9	664	23620	**Cocoa, butter**
					01.1.5.1.9	281	21631.02	**Oil, safflower**	01.1.5.3.0	1241	21700.01	**Margarine, liquid**
					01.1.5.1.9	297	21691.08	**Oil, poppy**	01.1.5.3.0	1242	21700.02	**Margarine, short**
					01.1.5.1.9	306	21691.09	**Vegetable tallow**	01.1.5.1.9	1273	21693.02	**Castor oil, hydrog. (opal wax)**
					01.1.5.1.9	307	21691.1	**Oil, stillingia**	01.1.5.1.9	1274	34550	Oil, boiled etc
					01.1.5.1.9	313	21691.11	**Oil, kapok**	01.1.5.1.9	1275	F1275	**Oil, hydrogenated**
			2922: Stimulants	2630: Coffee and products	01.2.2.0.1	656	1610	**Coffee, green**	01.2.2.0.1	657	23911	Coffee, roasted
									01.2.2.0.2	659	23912.02	Coffee, extracts
				2633: Cocoa Beans and products	01.1.8.5.2	661	1640	**Cocoa, beans**	01.1.8.5.9	665	F0665	Cocoa, powder & cake
					01.1.8.5.9	662	23610.01	Cocoa, paste	01.1.8.5.9	666	F0666	Chocolate products nes*
				2635: Tea (including mate)	01.2.3.0	667	1620	**Tea**	01.2.3.0.5	671	1630	Matcha
									01.2.3.0.9	672	23914	Tea, mate extracts
			2924: Alcoholic Beverages	2655: Wine	02.1.2.1	564	24212.02	**Wine**	02.1.2.1	565	24220	Vermouths & similar
				2656: Beer	02.2.3.0	51	24310.01	**Beer of barley**				
				2657: Beverages, Fermented	02.1.9.0	26	24230.01	**Beverages, fermented wheat**	02.2.3.0	82	24310.03	**Beer of millet**
					02.1.9.0	39	24230.02	**Beverages, fermented rice**	02.2.3.0	86	24310.04	**Beer of sorghum**
					02.2.3.0	66	24310.02	**Beer of maize**	02.1.9.0	517	24230.03	**Cider etc**
				2658: Beverages, Alcoholic	02.1.1.0	634	2413	**Beverages, distilled alcoholic**				
				2659: Alcohol, Non-Food		632	24110	Alcohol non food				
			2928: Miscellaneous	2680: Infant food	01.1.9.2.1	109	23991.01	**Preparations for infant consumption, usually containing some non-cereal ingredients.**				
				2899: Miscellaneous	01.1.9.3.9	1232	F1232	Food prep nes**				

Supplementary Figure 1: Schematic development of the Nutrient Balance Sheets Production, trade, stock changes and all outflows (**A**) and available food (**B**) are converted into daily per capita quantities (**C**). Food availability is replicated over four variables to create: (1) total availability; (2) availability minus food loss and waste (FLW) and cooking loss; (3) total availability minus cereal milling; and (4) total availability minus cereal milling, FLW and cooking loss (referred to as apparent intake). Milling losses for wheat, maize, sorghum, and millet are applied for (3) and (4) (**D** & **E**). Food composition is then calculated for 33 nutrients (**F**). Non-edible portions and losses due to retail and household waste and cooking are subtracted (**G, H** and **I**) to create balance quantities standardized for each nutrient (**J**). Iron bioavailability (3 separate algorithms) and absorbed zinc are then calculated (**K**). Resulting values are compared to national per capita requirements (**L**).

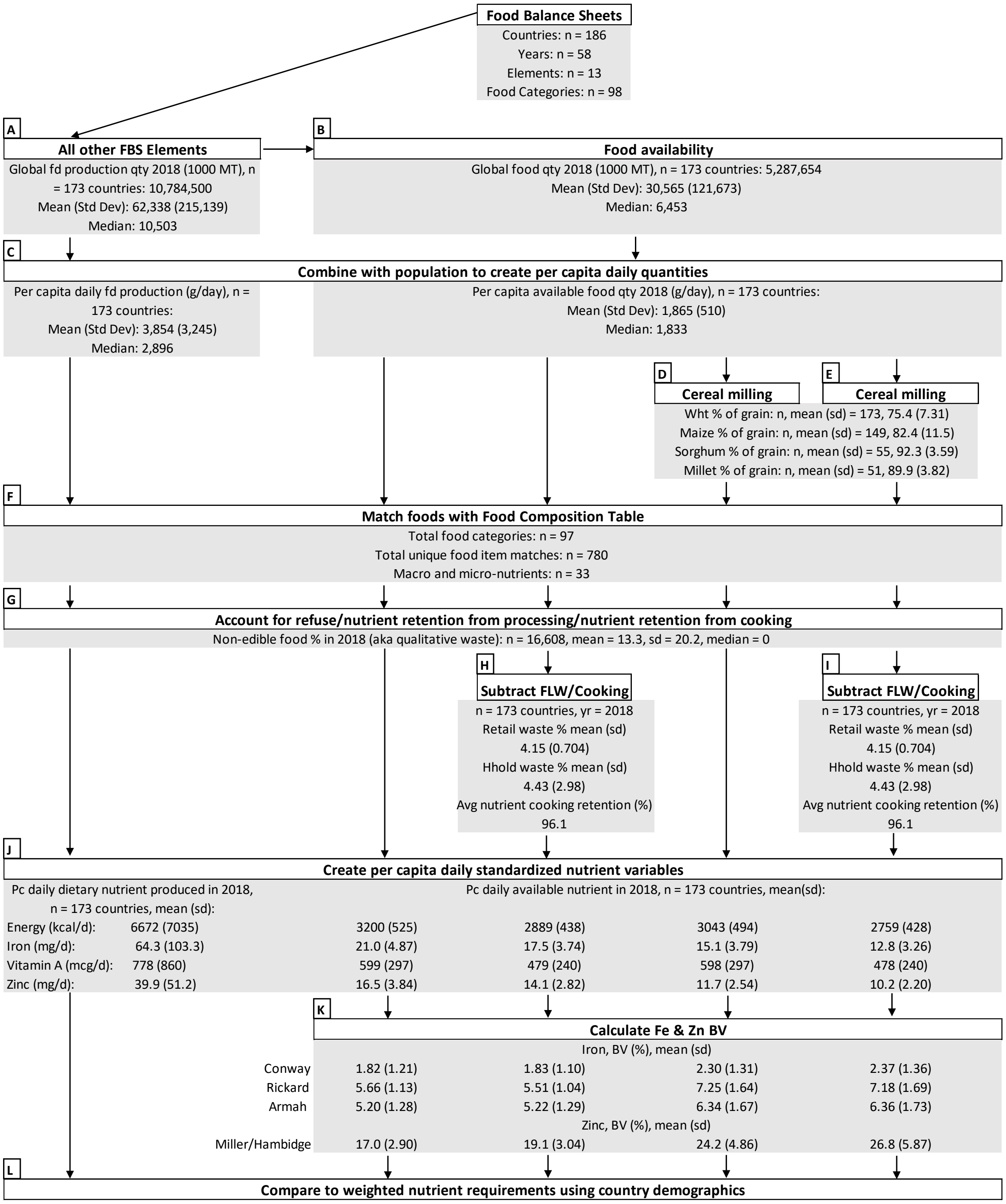

Supplementary Figure 2: Iron (Fe), zinc (Zn) and calcium (Ca) production and intake relative to requirements Annual per capita quantities of iron, zinc and calcium from food production and apparent intake were calculated for each country, then compared to total national average requirements (AR) based on EFSA Dietary Reference Values (DRVs) for the country's population in each 1-year age and sex group using annual country demographic composition data available from UN DESA World Population Prospects, 2019 revision, with additional calculations for each country's fraction of women who were pregnant or lactating. Annual values were then expressed as percentages of weighted per capita requirements in each year: ((pcNBS value - pc AR)/pc AR) * 100%. Fe and Zn are unadjusted for bioavailability and absorption, respectively.

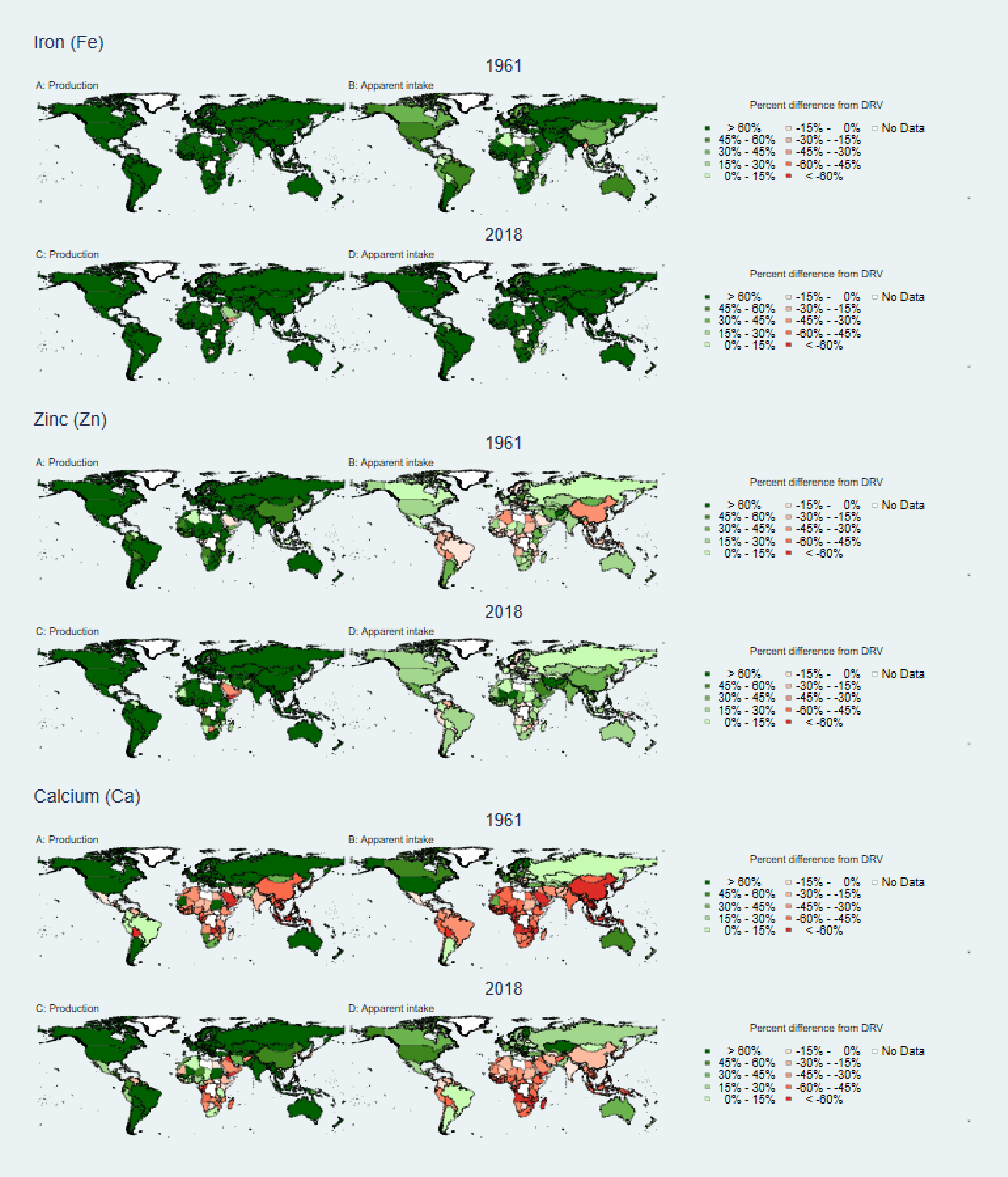

Supplementary Figure 3: Energy, protein and fiber production and intake relative to requirements Annual per capita quantities of dietary energy, protein and fiber from food production and apparent intake were calculated for each country, then compared to total national average requirements (AR) for energy and protein and adequate intakes (AI) for fiber based on EFSA Dietary Reference Values (DRVs) for the country's population in each 1-year age and sex group using annual country demographic composition data available from UN DESA World Population Prospects, 2019 revision, with additional calculations for each country's fraction of women who were pregnant or lactating. Annual values were then expressed as percentages of weighted per capita requirements in each year: ((pcNBS value - pc DRV)/pc DRV) * 100%.

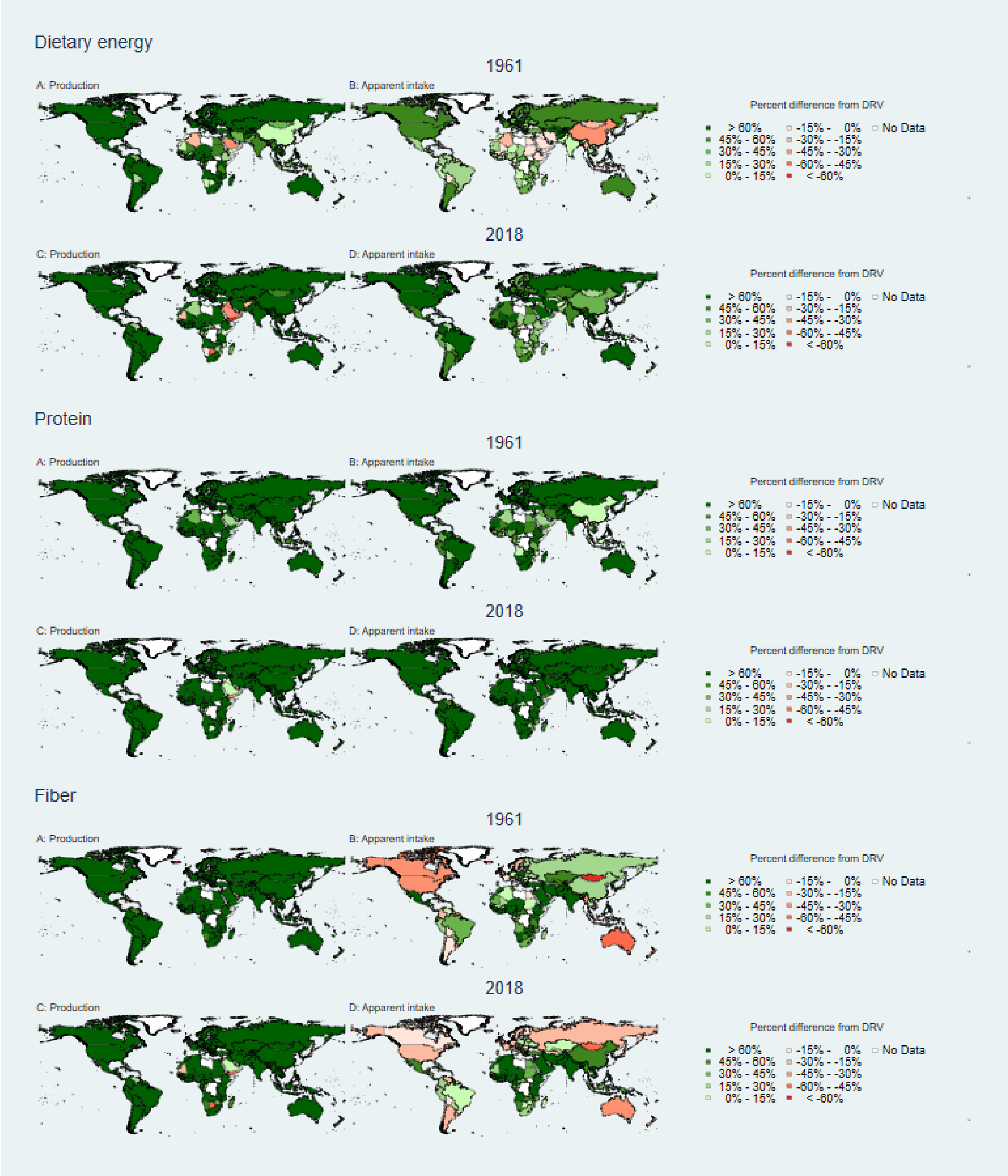

Supplementary Figure 4: Thiamin (B1), niacin (B3) and B6 production and intake relative to requirements Annual per capita quantities of thiamin, niacin, and vitaminB6 from food production and apparent intake were calculated for each country, then compared to total national average requirements (AR) based on EFSA Dietary Reference Values (DRVs) for the country's population in each 1-year age and sex group using annual country demographic composition data available from UN DESA World Population Prospects, 2019 revision, with additional calculations for each country's fraction of women who were pregnant or lactating. Annual values were then expressed as percentages of weighted per capita requirements in each year: ((pcNBS value - pc AR)/pc AR) * 100%.

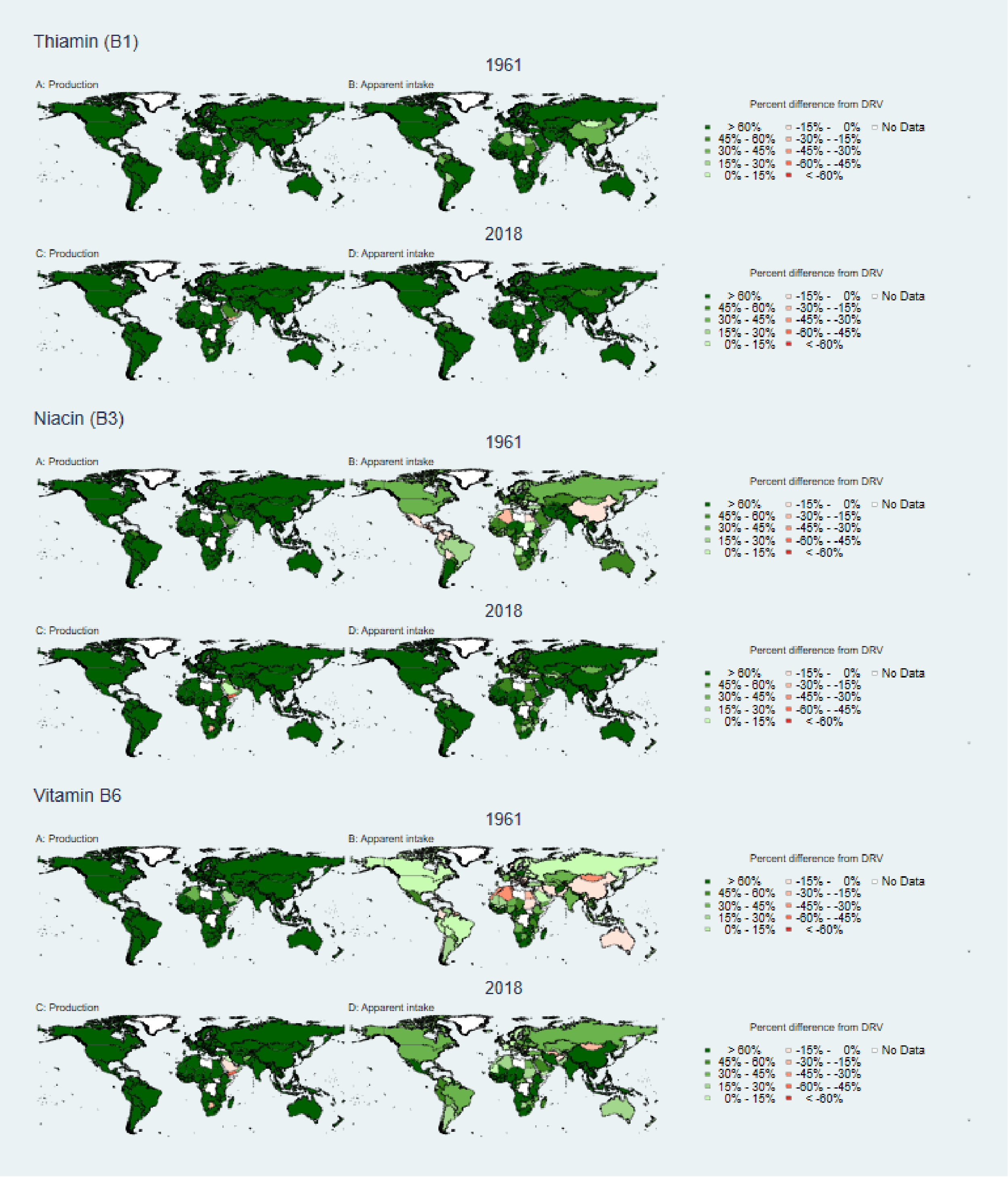

Supplementary Figure 5: Riboflavin (B2), pantothenic acid (B5) and B12 production and intake relative to requirements Annual per capita quantities of riboflavin, pantothenic acid, and vitamin B12 from food production and apparent intake were calculated for each country, then compared to total national average requirements (AR) for riboflavin and adequate intakes (AI) for pantothenic acid and B12 based on EFSA Dietary Reference Values (DRVs) for the country's population in each 1-year age and sex group using annual country demographic composition data available from UN DESA World Population Prospects, 2019 revision, with additional calculations for each country's fraction of women who were pregnant or lactating. Annual values were then expressed as percentages of weighted per capita requirements in each year: ((pcNBS value - pc DRV)/pc DRV) * 100%.

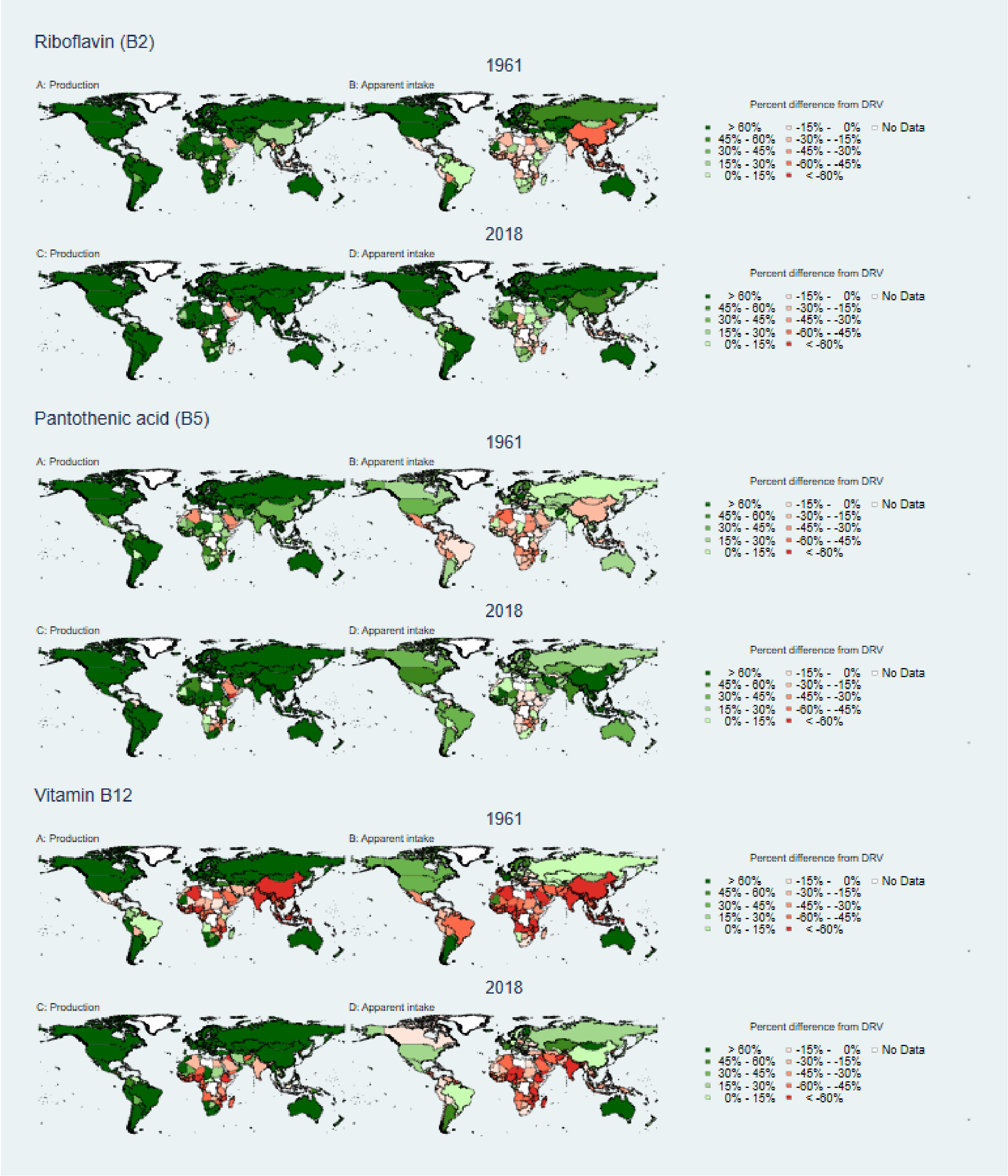

Supplementary Figure 6: Manganese (Mn), folate (B9) and vitamin C production and intake relative to requirements Annual per capita quantities of manganese, folate and vitamin C from food production and apparent intake were calculated for each country, then compared to total national average requirements (AR) for folate and vitamin C and adequate intakes (AI) for manganese based on EFSA Dietary Reference Values (DRVs) for the country's population in each 1-year age and sex group using annual country demographic composition data available from UN DESA World Population Prospects, 2019 revision, with additional calculations for each country's fraction of women who were pregnant or lactating. Annual values were then expressed as percentages of weighted per capita requirements in each year: ((pcNBS value - pc DRV)/pc DRV) * 100%.

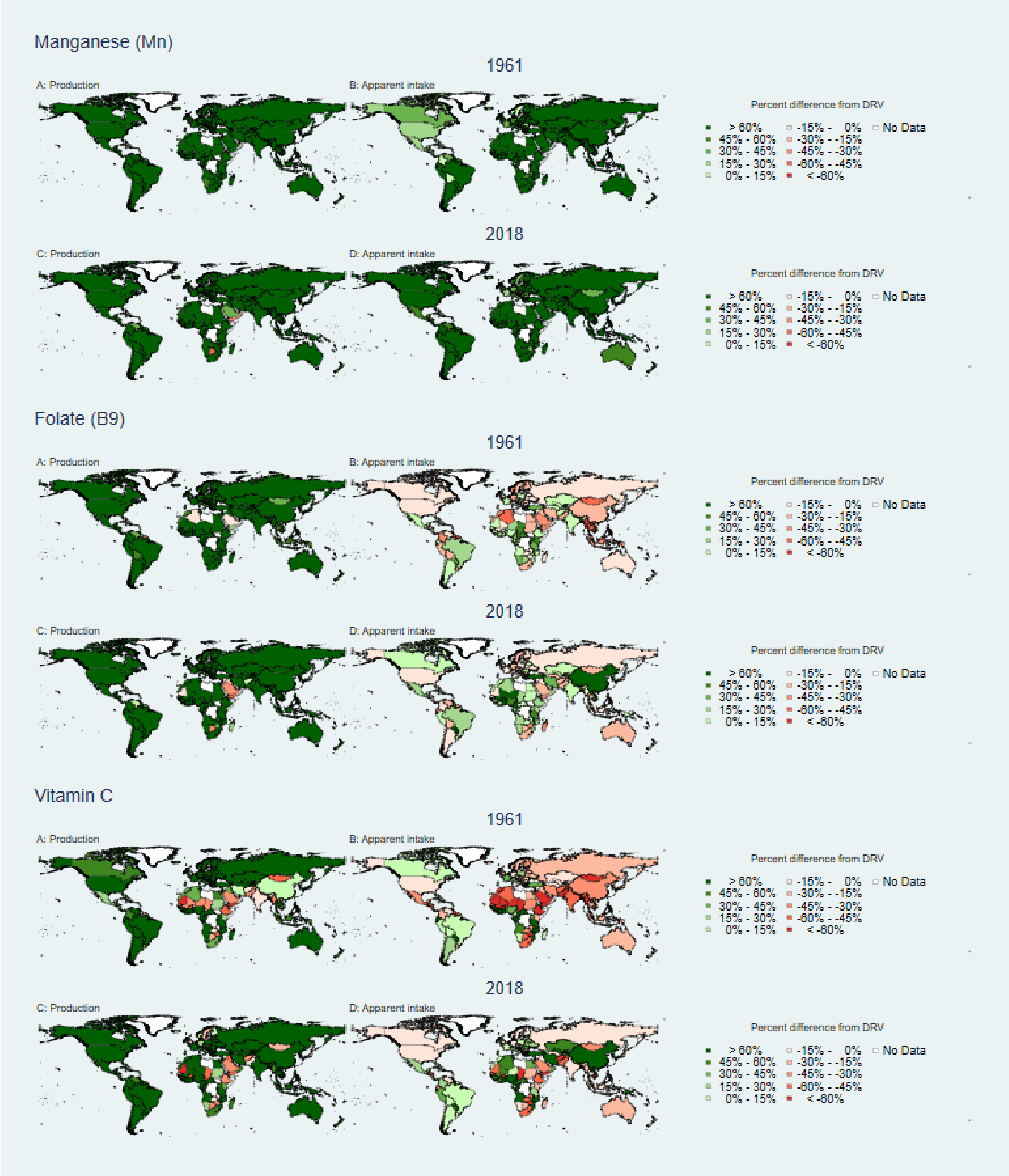

Supplementary Figure 7: Copper (Cu), magnesium (Mg) and selenium (Se) production and intake relative to requirements Annual per capita quantities of copper, magnesium and selenium from food production and apparent intake were calculated for each country, then compared to total national adequate intakes (AI) based on EFSA Dietary Reference Values (DRVs) for the country's population in each 1-year age and sex group using annual country demographic composition data available from UN DESA World Population Prospects, 2019 revision, with additional calculations for each country's fraction of women who were pregnant or lactating. Annual values were then expressed as percentages of weighted per capita requirements in each year: ((pcNBS value - pc AI)/pc AI) * 100%.

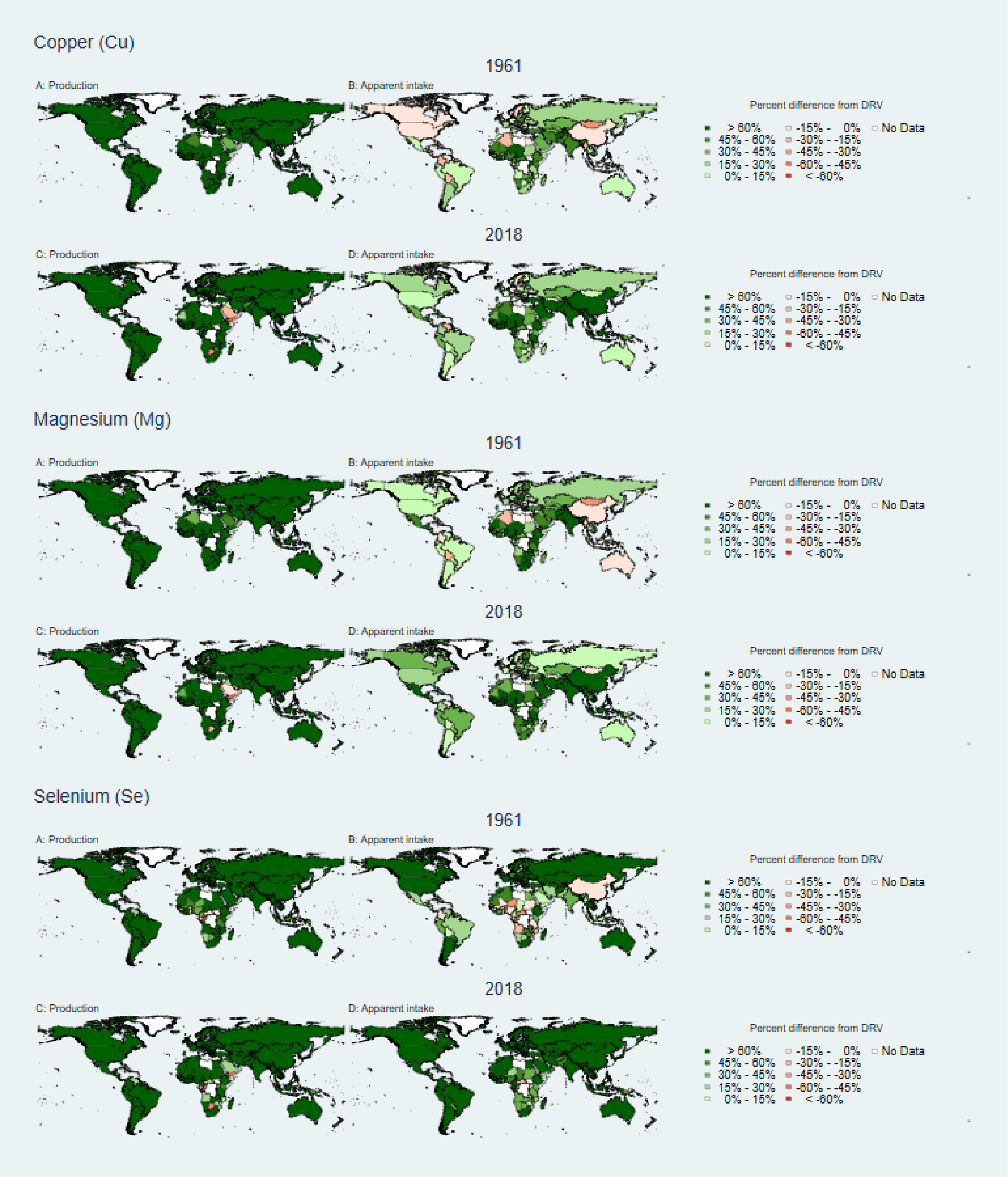

Supplementary Figure 8: Sodium (Na), Potassium (K) and phosphorous (P) production and intake relative to requirements Annual per capita quantities of sodium, potassium and phosphorous from food production and apparent intake were calculated for each country, then compared to total national adequate intakes (AI) based on EFSA Dietary Reference Values (DRVs) for the country's population in each 1-year age and sex group using annual country demographic composition data available from UN DESA World Population Prospects, 2019 revision, with additional calculations for each country's fraction of women who were pregnant or lactating. Annual values were then expressed as percentages of weighted per capita requirements in each year: ((pcNBS value - pc AI)/pc AI) * 100%.

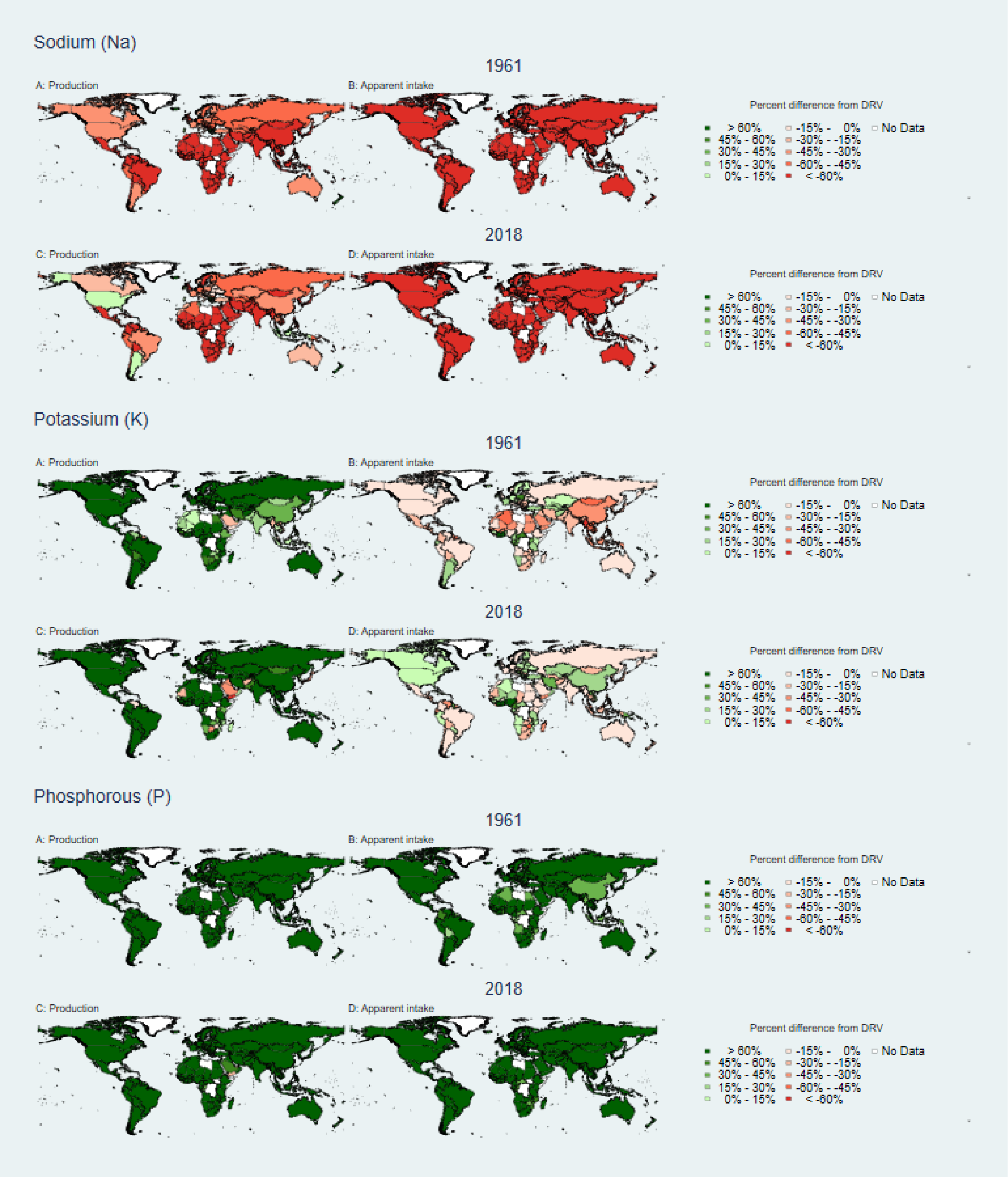

Supplementary Figure 9: Vitamin D, vitamin E and vitamin K production and intake relative to requirements Annual per capita quantities of vitamins D, E, and K from food production and apparent intake were calculated for each country, then compared to total national adequate intakes (AI) based on EFSA Dietary Reference Values (DRVs) for the country's population in each 1-year age and sex group using annual country demographic composition data available from UN DESA World Population Prospects, 2019 revision, with additional calculations for each country's fraction of women who were pregnant or lactating. Annual values were then expressed as percentages of weighted per capita requirements in each year: ((pcNBS value - pc AI)/pc AI) * 100%.

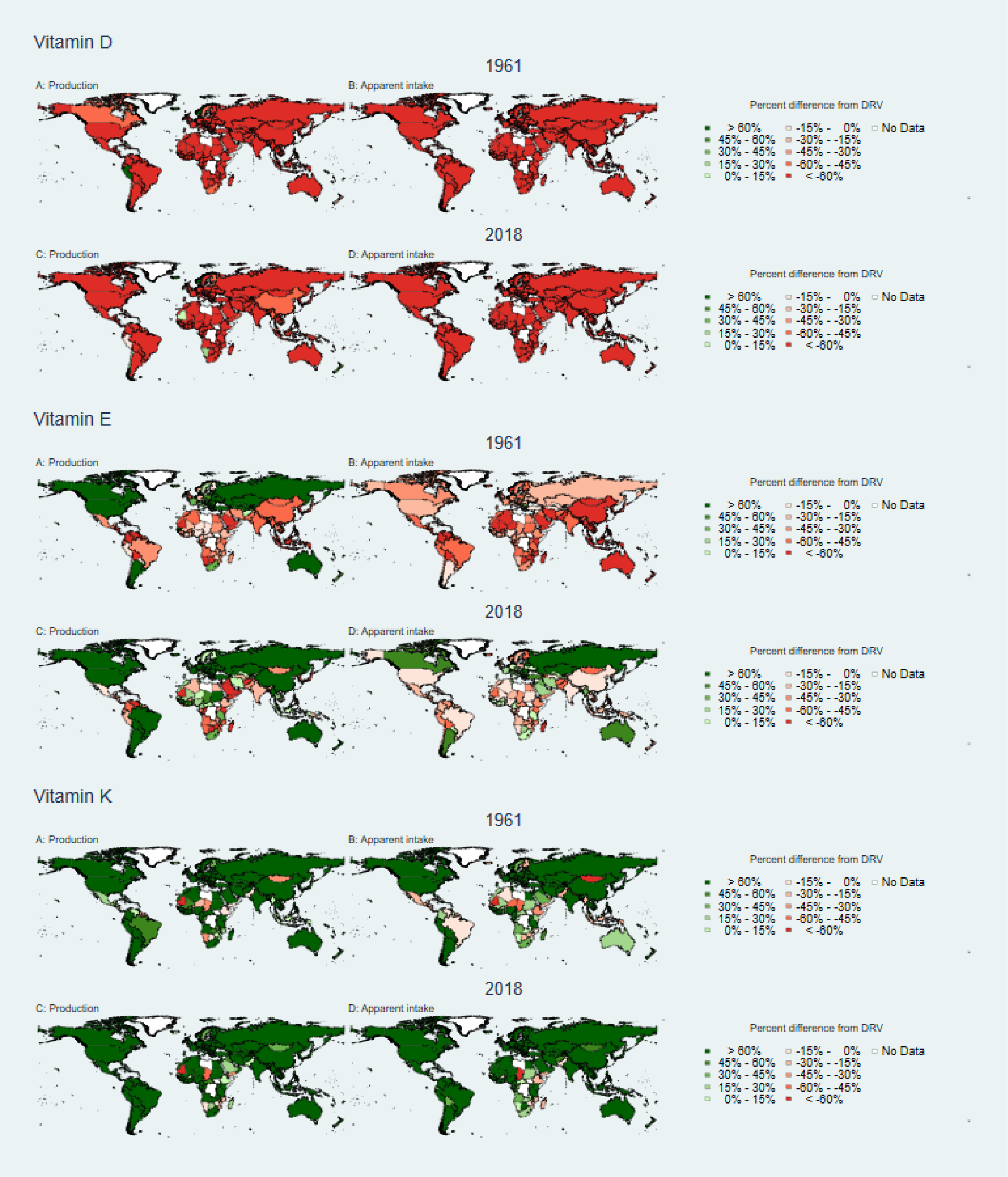

Supplementary Table 3: NBS food composition matches Food composition data come from the Nutrient Database for Standard Reference Legacy database (NDB) available at Food Data Central (FDC) (https://fdc.nal.usda.gov), supplemented by the West Africa Food Composition Table, 2019 (https://www.fao.org/infoods/infoods/tables-and-databases/faoinfoods-databases/en) shown in yellow highlight. Matches were based on definitions for FAO Food Balance Sheet (FBS) categories, using food items in primary commodity form or processed and cooked as specified in the FDC item name.

FBS_itemcode	FBS_itemname	fdc_id	ndb_id	fdc_name
2511	Wheat and products	168889	20071	Wheat, hard red spring
2511	Wheat and products	168890	20072	Wheat, hard red winter
2511	Wheat and products	168891	20073	Wheat, soft red winter
2511	Wheat and products	169719	20074	Wheat, hard white
2511	Wheat and products	169720	20075	Wheat, soft white
2511	Wheat and products	169721	20076	Wheat, durum
2511	Wheat and products	169743	20138	Wheat, Kamut khorasan, uncooked
2513	Barley and products	170283	20004	Barley, hulled
2514	Maize and products	168920	20314	Corn grain, white
2514	Maize and products	170288	20014	Corn grain, yellow
2515	Rye and products	168884	20062	Rye grain
2516	Oats	169705	20038	Oats
2516	Oats	173904	8120	Cereals, oats, regular and quick, not fortified, dry
2517	Millet and products	169702	20031	Millet, raw
2518	Sorghum and products	169716	20067	Sorghum grain
2520	Cereals, Other	168874	20035	Quinoa, uncooked
2520	Cereals, Other	168876	20042	Rice, brown, parboiled, dry, Uncle Ben's
2520	Cereals, Other	168883	20054	Rice, white, glutinous, unenriched, uncooked
2520	Cereals, Other	168884	20062	Rye grain
2520	Cereals, Other	168889	20071	Wheat, hard red spring
2520	Cereals, Other	168890	20072	Wheat, hard red winter
2520	Cereals, Other	168891	20073	Wheat, soft red winter
2520	Cereals, Other	168920	20314	Corn grain, white
2520	Cereals, Other	168931	20452	Rice, white, short-grain, raw, unenriched
2520	Cereals, Other	169702	20031	Millet, raw
2520	Cereals, Other	169703	20036	Rice, brown, long-grain, raw (Includes foods for USDA's Food Distribution Program)
2520	Cereals, Other	169705	20038	Oats
2520	Cereals, Other	169706	20040	Rice, brown, medium-grain, raw (Includes foods for USDA's Food Distribution Program)
2520	Cereals, Other	169716	20067	Sorghum grain
2520	Cereals, Other	169718	20069	Triticale
2520	Cereals, Other	169719	20074	Wheat, hard white
2520	Cereals, Other	169720	20075	Wheat, soft white
2520	Cereals, Other	169721	20076	Wheat, durum
2520	Cereals, Other	169726	20088	Wild rice, raw
2520	Cereals, Other	169743	20138	Wheat, Kamut khorasan, uncooked
2520	Cereals, Other	169756	20444	Rice, white, long-grain, regular, raw, unenriched
2520	Cereals, Other	169758	20446	Rice, white, long-grain, parboiled, unenriched, dry
2520	Cereals, Other	169760	20450	Rice, white, medium-grain, raw, unenriched
2520	Cereals, Other	170283	20004	Barley, hulled
2520	Cereals, Other	170286	20008	Buckwheat
2520	Cereals, Other	170288	20014	Corn grain, yellow
2520	Cereals, Other	173904	8120	Cereals, oats, regular and quick, not fortified, dry
2531	Potatoes and products	170026	11352	Potatoes, flesh and skin, raw
2531	Potatoes and products	170028	11354	Potatoes, white, flesh and skin, raw
2531	Potatoes and products	170029	11355	Potatoes, red, flesh and skin, raw
2532	Cassava and products	169985	11134	Cassava, raw
2533	Sweet potatoes	168482	11507	Sweet potato, raw, unprepared (Includes foods for USDA's Food Distribution Program)
2534	Sweet potatoes	WAFCT2019	02_049	Sweet potato, orange flesh, raw
2534	Roots, Other	168432	11258	Mountain yam, hawaii, raw
2534	Roots, Other	168482	11507	Sweet potato, raw, unprepared (Includes foods for USDA's Food Distribution Program)
2534	Roots, Other	168490	11697	Arrowroot, raw
2534	Roots, Other	169236	11226	Jerusalem-artichokes, raw
2534	Roots, Other	169308	11518	Taro, raw
2534	Roots, Other	169310	11525	Taro, tahitian, raw
2534	Roots, Other	169401	11991	Yautia (tannier), raw
2534	Roots, Other	169985	11134	Cassava, raw
2534	Roots, Other	170026	11352	Potatoes, flesh and skin, raw
2534	Roots, Other	170028	11354	Potatoes, white, flesh and skin, raw
2534	Roots, Other	170029	11355	Potatoes, red, flesh and skin, raw
2534	Roots, Other	170071	11601	Yam, raw
2534	Roots, Other	170073	11603	Yambean (jicama), raw
2535	Yams	168432	11258	Mountain yam, hawaii, raw
2535	Yams	170071	11601	Yam, raw
2536	Sugar cane	170674	19908	Sugar, turbinado
2537	Sugar beet	170674	19908	Sugar, turbinado
2541	Sugar non-centrifugal	170674	19908	Sugar, turbinado
2542	Sugar (Raw Equivalent)	170674	19908	Sugar, turbinado
2543	Sweeteners, Other	168142	44018	Sweeteners, tabletop, fructose, liquid
2543	Sweeteners, Other	168820	19304	Molasses
2543	Sweeteners, Other	168836	19349	Syrups, corn, dark
2543	Sweeteners, Other	168837	19350	Syrups, corn, light
2543	Sweeteners, Other	169658	19340	Sugars, maple
2543	Sweeteners, Other	169659	19351	Syrups, corn, high-fructose
2543	Sweeteners, Other	169660	19352	Syrups, malt
2543	Sweeteners, Other	169661	19353	Syrups, maple
2543	Sweeteners, Other	169662	19355	Syrups, sorghum
2543	Sweeteners, Other	169896	43216	Sweeteners, tabletop, fructose, dry, powder
2543	Sweeteners, Other	170276	19911	Syrup, maple, Canadian
2543	Sweeteners, Other	170277	19912	Sweetener, syrup, agave
2546	Beans	172425	16078	Mothbeans, mature seeds, raw
2546	Beans	173727	16001	Beans, adzuki, mature seeds, raw
2546	Beans	173734	16014	Beans, black, mature seeds, raw
2546	Beans	173738	16022	Beans, french, mature seeds, raw
2546	Beans	173742	16030	Beans, kidney, california red, mature seeds, raw
2546	Beans	173744	16032	Beans, kidney, red, mature seeds, raw
2546	Beans	173745	16037	Beans, navy, mature seeds, raw
2546	Beans	173748	16040	Beans, pink, mature seeds, raw
2546	Beans	173749	16045	Beans, small white, mature seeds, raw
2546	Beans	173751	16047	Beans, yellow, mature seeds, raw

Supplementary Table 3 (continued): NBS food composition matches

FBS_itemcode	FBS_itemname	fdc_id	ndb_id	fdc_name
2546	Beans	174252	16071	Lima beans, large, mature seeds, raw
2546	Beans	174255	16074	Lima beans, thin seeded (baby), mature seeds, raw
2546	Beans	174256	16080	Mung beans, mature seeds, raw
2546	Beans	174259	16083	Mungo beans, mature seeds, raw
2546	Beans	175186	16016	Beans, black turtle, mature seeds, raw
2546	Beans	175189	16019	Beans, cranberry (roman), mature seeds, raw
2546	Beans	175190	16024	Beans, great northern, mature seeds, raw (Includes foods for USDA's Food Distribution Program)
2546	Beans	175193	16027	Beans, kidney, all types, mature seeds, raw
2546	Beans	175196	16035	Beans, kidney, royal red, mature seeds, raw
2546	Beans	175199	16042	Beans, pinto, mature seeds, raw (Includes foods USDA's food distribution program)
2546	Beans	175202	16049	Beans, white, mature seeds, raw
2547	Peas	172428	16085	Peas, green, split, mature seeds, raw
2549	Pulses, Other and products	172420	16069	Lentils, raw
2549	Pulses, Other and products	172423	16076	lupins, mature seeds, raw
2549	Pulses, Other and products	172425	16078	Mothbeans, mature seeds, raw
2549	Pulses, Other and products	172428	16085	Peas, green, split, mature seeds, raw
2549	Pulses, Other and products	172436	16101	Pigeon peas, (red gram), mature seeds, raw
2549	Pulses, Other and products	173727	16001	Beans, adzuki, mature seeds, raw
2549	Pulses, Other and products	173734	16014	Beans, black, mature seeds, raw
2549	Pulses, Other and products	173738	16022	Beans, french, mature seeds, raw
2549	Pulses, Other and products	173742	16030	Beans, kidney, california red, mature seeds, raw
2549	Pulses, Other and products	173744	16032	Beans, kidney, red, mature seeds, raw
2549	Pulses, Other and products	173745	16037	Beans, navy, mature seeds, raw
2549	Pulses, Other and products	173748	16040	Beans, pink, mature seeds, raw
2549	Pulses, Other and products	173749	16045	Beans, small white, mature seeds, raw
2549	Pulses, Other and products	173751	16047	Beans, yellow, mature seeds, raw
2549	Pulses, Other and products	173756	16056	Chickpeas (garbanzo beans, bengal gram), mature seeds, raw
2549	Pulses, Other and products	173758	16062	Cowpeas, common (blackeyes, crowder, southern), mature seeds, raw
2549	Pulses, Other and products	174252	16071	Lima beans, large, mature seeds, raw
2549	Pulses, Other and products	174255	16074	Lima beans, thin seeded (baby), mature seeds, raw
2549	Pulses, Other and products	174256	16080	Mung beans, mature seeds, raw
2549	Pulses, Other and products	174259	16083	Mungo beans, mature seeds, raw
2549	Pulses, Other and products	174281	16133	Yardlong beans, mature seeds, raw
2549	Pulses, Other and products	174283	16135	Winged beans, mature seeds, raw
2549	Pulses, Other and products	174284	16144	Lentils, pink or red, raw
2549	Pulses, Other and products	175186	16016	Beans, black turtle, mature seeds, raw
2549	Pulses, Other and products	175189	16019	Beans, cranberry (roman), mature seeds, raw
2549	Pulses, Other and products	175190	16024	Beans, great northern, mature seeds, raw (Includes foods for USDA's Food Distribution Program)
2549	Pulses, Other and products	175193	16027	Beans, kidney, all types, mature seeds, raw
2549	Pulses, Other and products	175196	16035	Beans, kidney, royal red, mature seeds, raw
2549	Pulses, Other and products	175199	16042	Beans, pinto, mature seeds, raw (Includes foods USDA's food distribution program)
2549	Pulses, Other and products	175202	16049	Beans, white, mature seeds, raw
2549	Pulses, Other and products	175205	16052	Broadbeans (fava beans), mature seeds, raw
2549	Pulses, Other and products	175208	16060	Cowpeas, catjang, mature seeds, raw
2549	Pulses, Other and products	175210	16067	Hyacinth beans, mature seeds, raw
2551	Nuts and products	169408	12175	Nuts, chestnuts, japanese, dried
2551	Nuts and products	169413	12202	Nuts, chestnuts, japanese, raw
2551	Nuts and products	170157	12058	Nuts, acorns, raw
2551	Nuts and products	170162	12087	Nuts, cashew nuts, raw
2551	Nuts and products	170164	12093	Nuts, chestnuts, chinese, raw
2551	Nuts and products	170175	12128	Nuts, ginkgo nuts, dried
2551	Nuts and products	170177	12130	Nuts, hickorynuts, dried
2551	Nuts and products	170178	12131	Nuts, macadamia nuts, raw
2551	Nuts and products	170182	12142	Nuts, pecans
2551	Nuts and products	170184	12151	Nuts, pistachio nuts, raw
2551	Nuts and products	170186	12154	Nuts, walnuts, black, dried
2551	Nuts and products	170187	12155	Nuts, walnuts, english
2551	Nuts and products	170565	12059	Nuts, acorns, dried
2551	Nuts and products	170567	12061	Nuts, almonds
2551	Nuts and products	170569	12078	Nuts, brazilnuts, dried, unblanched
2551	Nuts and products	170570	12084	Nuts, butternuts, dried
2551	Nuts and products	170574	12097	Nuts, chestnuts, european, raw, unpeeled
2551	Nuts and products	170576	12099	Nuts, chestnuts, european, dried, unpeeled
2551	Nuts and products	170581	12120	Nuts, hazelnuts or filberts
2551	Nuts and products	170584	12127	Nuts, ginkgo nuts, raw
2551	Nuts and products	170590	12145	Nuts, pilinuts, dried
2551	Nuts and products	170591	12147	Nuts, pine nuts, dried
2551	Nuts and products	170592	12149	Nuts, pine nuts, pinyon, dried
2555	Soyabeans	174270	16108	Soybeans, mature seeds, raw
2556	Groundnuts (Shelled Eq)	172430	16087	Peanuts, all types, raw
2556	Groundnuts (Shelled Eq)	172432	16093	Peanuts, valencia, raw
2556	Groundnuts (Shelled Eq)	172434	16095	Peanuts, virginia, raw
2556	Groundnuts (Shelled Eq)	174263	16091	Peanuts, spanish, raw
2557	Sunflower seed	170562	12036	Seeds, sunflower seed kernels, dried
2558	Rape and Mustardseed	170929	2024	Spices, mustard seed, ground
2559	Cottonseed	170596	12160	Seeds, cottonseed kernels, roasted (glandless)
2560	Coconuts - Incl Copra	170169	12104	Nuts, cocounut meat, raw
2561	Sesame seed	170150	12023	Seeds, sesame seeds, whole, dried
2562	Palm kernels	WAFCT2019	06_029	Oil palm, nut (kernel), shelled, raw
2563	Olives (including preserved	169094	9193	Olives, ripe, canned (small-extra large)
2563	Olives (including preserved	169095	9194	Olives, ripe, canned (jumbo-super colossal)
2570	Oilcrops, Other	169094	9193	Olives, ripe, canned (small-extra large)
2570	Oilcrops, Other	169095	9194	Olives, ripe, canned (jumbo-super colossal)
2570	Oilcrops, Other	169414	12220	Seeds, flaxseed
2570	Oilcrops, Other	170148	12012	Seeds, hemp seed, hulled
2570	Oilcrops, Other	170150	12023	Seeds, sesame seeds, whole, dried
2570	Oilcrops, Other	170161	12077	Nuts, beechnuts, dried
2570	Oilcrops, Other	170169	12104	Nuts, cocounut meat, raw

Supplementary Table 3 (continued): NBS food composition matches

FBS_itemcode	FBS_itemname	fdc_id	ndb_id	fdc_name
2570	Oilcrops, Other	170558	12021	Seeds, safflower seed kernels, dried
2570	Oilcrops, Other	170562	12036	Seeds, sunflower seed kernels, dried
2570	Oilcrops, Other	170596	12160	Seeds, cottonseed kernels, roasted (glandless)
2570	Oilcrops, Other	170929	2024	Spices, mustard seed, ground
2570	Oilcrops, Other	171330	2033	Spices, poppy seed
2570	Oilcrops, Other	172430	16087	Peanuts, all types, raw
2570	Oilcrops, Other	172432	16093	Peanuts, valencia, raw
2570	Oilcrops, Other	172434	16095	Peanuts, virginia, raw
2570	Oilcrops, Other	174263	16091	Peanuts, spanish, raw
2570	Oilcrops, Other	174270	16108	Soybeans, mature seeds, raw
2571	Soyabean Oil	171012	4034	Oil, soybean, salad or cooking, (partially hydrogenated)
2571	Soyabean Oil	171411	4044	Oil, soybean, salad or cooking
2571	Soyabean Oil	171438	4699	Oil, industrial, soy, low linolenic
2571	Soyabean Oil	171439	4700	Oil, industrial, soy, ultra low linolenic
2571	Soyabean Oil	171440	4701	Oil, industrial, soy, fully hydrogenated
2571	Soyabean Oil	172362	4652	Oil, industrial, soy (partially hydrogenated), all purpose
2571	Soyabean Oil	172370	4669	Oil, vegetable, soybean, refined
2571	Soyabean Oil	173598	4650	Oil, industrial, soy, refined, for woks and light frying
2572	Groundnut Oil	171410	4042	Oil, peanut, salad or cooking
2573	Sunflowerseed Oil	171017	4060	Oil, sunflower, linoleic (less than 60%)
2573	Sunflowerseed Oil	171025	4506	Oil, sunflower, linoleic, (approx. 65%)
2573	Sunflowerseed Oil	172328	4545	Oil, sunflower, linoleic, (partially hydrogenated)
2573	Sunflowerseed Oil	172338	4584	Oil, sunflower, high oleic (70% and over)
2573	Sunflowerseed Oil	172357	4642	Oil, industrial, mid-oleic, sunflower
2574	Rape and Mustard Oil	171033	4678	Oil, vegetable, Natreon canola, high stability, non trans, high oleic (70%)
2574	Rape and Mustard Oil	171042	4698	Oil, industrial, canola, high oleic
2574	Rape and Mustard Oil	172336	4582	Oil, canola
2574	Rape and Mustard Oil	172337	4583	Oil, mustard
2574	Rape and Mustard Oil	172359	4644	Oil, industrial, canola for salads, woks and light frying
2574	Rape and Mustard Oil	172360	4645	Oil, industrial, canola (partially hydrogenated) oil for deep fat frying
2575	Cottonseed Oil	171024	4502	Oil, cottonseed, salad or cooking
2575	Cottonseed Oil	171441	4702	Oil, industrial, cottonseed, fully hydrogenated
2576	Palmkernel Oil	171422	4513	Vegetable oil, palm kernel
2576	Palmkernel Oil	171428	4534	Oil, babassu
2576	Palmkernel Oil	172366	4660	Oil, industrial, palm kernel (hydrogenated) , used for whipped toppings, non-dairy
2576	Palmkernel Oil	173600	4656	Oil, industrial, palm kernel, confection fat, uses similar to high quality cocoa butter
2576	Palmkernel Oil	173601	4657	Oil, industrial, palm kernel (hydrogenated), confection fat, uses similar to 95 degree hard butter
2576	Palmkernel Oil	173602	4658	Oil, industrial, palm kernel (hydrogenated), confection fat, intermediate grade product
2576	Palmkernel Oil	173603	4663	Oil, industrial, palm kernel (hydrogenated), filling fat
2577	Palm Oil	171015	4055	Oil, palm
2577	Palm Oil	WAFCT2019	11_004	Palm oil, red
2578	Coconut Oil	171412	4047	Oil, coconut
2578	Coconut Oil	172365	4659	Oil, industrial, coconut, confection fat, typical basis for ice cream coatings
2578	Coconut Oil	172367	4661	Oil, industrial, coconut (hydrogenated), used for whipped toppings and coffee whiteners
2578	Coconut Oil	173595	4646	Oil, industrial, coconut, principal uses candy coatings, oil sprays, roasting nuts
2579	Sesameseed Oil	171016	4058	Oil, sesame, salad or cooking
2580	Olive Oil	171413	4053	Oil, olive, salad or cooking
2581	Ricebran Oil	171013	4037	Oil, rice bran
2582	Maize Germ Oil	171029	4518	Oil, corn, industrial and retail, all purpose salad or cooking
2586	Oilcrops Oil, Other	167702	42231	Oil, flaxseed, cold pressed
2586	Oilcrops Oil, Other	171011	4031	Shortening, household, soybean (partially hydrogenated)-cottonseed (partially hydrogenated)
2586	Oilcrops Oil, Other	171014	4038	Oil, wheat germ
2586	Oilcrops Oil, Other	171018	4073	Margarine, regular, hard, soybean (hydrogenated)
2586	Oilcrops Oil, Other	171026	4510	Oil, safflower, salad or cooking, linoleic, (over 70%)
2586	Oilcrops Oil, Other	171027	4511	Oil, safflower, salad or cooking, high oleic (primary safflower oil of commerce)
2586	Oilcrops Oil, Other	171028	4517	Oil, grapeseed
2586	Oilcrops Oil, Other	171030	4528	Oil, walnut
2586	Oilcrops Oil, Other	171031	4529	Oil, almond
2586	Oilcrops Oil, Other	171032	4530	Oil, apricot kernel
2586	Oilcrops Oil, Other	171421	4501	Oil, cocoa butter
2586	Oilcrops Oil, Other	171423	4514	Oil, poppyseed
2586	Oilcrops Oil, Other	171424	4515	Oil, tomatoseed
2586	Oilcrops Oil, Other	171425	4516	Oil, teaseed
2586	Oilcrops Oil, Other	171427	4532	Oil, hazelnut
2586	Oilcrops Oil, Other	171429	4536	Oil, sheanut
2586	Oilcrops Oil, Other	171432	4684	Margarine, 80% fat, tub, CANOLA HARVEST Soft Spread (canola, palm and palm kernel oils)
2586	Oilcrops Oil, Other	172329	4546	Shortening bread, soybean (hydrogenated) and cottonseed
2586	Oilcrops Oil, Other	172330	4548	Shortening cake mix, soybean (hydrogenated) and cottonseed (hydrogenated)
2586	Oilcrops Oil, Other	172331	4556	Shortening frying (heavy duty), palm (hydrogenated)
2586	Oilcrops Oil, Other	172332	4559	Shortening household soybean (hydrogenated) and palm
2586	Oilcrops Oil, Other	172333	4560	Shortening frying (heavy duty), soybean (hydrogenated), linoleic (less than 1%)
2586	Oilcrops Oil, Other	172334	4570	Shortening, confectionery, fractionated palm
2586	Oilcrops Oil, Other	172335	4572	Oil, nutmeg butter
2586	Oilcrops Oil, Other	172349	4618	Margarine, regular, 80% fat, composite, tub, without salt
2586	Oilcrops Oil, Other	172369	4668	Margarine, industrial, soy and partially hydrogenated soy oil, use for baking, sauces and candy
2586	Oilcrops Oil, Other	173563	4541	Oil, cupu assu
2586	Oilcrops Oil, Other	173568	4551	Shortening confectionery, coconut (hydrogenated) and or palm kernel (hydrogenated)
2586	Oilcrops Oil, Other	173569	4554	Shortening industrial, soybean (hydrogenated) and cottonseed
2586	Oilcrops Oil, Other	173570	4573	Oil, ucuhuba butter
2586	Oilcrops Oil, Other	173573	4581	Oil, avocado
2586	Oilcrops Oil, Other	173574	4586	Shortening, special purpose for cakes and frostings, soybean (hydrogenated)
2586	Oilcrops Oil, Other	173575	4587	Shortening, special purpose for baking, soybean (hydrogenated) palm and cottonseed
2586	Oilcrops Oil, Other	173579	4595	Shortening, multipurpose, soybean (hydrogenated) and palm (hydrogenated)
2586	Oilcrops Oil, Other	173584	4615	Shortening, vegetable, household, composite
2586	Oilcrops Oil, Other	173585	4617	Margarine, regular, 80% fat, composite, stick, without salt
2586	Oilcrops Oil, Other	173587	4628	Margarine, 80% fat, stick, includes regular and hydrogenated corn and soybean oils
2586	Oilcrops Oil, Other	173599	4655	Margarine-like shortening, industrial, soy (partially hydrogenated), cottonseed, and soy, principal use flaky pastries
2586	Oilcrops Oil, Other	173605	4665	Margarine, industrial, non-dairy, cottonseed, soy oil (partially hydrogenated), for flaky pastries
2586	Oilcrops Oil, Other	173606	4666	Shortening, industrial, soy (partially hydrogenated) and corn for frying

Supplementary Table 3 (continued): NBS food composition matches

FBS_itemcode	FBS_itemname	fdc_id	ndb_id	fdc_name
2586	Oilcrops Oil, Other	173607	4667	Shortening, industrial, soy (partially hydrogenated) for baking and confections
2601	Tomatoes and products	170096	11696	Tomatoes, yellow, raw
2601	Tomatoes and products	170456	11527	Tomatoes, green, raw
2601	Tomatoes and products	170457	11529	Tomatoes, red, ripe, raw, year round average
2601	Tomatoes and products	170502	11695	Tomatoes, orange, raw
2601	Tomatoes and products	321360	100147	Tomatoes, grape, raw
2602	Onions	170000	11282	Onions, raw
2602	Onions	170008	11294	Onions, sweet, raw
2605	Vegetables, Other	167758	9307	Rhubarb, raw
2605	Vegetables, Other	167765	9326	Watermelon, raw
2605	Vegetables, Other	168389	11011	Asparagus, raw
2605	Vegetables, Other	168396	11031	Lima beans, immature seeds, raw
2605	Vegetables, Other	168405	11197	Cowpeas, young pods with seeds, raw
2605	Vegetables, Other	168407	11203	Cress, garden, raw
2605	Vegetables, Other	168409	11205	Cucumber, with peel, raw
2605	Vegetables, Other	168412	11213	Endive, raw
2605	Vegetables, Other	168421	11233	Kale, raw
2605	Vegetables, Other	168422	11239	Mushrooms, Chanterelle, raw
2605	Vegetables, Other	168423	11240	Mushrooms, morel, raw
2605	Vegetables, Other	168424	11241	Kohlrabi, raw
2605	Vegetables, Other	168429	11250	Lettuce, butterhead (includes boston and bibb types), raw
2605	Vegetables, Other	168431	11257	Lettuce, red leaf, raw
2605	Vegetables, Other	168434	11266	Mushrooms, brown, italian, or crimini, raw
2605	Vegetables, Other	168438	11274	Mustard spinach, (tendergreen), raw
2605	Vegetables, Other	168440	11276	New Zealand spinach, raw
2605	Vegetables, Other	168448	11422	Pumpkin, raw
2605	Vegetables, Other	168451	11430	Radishes, oriental, raw
2605	Vegetables, Other	168454	11435	Rutabagas, raw
2605	Vegetables, Other	168462	11457	Spinach, raw
2605	Vegetables, Other	168538	11900	Corn, sweet, white, raw
2605	Vegetables, Other	168565	11953	Squash, zucchini, baby, raw
2605	Vegetables, Other	168568	11960	Carrots, baby, raw
2605	Vegetables, Other	168574	11973	Beans, fava, in pod, raw
2605	Vegetables, Other	168576	11979	Peppers, jalapeno, raw
2605	Vegetables, Other	168580	11987	Mushrooms, oyster, raw
2605	Vegetables, Other	169092	9181	Melons, cantaloupe, raw
2605	Vegetables, Other	169093	9183	Melons, casaba, raw
2605	Vegetables, Other	169145	11080	Beets, raw
2605	Vegetables, Other	169205	11007	Artichokes, (globe or french), raw
2605	Vegetables, Other	169210	11026	Bamboo shoots, raw
2605	Vegetables, Other	169220	11191	Cowpeas (blackeyes), immature seeds, raw
2605	Vegetables, Other	169222	11199	Yardlong bean, raw
2605	Vegetables, Other	169228	11209	Eggplant, raw
2605	Vegetables, Other	169230	11215	Garlic, raw
2605	Vegetables, Other	169242	11238	Mushrooms, shiitake, raw
2605	Vegetables, Other	169246	11246	Leeks, (bulb and lower leaf-portion), raw
2605	Vegetables, Other	169247	11251	Lettuce, cos or romaine, raw
2605	Vegetables, Other	169248	11252	Lettuce, iceberg (includes crisphead types), raw
2605	Vegetables, Other	169249	11253	Lettuce, green leaf, raw
2605	Vegetables, Other	169251	11260	Mushrooms, white, raw
2605	Vegetables, Other	169255	11265	Mushrooms, portabella, raw
2605	Vegetables, Other	169256	11270	Mustard greens, raw
2605	Vegetables, Other	169260	11278	Okra, raw
2605	Vegetables, Other	169276	11429	Radishes, raw
2605	Vegetables, Other	169277	11437	Salsify, (vegetable oyster), raw
2605	Vegetables, Other	169320	11722	Beans, snap, yellow, raw
2605	Vegetables, Other	169335	11749	Cabbage, common (danish, domestic, and pointed types), freshly harvest, raw
2605	Vegetables, Other	169382	11950	Mushrooms, enoki, raw
2605	Vegetables, Other	169383	11951	Peppers, sweet, yellow, raw
2605	Vegetables, Other	169385	11957	Fennel, bulb, raw
2605	Vegetables, Other	169389	11965	Cauliflower, green, raw
2605	Vegetables, Other	169394	11976	Pepper, banana, raw
2605	Vegetables, Other	169395	11977	Peppers, serrano, raw
2605	Vegetables, Other	169403	11993	Mushrooms, maitake, raw
2605	Vegetables, Other	169404	11994	Broccoli, chinese, raw
2605	Vegetables, Other	169911	9184	Melons, honeydew, raw
2605	Vegetables, Other	169961	11052	Beans, snap, green, raw
2605	Vegetables, Other	169975	11109	Cabbage, raw
2605	Vegetables, Other	169977	11112	Cabbage, red, raw
2605	Vegetables, Other	169979	11119	Cabbage, chinese (pe-tsai), raw
2605	Vegetables, Other	169981	11122	Cardoon, raw
2605	Vegetables, Other	169986	11135	Cauliflower, raw
2605	Vegetables, Other	169988	11143	Celery, raw
2605	Vegetables, Other	169990	11145	Celtuce, raw
2605	Vegetables, Other	169991	11147	Chard, swiss, raw
2605	Vegetables, Other	169992	11152	Chicory greens, raw
2605	Vegetables, Other	169993	11154	Chicory roots, raw
2605	Vegetables, Other	169994	11156	Chives, raw
2605	Vegetables, Other	169998	11167	Corn, sweet, yellow, raw
2605	Vegetables, Other	170000	11282	Onions, raw
2605	Vegetables, Other	170005	11291	Onions, spring or scallions (includes tops and bulb), raw
2605	Vegetables, Other	170006	11292	Onions, young green, tops only
2605	Vegetables, Other	170007	11293	Onions, welsh, raw
2605	Vegetables, Other	170008	11294	Onions, sweet, raw
2605	Vegetables, Other	170010	11300	Peas, edible-podded, raw
2605	Vegetables, Other	170061	11568	Turnip greens, raw
2605	Vegetables, Other	170068	11591	Watercress, raw
2605	Vegetables, Other	170081	11637	Radishes, white icicle, raw
2605	Vegetables, Other	170096	11696	Tomatoes, yellow, raw

Supplementary Table 3 (continued): NBS food composition matches

FBS_itemcode	FBS_itemname	fdc_id	ndb_id	fdc_name
2605	Vegetables, Other	170375	11086	Beet greens, raw
2605	Vegetables, Other	170377	11088	Broadbeans, immature seeds, raw
2605	Vegetables, Other	170379	11090	Broccoli, raw
2605	Vegetables, Other	170383	11098	Brussels sprouts, raw
2605	Vegetables, Other	170388	11114	Cabbage, savoy, raw
2605	Vegetables, Other	170390	11116	Cabbage, chinese (pak-choi), raw
2605	Vegetables, Other	170392	11118	Cabbage, kimchi
2605	Vegetables, Other	170393	11124	Carrots, raw
2605	Vegetables, Other	170400	11141	Celeriac, raw
2605	Vegetables, Other	170404	11151	Chicory, witloof, raw
2605	Vegetables, Other	170406	11161	Collards, raw
2605	Vegetables, Other	170416	11297	Parsley, fresh
2605	Vegetables, Other	170417	11298	Parsnips, raw
2605	Vegetables, Other	170419	11304	Peas, green, raw
2605	Vegetables, Other	170427	11333	Peppers, sweet, green, raw
2605	Vegetables, Other	170456	11527	Tomatoes, green, raw
2605	Vegetables, Other	170457	11529	Tomatoes, red, ripe, raw, year round average
2605	Vegetables, Other	170465	11564	Turnips, raw
2605	Vegetables, Other	170487	11641	Squash, summer, all varieties, raw
2605	Vegetables, Other	170489	11643	Squash, winter, all varieties, raw
2605	Vegetables, Other	170497	11670	Peppers, hot chili, green, raw
2605	Vegetables, Other	170499	11677	Shallots, raw
2605	Vegetables, Other	170502	11695	Tomatoes, orange, raw
2605	Vegetables, Other	172238	2054	Capers, canned
2605	Vegetables, Other	321360	100147	Tomatoes, grape, raw
2611	Oranges, Mandarines	168195	9433	Clementines, raw
2611	Oranges, Mandarines	169097	9200	Oranges, raw, all commercial varieties
2611	Oranges, Mandarines	169105	9218	Tangerines, (mandarin oranges), raw
2612	Lemons, Limes and products	167746	9150	Lemons, raw, without peel
2612	Lemons, Limes and products	168155	9159	Limes, raw
2613	Grapefruit and products	167754	9295	Pummelo, raw
2613	Grapefruit and products	173033	9111	Grapefruit, raw, pink and red and white, all areas
2614	Citrus, Other	168154	9149	Kumquats, raw
2615	Bananas	173944	9040	Bananas, raw
2616	Plantains	168215	9542	Plantains, green, raw
2616	Plantains	169130	9277	Plantains, yellow, raw
2617	Apples and products	171688	9003	Apples, raw, with skin (Includes foods for USDA's Food Distribution Program)
2618	Pineapples and products	169124	9266	Pineapple, raw, all varieties
2619	Dates	168191	9421	Dates, medjool
2619	Dates	171726	9087	Dates, deglet noor
2620	Grapes and products (excl w	173040	9129	Grapes, muscadine, raw
2620	Grapes and products (excl w	174682	9131	Grapes, american type (slip skin), raw
2620	Grapes and products (excl w	174683	9132	Grapes, red or green (European type, such as Thompson seedless), raw
2625	Fruits, Other	167640	35155	Blueberries, wild, raw (Alaska Native)
2625	Fruits, Other	167746	9150	Lemons, raw, without peel
2625	Fruits, Other	167750	9287	Prickly pears, raw
2625	Fruits, Other	167754	9295	Pummelo, raw
2625	Fruits, Other	167755	9302	Raspberries, raw
2625	Fruits, Other	167759	9313	Sapodilla, raw
2625	Fruits, Other	167760	9314	Sapote, mamey, raw
2625	Fruits, Other	167762	9316	Strawberries, raw
2625	Fruits, Other	167763	9322	Tamarinds, raw
2625	Fruits, Other	167790	9450	Naranjilla (lulo) pulp, frozen, unsweetened
2625	Fruits, Other	168153	9148	Kiwifruit, green, raw
2625	Fruits, Other	168154	9149	Kumquats, raw
2625	Fruits, Other	168155	9159	Limes, raw
2625	Fruits, Other	168163	9296	Quinces, raw
2625	Fruits, Other	168176	9334	Feijoa, raw
2625	Fruits, Other	168191	9421	Dates, medjool
2625	Fruits, Other	168192	9422	Durian, raw or frozen
2625	Fruits, Other	168195	9433	Clementines, raw
2625	Fruits, Other	168211	9520	Kiwifruit, ZESPRI SunGold, raw
2625	Fruits, Other	168215	9542	Plantains, green, raw
2625	Fruits, Other	168982	35015	Blackberries, wild, raw (Alaska Native)
2625	Fruits, Other	168998	35203	Rose Hips, wild (Northern Plains Indians)
2625	Fruits, Other	169086	9164	Litchis, raw
2625	Fruits, Other	169088	9167	Loganberries, frozen
2625	Fruits, Other	169089	9172	Longans, raw
2625	Fruits, Other	169097	9200	Oranges, raw, all commercial varieties
2625	Fruits, Other	169105	9218	Tangerines, (mandarin oranges), raw
2625	Fruits, Other	169108	9231	Passion-fruit, (granadilla), purple, raw
2625	Fruits, Other	169118	9252	Pears, raw
2625	Fruits, Other	169124	9266	Pineapple, raw, all varieties
2625	Fruits, Other	169130	9277	Plantains, yellow, raw
2625	Fruits, Other	169134	9286	Pomegranates, raw
2625	Fruits, Other	169805	35030	Cranberry, low bush or lingenberry, raw (Alaska Native)
2625	Fruits, Other	169808	35043	Huckleberries, raw (Alaska Native)
2625	Fruits, Other	169908	9174	Loquats, raw
2625	Fruits, Other	169909	9175	Mammy-apple, (mamey), raw
2625	Fruits, Other	169910	9176	Mangos, raw
2625	Fruits, Other	169913	9190	Mulberries, raw
2625	Fruits, Other	169914	9191	Nectarines, raw
2625	Fruits, Other	169915	9192	Oheloberries, raw
2625	Fruits, Other	169926	9226	Papayas, raw
2625	Fruits, Other	169928	9236	Peaches, yellow, raw
2625	Fruits, Other	169941	9263	Persimmons, japanese, raw
2625	Fruits, Other	169943	9265	Persimmons, native, raw
2625	Fruits, Other	169949	9279	Plums, raw
2625	Fruits, Other	171688	9003	Apples, raw, with skin (Includes foods for USDA's Food Distribution Program)

Supplementary Table 3 (continued): NBS food composition matches

FBS_itemcode	FBS_itemname	fdc_id	ndb_id	fdc_name
2625	Fruits, Other	171697	9021	Apricots, raw
2625	Fruits, Other	171705	9037	Avocados, raw, all commercial varieties
2625	Fruits, Other	171711	9050	Blueberries, raw
2625	Fruits, Other	171714	9059	Breadfruit, raw
2625	Fruits, Other	171715	9060	Carambola, (starfruit), raw
2625	Fruits, Other	171719	9070	Cherries, sweet, raw
2625	Fruits, Other	171722	9078	Cranberries, raw
2625	Fruits, Other	171725	9086	Custard-apple, (bullock's-heart), raw
2625	Fruits, Other	171726	9087	Dates, deglet noor
2625	Fruits, Other	171727	9088	Elderberries, raw
2625	Fruits, Other	173021	9089	Figs, raw
2625	Fruits, Other	173030	9107	Gooseberries, raw
2625	Fruits, Other	173033	9111	Grapefruit, raw, pink and red and white, all areas
2625	Fruits, Other	173040	9129	Grapes, muscadine, raw
2625	Fruits, Other	173044	9139	Guavas, common, raw
2625	Fruits, Other	173944	9040	Bananas, raw
2625	Fruits, Other	173946	9042	Blackberries, raw
2625	Fruits, Other	173953	9062	Cherimoya, raw
2625	Fruits, Other	173954	9063	Cherries, sour, red, raw
2625	Fruits, Other	173963	9083	Currants, european black, raw
2625	Fruits, Other	173964	9084	Currants, red and white, raw
2625	Fruits, Other	174682	9131	Grapes, american type (slip skin), raw
2625	Fruits, Other	174683	9132	Grapes, red or green (European type, such as Thompson seedless), raw
2625	Fruits, Other	174687	9144	Jackfruit, raw
2630	Coffee and products	171890	14209	Beverages, coffee, brewed, prepared with tap water
2633	Cocoa Beans and products	169593	19165	Cocoa, dry powder, unsweetened
2633	Cocoa Beans and products	171421	4501	Oil, cocoa butter
2635	Tea (including mate)	171917	14278	Beverages, tea, green, brewed, regular
2635	Tea (including mate)	174120	14185	Beverages, tea, Oolong, brewed
2635	Tea (including mate)	174155	14544	Beverages, tea, black, brewed, prepared with distilled water
2640	Pepper	170931	2030	Spices, pepper, black
2640	Pepper	170933	2032	Spices, pepper, white
2641	Pimento	170932	2031	Spices, pepper, red or cayenne
2641	Pimento	171315	2001	Spices, allspice, ground
2641	Pimento	171319	2009	Spices, chili powder
2641	Pimento	171329	2028	Spices, paprika
2642	Cloves	171321	2011	Spices, cloves, ground
2645	Spices, Other	169231	11216	Ginger root, raw
2645	Spices, Other	170917	2004	Spices, bay leaf
2645	Spices, Other	170918	2005	Spices, caraway seed
2645	Spices, Other	170919	2006	Spices, cardamom
2645	Spices, Other	170920	2007	Spices, celery seed
2645	Spices, Other	170921	2012	Spices, coriander leaf, dried
2645	Spices, Other	170922	2013	Spices, coriander seed
2645	Spices, Other	170923	2014	Spices, cumin seed
2645	Spices, Other	170924	2015	Spices, curry powder
2645	Spices, Other	170925	2016	Spices, dill seed
2645	Spices, Other	170926	2021	Spices, ginger, ground
2645	Spices, Other	170927	2022	Spices, mace, ground
2645	Spices, Other	170928	2023	Spices, marjoram, dried
2645	Spices, Other	170930	2029	Spices, parsley, dried
2645	Spices, Other	170931	2030	Spices, pepper, black
2645	Spices, Other	170932	2031	Spices, pepper, red or cayenne
2645	Spices, Other	170933	2032	Spices, pepper, white
2645	Spices, Other	170934	2037	Spices, saffron
2645	Spices, Other	170935	2038	Spices, sage, ground
2645	Spices, Other	170936	2039	Spices, savory, ground
2645	Spices, Other	170937	2041	Spices, tarragon, dried
2645	Spices, Other	170938	2042	Spices, thyme, dried
2645	Spices, Other	171315	2001	Spices, allspice, ground
2645	Spices, Other	171316	2002	Spices, anise seed
2645	Spices, Other	171317	2003	Spices, basil, dried
2645	Spices, Other	171318	2008	Spices, chervil, dried
2645	Spices, Other	171319	2009	Spices, chili powder
2645	Spices, Other	171320	2010	Spices, cinnamon, ground
2645	Spices, Other	171321	2011	Spices, cloves, ground
2645	Spices, Other	171322	2017	Spices, dill weed, dried
2645	Spices, Other	171323	2018	Spices, fennel seed
2645	Spices, Other	171324	2019	Spices, fenugreek seed
2645	Spices, Other	171325	2020	Spices, garlic powder
2645	Spices, Other	171326	2025	Spices, nutmeg, ground
2645	Spices, Other	171327	2026	Spices, onion powder
2645	Spices, Other	171328	2027	Spices, oregano, dried
2645	Spices, Other	171329	2028	Spices, paprika
2645	Spices, Other	171331	2034	Spices, poultry seasoning
2645	Spices, Other	171332	2035	Spices, pumpkin pie spice
2645	Spices, Other	171333	2036	Spices, rosemary, dried
2645	Spices, Other	172231	2043	Spices, turmeric, ground
2655	Wine	173176	14057	Alcoholic beverage, wine, dessert, sweet
2655	Wine	173185	14084	Alcoholic beverage, wine, table, all
2655	Wine	174157	14553	Beverages, Wine, non-alcoholic
2655	Wine	175112	14536	Alcoholic beverage, wine, dessert, dry
2656	Beer	168746	14003	Alcoholic beverage, beer, regular, all
2656	Beer	168749	14006	Alcoholic beverage, beer, light
2656	Beer	169575	14013	Alcoholic beverage, beer, light, low carb
2656	Beer	171906	14251	Alcoholic beverages, beer, higher alcohol
2656	Beer	171907	14252	Beverages, Malt liquor beverage
2656	Beer	174141	14239	Alcoholic beverage, malt beer, hard lemonade
2656	Beer	174145	14248	Alcoholic beverage, beer, light, higher alcohol

Supplementary Table 3 (continued): NBS food composition matches

FBS_itemcode	FBS_itemname	fdc_id	ndb_id	fdc_name
2656	Beer	174863	14305	Malt beverage, includes non-alcoholic beer
2657	Beverages, Fermented	167723	43479	Alcoholic beverage, rice (sake)
2657	Beverages, Fermented	174146	14250	Beverages, AMBER, hard cider
2658	Beverages, Alcoholic	171919	14550	Alcoholic beverage, distilled, all (gin, rum, vodka, whiskey) 86 proof
2658	Beverages, Alcoholic	171920	14551	Alcoholic beverage, distilled, all (gin, rum, vodka, whiskey) 90 proof
2658	Beverages, Alcoholic	173168	14034	Alcoholic beverage, creme de menthe, 72 proof
2658	Beverages, Alcoholic	173663	14532	Alcoholic beverage, distilled, all (gin, rum, vodka, whiskey) 94 proof
2658	Beverages, Alcoholic	173664	14533	Alcoholic beverage, distilled, all (gin, rum, vodka, whiskey) 100 proof
2658	Beverages, Alcoholic	174815	14037	Alcoholic beverage, distilled, all (gin, rum, vodka, whiskey) 80 proof
2680	Infant food	168968	33864	Infant Formula, MEAD JOHNSON, ENFAMIL, Premium LIPIL, Infant, Liquid concentrate, not reconstituted
2680	Infant food	168969	33865	Infant Formula, MEAD JOHNSON, ENFAMIL, Premium, Infant, Liquid concentrate, not reconstituted
2680	Infant food	168974	33874	Infant formula, MEAD JOHNSON, ENFAMIL, Premature, 20 calories ready-to-feed Low iron
2680	Infant food	169789	33875	Infant formula, MEAD JOHNSON, ENFAMIL, Premature, 24 calories ready-to-feed Low iron
2680	Infant food	169790	33876	Infant Formula, MEAD JOHNSON, ENFAMIL, Premium, Infant, ready-to-feed
2680	Infant food	170986	3955	Infant Formula, MEAD JOHNSON, ENFAMIL, ENFACARE, ready-to-feed, with ARA and DHA
2680	Infant food	170991	3966	Infant formula, NESTLE, GOOD START SOY, with DHA and ARA, liquid concentrate
2680	Infant food	170993	3968	Toddler formula, MEAD JOHNSON, ENFAGROW PREMIUM (formerly ENFAMIL, LIPIL, NEXT STEP), ready-to-feed
2680	Infant food	170994	3986	Infant Formula, MEAD JOHNSON, ENFAMIL, Newborn, with DHA and ARA, ready-to-feed
2680	Infant food	170995	3987	Infant formula, GERBER, GOOD START 2 Soy, with iron, ready-to-feed
2680	Infant food	170996	3988	Infant formula, GERBER, GOOD START, PROTECT PLUS, ready-to-feed
2680	Infant food	170997	3989	Infant Formula, GERBER GOOD START 2, GENTLE PLUS, ready-to-feed
2680	Infant food	171371	3216	Babyfood, GERBER, GRADUATES Lil Biscuits Vanilla Wheat
2680	Infant food	171382	3952	Infant formula, ABBOTT NUTRITION, SIMILAC, ISOMIL, ADVANCE with iron, liquid concentrate
2680	Infant food	171383	3953	Infant formula, ABBOTT NUTRITION, SIMILAC, ISOMIL, ADVANCE with iron, ready-to-feed
2680	Infant food	171385	3960	Infant formula, NESTLE, GOOD START SUPREME, with iron, DHA and ARA, ready-to-feed
2680	Infant food	171386	3961	Infant formula, NESTLE, GOOD START SUPREME, with iron, DHA and ARA, prepared from liquid concentrate
2680	Infant food	171390	3982	Infant formula, MEAD JOHNSON, ENFAMIL, Enfagrow, Soy, Toddler ready-to-feed
2680	Infant food	171391	3983	Infant formula, MEAD JOHNSON, ENFAMIL, NUTRAMIGEN AA, ready-to-feed
2680	Infant food	171392	3984	Infant formula, MEAD JOHNSON, ENFAMIL, Premature, with iron, 20 calories, ready-to-feed
2680	Infant food	171393	3985	Infant formula, MEAD JOHNSON, ENFAMIL, Premature, with iron, 24 calories, ready-to-feed
2680	Infant food	171394	3990	Infant formula, GERBER, GOOD START 2, PROTECT PLUS, ready-to-feed
2680	Infant food	171395	3991	Infant formula, ABBOTT NUTRITION, SIMILAC, GO AND GROW, ready-to-feed, with ARA and DHA
2680	Infant food	171396	3992	Infant formula, ABBOTT NUTRITION, SIMILAC, Expert Care, Diarrhea, ready- to- feed with ARA and DHA
2680	Infant food	171397	3993	Infant formula, ABBOTT NUTRITION, SIMILAC, For Spit Up, ready-to-feed, with ARA and DHA
2680	Infant food	172289	3382	Infant formula, MEAD JOHNSON, Enfamil Premature High Protein 24 Calories, ready to feed, with ARA and DHA
2680	Infant food	172290	3384	Infant formula, MEAD JOHNSON, Enfamil Premature 30 Calories, ready to feed, with ARA and DHA
2680	Infant food	172293	3391	Infant formula, MEAD JOHNSON, Pregestimil 24 Calories, ready to feed, with ARA and DHA
2680	Infant food	172295	3393	Infant formula, MEAD JOHNSON, Enfamil for Supplementing, ready to feed, with ARA and DHA
2680	Infant food	172303	3812	Infant formula, MEAD JOHNSON, ENFAMIL, Infant, with iron, liquid concentrate, with ARA and DHA, reconstituted
2680	Infant food	172304	3815	Infant formula, MEAD JOHNSON, ENFAMIL LIPIL, with iron, ready-to-feed, with ARA and DHA
2680	Infant food	172305	3825	Infant formula, MEAD JOHNSON, ENFAMIL, LIPIL, low iron, ready to feed, with ARA and DHA
2680	Infant food	172306	3832	Infant formula, MEAD JOHNSON, ENFAMIL, Infant, ready-to-feed, with ARA and DHA
2680	Infant food	172309	3845	Infant formula, MEAD JOHNSON, ENFAMIL, NUTRAMIGEN, with iron, ready-to-feed, with ARA and DHA
2680	Infant food	172310	3846	Infant formula, ABBOTT NUTRITION, SIMILAC, ALIMENTUM, with iron, ready-to-feed
2680	Infant food	172312	3851	Infant formula, ABBOTT NUTRITION, SIMILAC, with iron, liquid concentrate, not reconstituted
2680	Infant food	172313	3860	Child formula, ABBOTT NUTRITION, PEDIASURE, ready-to-feed
2680	Infant food	172315	3864	Infant formula, MEAD JOHNSON, NEXT STEP, PROSOBEE, LIPIL, ready to feed, with ARA and DHA
2680	Infant food	172318	3935	Infant formula, ABBOTT NUTRITION, SIMILAC, ALIMENTUM, ADVANCE, ready-to-feed, with ARA and DHA
2680	Infant food	172319	3936	Infant formula, PBM PRODUCTS, store brand, ready-to-feed
2680	Infant food	172320	3937	Infant formula, PBM PRODUCTS, store brand, liquid concentrate, not reconstituted
2680	Infant food	172321	3942	Infant formula, MEAD JOHNSON, ENFAMIL, AR, ready-to-feed, with ARA and DHA
2680	Infant food	172323	3944	Infant formula, ABBOTT NUTRITION, SIMILAC NEOSURE, ready-to-feed, with ARA and DHA
2680	Infant food	172325	3946	Infant formula, ABBOTT NUTRITION, SIMILAC, SENSITIVE (LACTOSE FREE) ready-to-feed, with ARA and DHA
2680	Infant food	172326	3951	Infant formula, ABBOTT NUTRITION, SIMILAC, ADVANCE, with iron, liquid concentrate, not reconstituted
2680	Infant food	173522	3376	Infant formula, MEAD JOHNSON, Enfamil 24, ready to feed, with ARA and DHA
2680	Infant food	173524	3387	Infant formula, MEAD JOHNSON, Enfamil Reguline, ready to feed, with ARA and DHA
2680	Infant food	173525	3388	Infant formula, MEAD JOHNSON, Gentlease, ready to feed, with ARA and DHA
2680	Infant food	173527	3390	Infant formula, MEAD JOHNSON, Pregestimil 20 Calories, ready to feed, with ARA and DHA
2680	Infant food	173535	3801	Infant formula, NESTLE, GOOD START SUPREME, with iron, liquid concentrate, not reconstituted
2680	Infant food	173537	3818	Infant formula, MEAD JOHNSON, ENFAMIL, LIPIL, low iron, liquid concentrate, with ARA and DHA
2680	Infant food	173540	3823	Infant formula, MEAD JOHNSON, PROSOBEE, with iron, ready-to-feed
2680	Infant food	173541	3841	Infant formula, ABBOTT NUTRITION, SIMILAC, ISOMIL, with iron, ready-to-feed
2680	Infant food	173542	3842	Infant formula, ABBOTT NUTRITION, SIMILAC, ISOMIL, with iron, liquid concentrate
2680	Infant food	173544	3844	Infant formula, MEAD JOHNSON, ENFAMIL, NUTRAMIGEN, with iron, liquid concentrate not reconstituted, with ARA and DHA
2680	Infant food	173547	3854	Infant formula, MEAD JOHNSON, ENFAMIL, PROSOBEE, liquid concentrate, reconstituted, with ARA and DHA
2680	Infant food	173548	3857	Infant formula, MEAD JOHNSON, PROSOBEE, with iron, ready to feed, with ARA and DHA
2680	Infant food	173549	3859	Infant formula, NESTLE, GOOD START SOY, with DHA and ARA, ready-to-feed
2680	Infant food	173550	3870	Child formula, ABBOTT NUTRITION, PEDIASURE, ready-to-feed, with iron and fiber
2680	Infant food	173555	3939	Infant formula, PBM PRODUCTS, store brand, soy, ready-to-feed
2680	Infant food	173556	3940	Infant formula, PBM PRODUCTS, store brand, soy, liquid concentrate, not reconstituted
2680	Infant food	173558	3947	Infant formula, ABBOTT NUTRITION, SIMILAC, SENSITIVE, (LACTOSE FREE), liquid concentrate, with ARA and DHA
2680	Infant food	173560	3949	Infant formula, ABBOTT NUTRITION, SIMILAC, ADVANCE, with iron, ready-to-feed
2731	Bovine Meat	169483	13795	Beef, composite of trimmed retail cuts, separable lean and fat, trimmed to 1/8in fat, all grades, raw
2731	Bovine Meat	173815	17088	Veal, composite of trimmed retail cuts, separable lean and fat, raw
2731	Bovine Meat	174757	23482	Beef, composite of trimmed retail cuts, separable lean and fat, trimmed to 0in fat, all grades, raw
2732	Mutton & Goat Meat	172479	17001	Lamb, composite of trimmed retail cuts, separable lean and fat, trimmed to 1/4in fat, choice, raw
2732	Mutton & Goat Meat	172545	17226	Lamb, composite of trimmed retail cuts, separable lean and fat, trimmed to 1/8in fat, choice, raw
2732	Mutton & Goat Meat	175303	17168	Game meat, goat, raw
2733	Pigmeat	167888	10187	Pork, fresh, composite of trimmed retail cuts (leg, loin, shoulder, and spareribs), separable lean and fat, raw
2734	Poultry Meat	171081	5165	Turkey, whole, meat and skin, raw
2734	Poultry Meat	171447	5006	Chicken, broilers or fryers, meat and skin, raw
2734	Poultry Meat	172408	5139	Duck, domesticated, meat and skin, raw
2734	Poultry Meat	172416	5151	Guinea hen, meat and skin, raw
2734	Poultry Meat	174470	5146	Goose, domesticated, meat and skin, raw
2735	Meat, Other	167607	35049	Moose, meat, raw (Alaska Native)
2735	Meat, Other	167610	35056	Seal, bearded (Oogruk), meat, raw (Alaska Native)
2735	Meat, Other	167622	35080	Deer (venison), sitka, raw (Alaska Native)
2735	Meat, Other	167745	93600	Turtle, green, raw
2735	Meat, Other	167888	10187	Pork, fresh, composite of trimmed retail cuts (leg, loin, shoulder, and spareribs), separable lean and fat, raw

Supplementary Table 3 (continued): NBS food composition matches

FBS_itemcode	FBS_itemname	fdc_id	ndb_id	fdc_name
2735	Meat, Other	168025	35071	Seal, ringed, meat (Alaska Native)
2735	Meat, Other	168029	35081	Walrus, meat, raw (Alaska Native)
2735	Meat, Other	168148	80200	Frog legs, raw
2735	Meat, Other	168987	35034	Fish, devilfish, meat (Alaska Native)
2735	Meat, Other	169003	35229	Sea lion, Steller, meat (Alaska Native)
2735	Meat, Other	169483	13795	Beef, composite of trimmed retail cuts, separable lean and fat, trimmed to 1/8in fat, all grades, raw
2735	Meat, Other	169793	35007	Bear, black, meat (Alaska Native)
2735	Meat, Other	169794	35008	Bear, polar, meat, raw (Alaska Native)
2735	Meat, Other	169797	35011	Whale, beluga, meat, raw (Alaska Native)
2735	Meat, Other	171081	5165	Turkey, whole, meat and skin, raw
2735	Meat, Other	171447	5006	Chicken, broilers or fryers, meat and skin, raw
2735	Meat, Other	172408	5139	Duck, domesticated, meat and skin, raw
2735	Meat, Other	172416	5151	Guinea hen, meat and skin, raw
2735	Meat, Other	172418	5157	Quail, meat and skin, raw
2735	Meat, Other	172479	17001	Lamb, composite of trimmed retail cuts, separable lean and fat, trimmed to 1/4in fat, choice, raw
2735	Meat, Other	172518	17174	Game meat, muskrat, raw
2735	Meat, Other	172521	17177	Game meat, rabbit, domesticated, composite of cuts, raw
2735	Meat, Other	172523	17183	Game meat, squirrel, raw
2735	Meat, Other	172545	17226	Lamb, composite of trimmed retail cuts, separable lean and fat, trimmed to 1/8in fat, choice, raw
2735	Meat, Other	172832	5621	Emu, ground, raw
2735	Meat, Other	173815	17088	Veal, composite of trimmed retail cuts, separable lean and fat, raw
2735	Meat, Other	173845	17146	Game meat, bear, raw
2735	Meat, Other	173851	17156	Game meat, bison, separable lean only, raw
2735	Meat, Other	173853	17162	Game meat, caribou, raw
2735	Meat, Other	173855	17164	Game meat, deer, raw
2735	Meat, Other	174344	17172	Game meat, moose, raw
2735	Meat, Other	174347	17180	Game meat, rabbit, wild, raw
2735	Meat, Other	174470	5146	Goose, domesticated, meat and skin, raw
2735	Meat, Other	174472	5153	Pheasant, raw, meat and skin
2735	Meat, Other	174475	5160	Squab, (pigeon), meat and skin, raw
2735	Meat, Other	174481	5641	Ostrich, ground, raw
2735	Meat, Other	174757	23482	Beef, composite of trimmed retail cuts, separable lean and fat, trimmed to 0in fat, all grades, raw
2735	Meat, Other	175086	17170	Game meat, horse, raw
2735	Meat, Other	175292	17144	Game meat, antelope, raw
2735	Meat, Other	175293	17149	Bison, ground, grass-fed, raw
2735	Meat, Other	175294	17150	Game meat, beaver, raw
2735	Meat, Other	175296	17152	Game meat, beefalo, composite of cuts, raw
2735	Meat, Other	175297	17158	Game meat, boar, wild, raw
2735	Meat, Other	175299	17160	Game meat, buffalo, water, raw
2735	Meat, Other	175301	17166	Game meat, elk, raw
2735	Meat, Other	175303	17168	Game meat, goat, raw
2736	Offals, Edible	167857	10100	Pork, fresh, variety meats and by-products, ears, frozen, raw
2736	Offals, Edible	167859	10102	Pork, fresh, variety meats and by-products, feet, raw
2736	Offals, Edible	167862	10110	Pork, fresh, variety meats and by-products, liver, raw
2736	Offals, Edible	167864	10112	Pork, fresh, variety meats and by-products, lungs, raw
2736	Offals, Edible	167865	10117	Pork, fresh, variety meats and by-products, spleen, raw
2736	Offals, Edible	167867	10119	Pork, fresh, variety meats and by-products, stomach, raw
2736	Offals, Edible	168026	35072	Seal, ringed, liver (Alaska Native)
2736	Offals, Edible	168031	35083	Walrus, liver, raw (Alaska Native)
2736	Offals, Edible	168264	10096	Pork, fresh, variety meats and by-products, brain, raw
2736	Offals, Edible	168266	10098	Pork, fresh, variety meats and by-products, chitterlings, raw
2736	Offals, Edible	168267	10103	Pork, fresh, variety meats and by-products, heart, raw
2736	Offals, Edible	168269	10105	Pork, fresh, variety meats and by-products, jowl, raw
2736	Offals, Edible	168270	10106	Pork, fresh, variety meats and by-products, kidneys, raw
2736	Offals, Edible	168273	10115	Pork, fresh, variety meats and by-products, pancreas, raw
2736	Offals, Edible	168275	10121	Pork, fresh, variety meats and by-products, tongue, raw
2736	Offals, Edible	168291	10174	Pork, fresh, variety meats and by-products, tail, raw
2736	Offals, Edible	168622	13318	Beef, variety meats and by-products, brain, raw
2736	Offals, Edible	168625	13321	Beef, variety meats and by-products, heart, raw
2736	Offals, Edible	168628	13328	Beef, variety meats and by-products, lungs, raw
2736	Offals, Edible	168980	35013	Whale, beluga, liver, raw (Alaska Native)
2736	Offals, Edible	168983	35023	Caribou, liver, raw (Alaska Native)
2736	Offals, Edible	168985	35025	Caribou, tongue, raw (Alaska Native)
2736	Offals, Edible	169000	35226	Sea lion, Steller, liver (Alaska Native)
2736	Offals, Edible	169002	35228	Sea lion, Steller, heart (Alaska Native)
2736	Offals, Edible	169449	13323	Beef, variety meats and by-products, kidneys, raw
2736	Offals, Edible	169451	13325	Beef, variety meats and by-products, liver, raw
2736	Offals, Edible	169452	13331	Beef, variety meats and by-products, pancreas, raw
2736	Offals, Edible	169454	13333	Beef, variety meats and by-products, spleen, raw
2736	Offals, Edible	170194	13337	Beef, variety meats and by-products, thymus, raw
2736	Offals, Edible	170196	13339	Beef, variety meats and by-products, tongue, raw
2736	Offals, Edible	170599	13341	Beef, variety meats and by-products, tripe, raw
2736	Offals, Edible	171060	5027	Chicken, liver, all classes, raw
2736	Offals, Edible	171458	5025	Chicken, heart, all classes, raw
2736	Offals, Edible	171484	5175	Turkey, all classes, heart, raw
2736	Offals, Edible	171486	5177	Turkey, all classes, liver, raw
2736	Offals, Edible	172415	5150	Goose, liver, raw
2736	Offals, Edible	172525	17185	Lamb, variety meats and by-products, brain, raw
2736	Offals, Edible	172527	17191	Lamb, variety meats and by-products, heart, raw
2736	Offals, Edible	172529	17193	Veal, variety meats and by-products, heart, raw
2736	Offals, Edible	172531	17199	Lamb, variety meats and by-products, liver, raw
2736	Offals, Edible	172534	17202	Veal, variety meats and by-products, liver, raw
2736	Offals, Edible	172538	17210	Lamb, variety meats and by-products, pancreas, raw
2736	Offals, Edible	172540	17216	Veal, variety meats and by-products, spleen, raw
2736	Offals, Edible	172542	17218	Veal, variety meats and by-products, thymus, raw
2736	Offals, Edible	172615	17369	Lamb, New Zealand, imported, liver, raw
2736	Offals, Edible	172621	17379	Lamb, New Zealand, imported, tongue - swiss cut, raw
2736	Offals, Edible	173096	23445	Beef, New Zealand, imported, variety meats and by-products, tripe uncooked, raw
2736	Offals, Edible	174351	17188	Veal, variety meats and by-products, brain, raw

Supplementary Table 3 (continued): NBS food composition matches

FBS_itemcode	FBS_itemname	fdc_id	ndb_id	fdc_name
2736	Offals, Edible	174354	17195	Lamb, variety meats and by-products, kidneys, raw
2736	Offals, Edible	174356	17197	Veal, variety meats and by-products, kidneys, raw
2736	Offals, Edible	174359	17205	Lamb, variety meats and by-products, lungs, raw
2736	Offals, Edible	174361	17207	Veal, variety meats and by-products, lungs, raw
2736	Offals, Edible	174362	17212	Veal, variety meats and by-products, pancreas, raw
2736	Offals, Edible	174364	17214	Lamb, variety meats and by-products, spleen, raw
2736	Offals, Edible	174366	17220	Lamb, variety meats and by-products, tongue, raw
2736	Offals, Edible	174368	17222	Veal, variety meats and by-products, tongue, raw
2736	Offals, Edible	174437	17358	Lamb, New Zealand, imported, brains, raw
2736	Offals, Edible	174442	17367	Lamb, New Zealand, imported, kidney, raw
2736	Offals, Edible	174444	17373	Lamb, New Zealand, imported, heart, raw
2736	Offals, Edible	174467	5143	Duck, domesticated, liver, raw
2736	Offals, Edible	174723	23415	Beef, New Zealand, imported, variety meats and by-products, heart, raw
2736	Offals, Edible	174727	23423	Beef, New Zealand, imported, variety meats and by-products, kidney, raw
2736	Offals, Edible	174729	23425	Beef, New Zealand, imported, variety meats and by-products, liver, raw
2736	Offals, Edible	174738	23443	Beef, New Zealand, imported, variety meats and by-products, tongue, raw
2737	Fats, Animals, Raw	167861	10109	Pork, fresh, variety meats and by-products, leaf fat, raw
2737	Fats, Animals, Raw	170193	13335	Beef, variety meats and by-products, suet, raw
2737	Fats, Animals, Raw	171400	4001	Fat, beef tallow
2737	Fats, Animals, Raw	171401	4002	Lard
2737	Fats, Animals, Raw	173564	4542	Fat, chicken
2737	Fats, Animals, Raw	173571	4575	Fat, turkey
2737	Fats, Animals, Raw	173572	4576	Fat, goose
2740	Butter, Ghee	173430	1145	Butter, without salt
2743	Cream	170859	1053	Cream, fluid, heavy whipping
2744	Eggs	171287	1123	Egg, whole, raw, fresh
2744	Eggs	172189	1138	Egg, duck, whole, fresh, raw
2744	Eggs	172190	1139	Egg, goose, whole, fresh, raw
2744	Eggs	172191	1140	Egg, quail, whole, fresh, raw
2744	Eggs	172192	1141	Egg, turkey, whole, fresh, raw
2745	Honey	169640	19296	Honey
2761	Freshwater Fish	167639	35150	Fish, salmon, coho (silver), raw (Alaska Native)
2761	Freshwater Fish	167648	35169	Fish, sheefish, raw (Alaska Native)
2761	Freshwater Fish	168033	35089	Fish, whitefish, mixed species, raw (Alaska Native)
2761	Freshwater Fish	168045	35151	Fish, salmon, sockeye (red), raw (Alaska Native)
2761	Freshwater Fish	168046	35152	Fish, Salmon, Chum, raw (Alaska Native)
2761	Freshwater Fish	168047	35153	Fish, salmon, king (chinook), raw (Alaska Native)
2761	Freshwater Fish	171948	15004	Fish, bass, striped, raw
2761	Freshwater Fish	171950	15006	Fish, burbot, raw
2761	Freshwater Fish	171952	15008	Fish, carp, raw
2761	Freshwater Fish	171953	15013	Fish, cisco, raw
2761	Freshwater Fish	171960	15024	Fish, drum, freshwater, raw
2761	Freshwater Fish	173675	15053	Fish, milkfish, raw
2761	Freshwater Fish	173678	15060	Fish, perch, mixed species, raw
2761	Freshwater Fish	173680	15062	Fish, pike, northern, raw
2761	Freshwater Fish	173686	15076	Fish, salmon, Atlantic, wild, raw
2761	Freshwater Fish	173688	15078	Fish, salmon, chinook, raw
2761	Freshwater Fish	173691	15085	Fish, salmon, sockeye, raw
2761	Freshwater Fish	173695	15093	Fish, seatrout, mixed species, raw
2761	Freshwater Fish	173696	15094	Fish, shad, american, raw
2761	Freshwater Fish	173701	15104	Fish, sturgeon, mixed species, raw
2761	Freshwater Fish	173711	15130	Fish, whitefish, mixed species, raw
2761	Freshwater Fish	173715	15238	Fish, salmon, coho, farmed, raw
2761	Freshwater Fish	173717	15240	Fish, trout, rainbow, farmed, raw
2761	Freshwater Fish	174184	15003	Fish, bass, fresh water, mixed species, raw
2761	Freshwater Fish	174186	15010	Fish, catfish, channel, wild, raw
2761	Freshwater Fish	174193	15025	Fish, eel, mixed species, raw
2761	Freshwater Fish	175128	15064	Fish, pike, walleye, raw
2761	Freshwater Fish	175136	15081	Fish, salmon, coho, wild, raw
2761	Freshwater Fish	175138	15083	Fish, salmon, pink, raw
2761	Freshwater Fish	175146	15099	Fish, smelt, rainbow, raw
2761	Freshwater Fish	175150	15107	Fish, sucker, white, raw
2761	Freshwater Fish	175153	15114	Fish, trout, mixed species, raw
2761	Freshwater Fish	175154	15115	Fish, trout, rainbow, wild, raw
2761	Freshwater Fish	175165	15234	Fish, catfish, channel, farmed, raw
2761	Freshwater Fish	175167	15236	Fish, salmon, Atlantic, farmed, raw
2761	Freshwater Fish	175176	15261	Fish, tilapia, raw
2761	Freshwater Fish	175181	15274	Fish, trout, brook, raw, New York State
2762	Demersal Fish	167638	35149	Fish, halibut, raw, with skin (Alaska Native)
2762	Demersal Fish	169809	35046	Fish, lingcod, meat, raw (Alaska Native)
2762	Demersal Fish	171955	15015	Fish, cod, Atlantic, raw
2762	Demersal Fish	171958	15022	Fish, cusk, raw
2762	Demersal Fish	171962	15031	Fish, grouper, mixed species, raw
2762	Demersal Fish	171964	15033	Fish, haddock, raw
2762	Demersal Fish	171965	15038	Fish, halibut, Greenland, raw
2762	Demersal Fish	173670	15044	Fish, ling, raw
2762	Demersal Fish	173671	15045	Fish, lingcod, raw
2762	Demersal Fish	173676	15054	Fish, monkfish, raw
2762	Demersal Fish	173677	15059	Fish, pout, ocean, raw
2762	Demersal Fish	173684	15070	Fish, rockfish, Pacific, mixed species, raw
2762	Demersal Fish	173697	15095	Fish, shark, mixed species, raw
2762	Demersal Fish	173698	15101	Fish, snapper, mixed species, raw
2762	Demersal Fish	173700	15103	Fish, spot, raw
2762	Demersal Fish	173705	15112	Fish, tilefish, raw
2762	Demersal Fish	173710	15129	Fish, turbot, european, raw
2762	Demersal Fish	173713	15132	Fish, whiting, mixed species, raw
2762	Demersal Fish	173725	15266	Fish, pollock, Alaska, raw
2762	Demersal Fish	174191	15019	Fish, cod, Pacific, raw (may have been previously frozen)
2762	Demersal Fish	174192	15020	Fish, croaker, Atlantic, raw

Supplementary Table 3 (continued): NBS food composition matches

FBS_itemcode	FBS_itemname	fdc_id	ndb_id	fdc_name
2762	Demersal Fish	174196	15028	Fish, flatfish (flounder and sole species), raw
2762	Demersal Fish	174200	15036	Fish, halibut, Atlantic and Pacific, raw
2762	Demersal Fish	175123	15055	Fish, mullet, striped, raw
2762	Demersal Fish	175125	15057	Fish, ocean perch, Atlantic, raw
2762	Demersal Fish	175129	15065	Fish, pollock, Atlantic, raw
2762	Demersal Fish	175130	15066	Fish, pollock, Alaska, raw (may contain additives to retain moisture)
2762	Demersal Fish	175133	15073	Fish, roughy, orange, raw
2762	Demersal Fish	175134	15074	Fish, sablefish, raw
2762	Demersal Fish	175141	15090	Fish, scup, raw
2762	Demersal Fish	175142	15091	Fish, sea bass, mixed species, raw
2762	Demersal Fish	175144	15097	Fish, sheepshead, raw
2762	Demersal Fish	175162	15134	Fish, wolffish, Atlantic, raw
2762	Demersal Fish	175163	15135	Fish, yellowtail, mixed species, raw
2762	Demersal Fish	333476	15066	Fish, pollock, raw
2763	Pelagic Fish	171949	15005	Fish, bluefish, raw
2763	Pelagic Fish	171951	15007	Fish, butterfish, raw
2763	Pelagic Fish	171959	15023	Fish, mahimahi, raw
2763	Pelagic Fish	173669	15043	Fish, herring, Pacific, raw
2763	Pelagic Fish	173672	15050	Fish, mackerel, Pacific and jack, mixed species, raw
2763	Pelagic Fish	173673	15051	Fish, mackerel, spanish, raw
2763	Pelagic Fish	173682	15068	Fish, pompano, florida, raw
2763	Pelagic Fish	173703	15110	Fish, swordfish, raw
2763	Pelagic Fish	173706	15117	Fish, tuna, fresh, bluefin, raw
2763	Pelagic Fish	174182	15001	Fish, anchovy, european, raw
2763	Pelagic Fish	175116	15039	Fish, herring, Atlantic, raw
2763	Pelagic Fish	175119	15046	Fish, mackerel, Atlantic, raw
2763	Pelagic Fish	175122	15049	Fish, mackerel, king, raw
2763	Pelagic Fish	175151	15108	Fish, sunfish, pumpkin seed, raw
2763	Pelagic Fish	175156	15123	Fish, tuna, fresh, skipjack, raw
2763	Pelagic Fish	175159	15127	Fish, tuna, fresh, yellowfin, raw
2764	Marine Fish, Other	167638	35149	Fish, halibut, raw, with skin (Alaska Native)
2764	Marine Fish, Other	167639	35150	Fish, salmon, coho (silver), raw (Alaska Native)
2764	Marine Fish, Other	167648	35169	Fish, sheefish, raw (Alaska Native)
2764	Marine Fish, Other	168033	35089	Fish, whitefish, mixed species, raw (Alaska Native)
2764	Marine Fish, Other	168045	35151	Fish, salmon, sockeye (red), raw (Alaska Native)
2764	Marine Fish, Other	168046	35152	Fish, Salmon, Chum, raw (Alaska Native)
2764	Marine Fish, Other	168047	35153	Fish, salmon, king (chinook), raw (Alaska Native)
2764	Marine Fish, Other	169809	35046	Fish, lingcod, meat, raw (Alaska Native)
2764	Marine Fish, Other	171948	15004	Fish, bass, striped, raw
2764	Marine Fish, Other	171949	15005	Fish, bluefish, raw
2764	Marine Fish, Other	171950	15006	Fish, burbot, raw
2764	Marine Fish, Other	171951	15007	Fish, butterfish, raw
2764	Marine Fish, Other	171952	15008	Fish, carp, raw
2764	Marine Fish, Other	171953	15013	Fish, cisco, raw
2764	Marine Fish, Other	171955	15015	Fish, cod, Atlantic, raw
2764	Marine Fish, Other	171958	15022	Fish, cusk, raw
2764	Marine Fish, Other	171959	15023	Fish, mahimahi, raw
2764	Marine Fish, Other	171960	15024	Fish, drum, freshwater, raw
2764	Marine Fish, Other	171962	15031	Fish, grouper, mixed species, raw
2764	Marine Fish, Other	171964	15033	Fish, haddock, raw
2764	Marine Fish, Other	171965	15038	Fish, halibut, Greenland, raw
2764	Marine Fish, Other	173669	15043	Fish, herring, Pacific, raw
2764	Marine Fish, Other	173670	15044	Fish, ling, raw
2764	Marine Fish, Other	173671	15045	Fish, lingcod, raw
2764	Marine Fish, Other	173672	15050	Fish, mackerel, Pacific and jack, mixed species, raw
2764	Marine Fish, Other	173673	15051	Fish, mackerel, spanish, raw
2764	Marine Fish, Other	173675	15053	Fish, milkfish, raw
2764	Marine Fish, Other	173676	15054	Fish, monkfish, raw
2764	Marine Fish, Other	173677	15059	Fish, pout, ocean, raw
2764	Marine Fish, Other	173678	15060	Fish, perch, mixed species, raw
2764	Marine Fish, Other	173680	15062	Fish, pike, northern, raw
2764	Marine Fish, Other	173682	15068	Fish, pompano, florida, raw
2764	Marine Fish, Other	173684	15070	Fish, rockfish, Pacific, mixed species, raw
2764	Marine Fish, Other	173686	15076	Fish, salmon, Atlantic, wild, raw
2764	Marine Fish, Other	173688	15078	Fish, salmon, chinook, raw
2764	Marine Fish, Other	173691	15085	Fish, salmon, sockeye, raw
2764	Marine Fish, Other	173695	15093	Fish, seatrout, mixed species, raw
2764	Marine Fish, Other	173696	15094	Fish, shad, american, raw
2764	Marine Fish, Other	173697	15095	Fish, shark, mixed species, raw
2764	Marine Fish, Other	173698	15101	Fish, snapper, mixed species, raw
2764	Marine Fish, Other	173700	15103	Fish, spot, raw
2764	Marine Fish, Other	173701	15104	Fish, sturgeon, mixed species, raw
2764	Marine Fish, Other	173703	15110	Fish, swordfish, raw
2764	Marine Fish, Other	173705	15112	Fish, tilefish, raw
2764	Marine Fish, Other	173706	15117	Fish, tuna, fresh, bluefin, raw
2764	Marine Fish, Other	173710	15129	Fish, turbot, european, raw
2764	Marine Fish, Other	173711	15130	Fish, whitefish, mixed species, raw
2764	Marine Fish, Other	173713	15132	Fish, whiting, mixed species, raw
2764	Marine Fish, Other	173715	15238	Fish, salmon, coho, farmed, raw
2764	Marine Fish, Other	173717	15240	Fish, trout, rainbow, farmed, raw
2764	Marine Fish, Other	173725	15266	Fish, pollock, Alaska, raw
2764	Marine Fish, Other	174182	15001	Fish, anchovy, european, raw
2764	Marine Fish, Other	174184	15003	Fish, bass, fresh water, mixed species, raw
2764	Marine Fish, Other	174186	15010	Fish, catfish, channel, wild, raw
2764	Marine Fish, Other	174191	15019	Fish, cod, Pacific, raw (may have been previously frozen)
2764	Marine Fish, Other	174192	15020	Fish, croaker, Atlantic, raw
2764	Marine Fish, Other	174193	15025	Fish, eel, mixed species, raw
2764	Marine Fish, Other	174196	15028	Fish, flatfish (flounder and sole species), raw
2764	Marine Fish, Other	174200	15036	Fish, halibut, Atlantic and Pacific, raw

Supplementary Table 3 (continued): NBS food composition matches

FBS_itemcode	FBS_itemname	fdc_id	ndb_id	fdc_name
2764	Marine Fish, Other	175116	15039	Fish, herring, Atlantic, raw
2764	Marine Fish, Other	175119	15046	Fish, mackerel, Atlantic, raw
2764	Marine Fish, Other	175122	15049	Fish, mackerel, king, raw
2764	Marine Fish, Other	175123	15055	Fish, mullet, striped, raw
2764	Marine Fish, Other	175125	15057	Fish, ocean perch, Atlantic, raw
2764	Marine Fish, Other	175128	15064	Fish, pike, walleye, raw
2764	Marine Fish, Other	175129	15065	Fish, pollock, Atlantic, raw
2764	Marine Fish, Other	175130	15066	Fish, pollock, Alaska, raw (may contain additives to retain moisture)
2764	Marine Fish, Other	175133	15073	Fish, roughy, orange, raw
2764	Marine Fish, Other	175134	15074	Fish, sablefish, raw
2764	Marine Fish, Other	175136	15081	Fish, salmon, coho, wild, raw
2764	Marine Fish, Other	175138	15083	Fish, salmon, pink, raw
2764	Marine Fish, Other	175141	15090	Fish, scup, raw
2764	Marine Fish, Other	175142	15091	Fish, sea bass, mixed species, raw
2764	Marine Fish, Other	175144	15097	Fish, sheepshead, raw
2764	Marine Fish, Other	175146	15099	Fish, smelt, rainbow, raw
2764	Marine Fish, Other	175150	15107	Fish, sucker, white, raw
2764	Marine Fish, Other	175151	15108	Fish, sunfish, pumpkin seed, raw
2764	Marine Fish, Other	175153	15114	Fish, trout, mixed species, raw
2764	Marine Fish, Other	175154	15115	Fish, trout, rainbow, wild, raw
2764	Marine Fish, Other	175156	15123	Fish, tuna, fresh, skipjack, raw
2764	Marine Fish, Other	175159	15127	Fish, tuna, fresh, yellowfin, raw
2764	Marine Fish, Other	175162	15134	Fish, wolffish, Atlantic, raw
2764	Marine Fish, Other	175163	15135	Fish, yellowtail, mixed species, raw
2764	Marine Fish, Other	175165	15234	Fish, catfish, channel, farmed, raw
2764	Marine Fish, Other	175167	15236	Fish, salmon, Atlantic, farmed, raw
2764	Marine Fish, Other	175176	15261	Fish, tilapia, raw
2764	Marine Fish, Other	175181	15274	Fish, trout, brook, raw, New York State
2764	Marine Fish, Other	333476	15066	Fish, pollock, raw
2765	Crustaceans	171968	15143	Crustaceans, crab, dungeness, raw
2765	Crustaceans	171969	15144	Crustaceans, crab, queen, raw
2765	Crustaceans	174204	15139	Crustaceans, crab, blue, raw
2765	Crustaceans	174206	15145	Crustaceans, crayfish, mixed species, wild, raw
2765	Crustaceans	174208	15147	Crustaceans, lobster, northern, raw
2765	Crustaceans	174211	15154	Crustaceans, spiny lobster, mixed species, raw
2765	Crustaceans	175164	15136	Crustaceans, crab, alaska king, raw
2765	Crustaceans	175169	15242	Crustaceans, crayfish, mixed species, farmed, raw
2765	Crustaceans	175179	15270	Crustaceans, shrimp, raw
2766	Cephalopods	168019	35054	Octopus (Alaska Native)
2766	Cephalopods	174215	15163	Mollusks, cuttlefish, mixed species, raw
2766	Cephalopods	174218	15166	Mollusks, octopus, common, raw
2766	Cephalopods	174223	15175	Mollusks, squid, mixed species, raw
2767	Molluscs, Other	167744	90560	Mollusks, snail, raw
2767	Molluscs, Other	169803	35028	Cockles, raw (Alaska Native)
2767	Molluscs, Other	171978	15167	Mollusks, oyster, eastern, wild, raw
2767	Molluscs, Other	171983	15177	Mollusks, whelk, unspecified, raw
2767	Molluscs, Other	174212	15155	Mollusks, abalone, mixed species, raw
2767	Molluscs, Other	174214	15157	Mollusks, clam, mixed species, raw
2767	Molluscs, Other	174216	15164	Mollusks, mussel, blue, raw
2767	Molluscs, Other	174219	15171	Mollusks, oyster, Pacific, raw
2767	Molluscs, Other	174220	15172	Mollusks, scallop, mixed species, raw
2767	Molluscs, Other	175172	15245	Mollusks, oyster, eastern, farmed, raw
2768	Meat, Aquatic Mammals	167610	35056	Seal, bearded (Oogruk), meat, raw (Alaska Native)
2768	Meat, Aquatic Mammals	168025	35071	Seal, ringed, meat (Alaska Native)
2768	Meat, Aquatic Mammals	168029	35081	Walrus, meat, raw (Alaska Native)
2768	Meat, Aquatic Mammals	168987	35034	Fish, devilfish, meat (Alaska Native)
2768	Meat, Aquatic Mammals	169003	35229	Sea lion, Steller, meat (Alaska Native)
2768	Meat, Aquatic Mammals	169797	35011	Whale, beluga, meat, raw (Alaska Native)
2769	Aquatic Animals, Others	167612	35058	Oopah (tunicate), whole animal (Alaska Native)
2769	Aquatic Animals, Others	167618	35070	Sea cucumber, yane (Alaska Native)
2769	Aquatic Animals, Others	167745	93600	Turtle, green, raw
2769	Aquatic Animals, Others	168148	80200	Frog legs, raw
2769	Aquatic Animals, Others	168978	35004	Ascidians (tunughnak) (Alaska Native)
2775	Aquatic Plants	168456	11444	Seaweed, irishmoss, raw
2775	Aquatic Plants	168457	11445	Seaweed, kelp, raw
2775	Aquatic Plants	169250	11254	Lotus root, raw
2775	Aquatic Plants	169280	11442	Seaweed, agar, raw
2775	Aquatic Plants	169301	11503	Water convolvulus,raw
2775	Aquatic Plants	170091	11666	Seaweed, spirulina, raw
2775	Aquatic Plants	170496	11669	Seaweed, wakame, raw
2781	Fish, Body Oil	172340	4590	Fish oil, herring
2781	Fish, Body Oil	172341	4591	Fish oil, menhaden
2781	Fish, Body Oil	172343	4593	Fish oil, salmon
2781	Fish, Liver Oil	173577	4589	Fish oil, cod liver
2781	Fish, Body Oil	173578	4594	Fish oil, sardine
2805	Rice (Milled Equivalent)	168876	20042	Rice, brown, parboiled, dry, Uncle Ben's
2805	Rice (Milled Equivalent)	168883	20054	Rice, white, glutinous, unenriched, uncooked
2805	Rice (Milled Equivalent)	168931	20452	Rice, white, short-grain, raw, unenriched
2805	Rice (Milled Equivalent)	169703	20036	Rice, brown, long-grain, raw (Includes foods for USDA's Food Distribution Program)
2805	Rice (Milled Equivalent)	169706	20040	Rice, brown, medium-grain, raw (Includes foods for USDA's Food Distribution Program)
2805	Rice (Milled Equivalent)	169756	20444	Rice, white, long-grain, regular, raw, unenriched
2805	Rice (Milled Equivalent)	169758	20446	Rice, white, long-grain, parboiled, unenriched, dry
2805	Rice (Milled Equivalent)	169760	20450	Rice, white, medium-grain, raw, unenriched
2848	Milk - Excluding Butter	170882	1109	Milk, sheep, fluid
2848	Milk - Excluding Butter	171280	1108	Milk, indian buffalo, fluid
2848	Milk - Excluding Butter	172217	1211	Milk, whole, 3.25% milkfat, without added vitamin A and vitamin D

Supplementary Table 3 (continued): NBS food composition matches

FBS_itemcode	FBS_itemname	fdc_id	ndb_id	fdc_name
**Flour**				
Whole				
2511	Wheat and products	168944	20649	Wheat flour, whole-grain, soft wheat
Refined				
2511	Wheat and products	168913	20129	Wheat flours, bread, unenriched
2511	Wheat and products	168940	20635	Wheat flour, white (industrial), 11.5% protein, bleached, unenriched
2511	Wheat and products	169761	20481	Wheat flour, white, all-purpose, unenriched
2511	Wheat and products	172017	20624	Wheat flour, white (industrial), 9% protein, bleached, unenriched
2511	Wheat and products	172019	20629	Wheat flour, white (industrial), 10% protein, bleached, unenriched
2511	Wheat and products	172020	20641	Wheat flour, white (industrial), 13% protein, bleached, unenriched
2511	Wheat and products	172022	20646	Wheat flour, white (industrial), 15% protein, bleached, unenriched
Whole/refined				
2514	Maize and products	168929	20422	Cornmeal, degermed, unenriched, yellow
2514	Maize and products	172015	20522	Cornmeal, degermed, unenriched, white
Masa				
2514	Maize and products	169696	20019	Corn flour, masa, unenriched, white
Whole/refined				
2517	Millet and products	169702	20031	Millet, raw
2517	Millet and products	169702	20031	millet, refined
Whole/refined				
2518	Sorghum and products	168943	20648	Sorghum flour, whole-grain
2518	Sorghum and products	173262	20650	Sorghum flour, refined, unenriched

Supplementary Table 4: NBS food matches for phytate Food composition data come from PhyFoodComp1.0 available at the International Network of Food Data Systems (INFOODS) (https://www.fao.org/infoods/infoods/tables-and-databases/faoinfoods-databases/en/). Matches were based on definitions for FAO Food Balance Sheet (FBS) categories, using food items in primary commodity form or processed and cooked as specified in the FDC item name.

FBS_itemcode	FBS_itemname	PhyFoodComp1.0 food_item_id	food_name_english
2511	Wheat and products	1030220	Wheat, Bobwhite, MON 71800 (glyphosate tolerant), raw
2511	Wheat and products	1030221	Wheat, Bobwhite, MON 71800 (glyphosate tolerant), raw
2511	Wheat and products	1030222	Wheat, Bobwhite, raw
2511	Wheat and products	1030223	Wheat, Bobwhite, raw
2511	Wheat and products	1030224	Wheat, C-306, grown organic, raw
2511	Wheat and products	1030227	Wheat, grown non-organic, raw
2511	Wheat and products	1030228	Wheat, raw
2511	Wheat and products	1030230	Wheat, raw
2511	Wheat and products	1030234	Wheat, whole grain, raw
2511	Wheat and products	1030235	Wheat, whole grain, raw
2511	Wheat and products	1030237	Wheat, with gluten, raw
2513	Barley and products	1060036	Barley, dried
2513	Barley and products	1060038	Barley, grain, raw
2513	Barley and products	1060045	Barley, raw
2513	Barley and products	1060046	Barley, Sunnita, raw, 25 kGy irradiated
2513	Barley and products	1060050	Barley, Sunnita, raw
2513	Barley and products	1060053	Barley, whole grain, dried
2514	Maize and products	1020022	Corn, 113, raw
2514	Maize and products	1020023	Corn, 12, raw
2514	Maize and products	1020024	Corn, 14, raw
2514	Maize and products	1020025	Corn, 22, raw
2514	Maize and products	1020026	Corn, 35, raw
2514	Maize and products	1020027	Corn, 36, raw
2514	Maize and products	1020028	Corn, 37, raw
2514	Maize and products	1020029	Corn, 41, raw
2514	Maize and products	1020030	Corn, 42, raw
2514	Maize and products	1020031	Corn, 43, raw
2514	Maize and products	1020032	Corn, 91, raw
2514	Maize and products	1020037	Corn, LH82xB73, raw
2514	Maize and products	1020038	Corn, LH82xB73, raw
2514	Maize and products	1020041	Corn, Mugtama 45, raw
2514	Maize and products	1020042	Corn, NK603xB73 (glyphosate tolerant), raw
2514	Maize and products	1020043	Corn, NK603xB73 (glyphosate tolerant), raw
2514	Maize and products	1020045	Corn, raw
2514	Maize and products	1020048	Corn, Texas 17W, raw
2514	Maize and products	1020065	Maize, 1F5924, dried
2514	Maize and products	1020066	Maize, 2E4794, dried
2514	Maize and products	1020067	Maize, 2E5305, dried
2514	Maize and products	1020068	Maize, 3E4824, dried
2514	Maize and products	1020073	Maize, Bc 183 (1985), full grain maturity, raw
2514	Maize and products	1020074	Maize, Bc 183 (1985), full grain maturity, raw
2514	Maize and products	1020080	Maize, Bc 183 (1986), full grain maturity, raw
2514	Maize and products	1020081	Maize, Bc 183 (1986), full grain maturity, raw
2514	Maize and products	1020087	Maize, Bc 196 (1985), full grain maturity, raw
2514	Maize and products	1020088	Maize, Bc 196 (1985), full grain maturity, raw
2514	Maize and products	1020094	Maize, Bc 196 (1986), full grain maturity, raw
2514	Maize and products	1020095	Maize, Bc 196 (1986), full grain maturity, raw
2514	Maize and products	1020101	Maize, Bc 488 (1985), full grain maturity, raw
2514	Maize and products	1020102	Maize, Bc 488 (1985), full grain maturity, raw
2514	Maize and products	1020108	Maize, Bc 488 (1986), full grain maturity, raw
2514	Maize and products	1020109	Maize, Bc 488 (1986), full grain maturity, raw
2514	Maize and products	1020117	Maize, dried
2514	Maize and products	1020118	Maize, grain, raw
2514	Maize and products	1020119	Maize, grain, raw
2514	Maize and products	1020126	Maize, T1A1 (hybrid), transgenic, negative trait expression (NK603: herbicide tolerance), raw
2514	Maize and products	1020127	Maize, T1A1 (hybrid), transgenic, positive trait expression (NK603: herbicide tolerance), raw
2514	Maize and products	1020128	Maize, T1A2 (hybrid), transgenic, negative trait expression (NK603: herbicide tolerance), raw
2514	Maize and products	1020129	Maize, T1A2 (hybrid), transgenic, positive trait expression (NK603: herbicide tolerance), raw
2514	Maize and products	1020130	Maize, T1B1 (hybrid), transgenic, negative trait expression (NK603: herbicide tolerance), raw
2514	Maize and products	1020131	Maize, T1B1 (hybrid), transgenic, positive trait expression (NK603: herbicide tolerance), raw
2514	Maize and products	1020132	Maize, T1B2 (hybrid), transgenic, negative trait expression (NK603: herbicide tolerance), raw
2514	Maize and products	1020133	Maize, T1B2 (hybrid), transgenic, positive trait expression (NK603: herbicide tolerance), raw
2514	Maize and products	1020134	Maize, tender, raw
2514	Maize and products	1020135	Maize, tender, sweet, raw
2514	Maize and products	1020137	Maize, white, whole dried kernels
2514	Maize and products	1020138	Maize, white, whole grain, dried
2514	Maize and products	1020139	Maize, white, whole grain, fresh, raw
2514	Maize and products	1020140	Maize, white, whole grain, raw
2515	Rye and products	1060226	Rye, grain, raw
2516	Oats	1060192	Oats, raw
2517	Millet and products	1050075	Bajra, raw
2517	Millet and products	1050112	Millet, bulrush, grain, raw
2517	Millet and products	1050116	Millet, raw
2517	Millet and products	1050117	Millet, raw
2517	Millet and products	1050121	Millet, whole grain, raw
2517	Millet and products	1050183	Pearl millet, B 1, yellowish, raw
2517	Millet and products	1050184	Pearl millet, B 2, gray, raw
2517	Millet and products	1050185	Pearl millet, Gampela, whole, raw
2517	Millet and products	1050186	Pearl millet, Gampela, yellow, raw, hand-decorticated
2517	Millet and products	1050187	Pearl millet, Gampela, yellow, raw, mechanically-decorticated
2517	Millet and products	1050188	Pearl millet, Gampela, yellow, raw
2517	Millet and products	1050189	Pearl millet, IKMP 1, yellowish, raw
2517	Millet and products	1050190	Pearl millet, IKMP 2, gray yellow, raw
2517	Millet and products	1050191	Pearl millet, IKMP 3, gray, raw
2517	Millet and products	1050192	Pearl millet, IKMP 5, gray light, raw
2517	Millet and products	1050193	Pearl millet, IKMP-5, whole, raw
2517	Millet and products	1050194	Pearl millet, IKMV 8201, gray yellow, raw
2517	Millet and products	1050195	Pearl millet, KM, gray, raw

Supplementary Table 4 (continued): NBS food matches for phytate

FBS_itemcode	FBS_itemname	PhyFoodComp1.0 food_item_id	food_name_english
2517	Millet and products	1050196	Pearl millet, L. Nahartenga, yellow blade, raw
2517	Millet and products	1050197	Pearl millet, L. Zatiib, gray yellow, raw
2517	Millet and products	1050200	Pearl millet, raw
2517	Millet and products	1050201	Pearl millet, raw
2517	Millet and products	1050202	Pearl millet, SG, gray, raw
2517	Millet and products	1050203	Pearl millet, SOSATC 88, gray yellow, raw
2517	Millet and products	1050204	Pearl millet, TK, yellow, raw
2517	Millet and products	1050206	Pearl millet, XX, gray, raw
2517	Millet and products	1050210	Proso millet, TNAU-145, brown
2517	Millet and products	1050211	Proso millet, TNAU-145, polished
2517	Millet and products	1050212	Proso millet, TNAU-145, whole
2517	Millet and products	1050213	Ragi, raw
2517	Millet and products	1050214	Samai, raw
2517	Millet and products	1050215	Varagu, raw
2518	Sorghum and products	1040007	Jowar, raw
2518	Sorghum and products	1040065	Sorghum, Fibmigou, white, raw, hand-decorticated
2518	Sorghum and products	1040066	Sorghum, Fibmigou, white, raw
2518	Sorghum and products	1040067	Sorghum, grain, white, raw
2518	Sorghum and products	1040068	Sorghum, raw
2518	Sorghum and products	1040069	Sorghum, red, whole grain, dried
2518	Sorghum and products	1040070	Sorghum, white, steamed
2518	Sorghum and products	1040071	Sorghum, white, steamed, dried
2518	Sorghum and products	1040072	Sorghum, white, whole grain, dried
2518	Sorghum and products	1040073	Sorghum, whole grain, red, raw
2518	Sorghum and products	1040074	Sorghum, whole grain, white, raw
2520	Cereals, Other	1030133	Triticale, grain, raw
2520	Cereals, Other	1050077	Fonio, Dieni, ready-to-cook, raw, dehulled
2520	Cereals, Other	1050078	Fonio, Finiba, ready-to-cook, raw, dehulled
2520	Cereals, Other	1050079	Fonio, Finiba/Kassangara, ready-to-cook, raw, dehulled
2520	Cereals, Other	1050080	Fonio, Kassangara, ready-to-cook, raw, dehulled
2520	Cereals, Other	1050081	Fonio, Peazo, ready-to-cook, raw, dehulled
2520	Cereals, Other	1050082	Fonio, Peazo, ready-to-cook, raw, dehulled
2520	Cereals, Other	1050083	Fonio, Petama, ready-to-cook, raw, dehulled
2520	Cereals, Other	1050084	Fonio, Peye, ready-to-cook, raw, dehulled
2520	Cereals, Other	1050085	Fonio, Tama, ready-to-cook, raw, dehulled
2520	Cereals, Other	1050086	Fonio, Tamabe, ready-to-cook,raw, dehulled
2520	Cereals, Other	1050087	Fonio, Tamatioi, ready-to-cook, raw, dehulled
2520	Cereals, Other	1050088	Fonio, Tioi, ready-to-cook, raw, dehulled
2520	Cereals, Other	1060002	Amaranth, brown, grain, dried
2520	Cereals, Other	1060004	Amaranth, Centenario, grain, raw
2520	Cereals, Other	1060006	Amaranth, Oscar Blanco, grain, raw
2520	Cereals, Other	1060007	Amaranth, red, grain, dried
2520	Cereals, Other	1060008	Amaranth, white, grain, dried
2520	Cereals, Other	1060112	Buckwheat, broken whole grain, dried
2520	Cereals, Other	1060116	Buckwheat, Kora, raw
2520	Cereals, Other	1060117	Buckwheat, whole grain, dried
2520	Cereals, Other	1060222	Quinoa, raw
2520	Cereals, Other	1060230	Teff, mixed red and white, whole grain, raw
2520	Cereals, Other	1060231	Teff, red, whole grain, raw
2520	Cereals, Other	1060232	Teff, white, whole grain, raw
2520	Cereals, Other	1060233	Teff, whole grain, dried
2520	Cereals, Other	4010007	Amaranth globe, ABS-38-AWKA, raw
2520	Cereals, Other	4010009	Amaranth globe, AKS-33-EKPENE EDIENE x ABS-38-AWKA, raw
2520	Cereals, Other	4010011	Amaranth globe, AKS-33-EKPENE EDIENE, raw
2520	Cereals, Other	4010013	Amaranth globe, EBS-15-NKALAGU x ABS-38-AKWA, raw
2520	Cereals, Other	4010015	Amaranth globe, EBS-15-NKALAGU x AKS-33-EKPENE EDIENE, raw
2520	Cereals, Other	4010017	Amaranth globe, EBS-15-NKALAGU x IMS-20-NJIABA, raw
2520	Cereals, Other	4010019	Amaranth globe, EBS-15-NKALAGU, raw
2520	Cereals, Other	4010021	Amaranth globe, ENS-08-MBU x ABS-38-AWKA, raw
2520	Cereals, Other	4010023	Amaranth globe, ENS-08-MBU x AKS-33-EKPENE EDIENE, raw
2520	Cereals, Other	4010025	Amaranth globe, ENS-08-MBU x EBS-15-NKALAGU, raw
2520	Cereals, Other	4010027	Amaranth globe, ENS-08-MBU x IMS-20-NJIABA, raw
2520	Cereals, Other	4010029	Amaranth globe, ENS-08-MBU, raw
2520	Cereals, Other	4010031	Amaranth globe, IMS-20-NJIABA x ABS-38-AWKA, raw
2520	Cereals, Other	4010033	Amaranth globe, IMS-20-NJIABA x AKS-33-EKPENE EDIENE, raw
2520	Cereals, Other	4010035	Amaranth globe, IMS-20-NJIABA, raw
2531	Potatoes and products	2010003	Potato, baby red skin, raw
2531	Potatoes and products	2010005	Potato, brown skin, big, raw
2531	Potatoes and products	2010006	Potato, brown skin, small, raw
2531	Potatoes and products	2010015	Potato, Irish potato, raw
2531	Potatoes and products	2010016	Potato, Kennebec, raw, unpeeled
2531	Potatoes and products	2010019	Potato, Norland, raw, unpeeled
2531	Potatoes and products	2010020	Potato, raw
2531	Potatoes and products	2010021	Potato, Red LaSoda, raw, unpeeled
2531	Potatoes and products	2010022	Potato, red skin, raw
2531	Potatoes and products	2010023	Potato, red skin, raw
2531	Potatoes and products	2010025	Potato, Russet Burbank, raw, unpeeled
2531	Potatoes and products	2010026	Potato, Russet Norkotah, raw, unpeeled
2531	Potatoes and products	2010027	Potato, Russet, raw
2531	Potatoes and products	2010029	Potato, Superior, raw, unpeeled
2531	Potatoes and products	2010035	Potato, Yellow Finn, raw, unpeeled
2532	Cassava and products	2020016	Cassava, Kello 44/72, roots, raw
2532	Cassava and products	2020020	Cassava, Quelle 104/72, roots, raw
2532	Cassava and products	2020021	Cassava, raw
2533	Sweet potatoes	2010044	Sweet potato, brown skin, raw
2533	Sweet potatoes	2010049	Sweet potato, pink skin, raw
2533	Sweet potatoes	2010050	Sweet potato, raw
2533	Sweet potatoes	2010051	Sweet potato, raw
2533	Sweet potatoes	2010053	Sweet potato, white flesh, raw

Supplementary Table 4 (continued): NBS food matches for phytate

FBS_itemcode	FBS_itemname	PhyFoodComp1.0 food_item_id	food_name_english
2534	Roots, Other	2020027	Cocoyam, raw
2534	Roots, Other	2030015	Taro, raw
2534	Roots, Other	2040001	Taro (eddoe), raw
2534	Roots, Other	2050001	American yam bean root, IRNAS NÂ° 11
2534	Roots, Other	2050002	American yam bean root, IRNAS NÂ° 4
2534	Roots, Other	2050003	American yam bean root, IRNAS NÂ° 5
2534	Roots, Other	2050004	American yam bean root, IRNAS NÂ° 9
2534	Roots, Other	2050005	American yam bean root, Local
2534	Roots, Other	2050007	Anchote, roots, whole, raw
2534	Roots, Other	2050016	Jerusalem artichoke, raw
2535	Yams	2040006	Yam, Elephant, raw
2535	Yams	2040008	Yam, Hingurala, raw
2535	Yams	2040009	Yam, Ini ata, raw
2535	Yams	2040010	Yam, Kahata ala, raw
2535	Yams	2040011	Yam, Kombuwalli, raw
2535	Yams	2040012	Yam, ordinary, raw
2535	Yams	2040013	Yam, Raja ala, raw
2535	Yams	2040014	Yam, Rata ala, raw
2535	Yams	2040015	Yam, raw
2535	Yams	2040017	Yam, Thambala, raw
2535	Yams	2040018	Yam, tuber, raw
2535	Yams	2040020	Yam, wild, raw
2536	Sugar cane	14050002	Sugar
2537	Sugar beet	14050002	Sugar
2541	Sugar non-centrifugal	14050001	Jaggery
2542	Sugar (Raw Equivalent)	14050002	Sugar
2546	Beans	3010046	Bean, black turtle, dried
2546	Beans	3010047	Bean, black, split, raw
2546	Beans	3010048	Bean, brown, dried
2546	Beans	3010049	Bean, butter, dried
2546	Beans	3010051	Bean, great northern, dried
2546	Beans	3010053	Bean, Haricot kidney, raw
2546	Beans	3010054	Bean, pink, dried
2546	Beans	3010056	Bean, pinto and red, dried
2546	Beans	3010057	Bean, pinto, dried
2546	Beans	3010064	Bean, 'Vigna aconitifolia', seed, mature, sun-dried
2546	Beans	3010065	Bean, 'Vigna aconitifolia', seed, mature, sun-dried
2546	Beans	3010066	Bean, 'Vigna ambacensis', TVnu 306, Cream, seed, raw
2546	Beans	3010067	Bean, 'Vigna bourneae', seed, mature, sun-dried
2546	Beans	3010068	Bean, 'Vigna luteola', TVnu 24, Brown, seed, raw
2546	Beans	3010069	Bean, 'Vigna luteola', TVnu 29, Brown, seed, raw
2546	Beans	3010070	Bean, 'Vigna oblongifolia', TVnu 38, Brown-black, seed, raw
2546	Beans	3010073	Bean, 'Vigna racemosa', raw
2546	Beans	3010074	Bean, 'Vigna racemosa', TVnu 105, Brown mottled, seed, raw
2546	Beans	3010075	Bean, 'Vigna radiata', var. sublota, seed, mature, sun-dried
2546	Beans	3010076	Bean, 'Vigna reticulata', TVnu 225, Light Brown, seed, raw
2546	Beans	3010077	Bean, 'Vigna trilobata', seed, mature, sun-dried
2546	Beans	3010078	Bean, 'Vigna umbellata', seed, mature, sun-dried
2546	Beans	3010079	Bean, 'Vigna unguiculata dekindtiana', TVnu 278, Brown, seed, raw
2546	Beans	3010080	Bean, 'Vigna unguiculata', Black, seed, mature, sun-dried
2546	Beans	3010081	Bean, 'Vigna unguiculata', Maroon, seed, mature, sun-dried
2546	Beans	3010082	Bean, 'Vigna unguiculata', seed, mature, raw
2546	Beans	3010083	Bean, 'Vigna unguiculata', seed, mature, sun-dried
2546	Beans	3010084	Bean, 'Vigna vexillata macrosperma', TVnu 72, Green-brown, seed, raw
2546	Beans	3010085	Bean, 'Vigna vexillata macrosperma', TVnu 73A, Black, seed, raw
2546	Beans	3010086	Bean, 'Vigna vexillata', seed, mature, sun-dried
2546	Beans	3010087	Bean, 'Vigna vexillata', TVnu 71, Brown, seed, raw
2546	Beans	3010124	Black bean, raw
2546	Beans	3010125	Black gram, dal
2546	Beans	3010127	Black gram, whole, raw
2546	Beans	3010191	Common bean, A321, raw
2546	Beans	3010192	Common bean, A321, raw
2546	Beans	3010193	Common bean, A321, raw
2546	Beans	3010194	Common bean, A321, raw
2546	Beans	3010195	Common bean, A410, raw
2546	Beans	3010196	Common bean, A410, raw
2546	Beans	3010197	Common bean, A410, raw
2546	Beans	3010198	Common bean, Baki wake, seed, raw
2546	Beans	3010203	Common bean, Cal1, raw
2546	Beans	3010204	Common bean, Calima, raw
2546	Beans	3010205	Common bean, Calima, raw
2546	Beans	3010206	Common bean, Calima, raw
2546	Beans	3010207	Common bean, Calima, raw
2546	Beans	3010208	Common bean, Dermaso, raw
2546	Beans	3010209	Common bean, Diamante Negro, seed, raw
2546	Beans	3010212	Common bean, Dore, raw
2546	Beans	3010213	Common bean, Dore, raw
2546	Beans	3010214	Common bean, Horoz, raw
2546	Beans	3010215	Common bean, Inta Linea 628-08, bright red, raw
2546	Beans	3010216	Common bean, Inta Linea 628-09, dark red, raw
2546	Beans	3010217	Common bean, Inta Rojo, non-uniform red, raw
2546	Beans	3010218	Common bean, Kidney, 10kGy irradiated, raw
2546	Beans	3010219	Common bean, Kidney, 5kGy irradiated, raw
2546	Beans	3010220	Common bean, Kidney, 7.5kGy irradiated, raw
2546	Beans	3010222	Common bean, Kidney, fresh, raw
2546	Beans	3010223	Common bean, Kidney, raw
2546	Beans	3010224	Common bean, Kidney, raw
2546	Beans	3010225	Common bean, Kidney, red, raw
2546	Beans	3010226	Common bean, Kidney, red, whole, sun-dried

Supplementary Table 4 (continued): NBS food matches for phytate

FBS_itemcode	FBS_itemname	PhyFoodComp1.0 food_item_id	food_name_english
2546	Beans	3010228	Common bean, Kwakiul, seed, raw
2546	Beans	3010230	Common bean, Ouro branco, seed, raw
2546	Beans	3010234	Common bean, Perola, seed, raw
2546	Beans	3010237	Common bean, Pink-mottled cream, seed, raw
2546	Beans	3010241	Common bean, Pinto, raw
2546	Beans	3010242	Common bean, raw
2546	Beans	3010243	Common bean, Seker, raw
2546	Beans	3010244	Common bean, Sen46, black, raw
2546	Beans	3010246	Common bean, TalismÃ£, seed, raw
2546	Beans	3010249	Common bean, var. A321, seed, raw
2546	Beans	3010250	Common bean, var. A321, seed, raw
2546	Beans	3010251	Common bean, var. A321, seed, raw
2546	Beans	3010252	Common bean, var. A321, seed, raw
2546	Beans	3010253	Common bean, var. A410, seed, raw
2546	Beans	3010254	Common bean, var. A410, seed, raw
2546	Beans	3010255	Common bean, var. A410, seed, raw
2546	Beans	3010256	Common bean, var. Calima, seed, raw
2546	Beans	3010257	Common bean, var. Calima, seed, raw
2546	Beans	3010258	Common bean, var. Calima, seed, raw
2546	Beans	3010259	Common bean, var. Calima, seed, raw
2546	Beans	3010260	Common bean, var. Dore de Kirundo, seed, raw
2546	Beans	3010261	Common bean, var. Dore de Kirundo, seed, raw
2546	Beans	3010262	Common bean, White, seed, raw
2546	Beans	3010266	Common bean, white, whole, raw
2546	Beans	3010405	Faba bean, Qidou 2, raw
2546	Beans	3010406	Faba bean, Qidou 2, raw
2546	Beans	3010437	Field bean, black, raw
2546	Beans	3010438	Field bean, brown, raw
2546	Beans	3010439	Field bean, white, raw
2546	Beans	3010443	Green gram, dal
2546	Beans	3010444	Green gram, raw
2546	Beans	3010445	Green gram, split, raw
2546	Beans	3010450	Green gram, whole, raw
2546	Beans	3010457	Haricot bean, white, whole grain, sun-dried
2546	Beans	3010504	Lima bean, fresh, raw
2546	Beans	3010506	Lima bean, dried
2546	Beans	3010507	Lima bean, raw
2546	Beans	3010545	Moth bean, raw
2546	Beans	3010555	Mung bean, Asha, raw
2546	Beans	3010557	Mung bean, MH 124, raw
2546	Beans	3010558	Mung bean, MH 125, raw
2546	Beans	3010559	Mung bean, MH 318, raw
2546	Beans	3010560	Mung bean, MH 421, raw
2546	Beans	3010561	Mung bean, MH 539, raw
2546	Beans	3010562	Mung bean, MH 560, raw
2546	Beans	3010563	Mung bean, MH 564, raw
2546	Beans	3010572	Mung bean, Muskan, raw
2546	Beans	3010581	Mung bean, raw
2546	Beans	3010582	Mung bean, raw
2546	Beans	3010583	Mung bean, Satya, raw
2546	Beans	3010590	Mung bean, var. Giza-1, seed, whole, raw
2546	Beans	3010632	Rajmah, black, raw
2546	Beans	3010633	Rajmah, brown, raw
2546	Beans	3010634	Rajmah, red, raw
2546	Beans	3010641	Rice bean, Beziamah, seeds, light brown, raw
2546	Beans	3010642	Rice bean, Beziarnah, oven-dried
2546	Beans	3010645	Rice bean, raw
2546	Beans	3010646	Rice bean, RBL-121, oven-dried
2546	Beans	3010651	Rice bean, RBL-121, seeds, greenish brown, raw
2546	Beans	3010652	Rice bean, RBL-4, oven-dried
2546	Beans	3010657	Rice bean, RBL-4, seed, brown, raw
2546	Beans	3010658	Rice bean, RBL-6, oven-dried
2546	Beans	3010662	Rice bean, RBL-6, seed, light brown, raw
2546	Beans	3010693	Tepary bean, Domestic brown, raw
2546	Beans	3010694	Tepary bean, Domestic brown, raw
2546	Beans	3010695	Tepary bean, Domestic white, raw
2546	Beans	3010696	Tepary bean, Domestic white, raw
2546	Beans	3010697	Tepary bean, wild, raw
2547	Peas	3010052	Bean, green, split, raw
2547	Peas	3010441	Grass pea, split, dried
2547	Peas	3010442	Grass pea, split, dried
2547	Peas	3010451	Grass pea, dried
2547	Peas	3010592	Pea, 10kGy irradiated, dried
2547	Peas	3010593	Pea, 5kGy irradiated, dried
2547	Peas	3010594	Pea, 7.5kGy irradiated, dried
2547	Peas	3010595	Pea, Agat, dried
2547	Peas	3010596	Pea, Agra, dried
2547	Peas	3010597	Pea, Albatros, dried
2547	Peas	3010598	Pea, Ametyst, dried
2547	Peas	3010599	Pea, Diament, dried
2547	Peas	3010600	Pea, dried
2547	Peas	3010601	Pea, dried
2547	Peas	3010602	Pea, dried
2547	Peas	3010603	Pea, dried
2547	Peas	3010607	Pea, Ergo, dried
2547	Peas	3010608	Pea, field, whole grain, sun-dried
2547	Peas	3010609	Pea, Finale, whole seed, dried
2547	Peas	3010610	Pea, Hermes, dried
2547	Peas	3010611	Pea, Imposant, whole seed, dried

Supplementary Table 4 (continued): NBS food matches for phytate

FBS_itemcode	FBS_itemname	PhyFoodComp1.0 food_item_id	food_name_english
2547	Peas	3010612	Pea, Karat, dried
2547	Peas	3010613	Pea, Koral, dried
2547	Peas	3010614	Pea, Kwestor, dried
2547	Peas	3010615	Pea, Piast, dried
2547	Peas	3010616	Pea, Rodan, dried
2547	Peas	3010617	Pea, Rondo, whole seed, dried
2547	Peas	3010618	Pea, Rubin, dried
2547	Peas	3010619	Pea, split, dried
2547	Peas	3010620	Pea, split, dried
2547	Peas	3010621	Pea, Szafir, dried
2547	Peas	3010622	Pea, Tegma, dried
2549	Pulses, Other and product	3010002	African faba bean, seed, raw
2549	Pulses, Other and product	3010006	African locust bean, Boki, mature seeds, raw
2549	Pulses, Other and product	3010009	African locust bean, Obanliku, mature seeds, raw
2549	Pulses, Other and product	3010012	African locust bean, Obudu, mature seeds, raw
2549	Pulses, Other and product	3010015	African locust bean, raw
2549	Pulses, Other and product	3010017	African oil bean, raw
2549	Pulses, Other and product	3010019	African oil bean, seed, raw
2549	Pulses, Other and product	3010023	African yam bean, seed, raw
2549	Pulses, Other and product	3010031	Bambara groundnut, Ci12, raw
2549	Pulses, Other and product	3010032	Bambara groundnut, raw
2549	Pulses, Other and product	3010033	Bambara groundnut, raw
2549	Pulses, Other and product	3010039	Bambara groundnut, seed, raw
2549	Pulses, Other and product	3010040	Bambara groundnut, seed, red, raw
2549	Pulses, Other and product	3010041	Bambara groundnut, seed, white, raw
2549	Pulses, Other and product	3010044	Bambara groundnut, seed, raw
2549	Pulses, Other and product	3010050	Bean, 'Cassia laevigata', seed, raw
2549	Pulses, Other and product	3010090	Bean, White, California small white, raw
2549	Pulses, Other and product	3010091	Bean, 'Xylia xylocarpa', seed, sun-dried
2549	Pulses, Other and product	3010100	Bengal gram, brown, whole grain, raw
2549	Pulses, Other and product	3010101	Bengal gram, brown, whole grain, raw
2549	Pulses, Other and product	3010102	Bengal gram, brown, whole grain, raw
2549	Pulses, Other and product	3010111	Bengal gram, dal
2549	Pulses, Other and product	3010117	Bengal gram, white, whole grain, raw
2549	Pulses, Other and product	3010118	Bengal gram, white, whole grain, raw
2549	Pulses, Other and product	3010122	Bengal gram, whole, raw
2549	Pulses, Other and product	3010123	Bengal gram, whole, raw
2549	Pulses, Other and product	3010132	Broad bean, Giza 716, raw
2549	Pulses, Other and product	3010133	Broad bean, whole, raw
2549	Pulses, Other and product	3010140	Chickpea, C-235, grown organic, raw
2549	Pulses, Other and product	3010142	Chickpea, Desi, seed, raw
2549	Pulses, Other and product	3010143	Chickpea, grown inorganic, raw
2549	Pulses, Other and product	3010146	Chickpea, Kabuli, Giza 1, whole, dried
2549	Pulses, Other and product	3010149	Chickpea, Kabuli, Giza 2-L, whole, dried
2549	Pulses, Other and product	3010152	Chickpea, Kabuli, Giza 2-U, whole, dried
2549	Pulses, Other and product	3010153	Chickpea, Kabuli, seed, raw
2549	Pulses, Other and product	3010162	Chickpea, raw
2549	Pulses, Other and product	3010163	Chickpea, raw
2549	Pulses, Other and product	3010164	Chickpea, raw
2549	Pulses, Other and product	3010165	Chickpea, raw
2549	Pulses, Other and product	3010166	Chickpea, raw
2549	Pulses, Other and product	3010182	Chickpea, white, raw
2549	Pulses, Other and product	3010183	Chickpea, whole, raw
2549	Pulses, Other and product	3010184	Chickpea, whole, raw
2549	Pulses, Other and product	3010365	Cowpea, white, raw
2549	Pulses, Other and product	3010367	Faba bean, 1 irrigation/week, raw
2549	Pulses, Other and product	3010368	Faba bean, 2 irrigation/week, raw
2549	Pulses, Other and product	3010370	Faba bean, Aguadulce, whole seed, raw
2549	Pulses, Other and product	3010373	Faba bean, Big Qinpi, raw
2549	Pulses, Other and product	3010377	Faba bean, Giza 2, whole, raw
2549	Pulses, Other and product	3010380	Faba bean, Manfredini, whole seed, raw
2549	Pulses, Other and product	3010382	Faba bean, plant fertilized by 200 kg/ha P2O5 superphosphate and with 50 kg/ha sulphur, 1 irrigation/week, raw
2549	Pulses, Other and product	3010383	Faba bean, plant fertilized by 200 kg/ha P2O5 superphosphate and with 50 kg/ha sulphur, 2 irrigation/week, raw
2549	Pulses, Other and product	3010384	Faba bean, plant fertilized by 200 kg/ha P2O5 superphosphate, 1 irrigation/week, raw
2549	Pulses, Other and product	3010385	Faba bean, plant fertilized by 200 kg/ha P2O5 superphosphate, 2 irrigation/week, raw
2549	Pulses, Other and product	3010386	Faba bean, plant fertilized with 50 kg/ha sulphur, 1 irrigation/week, raw
2549	Pulses, Other and product	3010387	Faba bean, plant fertilized with 50 kg/ha sulphur, 2 irrigation/week, raw
2549	Pulses, Other and product	3010388	Faba bean, plant inoculated with mycorrhiza and fertilized by 200 kg/ha P2O5 superphosphate and with 50 kg/ha sulphur, 1 irrigation/week, raw
2549	Pulses, Other and product	3010389	Faba bean, plant inoculated with mycorrhiza and fertilized by 200 kg/ha P2O5 superphosphate and with 50 kg/ha sulphur, 2 irrigation/week, raw
2549	Pulses, Other and product	3010390	Faba bean, plant inoculated with mycorrhiza and fertilized by 200 kg/ha P2O5 superphosphate, 1 irrigation/week, raw
2549	Pulses, Other and product	3010391	Faba bean, plant inoculated with mycorrhiza and fertilized by 200 kg/ha P2O5 superphosphate, 2 irrigation/week, raw
2549	Pulses, Other and product	3010392	Faba bean, plant inoculated with mycorrhiza and fertilized with 50 kg/ha sulphur, 1 irrigation/week, raw
2549	Pulses, Other and product	3010393	Faba bean, plant inoculated with mycorrhiza and fertilized with 50 kg/ha sulphur, 2 irrigation/week, raw
2549	Pulses, Other and product	3010394	Faba bean, plant inoculated with mycorrhiza, 1 irrigation/week, raw
2549	Pulses, Other and product	3010395	Faba bean, plant inoculated with mycorrhiza, 2 irrigation/week, raw
2549	Pulses, Other and product	3010396	Faba bean, Polo, whole seed, raw
2549	Pulses, Other and product	3010416	Faba bean, raw
2549	Pulses, Other and product	3010417	Faba bean, raw
2549	Pulses, Other and product	3010421	Faba bean, Vesuvio, whole seed, raw
2549	Pulses, Other and product	3010436	Feathertree bean, raw
2549	Pulses, Other and product	3010458	Horse gram, whole, raw
2549	Pulses, Other and product	3010463	Itching bean, var. pruriens, Aliyar, raw
2549	Pulses, Other and product	3010464	Itching bean, var. pruriens, Anaikatti, raw
2549	Pulses, Other and product	3010465	Itching bean, var. pruriens, Ayyanarkoil, raw
2549	Pulses, Other and product	3010466	Itching bean, var. pruriens, Seithur, raw
2549	Pulses, Other and product	3010467	Itching bean, var. pruriens, Sivagiri, raw
2549	Pulses, Other and product	3010468	Jackbean, raw
2549	Pulses, Other and product	3010470	Lablab bean, raw
2549	Pulses, Other and product	3010472	Lentil, raw, 10kGy irradiated

Supplementary Table 4 (continued): NBS food matches for phytate

FBS_itemcode	FBS_itemname	PhyFoodComp1.0 food_item_id	food_name_english
2549	Pulses, Other and product	3010473	Lentil, raw, 5kGy irradiated
2549	Pulses, Other and product	3010474	Lentil, raw, 7.5kGy irradiated
2549	Pulses, Other and product	3010476	Lentil, Brown, raw
2549	Pulses, Other and product	3010477	Lentil, Brown, seed, raw
2549	Pulses, Other and product	3010483	Lentil, dal
2549	Pulses, Other and product	3010484	Lentil, Pardina, seed, raw
2549	Pulses, Other and product	3010488	Lentil, raw
2549	Pulses, Other and product	3010489	Lentil, raw
2549	Pulses, Other and product	3010490	Lentil, raw
2549	Pulses, Other and product	3010491	Lentil, raw
2549	Pulses, Other and product	3010492	Lentil, raw
2549	Pulses, Other and product	3010493	Lentil, raw
2549	Pulses, Other and product	3010494	Lentil, raw
2549	Pulses, Other and product	3010498	Lentil, whole grain, sun-dried
2549	Pulses, Other and product	3010499	Lentil, whole, brown, raw
2549	Pulses, Other and product	3010500	Lentil, whole, yellowish, raw
2549	Pulses, Other and product	3010518	Locust bean, seed, raw
2549	Pulses, Other and product	3010520	Longleaf Milkpea, dried
2549	Pulses, Other and product	3010536	Mexican palo verde bean, raw
2549	Pulses, Other and product	3010537	Mexican palo verde bean, raw
2549	Pulses, Other and product	3010538	Mexican palo verde bean, raw
2549	Pulses, Other and product	3010546	Mucuna, Mottle, seed, mature, air-dried, raw
2549	Pulses, Other and product	3010547	Mucuna, seed, mature, air-dried, raw
2549	Pulses, Other and product	3010548	Mucuna, seed, mature, air-dried, raw
2549	Pulses, Other and product	3010549	Mucuna, seed, mature, air-dried, raw
2549	Pulses, Other and product	3010550	Mucuna, seed, mature, air-dried, raw
2549	Pulses, Other and product	3010551	Mucuna, var. utilis, seed, mature, sun-dried, raw
2549	Pulses, Other and product	3010553	Mucuna, White, seed, mature, air-dried, raw
2549	Pulses, Other and product	3010627	Pigeon pea, raw
2549	Pulses, Other and product	3010628	Pigeon pea, raw
2549	Pulses, Other and product	3010635	Red gram, dal
2549	Pulses, Other and product	3010636	Red gram, raw
2549	Pulses, Other and product	3010638	Red gram, whole, raw
2549	Pulses, Other and product	3010664	Sesbania, raw
2549	Pulses, Other and product	3010665	Sesbania, raw
2549	Pulses, Other and product	3010666	Sesbania, raw
2549	Pulses, Other and product	3010684	Stink bean, dry heated
2549	Pulses, Other and product	3010688	Stink bean, raw
2549	Pulses, Other and product	3010698	Velvet bean, black coloured seed coat, raw
2549	Pulses, Other and product	3010700	Velvet bean, Black, whole seed, raw
2549	Pulses, Other and product	3010701	Velvet bean, Cream, seed, sun-dried
2549	Pulses, Other and product	3010702	Velvet bean, Maroon, seed, sun-dried
2549	Pulses, Other and product	3010703	Velvet bean, Mottled, seed, sun-dried
2549	Pulses, Other and product	3010704	Velvet bean, Mottled, seed, sun-dried
2549	Pulses, Other and product	3010705	Velvet bean, seed, mature, sun-dried
2549	Pulses, Other and product	3010706	Velvet bean, seed, sun-dried
2549	Pulses, Other and product	3010707	Velvet bean, seed, sun-dried
2549	Pulses, Other and product	3010708	Velvet bean, underutilized type, raw
2549	Pulses, Other and product	3010709	Velvet bean, white coloured seed coat, raw
2549	Pulses, Other and product	3010712	Velvet bean, White, whole seed, raw
2549	Pulses, Other and product	3010713	Velvet bean, White, whole seed, raw
2549	Pulses, Other and product	3010714	Winged bean, 034(b), raw
2549	Pulses, Other and product	3010715	Winged bean, 039(a)a, raw
2549	Pulses, Other and product	3010716	Winged bean, 042(a), raw
2549	Pulses, Other and product	3010717	Winged bean, 044(a), raw
2549	Pulses, Other and product	3010718	Winged bean, 046(b)/ba, raw
2549	Pulses, Other and product	3010719	Winged bean, 048(a), raw
2549	Pulses, Other and product	3010720	Winged bean, 050(a), raw
2549	Pulses, Other and product	3010721	Winged bean, 051(b), raw
2549	Pulses, Other and product	3010722	Winged bean, 079(c)/b(a), raw
2549	Pulses, Other and product	3010723	Winged bean, 157(d), raw
2549	Pulses, Other and product	3010724	Winged bean, 181(b), raw
2549	Pulses, Other and product	3010725	Winged bean, 184(a)(b), raw
2549	Pulses, Other and product	3010726	Winged bean, 188(d), raw
2549	Pulses, Other and product	3010727	Winged bean, 195(b), raw
2549	Pulses, Other and product	3010728	Winged bean, 201(a), raw
2549	Pulses, Other and product	3010729	Winged bean, 207, raw
2549	Pulses, Other and product	4030054	Grass pea, fresh, raw
2549	Pulses, Other and product	4030055	Grass pea, fresh, raw
2549	Pulses, Other and product	4030062	Grass pea, Kwestor, raw
2551	Nuts and products	6010016	Almond, raw
2551	Nuts and products	6010019	Arecanut, brown, dried
2551	Nuts and products	6010020	Arecanut, fresh, raw
2551	Nuts and products	6010021	Arecanut, red, dried
2551	Nuts and products	6010032	Brazil nut, raw
2551	Nuts and products	6010036	Cashew nut, raw
2551	Nuts and products	6010037	Cashew nut, raw
2551	Nuts and products	6010038	Cashew nut, raw
2551	Nuts and products	6010079	Hazelnut, raw
2551	Nuts and products	6010098	Macadamia nut, raw
2551	Nuts and products	6010099	Macadamia nuts, raw
2551	Nuts and products	6010146	Pecan nut, Desirable, raw
2551	Nuts and products	6010150	Pine nut, raw
2551	Nuts and products	6010151	Pine nut, raw
2551	Nuts and products	6010154	Pistachio, raw
2551	Nuts and products	6010182	Walnut, raw
2551	Nuts and products	6010183	Walnut, raw
2551	Nuts and products	6010201	Wonderful kola, seed, raw
2555	Soyabeans	3020034	Soybean, Asmara, mature seed (6 weeks), raw

Supplementary Table 4 (continued): NBS food matches for phytate

FBS_itemcode	FBS_itemname	PhyFoodComp1.0 food_item_id	food_name_english
2555	Soyabeans	3020035	Soybean, brown, raw
2555	Soyabeans	3020036	Soybean, C & W brand
2555	Soyabeans	3020038	Soybean, Crawford, whole, raw
2555	Soyabeans	3020043	Soybean, Daewon, dried
2555	Soyabeans	3020044	Soybean, Dekabig, raw
2555	Soyabeans	3020045	Soybean, dried
2555	Soyabeans	3020046	Soybean, dried
2555	Soyabeans	3020047	Soybean, dried
2555	Soyabeans	3020050	Soybean, Giza 82, raw
2555	Soyabeans	3020051	Soybean, Glyphosate-Tolerant Soybean 40-3-2, raw
2555	Soyabeans	3020052	Soybean, Jinpum n.2, dried
2555	Soyabeans	3020057	Soybean, Mooncake, mature seed (6 weeks), raw
2555	Soyabeans	3020059	Soybean, prennial, dried
2555	Soyabeans	3020060	Soybean, Puleun, dried
2555	Soyabeans	3020061	Soybean, raw
2555	Soyabeans	3020062	Soybean, raw
2555	Soyabeans	3020063	Soybean, raw
2555	Soyabeans	3020064	Soybean, Red Mill brand, raw
2555	Soyabeans	3020065	Soybean, Safeway brand, sweet
2555	Soyabeans	3020066	Soybean, Seonheuk, dried
2555	Soyabeans	3020068	Soybean, Sinpaldal n.2, dried
2555	Soyabeans	3020069	Soybean, Somyeong, dried
2555	Soyabeans	3020070	Soybean, Taewang, dried
2555	Soyabeans	3020071	Soybean, Taiwan 75-M, dried, 150Gy irradiated
2555	Soyabeans	3020072	Soybean, Taiwan 75-M, dried, 150Gy irradiated
2555	Soyabeans	3020073	Soybean, Taiwan 75-P, dried, 150Gy irradiated
2555	Soyabeans	3020074	Soybean, Taiwan 75-P, dried, 150Gy irradiated
2555	Soyabeans	3020077	Soybean, white, raw
2555	Soyabeans	3020079	Soybean, ZC3-M, dried, 150Gy irradiated
2555	Soyabeans	3020080	Soybean, ZC3-M, dried, 150Gy irradiated
2555	Soyabeans	3020081	Soybean, ZC3-P, dried, 150Gy irradiated
2555	Soyabeans	3020082	Soybean, ZC3-P, dried, 150Gy irradiated
2555	Soyabeans	3020083	Soybeans, Bragg, 112kg of P2O5/ha, raw
2555	Soyabeans	3020084	Soybeans, Bragg, 56kg of P2O5/ha, raw
2555	Soyabeans	3020085	Soybeans, Bragg, raw
2555	Soyabeans	3020086	Soybeans, Punja-1, 112kg of P2O5/ha, raw
2555	Soyabeans	3020087	Soybeans, Punja-1, 56kg of P2O5/ha, raw
2555	Soyabeans	3020088	Soybeans, Punja-1, raw
2556	Groundnuts (Shelled Eq)	6010069	Ground nut, raw
2556	Groundnuts (Shelled Eq)	6010073	Groundnut, fresh, raw
2556	Groundnuts (Shelled Eq)	6010074	Groundnut, mature, raw
2556	Groundnuts (Shelled Eq)	6010127	Peanut, raw
2556	Groundnuts (Shelled Eq)	6010139	Peanut, raw
2556	Groundnuts (Shelled Eq)	6010140	Peanut, raw
2556	Groundnuts (Shelled Eq)	6010141	Peanut, raw
2557	Sunflower seed	6010173	Sunflower, seed, raw
2558	Rape and Mustardseed	6010120	Mustard, seed, raw
2559	Cottonseed	6010049	Cottonseed, seed flour, ISA BC4, dehulled, defatted
2560	Coconuts - Incl Copra	5020021	Coconut, kernel, fresh, raw
2561	Sesame seed	6010161	Sesame seed, grown inorganic, raw
2561	Sesame seed	6010162	Sesame seed, HT-1, grown organic, raw
2561	Sesame seed	6010163	Sesame seed, raw
2561	Sesame seed	6010164	Sesame seed, whole, raw
2562	Palm kernels	Wessels et al (2012)	Palm kernels
2570	Oilcrops, Other	6010089	Linseed, raw
2570	Oilcrops, Other	6010155	Poppy seed, McCormick
2570	Oilcrops, Other	6010160	Safflower, seed, raw
2572	Groundnut Oil	12010003	Groundnut oil
2577	Palm Oil	12020001	Palm oil, orange
2601	Tomatoes and products	4030107	Tomato, bitter, raw
2601	Tomatoes and products	4030108	Tomato, green, raw
2601	Tomatoes and products	4030109	Tomato, raw
2601	Tomatoes and products	4030110	Tomato, ripe, hybrid, raw
2601	Tomatoes and products	4030111	Tomato, ripe, raw
2602	Onions	4030087	Onion, big, raw
2602	Onions	4030088	Onion, raw
2602	Onions	4030089	Onion, raw
2602	Onions	4030090	Onion, small, raw
2605	Vegetables, Other	2050008	Beet root, raw
2605	Vegetables, Other	2050009	Beet, red beet, raw
2605	Vegetables, Other	2050014	Fluted pumpkin root, mature plant, raw
2605	Vegetables, Other	2050021	Parsnip, raw
2605	Vegetables, Other	2050022	Radish, raw
2605	Vegetables, Other	2050029	Turnip, raw
2605	Vegetables, Other	2050030	Turnip, raw
2605	Vegetables, Other	4010002	African lettuce, leaves, raw
2605	Vegetables, Other	4010006	Amaranth globe, ABS-38-AWKA, dried
2605	Vegetables, Other	4010008	Amaranth globe, AKS-33-EKPENE EDIENE x ABS-38-AWKA, dried
2605	Vegetables, Other	4010010	Amaranth globe, AKS-33-EKPENE EDIENE, dried
2605	Vegetables, Other	4010012	Amaranth globe, EBS-15-NKALAGU x ABS-38-AKWA, dried
2605	Vegetables, Other	4010014	Amaranth globe, EBS-15-NKALAGU x AKS-33-EKPENE EDIENE, dried
2605	Vegetables, Other	4010016	Amaranth globe, EBS-15-NKALAGU x IMS-20-NJIABA, dried
2605	Vegetables, Other	4010018	Amaranth globe, EBS-15-NKALAGU, dried
2605	Vegetables, Other	4010020	Amaranth globe, ENS-08-MBU x ABS-38-AWKA, dried
2605	Vegetables, Other	4010022	Amaranth globe, ENS-08-MBU x AKS-33-EKPENE EDIENE, dried
2605	Vegetables, Other	4010024	Amaranth globe, ENS-08-MBU x EBS-15-NKALAGU, dried
2605	Vegetables, Other	4010026	Amaranth globe, ENS-08-MBU x IMS-20-NJIABA, dried
2605	Vegetables, Other	4010028	Amaranth globe, ENS-08-MBU, dried
2605	Vegetables, Other	4010030	Amaranth globe, IMS-20-NJIABA x ABS-38-AWKA, dried
2605	Vegetables, Other	4010032	Amaranth globe, IMS-20-NJIABA x AKS-33-EKPENE EDIENE, dried

Supplementary Table 4 (continued): NBS food matches for phytate

FBS_itemcode	FBS_itemname	PhyFoodComp1.0 food_item_id	food_name_english
2605	Vegetables, Other	4010034	Amaranth globe, IMS-20-NJIABA, dried
2605	Vegetables, Other	4010055	Beet greens, raw
2605	Vegetables, Other	4010078	Brussels sprouts, raw
2605	Vegetables, Other	4010080	Buffalo spinach, leaves, raw
2605	Vegetables, Other	4010086	Cabbage, Chinese, raw
2605	Vegetables, Other	4010088	Cabbage, collard greens, raw
2605	Vegetables, Other	4010089	Cabbage, green, raw
2605	Vegetables, Other	4010093	Cabbage, raw
2605	Vegetables, Other	4010094	Cabbage, violet, raw
2605	Vegetables, Other	4010099	Cassava, leaves, destalked, raw
2605	Vegetables, Other	4010100	Cassava, leaves, raw
2605	Vegetables, Other	4010101	Cassava, leaves, raw
2605	Vegetables, Other	4010102	Cassava, leaves, raw
2605	Vegetables, Other	4010103	Cassava, mature leaves, raw
2605	Vegetables, Other	4010104	Cassava, very young leaves, raw
2605	Vegetables, Other	4010105	Cassava, young leaves, raw
2605	Vegetables, Other	4010108	Cauliflower, raw
2605	Vegetables, Other	4010109	Cauliflower, raw
2605	Vegetables, Other	4010113	Chinese violet, leaves, raw
2605	Vegetables, Other	4010123	Collard, raw
2605	Vegetables, Other	4010126	Common nettle, leaves, wild, raw
2605	Vegetables, Other	4010148	Garden cress, raw
2605	Vegetables, Other	4010170	Lettuce, raw
2605	Vegetables, Other	4010178	Mustard, leaves, raw
2605	Vegetables, Other	4010179	Mustard, leaves, raw
2605	Vegetables, Other	4010196	Pak Choi, leaves, raw
2605	Vegetables, Other	4010227	Sorrell, leaves, raw
2605	Vegetables, Other	4010229	Spinach, raw
2605	Vegetables, Other	4010248	Water cress, leaves, raw
2605	Vegetables, Other	4010254	Waterleaf leaves, raw
2605	Vegetables, Other	4010255	Waterleaf leaves, wild, raw
2605	Vegetables, Other	4010258	Yellow sawah lettuce, leaves, raw
2605	Vegetables, Other	4020001	Babycorn, raw
2605	Vegetables, Other	4020002	Carrot, orange, raw
2605	Vegetables, Other	4020003	Carrot, raw
2605	Vegetables, Other	4020004	Carrot, raw
2605	Vegetables, Other	4020005	Carrot, red, raw
2605	Vegetables, Other	4020006	Cucumber, orange, round, raw
2605	Vegetables, Other	4020007	Pumpkin, orange, round, raw
2605	Vegetables, Other	4020008	Sweet pepper, yellow, raw
2605	Vegetables, Other	4020009	Zucchini, yellow, raw
2605	Vegetables, Other	4030001	Ash gourd, raw
2605	Vegetables, Other	4030002	Avocado fruit, raw
2605	Vegetables, Other	4030003	Bamboo shoot, tender, raw
2605	Vegetables, Other	4030004	Bitter gourd, jagged, smooth ridges, elongate, raw
2605	Vegetables, Other	4030005	Bitter gourd, jagged, teeth ridges, elongate, raw
2605	Vegetables, Other	4030006	Bitter gourd, jagged, teeth ridges, short, raw
2605	Vegetables, Other	4030008	Bottle gourd, elongate, dark green, raw
2605	Vegetables, Other	4030009	Bottle gourd, elongate, pale green, raw
2605	Vegetables, Other	4030010	Bottle gourd, raw
2605	Vegetables, Other	4030011	Bottle gourd, round, pale green, raw
2605	Vegetables, Other	4030012	Capsicum, green, raw
2605	Vegetables, Other	4030013	Capsicum, red, raw
2605	Vegetables, Other	4030014	Celery stalk, raw
2605	Vegetables, Other	4030015	Cho-cho-marrow, raw
2605	Vegetables, Other	4030018	Cowslip creeper, raw
2605	Vegetables, Other	4030022	Cucumber, bitter, raw
2605	Vegetables, Other	4030024	Cucumber, green, elongate, raw
2605	Vegetables, Other	4030025	Cucumber, green, short, raw
2605	Vegetables, Other	4030027	Eggplant, Brinjal-1, raw
2605	Vegetables, Other	4030028	Eggplant, Brinjal-10, raw
2605	Vegetables, Other	4030029	Eggplant, Brinjal-11, raw
2605	Vegetables, Other	4030030	Eggplant, Brinjal-12, raw
2605	Vegetables, Other	4030031	Eggplant, Brinjal-13, raw
2605	Vegetables, Other	4030032	Eggplant, Brinjal-14, raw
2605	Vegetables, Other	4030033	Eggplant, Brinjal-15, raw
2605	Vegetables, Other	4030034	Eggplant, Brinjal-16, raw
2605	Vegetables, Other	4030035	Eggplant, Brinjal-17, raw
2605	Vegetables, Other	4030036	Eggplant, Brinjal-18, raw
2605	Vegetables, Other	4030037	Eggplant, Brinjal-19, raw
2605	Vegetables, Other	4030038	Eggplant, Brinjal-2, raw
2605	Vegetables, Other	4030039	Eggplant, Brinjal-20, raw
2605	Vegetables, Other	4030040	Eggplant, Brinjal-21, raw
2605	Vegetables, Other	4030041	Eggplant, Brinjal-3, raw
2605	Vegetables, Other	4030042	Eggplant, Brinjal-4, raw
2605	Vegetables, Other	4030043	Eggplant, Brinjal-5, raw
2605	Vegetables, Other	4030044	Eggplant, Brinjal-6, raw
2605	Vegetables, Other	4030045	Eggplant, Brinjal-7, raw
2605	Vegetables, Other	4030046	Eggplant, Brinjal-8, raw
2605	Vegetables, Other	4030047	Eggplant, Brinjal-9, raw
2605	Vegetables, Other	4030048	Eggplant, raw
2605	Vegetables, Other	4030063	Kovai, big, raw
2605	Vegetables, Other	4030064	Kovai, small, raw
2605	Vegetables, Other	4030065	Mushroom, 'Agaricus bisporus', raw
2605	Vegetables, Other	4030066	Mushroom, 'Auricularia auricula', dried
2605	Vegetables, Other	4030067	Mushroom, 'Calvatia cyathiformis', dried
2605	Vegetables, Other	4030068	Mushroom, 'Hirneola auriculajudae', fresh, raw
2605	Vegetables, Other	4030069	Mushroom, 'Lentinus brunneofloccosus', fresh, raw
2605	Vegetables, Other	4030070	Mushroom, 'Lentinus subnudud', dried

Supplementary Table 4 (continued): NBS food matches for phytate

FBS_itemcode	FBS_itemname	PhyFoodComp1.0 food_item_id	food_name_english
2605	Vegetables, Other	4030071	Mushroom, 'Pleurotus ostreatus', fresh, raw
2605	Vegetables, Other	4030072	Mushroom, 'Pleurotus sajor caju', raw
2605	Vegetables, Other	4030073	Mushroom, 'Psathyrella atroumbonata', dried
2605	Vegetables, Other	4030074	Mushroom, 'Psathyrella tuberculata', fresh, raw
2605	Vegetables, Other	4030075	Mushroom, 'Schizophyllum commune', dried
2605	Vegetables, Other	4030076	Mushroom, 'Termitomyces letestui', fresh, raw
2605	Vegetables, Other	4030077	Mushroom, 'Termitomyces microcarpus', dried
2605	Vegetables, Other	4030078	Mushroom, 'Termitomyces robustus', dried
2605	Vegetables, Other	4030079	Mushroom, 'Volvariella volvacea', fresh, raw
2605	Vegetables, Other	4030081	Okra, fruit, washed, dried
2605	Vegetables, Other	4030084	Okra, raw
2605	Vegetables, Other	4030085	Okra, raw
2605	Vegetables, Other	4030086	Okra, raw
2605	Vegetables, Other	4030092	Parwar, raw
2605	Vegetables, Other	4030094	Pumpkin, green, cylindrical, raw
2605	Vegetables, Other	4030095	Radish, elongate, red skin, raw
2605	Vegetables, Other	4030096	Radish, elongate, white skin, raw
2605	Vegetables, Other	4030097	Radish, round, red skin, raw
2605	Vegetables, Other	4030098	Radish, round, white skin, raw
2605	Vegetables, Other	4030099	Ridge gourd, raw
2605	Vegetables, Other	4030100	Ridge gourd, smooth skin, raw
2605	Vegetables, Other	4030101	Snake gourd, long, dark green, raw
2605	Vegetables, Other	4030102	Snake gourd, long, pale green, raw
2605	Vegetables, Other	4030103	Snake gourd, short, raw
2605	Vegetables, Other	4030106	Tinda, tender, raw
2605	Vegetables, Other	4030112	Zucchini, green, raw
2605	Vegetables, Other	15010021	Chili pepper, Green, raw
2605	Vegetables, Other	15010022	Chili pepper, Green-1, raw
2605	Vegetables, Other	15010023	Chili pepper, Green-2, raw
2605	Vegetables, Other	15010024	Chili pepper, Green-3, raw
2605	Vegetables, Other	15010025	Chili pepper, Green-4, raw
2605	Vegetables, Other	15010026	Chili pepper, Green-5, raw
2605	Vegetables, Other	15010027	Chili pepper, Green-6, raw
2605	Vegetables, Other	15010028	Chili pepper, Green-7, raw
2605	Vegetables, Other	15010029	Chili pepper, raw
2605	Vegetables, Other	15010055	Garlic, big clove, raw
2605	Vegetables, Other	15010058	Garlic, Kashmir, single clove, raw
2605	Vegetables, Other	15010059	Garlic, small clove, raw
2605	Vegetables, Other	15010075	Parsley, raw
2611	Oranges, Mandarines	5010030	Orange, raw
2614	Citrus, Other	5010012	Citron, fresh whole fruit, raw
2615	Bananas	5010006	Banana, Montham, ripe, raw
2615	Bananas	5010007	Banana, Poovam, ripe, raw
2615	Bananas	5010008	Banana, raw
2615	Bananas	5010010	Banana, red, ripe, raw
2615	Bananas	5010011	Banana, Robusta, ripe, raw
2616	Plantains	2060010	Plantain, green, raw
2617	Apples and products	5020004	Apple, big, raw
2617	Apples and products	5020005	Apple, green, raw
2617	Apples and products	5020006	Apple, Kashmir, small, raw
2617	Apples and products	5020007	Apple, small, raw
2618	Pineapples and products	5010042	Pineapple, raw
2619	Dates	5030003	Dates, dried, dark brown
2619	Dates	5030004	Dates, dried, pale brown
2620	Grapes and products (excl	5020032	Grapes, fresh fruit, raw
2620	Grapes and products (excl	5020033	Grapes, seeded, round, black, raw
2620	Grapes and products (excl	5020034	Grapes, seeded, round, green, raw
2620	Grapes and products (excl	5020035	Grapes, seeded, round, red, raw
2620	Grapes and products (excl	5020036	Grapes, seedless, oval, black, raw
2620	Grapes and products (excl	5020037	Grapes, seedless, round, black, raw
2620	Grapes and products (excl	5020038	Grapes, seedless, round, green, raw
2625	Fruits, Other	5010013	Mango, Banganapalli, ripe, raw
2625	Fruits, Other	5010014	Mango, Gulabkhas, ripe, raw
2625	Fruits, Other	5010015	Mango, Himsagar, ripe, raw
2625	Fruits, Other	5010016	Mango, Kesar, ripe, raw
2625	Fruits, Other	5010017	Mango, Neelam, ripe, raw
2625	Fruits, Other	5010018	Mango, Paheri, ripe, raw
2625	Fruits, Other	5010019	Mango, ripe, raw
2625	Fruits, Other	5010020	Mango, ripe, raw
2625	Fruits, Other	5010021	Mango, Totapari, ripe, raw
2625	Fruits, Other	5010022	Mangosteen, raw
2625	Fruits, Other	5010023	Melon, orange flesh, ripe, raw
2625	Fruits, Other	5010025	Monkey-jack, yellowish-orange flesh, raw
2625	Fruits, Other	5010026	Musk melon, light orange flesh, ripe, raw
2625	Fruits, Other	5010027	Musk melon, orange flesh, raw
2625	Fruits, Other	5010028	Musk melon, yellow flesh, raw
2625	Fruits, Other	5010031	Papaya, raw
2625	Fruits, Other	5010032	Papaya, ripe, raw
2625	Fruits, Other	5010033	Papaya, ripe, raw
2625	Fruits, Other	5010034	Passion fruit, fresh pulp, raw
2625	Fruits, Other	5010039	Pawpaw, ripe, yellow, waxed, raw
2625	Fruits, Other	5010040	Pawpaw, ripe, yellow, waxed, raw
2625	Fruits, Other	5010041	Peach, raw
2625	Fruits, Other	5010044	Pomelo, raw
2625	Fruits, Other	5010045	Star fruit, raw
2625	Fruits, Other	5020008	Avocado pear, raw
2625	Fruits, Other	5020009	Bael fruit, raw
2625	Fruits, Other	5020017	Blackberry, raw
2625	Fruits, Other	5020019	Breadfruit, raw

Supplementary Table 4 (continued): NBS food matches for phytate

FBS_itemcode	FBS_itemname	PhyFoodComp1.0 food_item_id	food_name_english
2625	Fruits, Other	5020020	Cherries, red, raw
2625	Fruits, Other	5020022	Currants, black, raw
2625	Fruits, Other	5020023	Custard apple, fresh whole, fruit without seeds
2625	Fruits, Other	5020024	Custard apple, raw
2625	Fruits, Other	5020025	Emblic, raw
2625	Fruits, Other	5020026	Fig, green, raw
2625	Fruits, Other	5020027	Fig, raw
2625	Fruits, Other	5020028	Fig, ripe, raw
2625	Fruits, Other	5020031	Gooseberry, raw
2625	Fruits, Other	5020039	Guava, fresh whole fruit, raw
2625	Fruits, Other	5020040	Guava, pink flesh, raw
2625	Fruits, Other	5020041	Guava, white flesh, raw
2625	Fruits, Other	5020043	Indian cucumber, whole fruit, raw
2625	Fruits, Other	5020044	Indian fig tree, whole fruit, raw
2625	Fruits, Other	5020045	Jackfruit, raw
2625	Fruits, Other	5020046	Jackfruit, ripe, raw
2625	Fruits, Other	5020047	Jambolan, raw
2625	Fruits, Other	5020048	Jambu fruit, ripe, raw
2625	Fruits, Other	5020049	Jujube, wihout seeds, raw
2625	Fruits, Other	5020051	Karonda fruit, raw
2625	Fruits, Other	5020052	Litchi, raw
2625	Fruits, Other	5020053	Mango, green, raw
2625	Fruits, Other	5020054	Mango, unripe, raw
2625	Fruits, Other	5020055	Manila tamarind, raw
2625	Fruits, Other	5020056	Mello, fresh whole fruit, raw
2625	Fruits, Other	5020057	Pear, raw
2625	Fruits, Other	5020059	Plum, raw
2625	Fruits, Other	5020060	Pomegranate, maroon seeds, raw
2625	Fruits, Other	5020061	Prickly pear, fresh flesh
2625	Fruits, Other	5020062	Quince, OHM13, fruit, raw
2625	Fruits, Other	5020063	Quince, OHM14, fruit, raw
2625	Fruits, Other	5020064	Quince, OHM2, fruit, raw
2625	Fruits, Other	5020065	Quince, PUM, fruit, raw
2625	Fruits, Other	5020066	Quince, ZM6, fruit, raw
2625	Fruits, Other	5020067	Quince, ZM9, fruit, raw
2625	Fruits, Other	5020068	Rambutan, raw
2625	Fruits, Other	5020072	Strawberry, raw
2625	Fruits, Other	5020077	Watermelon (tinda), raw
2625	Fruits, Other	5020078	Watermelon, dark green, raw
2625	Fruits, Other	5020079	Watermelon, pale green, raw
2625	Fruits, Other	5020080	Watermelon, ripe, raw
2625	Fruits, Other	5020083	Zizyphus, raw
2630	Coffee and products	13030007	Coffee for brewing, dried, Maxwell House, Master Blend
2633	Cocoa Beans and products	13030002	Cocoa powder, industrial
2633	Cocoa Beans and products	13030003	Cocoa powder, industrial
2633	Cocoa Beans and products	13030004	Cocoa powder, industrial
2633	Cocoa Beans and products	13030005	Cocoa powder, industrial
2633	Cocoa Beans and products	13030006	Cocoa powder, industrial
2635	Tea (including mate)	13030020	Tea for brewing, dried, Lipton, Decaffeinated
2635	Tea (including mate)	13030021	Tea, instant, Nestea
2640	Pepper	15010008	African pepper seed, dried
2640	Pepper	15010014	Black pepper, dried
2640	Pepper	15010015	Black pepper, dried, powdered
2640	Pepper	15010066	Lalot pepper, leaves, raw
2640	Pepper	15010078	Pippali, dried
2641	Pimento	15010020	Chili pepper, dried
2641	Pimento	15010030	Chili pepper, red, dried
2641	Pimento	15010080	Red chilies, dried, powdered
2641	Pimento	15010083	Sweet pepper, seed kernel
2642	Cloves	15010032	Clove, dried
2642	Cloves	15010034	Clove, seed, dried
2645	Spices, Other	4010106	Cassia, leaves, fresh, raw
2645	Spices, Other	6010008	African nutmeg, seed, dried
2645	Spices, Other	6010009	African nutmeg, seed, dried
2645	Spices, Other	15010001	African basil (scent leaves), dried
2645	Spices, Other	15010002	African basil (scent leaves), raw
2645	Spices, Other	15010003	African basil, dried
2645	Spices, Other	15010004	African basil, leaves, raw
2645	Spices, Other	15010005	African basil, raw
2645	Spices, Other	15010009	African spice, 'Cochlospermum spp.', rootâ powder
2645	Spices, Other	15010010	Ajowan, dried, powdered
2645	Spices, Other	15010018	Cardamom, black, dried
2645	Spices, Other	15010019	Cardamom, green, dried
2645	Spices, Other	15010031	Cinnamon, leaves, dried
2645	Spices, Other	15010036	Coriander, dried, powdered
2645	Spices, Other	15010037	Coriander, leaves, raw
2645	Spices, Other	15010038	Coriander, seeds, dried
2645	Spices, Other	15010039	Culantro, leaves, raw
2645	Spices, Other	15010040	Culantro, leaves, raw
2645	Spices, Other	15010041	Cumin, seeds, dried
2645	Spices, Other	15010042	Cumin, seeds, dried, powdered
2645	Spices, Other	15010043	Curry leaves, raw
2645	Spices, Other	15010044	Curry tree, leaves, raw
2645	Spices, Other	15010049	Fenugreek seed, dried
2645	Spices, Other	15010050	Fenugreek seed, Pusa, dried
2645	Spices, Other	15010053	Fenugreek seed, raw
2645	Spices, Other	15010054	Fenugreek seed, white, roasted, powdered
2645	Spices, Other	15010060	Ginger, dried, powdered
2645	Spices, Other	15010061	Ginger, fresh, raw

Supplementary Table 4 (continued): NBS food matches for phytate

FBS_itemcode	FBS_itemname	PhyFoodComp1.0 food_item_id	food_name_english
2645	Spices, Other	15010067	Mace, dried
2645	Spices, Other	15010068	Mango ginger, raw
2645	Spices, Other	15010069	Mentha, leaves, raw
2645	Spices, Other	15010070	Mexican mint, leaves, water-washed, raw
2645	Spices, Other	15010071	Mint, leaves, raw
2645	Spices, Other	15010072	Nutmeg, seed, dried
2645	Spices, Other	15010073	Nutmeg, seed, dried
2645	Spices, Other	15010074	Omum, dried
2645	Spices, Other	15010076	Pepper, Alligator, big variety, seed, dried
2645	Spices, Other	15010077	Pepper, Alligator, small variety, seed, dried
2645	Spices, Other	15010079	Poppy seeds, dried
2645	Spices, Other	15010084	Turmeric, dried, powdered
2761	Freshwater Fish	10010001	Tilapia fish, boiled
2761	Freshwater Fish	10070003	Tilapia fish, dried
2763	Pelagic Fish	10020001	Herring (west african herring), boiled
2763	Pelagic Fish	10030001	Spanish fish, large
2763	Pelagic Fish	10070001	Herring (west african herring), dried
2763	Pelagic Fish	10070002	Spanish fish, dried
2764	Marine Fish, Other	10010001	Tilapia fish, boiled
2764	Marine Fish, Other	10020001	Herring (west african herring), boiled
2764	Marine Fish, Other	10030001	Spanish fish, large
2764	Marine Fish, Other	10070001	Herring (west african herring), dried
2764	Marine Fish, Other	10070002	Spanish fish, dried
2764	Marine Fish, Other	10070003	Tilapia fish, dried
2775	Aquatic Plants	2050018	Lotus root, raw
2775	Aquatic Plants	4010247	Water chestnut, leaves, raw
2775	Aquatic Plants	4010249	Water hyacinth, leaves, raw
2775	Aquatic Plants	4010250	Water spinach, leaves, raw
2775	Aquatic Plants	4010251	Water spinach, leaves, raw
2775	Aquatic Plants	4010252	Water spinach, leaves, raw
2775	Aquatic Plants	4010253	Water spinach, raw
2805	Rice (Milled Equivalent)	1010063	Rice, Agb0101, brown, genetically modified, raw
2805	Rice (Milled Equivalent)	1010064	Rice, Anjung, conventional, brown, raw
2805	Rice (Milled Equivalent)	1010065	Rice, Bar68-1, brown, genetically modified, raw, milled
2805	Rice (Milled Equivalent)	1010066	Rice, Bar68-1, brown, genetically modified, raw
2805	Rice (Milled Equivalent)	1010067	Rice, Basmati, parboiled
2805	Rice (Milled Equivalent)	1010068	Rice, Bengal, rough, nontransgenic, conventional herbicide system, raw
2805	Rice (Milled Equivalent)	1010069	Rice, Bengal, rough, transgenic, conventional herbicide system, raw
2805	Rice (Milled Equivalent)	1010070	Rice, Bengal, rough, transgenic, liberty herbicide system, raw
2805	Rice (Milled Equivalent)	1010071	Rice, Bg 352/Bg300, parboiled
2805	Rice (Milled Equivalent)	1010072	Rice, Bg 352/Bg300, parboiled
2805	Rice (Milled Equivalent)	1010073	Rice, Bg 352/Bg300, parboiled
2805	Rice (Milled Equivalent)	1010074	Rice, Bg 352/Bg300, parboiled
2805	Rice (Milled Equivalent)	1010075	Rice, Bg 352/Bg300, raw
2805	Rice (Milled Equivalent)	1010076	Rice, Bg 352/Bg300, raw
2805	Rice (Milled Equivalent)	1010077	Rice, Bg 352/Bg300, raw
2805	Rice (Milled Equivalent)	1010078	Rice, Bg 352/Bg300, raw
2805	Rice (Milled Equivalent)	1010079	Rice, Bg 379-2, parboiled
2805	Rice (Milled Equivalent)	1010080	Rice, Bg 379-2, parboiled
2805	Rice (Milled Equivalent)	1010081	Rice, Bg 379-2, raw
2805	Rice (Milled Equivalent)	1010082	Rice, Bg 379-2, raw
2805	Rice (Milled Equivalent)	1010083	Rice, Bg403, raw
2805	Rice (Milled Equivalent)	1010084	Rice, Bg94-1/At354, parboiled
2805	Rice (Milled Equivalent)	1010085	Rice, Bg94-1/At354, raw
2805	Rice (Milled Equivalent)	1010086	Rice, Bg94-1/At354, raw
2805	Rice (Milled Equivalent)	1010087	Rice, black, raw
2805	Rice (Milled Equivalent)	1010091	Rice, BR-28, parboiled, milled
2805	Rice (Milled Equivalent)	1010092	Rice, brown, dried, Manischewitz (kosher)
2805	Rice (Milled Equivalent)	1010094	Rice, brown, raw
2805	Rice (Milled Equivalent)	1010095	Rice, brown, raw
2805	Rice (Milled Equivalent)	1010096	Rice, brown, raw
2805	Rice (Milled Equivalent)	1010097	Rice, brown, raw
2805	Rice (Milled Equivalent)	1010099	Rice, Calrose, brown, raw
2805	Rice (Milled Equivalent)	1010102	Rice, D68, brown, raw, milled
2805	Rice (Milled Equivalent)	1010103	Rice, D68, brown, raw
2805	Rice (Milled Equivalent)	1010104	Rice, Dongjin, conventional, brown, raw
2805	Rice (Milled Equivalent)	1010107	Rice, Giant embryonic, raw
2805	Rice (Milled Equivalent)	1010108	Rice, Goami, raw
2805	Rice (Milled Equivalent)	1010109	Rice, Green, raw
2805	Rice (Milled Equivalent)	1010110	Rice, H-4, parboiled
2805	Rice (Milled Equivalent)	1010111	Rice, H-4, raw
2805	Rice (Milled Equivalent)	1010112	Rice, Heilongjiang, raw
2805	Rice (Milled Equivalent)	1010113	Rice, Iksan483, genetically modified, brown, raw
2805	Rice (Milled Equivalent)	1010114	Rice, Indica, brown, raw
2805	Rice (Milled Equivalent)	1010115	Rice, Indica, dehulled, raw, milled
2805	Rice (Milled Equivalent)	1010116	Rice, Indica, Liangyou 2186, brown, raw
2805	Rice (Milled Equivalent)	1010117	Rice, Indica, Liangyou 2186, milled, raw
2805	Rice (Milled Equivalent)	1010118	Rice, Indica, Liangyou Kefeng No. 6, brown, genetically modified, raw
2805	Rice (Milled Equivalent)	1010119	Rice, Indica, Liangyou Kefeng No. 6, genetically modified, milled, raw
2805	Rice (Milled Equivalent)	1010130	Rice, IRR, brown, raw
2805	Rice (Milled Equivalent)	1010131	Rice, IRR, dehulled, milled, raw
2805	Rice (Milled Equivalent)	1010132	Rice, Japonica, Huai269, dehulled, dried, high N fertilization
2805	Rice (Milled Equivalent)	1010133	Rice, Japonica, Huai269, dehulled, dried, high N fertilization
2805	Rice (Milled Equivalent)	1010134	Rice, Japonica, Huai269, dehulled, dried, moderate N fertilization
2805	Rice (Milled Equivalent)	1010135	Rice, Japonica, Huai269, dehulled, dried, moderate N fertilization
2805	Rice (Milled Equivalent)	1010136	Rice, Japonica, Huai269, dehulled, dried, without N fertilization
2805	Rice (Milled Equivalent)	1010137	Rice, Japonica, Huaidao6, dehulled, dried, high N fertilization
2805	Rice (Milled Equivalent)	1010138	Rice, Japonica, Huaidao6, dehulled, dried, high N fertilization
2805	Rice (Milled Equivalent)	1010139	Rice, Japonica, Huaidao6, dehulled, dried, moderate N fertilization

Supplementary Table 4 (continued): NBS food matches for phytate

FBS_itemcode	FBS_itemname	PhyFoodComp1.0 food_item_id	food_name_english
2805	Rice (Milled Equivalent)	1010140	Rice, Japonica, Huaidao6, dehulled, dried, moderate N fertilization
2805	Rice (Milled Equivalent)	1010141	Rice, Japonica, Huaidao6, dehulled, dried, without N fertilization
2805	Rice (Milled Equivalent)	1010142	Rice, Japonica, Huaidao7, dehulled, dried, high N fertilization
2805	Rice (Milled Equivalent)	1010143	Rice, Japonica, Huaidao7, dehulled, dried, high N fertilization
2805	Rice (Milled Equivalent)	1010144	Rice, Japonica, Huaidao7, dehulled, dried, moderate N fertilization
2805	Rice (Milled Equivalent)	1010145	Rice, Japonica, Huaidao7, dehulled, dried, moderate N fertilization
2805	Rice (Milled Equivalent)	1010146	Rice, Japonica, Huaidao7, dehulled, dried, without N fertilization
2805	Rice (Milled Equivalent)	1010147	Rice, Japonica, Huaidao9, dehulled, dried, high N fertilization
2805	Rice (Milled Equivalent)	1010148	Rice, Japonica, Huaidao9, dehulled, dried, high N fertilization
2805	Rice (Milled Equivalent)	1010149	Rice, Japonica, Huaidao9, dehulled, dried, moderate N fertilization
2805	Rice (Milled Equivalent)	1010150	Rice, Japonica, Huaidao9, dehulled, dried, moderate N fertilization
2805	Rice (Milled Equivalent)	1010151	Rice, Japonica, Huaidao9, dehulled, dried, without N fertilization
2805	Rice (Milled Equivalent)	1010152	Rice, Japonica, Huajing6, dehulled, dried, high N fertilization
2805	Rice (Milled Equivalent)	1010153	Rice, Japonica, Huajing6, dehulled, dried, high N fertilization
2805	Rice (Milled Equivalent)	1010154	Rice, Japonica, Huajing6, dehulled, dried, moderate N fertilization
2805	Rice (Milled Equivalent)	1010155	Rice, Japonica, Huajing6, dehulled, dried, moderate N fertilization
2805	Rice (Milled Equivalent)	1010156	Rice, Japonica, Huajing6, dehulled, dried, without N fertilization
2805	Rice (Milled Equivalent)	1010157	Rice, Japonica, Nanjing43, dehulled, dried, high N fertilization
2805	Rice (Milled Equivalent)	1010158	Rice, Japonica, Nanjing43, dehulled, dried, high N fertilization
2805	Rice (Milled Equivalent)	1010159	Rice, Japonica, Nanjing43, dehulled, dried, moderate N fertilization
2805	Rice (Milled Equivalent)	1010160	Rice, Japonica, Nanjing43, dehulled, dried, moderate N fertilization
2805	Rice (Milled Equivalent)	1010161	Rice, Japonica, Nanjing43, dehulled, dried, without N fertilization
2805	Rice (Milled Equivalent)	1010162	Rice, Japonica, Ningjing1, dehulled, dried, high N fertilization
2805	Rice (Milled Equivalent)	1010163	Rice, Japonica, Ningjing1, dehulled, dried, high N fertilization
2805	Rice (Milled Equivalent)	1010164	Rice, Japonica, Ningjing1, dehulled, dried, moderate N fertilization
2805	Rice (Milled Equivalent)	1010165	Rice, Japonica, Ningjing1, dehulled, dried, moderate N fertilization
2805	Rice (Milled Equivalent)	1010166	Rice, Japonica, Ningjing1, dehulled, dried, without N fertilization
2805	Rice (Milled Equivalent)	1010167	Rice, Japonica, Ningjing2, dehulled, dried, high N fertilization
2805	Rice (Milled Equivalent)	1010168	Rice, Japonica, Ningjing2, dehulled, dried, high N fertilization
2805	Rice (Milled Equivalent)	1010169	Rice, Japonica, Ningjing2, dehulled, dried, moderate N fertilization
2805	Rice (Milled Equivalent)	1010170	Rice, Japonica, Ningjing2, dehulled, dried, moderate N fertilization
2805	Rice (Milled Equivalent)	1010171	Rice, Japonica, Ningjing2, dehulled, dried, without N fertilization
2805	Rice (Milled Equivalent)	1010172	Rice, Japonica, Ningjing3, dehulled, dried, high N fertilization
2805	Rice (Milled Equivalent)	1010173	Rice, Japonica, Ningjing3, dehulled, dried, high N fertilization
2805	Rice (Milled Equivalent)	1010174	Rice, Japonica, Ningjing3, dehulled, dried, moderate N fertilization
2805	Rice (Milled Equivalent)	1010175	Rice, Japonica, Ningjing3, dehulled, dried, moderate N fertilization
2805	Rice (Milled Equivalent)	1010176	Rice, Japonica, Ningjing3, dehulled, dried, without N fertilization
2805	Rice (Milled Equivalent)	1010177	Rice, Japonica, Sidao10, dehulled, dried, high N fertilization
2805	Rice (Milled Equivalent)	1010178	Rice, Japonica, Sidao10, dehulled, dried, high N fertilization
2805	Rice (Milled Equivalent)	1010179	Rice, Japonica, Sidao10, dehulled, dried, moderate N fertilization
2805	Rice (Milled Equivalent)	1010180	Rice, Japonica, Sidao10, dehulled, dried, moderate N fertilization
2805	Rice (Milled Equivalent)	1010181	Rice, Japonica, Sidao10, dehulled, dried, without N fertilization
2805	Rice (Milled Equivalent)	1010182	Rice, Japonica, Sujing45, dehulled, dried, high N fertilization
2805	Rice (Milled Equivalent)	1010183	Rice, Japonica, Sujing45, dehulled, dried, high N fertilization
2805	Rice (Milled Equivalent)	1010184	Rice, Japonica, Sujing45, dehulled, dried, moderate N fertilization
2805	Rice (Milled Equivalent)	1010185	Rice, Japonica, Sujing45, dehulled, dried, moderate N fertilization
2805	Rice (Milled Equivalent)	1010186	Rice, Japonica, Sujing45, dehulled, dried, without N fertilization
2805	Rice (Milled Equivalent)	1010187	Rice, Japonica, W3668, dehulled, dried, high N fertilization
2805	Rice (Milled Equivalent)	1010188	Rice, Japonica, W3668, dehulled, dried, high N fertilization
2805	Rice (Milled Equivalent)	1010189	Rice, Japonica, W3668, dehulled, dried, moderate N fertilization
2805	Rice (Milled Equivalent)	1010190	Rice, Japonica, W3668, dehulled, dried, moderate N fertilization
2805	Rice (Milled Equivalent)	1010191	Rice, Japonica, W3668, dehulled, dried, without N fertilization
2805	Rice (Milled Equivalent)	1010192	Rice, Japonica, Wu9516, dehulled, dried, high N fertilization
2805	Rice (Milled Equivalent)	1010193	Rice, Japonica, Wu9516, dehulled, dried, high N fertilization
2805	Rice (Milled Equivalent)	1010194	Rice, Japonica, Wu9516, dehulled, dried, moderate N fertilization
2805	Rice (Milled Equivalent)	1010195	Rice, Japonica, Wu9516, dehulled, dried, moderate N fertilization
2805	Rice (Milled Equivalent)	1010196	Rice, Japonica, Wu9516, dehulled, dried, without N fertilization
2805	Rice (Milled Equivalent)	1010197	Rice, Japonica, Wu9520, dehulled, dried, high N fertilization
2805	Rice (Milled Equivalent)	1010198	Rice, Japonica, Wu9520, dehulled, dried, high N fertilization
2805	Rice (Milled Equivalent)	1010199	Rice, Japonica, Wu9520, dehulled, dried, moderate N fertilization
2805	Rice (Milled Equivalent)	1010200	Rice, Japonica, Wu9520, dehulled, dried, moderate N fertilization
2805	Rice (Milled Equivalent)	1010201	Rice, Japonica, Wu9520, dehulled, dried, without N fertilization
2805	Rice (Milled Equivalent)	1010202	Rice, Japonica, Wu9522, dehulled, dried, high N fertilization
2805	Rice (Milled Equivalent)	1010203	Rice, Japonica, Wu9522, dehulled, dried, high N fertilization
2805	Rice (Milled Equivalent)	1010204	Rice, Japonica, Wu9522, dehulled, dried, moderate N fertilization
2805	Rice (Milled Equivalent)	1010205	Rice, Japonica, Wu9522, dehulled, dried, moderate N fertilization
2805	Rice (Milled Equivalent)	1010206	Rice, Japonica, Wu9522, dehulled, dried, without N fertilization
2805	Rice (Milled Equivalent)	1010207	Rice, Japonica, Wu9746, dehulled, dried, high N fertilization
2805	Rice (Milled Equivalent)	1010208	Rice, Japonica, Wu9746, dehulled, dried, high N fertilization
2805	Rice (Milled Equivalent)	1010209	Rice, Japonica, Wu9746, dehulled, dried, moderate N fertilization
2805	Rice (Milled Equivalent)	1010210	Rice, Japonica, Wu9746, dehulled, dried, moderate N fertilization
2805	Rice (Milled Equivalent)	1010211	Rice, Japonica, Wu9746, dehulled, dried, without N fertilization
2805	Rice (Milled Equivalent)	1010212	Rice, Japonica, Wujing15, dehulled, dried, high N fertilization
2805	Rice (Milled Equivalent)	1010213	Rice, Japonica, Wujing15, dehulled, dried, high N fertilization
2805	Rice (Milled Equivalent)	1010214	Rice, Japonica, Wujing15, dehulled, dried, moderate N fertilization
2805	Rice (Milled Equivalent)	1010215	Rice, Japonica, Wujing15, dehulled, dried, moderate N fertilization
2805	Rice (Milled Equivalent)	1010216	Rice, Japonica, Wujing15, dehulled, dried, without N fertilization
2805	Rice (Milled Equivalent)	1010217	Rice, Japonica, Wuyujing3, dehulled, dried, high N fertilization
2805	Rice (Milled Equivalent)	1010218	Rice, Japonica, Wuyujing3, dehulled, dried, high N fertilization
2805	Rice (Milled Equivalent)	1010219	Rice, Japonica, Wuyujing3, dehulled, dried, moderate N fertilization
2805	Rice (Milled Equivalent)	1010220	Rice, Japonica, Wuyujing3, dehulled, dried, moderate N fertilization
2805	Rice (Milled Equivalent)	1010221	Rice, Japonica, Wuyujing3, dehulled, dried, without N fertilization
2805	Rice (Milled Equivalent)	1010222	Rice, Japonica, Xiushui09, dehulled, dried, high N fertilization
2805	Rice (Milled Equivalent)	1010223	Rice, Japonica, Xiushui09, dehulled, dried, high N fertilization
2805	Rice (Milled Equivalent)	1010224	Rice, Japonica, Xiushui09, dehulled, dried, moderate N fertilization
2805	Rice (Milled Equivalent)	1010225	Rice, Japonica, Xiushui09, dehulled, dried, moderate N fertilization
2805	Rice (Milled Equivalent)	1010226	Rice, Japonica, Xiushui09, dehulled, dried, without N fertilization
2805	Rice (Milled Equivalent)	1010227	Rice, Japonica, Xiushui11, dehulled, dried, high N fertilization
2805	Rice (Milled Equivalent)	1010228	Rice, Japonica, Xiushui11, dehulled, dried, high N fertilization

Supplementary Table 4 (continued): NBS food matches for phytate

FBS_itemcode	FBS_itemname	PhyFoodComp1.0 food_item_id	food_name_english
2805	Rice (Milled Equivalent)	1010229	Rice, Japonica, Xiushui11, dehulled, dried, moderate N fertilization
2805	Rice (Milled Equivalent)	1010230	Rice, Japonica, Xiushui11, dehulled, dried, moderate N fertilization
2805	Rice (Milled Equivalent)	1010231	Rice, Japonica, Xiushui11, dehulled, dried, without N fertilization
2805	Rice (Milled Equivalent)	1010232	Rice, Japonica, Xiushui63, dehulled, dried, high N fertilization
2805	Rice (Milled Equivalent)	1010233	Rice, Japonica, Xiushui63, dehulled, dried, high N fertilization
2805	Rice (Milled Equivalent)	1010234	Rice, Japonica, Xiushui63, dehulled, dried, moderate N fertilization
2805	Rice (Milled Equivalent)	1010235	Rice, Japonica, Xiushui63, dehulled, dried, moderate N fertilization
2805	Rice (Milled Equivalent)	1010236	Rice, Japonica, Xiushui63, dehulled, dried, without N fertilization
2805	Rice (Milled Equivalent)	1010237	Rice, Japonica, Xudao3, dehulled, dried, high N fertilization
2805	Rice (Milled Equivalent)	1010238	Rice, Japonica, Xudao3, dehulled, dried, high N fertilization
2805	Rice (Milled Equivalent)	1010239	Rice, Japonica, Xudao3, dehulled, dried, moderate N fertilization
2805	Rice (Milled Equivalent)	1010240	Rice, Japonica, Xudao3, dehulled, dried, moderate N fertilization
2805	Rice (Milled Equivalent)	1010241	Rice, Japonica, Xudao3, dehulled, dried, without N fertilization
2805	Rice (Milled Equivalent)	1010242	Rice, Japonica, Xudao4, dehulled, dried, high N fertilization
2805	Rice (Milled Equivalent)	1010243	Rice, Japonica, Xudao4, dehulled, dried, high N fertilization
2805	Rice (Milled Equivalent)	1010244	Rice, Japonica, Xudao4, dehulled, dried, moderate N fertilization
2805	Rice (Milled Equivalent)	1010245	Rice, Japonica, Xudao4, dehulled, dried, moderate N fertilization
2805	Rice (Milled Equivalent)	1010246	Rice, Japonica, Xudao4, dehulled, dried, without N fertilization
2805	Rice (Milled Equivalent)	1010247	Rice, Japonica, Xudao5, dehulled, dried, high N fertilization
2805	Rice (Milled Equivalent)	1010248	Rice, Japonica, Xudao5, dehulled, dried, high N fertilization
2805	Rice (Milled Equivalent)	1010249	Rice, Japonica, Xudao5, dehulled, dried, moderate N fertilization
2805	Rice (Milled Equivalent)	1010250	Rice, Japonica, Xudao5, dehulled, dried, moderate N fertilization
2805	Rice (Milled Equivalent)	1010251	Rice, Japonica, Xudao5, dehulled, dried, without N fertilization
2805	Rice (Milled Equivalent)	1010252	Rice, Japonica, Yanjing2, dehulled, dried, high N fertilization
2805	Rice (Milled Equivalent)	1010253	Rice, Japonica, Yanjing2, dehulled, dried, high N fertilization
2805	Rice (Milled Equivalent)	1010254	Rice, Japonica, Yanjing2, dehulled, dried, moderate N fertilization
2805	Rice (Milled Equivalent)	1010255	Rice, Japonica, Yanjing2, dehulled, dried, moderate N fertilization
2805	Rice (Milled Equivalent)	1010256	Rice, Japonica, Yanjing2, dehulled, dried, without N fertilization
2805	Rice (Milled Equivalent)	1010257	Rice, Japonica, Yanjing5, dehulled, dried, high N fertilization
2805	Rice (Milled Equivalent)	1010258	Rice, Japonica, Yanjing5, dehulled, dried, high N fertilization
2805	Rice (Milled Equivalent)	1010259	Rice, Japonica, Yanjing5, dehulled, dried, moderate N fertilization
2805	Rice (Milled Equivalent)	1010260	Rice, Japonica, Yanjing5, dehulled, dried, moderate N fertilization
2805	Rice (Milled Equivalent)	1010261	Rice, Japonica, Yanjing5, dehulled, dried, without N fertilization
2805	Rice (Milled Equivalent)	1010262	Rice, Japonica, Yanjing9, dehulled, dried, high N fertilization
2805	Rice (Milled Equivalent)	1010263	Rice, Japonica, Yanjing9, dehulled, dried, high N fertilization
2805	Rice (Milled Equivalent)	1010264	Rice, Japonica, Yanjing9, dehulled, dried, moderate N fertilization
2805	Rice (Milled Equivalent)	1010265	Rice, Japonica, Yanjing9, dehulled, dried, moderate N fertilization
2805	Rice (Milled Equivalent)	1010266	Rice, Japonica, Yanjing9, dehulled, dried, without N fertilization
2805	Rice (Milled Equivalent)	1010267	Rice, Japonica, Zaofeng9, dehulled, dried, high N fertilization
2805	Rice (Milled Equivalent)	1010268	Rice, Japonica, Zaofeng9, dehulled, dried, high N fertilization
2805	Rice (Milled Equivalent)	1010269	Rice, Japonica, Zaofeng9, dehulled, dried, moderate N fertilization
2805	Rice (Milled Equivalent)	1010270	Rice, Japonica, Zaofeng9, dehulled, dried, moderate N fertilization
2805	Rice (Milled Equivalent)	1010271	Rice, Japonica, Zaofeng9, dehulled, dried, without N fertilization
2805	Rice (Milled Equivalent)	1010272	Rice, Japonica, Zhendao10, dehulled, dried, high N fertilization
2805	Rice (Milled Equivalent)	1010273	Rice, Japonica, Zhendao10, dehulled, dried, high N fertilization
2805	Rice (Milled Equivalent)	1010274	Rice, Japonica, Zhendao10, dehulled, dried, moderate N fertilization
2805	Rice (Milled Equivalent)	1010275	Rice, Japonica, Zhendao10, dehulled, dried, moderate N fertilization
2805	Rice (Milled Equivalent)	1010276	Rice, Japonica, Zhendao10, dehulled, dried, without N fertilization
2805	Rice (Milled Equivalent)	1010277	Rice, Japonica, Zhendao88, dehulled, dried, high N fertilization
2805	Rice (Milled Equivalent)	1010278	Rice, Japonica, Zhendao88, dehulled, dried, high N fertilization
2805	Rice (Milled Equivalent)	1010279	Rice, Japonica, Zhendao88, dehulled, dried, moderate N fertilization
2805	Rice (Milled Equivalent)	1010280	Rice, Japonica, Zhendao88, dehulled, dried, moderate N fertilization
2805	Rice (Milled Equivalent)	1010281	Rice, Japonica, Zhendao88, dehulled, dried, without N fertilization
2805	Rice (Milled Equivalent)	1010282	Rice, Japonica, Zhendao99, dehulled, dried, high N fertilization
2805	Rice (Milled Equivalent)	1010283	Rice, Japonica, Zhendao99, dehulled, dried, high N fertilization
2805	Rice (Milled Equivalent)	1010284	Rice, Japonica, Zhendao99, dehulled, dried, moderate N fertilization
2805	Rice (Milled Equivalent)	1010285	Rice, Japonica, Zhendao99, dehulled, dried, moderate N fertilization
2805	Rice (Milled Equivalent)	1010286	Rice, Japonica, Zhendao99, dehulled, dried, without N fertilization
2805	Rice (Milled Equivalent)	1010287	Rice, Junam, conventional, brown, raw
2805	Rice (Milled Equivalent)	1010288	Rice, Ld 355/Bg450, parboiled
2805	Rice (Milled Equivalent)	1010289	Rice, Ld 355/Bg450, raw
2805	Rice (Milled Equivalent)	1010292	Rice, milled, raw
2805	Rice (Milled Equivalent)	1010293	Rice, milled, raw
2805	Rice (Milled Equivalent)	1010294	Rice, milled, raw
2805	Rice (Milled Equivalent)	1010295	Rice, milled, raw
2805	Rice (Milled Equivalent)	1010296	Rice, milled, raw
2805	Rice (Milled Equivalent)	1010297	Rice, milled, raw
2805	Rice (Milled Equivalent)	1010298	Rice, milled, raw
2805	Rice (Milled Equivalent)	1010299	Rice, milled, raw
2805	Rice (Milled Equivalent)	1010304	Rice, Milyang204, genetically modified, brown, raw
2805	Rice (Milled Equivalent)	1010305	Rice, Nakdongbyeo, brown, raw
2805	Rice (Milled Equivalent)	1010308	Rice, Panjin Pearl, raw
2805	Rice (Milled Equivalent)	1010312	Rice, parboiled, milled
2805	Rice (Milled Equivalent)	1010313	Rice, polished, raw, milled
2805	Rice (Milled Equivalent)	1010314	Rice, polished, raw
2805	Rice (Milled Equivalent)	1010317	Rice, raw
2805	Rice (Milled Equivalent)	1010318	Rice, raw
2805	Rice (Milled Equivalent)	1010319	Rice, raw
2805	Rice (Milled Equivalent)	1010320	Rice, RD-6, raw
2805	Rice (Milled Equivalent)	1010322	Rice, RD-8, brown, raw, dehulled
2805	Rice (Milled Equivalent)	1010328	Rice, red, raw
2805	Rice (Milled Equivalent)	1010332	Rice, Thailand, raw
2805	Rice (Milled Equivalent)	1010333	Rice, Tianjin Xiao Zhan, raw
2805	Rice (Milled Equivalent)	1010334	Rice, unpolished, raw
2805	Rice (Milled Equivalent)	1010337	Rice, white, long grains, raw
2805	Rice (Milled Equivalent)	1010338	Rice, white, Manischewitz's brand (kosher), raw
2805	Rice (Milled Equivalent)	1010339	Rice, Calrose, white, raw
2805	Rice (Milled Equivalent)	1010340	Rice, white, raw
2805	Rice (Milled Equivalent)	1010341	Rice, white, short grains, raw
2805	Rice (Milled Equivalent)	1010342	Rice, white, sunned, aromatic
2805	Rice (Milled Equivalent)	1010343	Rice, white, sunned, polished, milled
2805	Rice (Milled Equivalent)	1010345	Rice, whole grain, raw
2848	Milk - Excluding Butter	11010002	Milk, cow, raw

Supplementary Table 4 (continued): NBS food matches for phytate

FBS_itemcode	FBS_itemname	PhyFoodComp1.0 food_item_id	food_name_english
**Flour**			
Whole			
2511	Wheat and products	1030185	Wheat flour, atta
2511	Wheat and products	1030209	Wheat flour, whole grain
2511	Wheat and products	1030210	Wheat flour, whole grain
2511	Wheat and products	1030211	Wheat flour, whole grain
2511	Wheat and products	1030212	Wheat flour, whole grain
2511	Wheat and products	1030213	Wheat flour, whole grain
2511	Wheat and products	1030214	Wheat flour, whole grain
2511	Wheat and products	1030215	Wheat flour, whole grain
2511	Wheat and products	1030216	Wheat flour, whole grain, Pillsbury
2511	Wheat and products	1030217	Wheat flour, whole meal
Refined			
2511	Wheat and products	1030168	Wheat flour
2511	Wheat and products	1030169	Wheat flour
2511	Wheat and products	1030170	Wheat flour
2511	Wheat and products	1030171	Wheat flour
2511	Wheat and products	1030172	Wheat flour
2511	Wheat and products	1030173	Wheat flour
2511	Wheat and products	1030174	Wheat flour
2511	Wheat and products	1030175	Wheat flour
2511	Wheat and products	1030176	Wheat flour
2511	Wheat and products	1030181	Wheat flour, 50% extraction
2511	Wheat and products	1030182	Wheat flour, 75% extraction
2511	Wheat and products	1030183	Wheat flour, 85% extraction
2511	Wheat and products	1030184	Wheat flour, all-purpose, General Mills
2511	Wheat and products	1030186	Wheat flour, Back Cross of Roshan
2511	Wheat and products	1030187	Wheat flour, Back Cross of Roshan, hydrothermaled
2511	Wheat and products	1030191	Wheat flour, Tajan
2511	Wheat and products	1030192	Wheat flour, Tajan, hydrothermaled
2511	Wheat and products	1030193	Wheat flour, type 500-1
2511	Wheat and products	1030194	Wheat flour, type 500-2
2511	Wheat and products	1030196	Wheat flour, type for leavened pastry
2511	Wheat and products	1030201	Wheat flour, white
2511	Wheat and products	1030202	Wheat flour, white
2511	Wheat and products	1030203	Wheat flour, white
2511	Wheat and products	1030204	Wheat flour, white
2511	Wheat and products	1030205	Wheat flour, white
2511	Wheat and products	1030206	Wheat flour, white
2511	Wheat and products	1030207	Wheat flour, white, packaged
2511	Wheat and products	1030208	Wheat flour, white, refined
2511	Wheat and products	1030219	Wheat, baking flour
Whole/refined			
2514	Maize and products	1020060	Maize flour, 65% extraction
Whole/refined			
2517	Millet and products	1050076	Finger millet flour
2517	Millet and products	1050100	Foxtail millet flour
2517	Millet and products	1050101	Foxtail millet flour
2517	Millet and products	1050123	Pearl millet flour, Ashana, dehulled seed
2517	Millet and products	1050124	Pearl millet flour, Ashana, dehulled seed, 30 days storage
2517	Millet and products	1050125	Pearl millet flour, Ashana, dehulled seed, 60 days storage
2517	Millet and products	1050129	Pearl millet flour, Ashana, dehulled seed, irradiated
2517	Millet and products	1050130	Pearl millet flour, Ashana, dehulled seed, irradiated, 30 days storage
2517	Millet and products	1050131	Pearl millet flour, Ashana, dehulled seed, irradiated, 60 days storage
2517	Millet and products	1050135	Pearl millet flour, Ashana, whole seed
2517	Millet and products	1050136	Pearl millet flour, Ashana, whole seed, 30 days storage
2517	Millet and products	1050137	Pearl millet flour, Ashana, whole seed, 60 days storage
2517	Millet and products	1050141	Pearl millet flour, Ashana, whole seed, raw, irradiated
2517	Millet and products	1050142	Pearl millet flour, Ashana, whole seed, irradiated, 30 days storage
2517	Millet and products	1050143	Pearl millet flour, Ashana, whole seed, irradiated, 60 days storage
2517	Millet and products	1050147	Pearl millet flour, Dembi, dehulled seed
2517	Millet and products	1050148	Pearl millet flour, Dembi, dehulled seed, 30 days storage
2517	Millet and products	1050149	Pearl millet flour, Dembi, dehulled seed, 60 days storage
2517	Millet and products	1050153	Pearl millet flour, Dembi, dehulled seed, irradiated
2517	Millet and products	1050154	Pearl millet flour, Dembi, dehulled seed, irradiated, 60 days storage
2517	Millet and products	1050158	Pearl millet flour, Dembi, dehulled seed,irradiated, 30 days storage
2517	Millet and products	1050159	Pearl millet flour, Dembi, whole seed
2517	Millet and products	1050160	Pearl millet flour, Dembi, whole seed, 30 days storage
2517	Millet and products	1050161	Pearl millet flour, Dembi, whole seed, 60 days storage
2517	Millet and products	1050165	Pearl millet flour, Dembi, whole seed, raw, irradiated
2517	Millet and products	1050166	Pearl millet flour, Dembi, whole seed, irradiated, 30 days storage
2517	Millet and products	1050167	Pearl millet flour, Dembi, whole seed, irradiated, 60 days storage
2517	Millet and products	1050171	Pearl millet flour, HHB-67
2517	Millet and products	1050172	Pearl millet flour, IS 833
2517	Millet and products	1050173	Pearl millet flour, IS 843
2517	Millet and products	1050174	Pearl millet flour, IS 880004
2517	Millet and products	1050175	Pearl millet flour, IS 89111
2517	Millet and products	1050176	Pearl millet flour, IS 91333
2517	Millet and products	1050177	Pearl millet flour, IS 91666
2517	Millet and products	1050178	Pearl millet flour, IS 91777
2517	Millet and products	1050179	Pearl millet flour, Kabti
2517	Millet and products	1050180	Pearl millet flour, Tihama
2517	Millet and products	1050181	Pearl millet flour, YD-X3
Whole/refined			
2518	Sorghum and products	1040013	Sorghum flour
2518	Sorghum and products	1040015	Sorghum flour, Dabar, whole grain, 10KGy irradiated, dried
2518	Sorghum and products	1040019	Sorghum flour, Dabar, whole grain, 15KGy irradiated, dried
2518	Sorghum and products	1040023	Sorghum flour, Dabar, whole grain, 5KGy irradiated, dried
2518	Sorghum and products	1040027	Sorghum flour, Dabar, whole grain, dried
2518	Sorghum and products	1040030	Sorghum flour, ground-dried
2518	Sorghum and products	1040032	Sorghum flour, Karamaka, whole grain, 10KGy irradiated, dried
2518	Sorghum and products	1040036	Sorghum flour, Karamaka, whole grain, 15KGy irradiated, dried
2518	Sorghum and products	1040039	Sorghum flour, Karamaka, whole grain, 5KGy irradiated, dried
2518	Sorghum and products	1040044	Sorghum flour, Karamaka, whole grain, dried
2518	Sorghum and products	1040047	Sorghum flour, Tabat
2518	Sorghum and products	1040049	Sorghum flour, Wad Ahmed, whole grain, 10KGy irradiated, dried
2518	Sorghum and products	1040053	Sorghum flour, Wad Ahmed, whole grain, 15KGy irradiated, dried
2518	Sorghum and products	1040057	Sorghum flour, Wad Ahmed, whole grain, 5KGy irradiated, dried
2518	Sorghum and products	1040061	Sorghum flour, Wad Ahmed, whole grain, dried

Supplementary Table 4 (continued): NBS food matches for phytate

FBS_itemcode	FBS_itemname	PhyFoodComp1.0 food_item_id	food_name_english
No matches- assumed to be 0			
2543	Sweeteners, Other		
2563	Olives (including preserved)		
2571	Soyabean Oil		
2573	Sunflowerseed Oil		
2574	Rape and Mustard Oil		
2575	Cottonseed Oil		
2576	Palmkernel Oil		
2578	Coconut Oil		
2579	Sesameseed Oil		
2580	Olive Oil		
2581	Ricebran Oil		
2582	Maize Germ Oil		
2586	Oilcrops Oil, Other		
2612	Lemons, Limes and products		
2613	Grapefruit and products		
2655	Wine		
2656	Beer		
2657	Beverages, Fermented		
2658	Beverages, Alcoholic		
2659	Alcohol, Non-Food		
2680	Infant food		
2731	Bovine Meat		
2732	Mutton & Goat Meat		
2733	Pigmeat		
2734	Poultry Meat		
2735	Meat, Other		
2736	Offals, Edible		
2737	Fats, Animals, Raw		
2740	Butter, Ghee		
2743	Cream		
2744	Eggs		
2745	Honey		
2762	Demersal Fish		
2765	Crustaceans		
2766	Cephalopods		
2767	Molluscs, Other		
2768	Meat, Aquatic Mammals		
2769	Aquatic Animals, Others		
2781	Fish, Body Oil		
2782	Fish, Liver Oil		

Supplementary Formula 1: Expression of FBS elements as daily per capita quantities

For each FBS element i of FBS category j in country k and year l,

PCE_{ijkl} =

$$(E_{ijkl} * G) / (P_{kl} * D) \quad \textbf{(1)}$$

where PCE = per capita element (g/person·day), E = element (1000 MT), G is a conversion factor (1000000 g/MT), P = population (1000 persons) and D is a conversion factor (365 days/year). Values of 0 for FBS data appear either as 0 or as missing in the database. Therefore, all calculated PCE_{ijk} values in the NBS appearing as missing were replaced with 0 in preparation for calculating nutrient values, to distinguish null food values from missing values attributable to the absence of a particular nutrient supplied in the food.

Supplementary Formula 2: Per capita nutrient quantities of FBS elements

For each nutrient h and element i associated with FBS category j in country k and year l,

$PCNE_{hijkl}$ =

$$(PCE_{ijkl} * (1-(R_j / 100)) * C_j * (FCT_{hj} / 100) \quad \textbf{(2)}$$

where PCNE = per capita nutrient of [element] (units/person·day), PCE = per capita [element] (g/person·day), R = refuse percentage (%), C = conversion factor (g modified form / g primary commodity), and FCT is the quantity of nutrient in 100 grams of edible portion ([units]/100g edible portion). A second FCT variable called FCT_ALT containing alternative values for vitamin A in sweet potato was used for all but high-income countries as well as the former countries Ethiopia PDR, Serbia and Montenegro, Sudan (former), USSR, and Yugoslav SFR.

Supplementary Formula 3: Import ratio

For each FBS category j in country k and year l,

IMP_RAT_{jkl} =

$$(PCI_{jkl}/(PCP_{jkl} + PCI_{jkl} - PCEX_{jkl})) \quad \textbf{(3)}$$

where IMP_RAT = the import ratio (unitless), PCP = per capita production (g/person·day), PCI = per capita imports (g/person·day), and PCEX = per capita exports (g/person·day).

Supplementary Formula 4: Production ratio

For each FBS category j in country k and year l,

$PROD\_RAT_{jkl}$ =

$$((PCP_{jkl} - PCEX_{jkl})/(PCP_{ijkl} + PCI_{jkl} - PCEX_{jkl})) \quad \textbf{(4)}$$

Supplementary Formula 5a: Vitamin A from palm oil production in SSA

For each nutrient h and element i associated with FBS category j in country k and year l,

$PCNE_{hijkl}$ =

$(PCE_{ijkl} * 0.3 * (1-(R_j / 100)) * C_j * (FCT_{hj} / 100)) + (PCE_{ijkl} * 0.7 * (1-(R_j / 100)) * C_j * (FCT\_ALT_{hj} / 100))$ **(5a)**

where nutrient h is restricted to Vitamin A, element i is restricted to Production, category j is restricted to Palm Oil, and country k is restricted to countries in SSA, PCNE = per capita vitamin A from production (mcg/person·day), PCE = per capita production (g/person·day), R = refuse percentage (%), C = conversion factor for palm oil (g modified form / g primary commodity), FCT is the quantity of vitamin A in refined palm oil (mcg/100g edible portion) and FCT_ALT is the quantity of vitamin A in red palm oil (mcg/100g edible portion). 70% of retained production was assumed to be consumed as red palm oil.

Supplementary Formula 5b: Vitamin A from palm oil for non-production variables in SSA

For each nutrient h and element i associated with FBS category j in country k and year l,

$PCNE_{hijkl} =$

$(PCE_{ijkl} * IMP\_RAT_{jkl} * (1-(R_j / 100)) * C_j * (FCT_{hj} / 100)) + (PCE_{ijkl} * PROD\_RAT_{jkl} * 0.3 * (1-(R_j / 100)) * C_j * (FCT_{hj} / 100)) + (PCE_{ijkl} * PROD\_RAT_{jkl} * 0.7 * (1-(R_j / 100)) * C_j * (FCT\_ALT_{hj} / 100))$ **(5b)**

where nutrient h is restricted to Vitamin A, element i is restricted to Stocks, Domestic Supply, Seed, Feed, Processing, Losses, Other and Food, category j is restricted to Palm Oil, country k is restricted to countries in SSA, PCNE = per capita vitamin A from [element] (mcg/person·day), PCE = per capita [element] (g/person·day), IMP_RAT = the import ratio (unitless), PROD_RAT = the production ratio (unitless), R = refuse percentage (%), C = conversion factor for palm oil (g modified form / g primary commodity), FCT is the quantity of vitamin A in refined palm oil (mcg/100g edible portion) and FCT_ALT is the quantity of vitamin A in red palm oil (mcg/100g edible portion). The vitamin A compositions of stocks, domestic supply, feed, seed, manufacturing, other, waste and food were calculated based on the production ratio where 70% of production is consumed as red palm oil.

Supplementary Formula 6a: Nutrients net of FLW and cooking loss

For each nutrient h and element i (Food only) associated with FBS category j (except fish and seafood) in country k and year l,

$PCNE_{hijkl} =$

$(PCE_{ijkl} * (1-(R_j / 100)) * C_j * (FCT_{hj} / 100) * (RET_{hj}/100)*(1-((DWASTE_{jk}+CWASTE_{jk})/100))$ **(6a)**

where element i is restricted to Food, category j is restricted to all but those for fish and seafood, PCNE = per capita nutrient of [element] (units/person·day), PCE = per capita [element] (g/person·day), R = refuse percentage (%), C = conversion factor (g modified form / g primary commodity), FCT is the quantity of nutrient in 100 grams of edible portion ([units]/100g edible portion), RET is the percent nutrient retention after food/cooking preparation (%), DWASTE is the percent food waste from retail and distribution (%) and CWASTE is the percent food waste

from household consumption (%). A second FCT variable called FCT_ALT containing alternative values for vitamin A in sweet potato was used for all but high-income countries as well as the former countries Ethiopia PDR, Serbia and Montenegro, Sudan (former), USSR, and Yugoslav SFR.

Supplementary Formula 6b: Nutrients net of FLW and cooking loss for seafood

For each nutrient h and element i associated with FBS category j (fish and seafood only) in country k and year l,

$PCNE_{hijkl}$ =

$$(PCE_{ijkl} * (1-(R_j / 100)) * C_j * (FCT_{hj} / 100)*(RET_{hj}/100)*(1-((PHLOSS_{jk} + DWASTE_{jk} + CWASTE_{jk})/100)) \quad \textbf{(6b)}$$

where element i is restricted to Food, category j is restricted those for fish and seafood, PCNE = per capita nutrient of [element] (units/person·day), PCE = per capita [element] (g/person·day) and is restricted to Food only, R = refuse percentage (%), C = conversion factor (g modified form / g primary commodity), FCT is the quantity of nutrient in 100 grams of edible portion ([units]/100g edible portion) RET is the percent nutrient retention after food/cooking preparation (%), PHLOSS is the post-harvest loss (%), DWASTE is the percent food waste from retail and distribution (%) and CWASTE is the percent food waste from household consumption (%).

Supplementary Formula 6c: Vitamin A from palm oil in SSA net of FLW and cooking

For each nutrient h and element i associated with FBS category j in country k and year l,

$PCNE_{hijkl}$ =

$$(PCE_{ijkl} * IMP\_RAT_{jkl} * (1-(R_j / 100)) * C_j * (FCT_{hj} / 100) * (RET_{hj}/100) * (1-((DWASTE_{jk}+CWASTE_{jk})/100))) + (PCE_{ijkl} * PROD\_RAT_{jkl} * 0.3 * (1-(R_j / 100)) * C_j * (FCT_{hj} / 100) * (RET_{hj}/100) * (1-((DWASTE_{jk}+CWASTE_{jk})/100))) + (PCE_{ijkl} * PROD\_RAT_{jkl} * 0.7 * (1-(R_j / 100)) * C_j * (FCT\_ALT_{hj} / 100) * (RET_{hj}/100) * (1-((DWASTE_{jk}+CWASTE_{jk})/100))) \quad \textbf{(6c)}$$

where nutrient h is restricted to Vitamin A, element i is restricted Food, category j is restricted to Palm Oil, country k is restricted to countries in SSA, PCNE = per capita vitamin A from [element] (mcg/person·day), PCE = per capita [element] (g/person·day), IMP_RAT = the import ratio (unitless), PROD_RAT = the production ratio (unitless), R = refuse percentage (%), C = conversion factor for palm oil (g modified form / g primary commodity), FCT is the quantity of vitamin A in refined palm oil (mcg/100g edible portion), FCT_ALT is the quantity of vitamin A in red palm oil (mcg/100g edible portion), RET is the percent nutrient retention after food/cooking preparation (%), DWASTE is the percent food waste from retail and distribution (%) and CWASTE is the percent food waste from household consumption (%).

Supplementary Formula 7a: Nutrients from wheat and wheat flour

For each nutrient h and element i (Food only) associated with FBS category j in country k and year l,

$PCNE_{hijkl}$ =

$(PCWF_{ijkl} * (1-(RF1_j / 100)) * CF1_j * (FCT\_F1_{hj} / 100)) + (PCWWF_{ijkl} * (1-(RF2_j / 100)) * CF2_j * (FCT\_F2_{hj} / 100))$ **(7a)**

where element i is restricted to Food, FBS category j is restricted to Wheat, PCNE = per capita nutrient of [element] (units/person·day), PCWF = per capita white flour availability (g/person·day), PCWWF = per capita whole wheat flour availability (g/person·day), RF1 = refuse percentage for white wheat flour (%), RF2 = refuse percentage for whole wheat flours (%), CF1 = conversion factor for white wheat flour (g modified form / g primary commodity), CF2 = conversion factor for whole wheat flour (g modified form / g primary commodity), FCT_F1 is the quantity of nutrient in 100 grams of edible portion ([units]/100g edible portion) for white wheat flour, and FCT_F2 is the quantity of nutrient in 100 grams of edible portion ([units]/100g edible portion) for whole wheat flour.

Supplementary Formula 7b: Nutrients from maize, millet, sorghum and flours

For each nutrient h and element i (Food only) associated with FBS category j in country k and year l,

$PCNE_{hijkl}$ =

$(PCF_{ijkl} * (1-(RF1_j / 100)) * CF1_j * (FCT\_F1_{hj} / 100)) + (PCG_{ijkl} * (1-(R_j / 100)) * C_j * (FCT_{hj} / 100))$ **(7b)**

where element i is restricted to Food, FBS category j is restricted to Maize, Millet and Sorghum, PCNE = per capita nutrient of [element] (units/person·day), PCF = per capita flour availability (g/person·day), PCG = per capita grain availability (g/person·day), R = refuse percentage for grain (%), RF1 = refuse percentage for flour (%), C = conversion factor for grain (g modified form / g primary commodity), CF1 = conversion factor for flour (g modified form / g primary commodity), FCT is the quantity of nutrient in 100 grams of edible portion ([units]/100g edible portion) for grain and FCT_F1 is the quantity of nutrient in 100 grams of edible portion ([units]/100g edible portion) for flour.

Supplementary Formula 7c: Nutrients from maize and masa harina

For each nutrient h and element i (Food only) associated with FBS category j in country k and year l,

$PCNE_{hijkl}$ =

$(PCF_{ijkl} * (1-(RF2_j / 100)) * CF2_j * (FCT\_F2_{hj} / 100)) + (PCG_{ijkl} * (1-(R_j / 100)) * C_j * (FCT_{hj} / 100))$ **(7c)**

where element i is restricted to Food, FBS category j is restricted to Maize, FBS country k is limited to those in Central America, PCNE = per capita nutrient of [element] (units/person·day), PCF = per capita masa flour availability (g/person·day), PCG = per capita maize grain availability (g/person·day), R = refuse percentage for maize grain (%), RF2 = refuse percentage for masa harina (%), C = conversion factor for masa harina (g modified form / g primary

commodity), CF2 = conversion factor for masa harina (g modified form / g primary commodity), FCT is the quantity of nutrient in 100 grams of edible portion ([units]/100g edible portion) for maize grain and FCT_F2 is the quantity of nutrient in 100 grams of edible portion ([units]/100g edible portion) for masa harina.

Supplementary Formula 8a: Nutrients from wheat and flour net of FLW and cooking loss

For each nutrient h and element i (Food only) associated with FBS category j in country k and year l,

$PCNE_{hijkl}$ =

$$(PCWF_{ijkl} * (1-(RF1_j / 100)) * CF1_j * (FCT\_F1_{hj} / 100) * (RET\_FL_{hj}/100)*(1-((DWASTE_{jk}+CWASTE_{jk})/100))) + (PCWWF_{ijkl} * (1-(RF2_j / 100)) * CF2_j * (FCT\_F2_{hj} / 100) * (RET\_FL_{hj}/100)*(1-((DWASTE_{jk}+CWASTE_{jk})/100))) \quad \textbf{(8a)}$$

where element i is restricted to Food, FBS category j is restricted to Wheat, PCNE = per capita nutrient of [element] (units/person·day), PCWF = per capita white flour availability (g/person·day), PCWWF = per capita whole wheat flour availability (g/person·day), RF1 = refuse percentage for white wheat flour (%), RF2 = refuse percentage for whole wheat flours (%), CF1 = conversion factor for white wheat flour (g modified form / g primary commodity), CF2 = conversion factor for whole wheat flour (g modified form / g primary commodity), FCT_F1 is the quantity of nutrient in 100 grams of edible portion ([units]/100g edible portion) for white wheat flour, FCT_F2 is the quantity of nutrient in 100 grams of edible portion ([units]/100g edible portion) for whole wheat flour, RET_FL is the percent nutrient retention after food/cooking preparation (%) for flour, DWASTE is the percent food waste from retail and distribution (%) and CWASTE is the percent food waste from household consumption (%).

Supplementary Formula 8b: Nutrients from maize, millet, sorghum and flours net of FLW and cooking loss

For each nutrient h and element i (Food only) associated with FBS category j in country k and year l,

$PCNE_{hijkl}$ =

$$(PCF_{ijkl} * (1-(RF1_j / 100)) * CF1_j * (FCT\_F1_{hj} / 100) * (RET\_FL_{hj}/100)*(1-((DWASTE_{jk}+CWASTE_{jk})/100))) + (PCG_{ijkl} * (1-(R_j / 100)) * C_j * (FCT_{hj} / 100) * (RET_{hj}/100)*(1-((DWASTE_{jk}+CWASTE_{jk})/100))) \quad \textbf{(8b)}$$

where element i is restricted to Food, FBS category j is restricted to Maize, Millet and Sorghum, PCNE = per capita nutrient of [element] (units/person·day), PCF = per capita flour availability (g/person·day), PCG = per capita grain availability (g/person·day), R = refuse percentage for grain (%), RF1 = refuse percentage for flour (%), C = conversion factor for grain (g modified form / g primary commodity), CF1 = conversion factor for flour (g modified form / g primary commodity), FCT is the quantity of nutrient in 100 grams of edible portion ([units]/100g edible portion) for grain and FCT_F1 is the quantity of nutrient in 100 grams of edible portion ([units]/100g edible portion) for flour, RET is the percent nutrient retention after food/cooking

preparation (%) for grain, RET_FL is the percent nutrient retention after food/cooking preparation (%) for flour, DWASTE is the percent food waste from retail and distribution (%) and CWASTE is the percent food waste from household consumption (%).

Supplementary Formula 8c: Nutrients from maize and masa harina net of FLW and cooking loss

For each nutrient h and element i (Food only) associated with FBS category j in country k and year l,

$PCNE_{hijkl}$ =

$$(PCF_{ijkl} * (1-(RF2_j / 100)) * CF2_j * (FCT\_F2_{hj} / 100) * (RET\_FL_{hj}/100)*(1-((DWASTE_{jk}+CWASTE_{jk})/100))) + (PCG_{ijkl} * (1-(R_j / 100)) * C_j * (FCT_{hj} / 100) * (RET_{hj}/100)*(1-((DWASTE_{jk}+CWASTE_{jk})/100))) \quad \textbf{(8c)}$$

where element i is restricted to Food, FBS category j is restricted to Maize, FBS country k is limited to those in Central America, PCNE = per capita nutrient of [element] (units/person·day), PCF = per capita masa flour availability (g/person·day), PCG = per capita maize grain availability (g/person·day), R = refuse percentage for maize grain (%), RF2 = refuse percentage for masa harina (%), C = conversion factor for masa harina (g modified form / g primary commodity), CF2 = conversion factor for masa harina (g modified form / g primary commodity), FCT is the quantity of nutrient in 100 grams of edible portion ([units]/100g edible portion) for maize grain and FCT_F2 is the quantity of nutrient in 100 grams of edible portion ([units]/100g edible portion) for masa harina, RET is the percent nutrient retention after food/cooking preparation (%) for grain, RET_FL is the percent nutrient retention after food/cooking preparation (%) for flour, DWASTE is the percent food waste from retail and distribution (%) and CWASTE is the percent food waste from household consumption (%).

Supplementary Formula 9: Per capita heme iron

For each FBS category j in country k and year l:

$PCHFE_{jkl}$ =

$$(PCFE_{jkl} * HFEP_j) \quad \textbf{(9)}$$

where PCHFE = per capita heme iron (mg/person·day), PCFE = per capita iron (mg/person·day) and HFEP = heme iron proportion (equal to 0.4 for heme-containing foods and 0 for non-heme foods).

Supplementary Formula 10: Per capita nonheme iron

$PCNHFE_{jkl}$ =

$$(PCFE_{jkl} * NHFEP_j) \quad \textbf{(10)}$$

where PCNHFE = per capita non-heme iron (mg/person · day), PCFE = per capita iron (mg/person · day), and NHFEP = non-heme iron proportion (equal to 0.6 for heme-containing foods and 1.0 for non-heme foods).

Supplementary Formula 11: Log percent nonheme iron availability using Conway algorithm

For each country k in year l:

$LPNHFE_{kl}$ =

$$0.9385 + (0.0010 * VCFruits_{kl}) + (0.0009 * AT_{kl}) - (0.0052 * BeanLntl_{kl}) - (0.0016 * WhlGrn_{kl}) - (0.0020 * Tea_{kl}) - (0.0012 * Dairy_{kl}) - (0.0032 * Eggs_{kl}) - (0.0062 * Soya_{kl}) - (0.0087 * Nuts_{kl}) \quad \mathbf{(11)}$$

where LPNHFE = $\log_{10}$ percent non-heme iron availability (%), VCFruits = high vitamin C fruit (g/person · day), AT = animal tissue (g/person · day), BeanLntl = beans and lentils (g/person · day), WhlGrn = wholegrain cereals (g/person · day), Tea = black tea infusion equivalents (g/person · day), Dairy = all dairy (g/person · day), Eggs = any eggs (g/person · day), Soya = soya beans (g/person · day), and Nuts = any nuts (g/person · day). The percent of non-heme iron bioavailability was determined by taking the antilog of $LPNHFE_{kl}$.

Supplementary Formula 12: Per capita bioavailable iron based on Conway algorithm

For each food category j in country k in year l:

$PCBVFEC_{kl}$ =

$$(PCHFE_{jkl} * 0.25) + (PCNHFE_{jkl} * (PNHFEC_{kl}/100)) \quad \mathbf{(12)}$$

where PCBVFEC = per capita bioavailable iron using the Conway algorithm (mg/person · day), PCHFE = per capita heme iron (mg/person · day), PCNHFE = per capita non-heme iron (mg/person · day), and PNHFEC = percent non-heme iron availability using the Conway algorithm (%).

Supplementary Formula 13: Percent nonheme iron availability using Rickard (2009) algorithm

For each country k in year l:

$PNHFER_{kl}$ =

$$22.42 * [(1 + \ln(1 + 0.0056 * AA_{kl})) * (1 + \ln(1 + 0.0008 * AT_{kl}))] / [[(1 + \ln(1 + 0.0008 * C_{kl})) * (1 + \ln(1 + 0.0033 * P_{kl})) * (1 + \ln(1 + 0.0004 * P_{kl}))) * (1 + \ln(1 + 0.0424 * NH_{kl}))] \quad \mathbf{(13)}$$

where PNHFER = percent non-heme iron availability using the Rickard algorithm (%), AA = ascorbic acid (mg/person · day), AT = animal tissue (g/person · day), C = calcium (mg/person · day), P = phytate (mg/person · day), PO = polyphenol from tea (mg), NH = non-heme iron (mg/person · day), and HI = heme iron (mg/person · day).

Supplementary Formula 14: Per capita bioavailable iron based on Rickard (2009) algorithm

For each food category j in country k in year l:

$PCBVFER_{kl}$ =

$$(PCHFE_{jkl} * 0.25) + (PCNHFE_{jkl} * (PNHFER_{kl}/100)) \quad \textbf{(14)}$$

where PCBVFER = per capita bioavailable iron using the Rickard algorithm (mg/person · day), PCHFE = per capita heme iron (mg/person · day), PCNHFE = per capita non-heme iron (mg/person · day), and PNHFER = percent non-heme iron availability using the Rickard algorithm (%).

Supplementary Formula 15: Log percent nonheme iron availability using Armah (2013) algorithm

For each country k in year l:

$LPNHFEA_{kl}$ =

$$6.294 – 0.709 * \ln(SF_{kl}) + 0.119 * \ln(C_{kl}) + 0.006 * \ln(MFP_{kl} + 1) – (0.055 * \ln(T_{kl} + 1) – 0.247 * \ln(P_{kl}) – 0.137 * \ln(Ca_{kl}) = 0.083 * \ln(NH_{kl}) \quad \textbf{(15)}$$

where LPNHFEA = natural log percent non-heme iron availability using the Armah algorithm (%), SF = serum ferritin (mcg/L), C = vitamin C (mg/person · day), MFP = meat, fish and poultry (g/person · day), T = tea (number of cups/person · day), P = phytate (mg/person · day), Ca = calcium (mg/person · day), NH = non-heme iron (mg/person · day). The percent non-heme iron bioavailability was determined by taking the antilog of $LPNHFEA_{kl.}$ A constant value for serum ferritin corresponding to a reference dose absorption of 40% (21.7 mcg/L of serum ferritin) was used to adapt the algorithm to predict the amount of available non-heme iron at a constant level of serum ferritin.

Supplementary Formula 16: Per capita iron absorption based on Armah (2013) algorithm

For each food category j in country k in year l:

$PCBVFEA_{kl}$ =

$$(PCHFE_{jkl} * 0.25) + (PCNHFE_{jkl} * (PNHFEA_{kl}/100)) \quad \textbf{(16)}$$

where PCBVFEA = per capita iron absorption using the Armah algorithm (mg/person · day), PCHFE = per capita heme iron (mg/person · day), PCNHFE = per capita non-heme iron (mg/person · day), and PNHFEA = percent non-heme iron absorption using the Armah algorithm (%).

Supplementary Formula 17: Total per capita absorbed zinc using Hambidge (2010) algorithm

For each country k in year l:

$$TAZ_{kl} = 0.5 * [Amax + TDZ + K_R *(1 + TDP/K_P) – \sqrt{} (A_{max} + TDZ + K_R * (1 + TDP/K_P))^2 – 4 * A_{max} * TDZ)] \quad \textbf{(17)}$$

where TAZ = total absorbable zinc (mmol/person · day), TDZ = total dietary zinc (mmol/person · day), TDP = total dietary phytate (mg/person · day), A_{max} (maximum absorbed zinc) = 0.091, K_R (Equilibrium dissociation constant of the zinc-receptor binding) = 0.033, and K_P (Equilibrium dissociation constant of the zinc-phytate binding) = 0.68. TAZ was converted to milligrams using the ratio 1mmol zinc = 65.38 mg.

Supplementary Formula 18: Number of pregnant women

For each age group j in country k and year l:

$PRGWMN_{jkl}$ =

$$WRA_{jkl}/1000 * \{(ASFR_{jkl} * 0.75) + (ABRT_k * 0.167) + ((ASFR_{jkl} * 0.20) + (ABRT_k * 0.10) * 0.25)\} \quad \textbf{(18)}$$

where PRGWMN = number of pregnant women, ASFR = age-specific fertility rate (births per 1000 women), and ABRT = the induced abortion rate (induced abortions per 1000 women). The number of miscarriages were calculated as 20% of live births plus 10% of induced abortions (Sedgh et al., 2014). The following durations specified in the CDC formula were also applied: births = 9 months or 0.75 year; induced abortions = 2 months or 0.167 year; and miscarriages = 3 months or 0.25 year (CDC, 2016).

Supplementary Formula 19: Number of lactating women

For each age group j in country k and year l:

$LACWMN_{jkl}$ =

$$WRA_{jkl}/1000 * ASFR_{jkl} * 0.50 \quad \textbf{(19)}$$

where LACWMN = number of lactating women and ASFR = age-specific fertility rate (births per 1000 women).

Supplementary Formula 20: Total daily nutrient requirements by demographic stratum

For each micronutrient h, sex and pregnancy/lactation group i in age group j in country k and year l,

$NREQPOP_{hijkl}$ =

$$POP_{ijkl} * REQ_{hij} \qquad \textbf{(20)}$$

where NREQPOP = total daily group-level nutrient requirements (1000 [units]/day), POP = population size (1000 persons), and REQ = AR or IA ([units]/person · day).

Supplementary Formula 21: Total daily national nutrient requirements

For each nutrient h, sex and pregnancy/lactation group i in age group j in country k and year l,

$NREQCNTRY_{hkl}$ =

$$\sum_{i=1}^{n} \sum_{j=1}^{n} NREQPOP_{hijkl} \qquad \textbf{(21)}$$

where NREQCNTRY = total daily national nutrient requirements (1000 [units]/day), and NREQPOP = total daily group-level nutrient requirements (1000 [units]/day).

Supplementary Formula 22: Total daily national per capita nutrient requirements

For each nutrient h, in country k and year l,

$PCREQCNTRY_{hkl}$ =

$$NREQCNTRY_{hkl} / POP_{kl} \qquad \textbf{(22)}$$

where PCREQCNTRY = daily national per capita nutrient requirements ([units]/person * day) and NREQCNTRY = total daily national nutrient requirements (1000 [units]/day).
