# Supplementary material for: Tracing global flows of bioactive compounds from farm to fork in Nutrient Balance Sheets can help guide intervention towards healthier food supplies
Source: Nat Food. Author manuscript; Available in PMC 2022 Oct 11. (PMC7613697; doi:10.1038/s43016-022-00585-w)
Supplement: Supplementary Table 1 [file EMS153904-supplement-Supplementary_Table_1.docx]

| **Num** | **Nutrient** | **Unit** | **Num** | **Nutrient** | **Unit** |
| --- | --- | --- | --- | --- | --- |
| **1** | Energy | kcal | **20** | Vitamin D (D2+D3) | mcg |
| **2** | Protein | g | **21** | Vitamin C, total ascorbic acid | mg |
| **3** | Total lipid or fat | g | **22** | Thiamin | mg |
| **4** | Carbohydrate by difference | g | **23** | Riboflavin | mg |
| **5** | Total dietary fiber | g | **24** | Niacin | mg |
| **6** | Calcium, Ca | mg | **25** | Pantothenic acid | mg |
| **7** | Iron, Fe | mg | **26** | Vitamin B6 | mg |
| **8** | Iron, heme, HFe | mg | **27** | Vitamin B12 | mcg |
| **9** | Iron, non-heme, NHFe | mg | **28** | Choline, total | mg |
| **10** | Magnesium, Mg | mg | **29** | Vitamin K (phylloquinone) | mcg |
| **11** | Phosphorous, P | mg | **30** | Folate, food | mcg |
| **12** | Potassium, K | mg | **31** | Cholesterol | mg |
| **13** | Sodium, Na | mg | **32** | Fatty acids, total trans | g |
| **14** | Zinc, Zn | mg | **33** | Fatty acids, total saturated | g |
| **15** | Copper, Cu | mg | **34** | Fatty acids, total monounsaturated | g |
| **16** | Manganese, Mn | mg | **35** | Fatty acids, total polyunsaturated | g |
| **17** | Selenium, Se | mcg |  | **Antinutrients** |  |
| **18** | Vitamin A, RAE | mcg | **36** | Phytate | mg |
| **19** | Vitamin E (alpha-tocopherol) | mg | **37** | Tannic acid equivalents | mg |

**Supplementary Table 1: List of nutrients included in NBS** Macro and micronutrients were selected from the list of available nutrients in the Nutrient Database for Standard Reference Legacy Database (NDB) available at Food Data Central, focusing on essential nutrients for which there are Dietary Reference Values (DRVs) and/or Reference Intake Ranges (RIs). Simple sugars, sugar alcohols, alternative forms of vitamins A, D, E and K, provitamins A, amino acids, most polyphenols, and specific fatty acid chains are excluded. Two “antinutrients” are included to estimate iron and zinc bioavailability: 1) phytate data were obtained from PhyFoodComp1.0 available at INFOODS; and 2) polyphenol values for tea only were obtained from Table A1 from Hallberg and Hulthén (2000).
