# Supplementary material for: Tracing global flows of bioactive compounds from farm to fork in Nutrient Balance Sheets can help guide intervention towards healthier food supplies
Source: Nat Food. Author manuscript; Available in PMC 2022 Oct 11. (PMC7613697; doi:10.1038/s43016-022-00585-w)
Supplement: Supplementary Table 2 [file EMS153904-supplement-Supplementary_Table_2.docx]

| **Fruit and Veg (FV)** | **Heme Source Iron (HIS)** | **Food Group (FG)** | **Food Subgroup (FSG)** | **Food Category (FC)** | **Food Product (FP)** | | | | | | | |
| --- | --- | --- | --- | --- | --- | --- | --- | --- | --- | --- | --- | --- |
|  |  | NBS food group | FAO/FBS food group name | FAO/FBS food category | **SUA item** | | | | **SUA item** | | | |
|  |  |  |  |  | COICOP 2018 code | COICOP 2018 code | COICOP 2018 code | COICOP 2018 code | COICOP 2018 code | FAO/FBS item code | CPC 2.1 code | SUA item name |
| 0: No | 0: No | 1: Starchy Staples | 2905: Cereals - Excluding Beer | 2511: Wheat and products | 01.1.1.1.1 | 15 | 111 | ***Wheat*** | 01.1.1.3.9 | 22 | F0022 | Pastry |
|  |  |  |  |  | 01.1.1.2.1 | 16 | 23110 | Flour, wheat | 01.1.1.9.0 | 23 | 23220.01 | Starch, wheat |
|  |  |  |  |  | 01.1.1.9.0 | 17 | 39120.01 | Bran, wheat | 01.1.1.9.0 | 24 | 23220.02 | Gluten, wheat |
|  |  |  |  |  | 01.1.1.5.0 | 18 | 23710 | Macaroni | 01.1.1.4.0 | 41 | 23140.03 | Cereals, breakfast |
|  |  |  |  |  | 01.1.1.9.0 | 19 | 23140.01 | Germ, wheat | 01.1.1.9.0 | 110 | 23490.01 | Wafers |
|  |  |  |  |  | 01.1.1.3.1 | 20 | F0020 | Bread | 01.1.1.9.0 | 114 | 23180 | Mixes and doughs |
|  |  |  |  |  | 01.1.1.4.0 | 21 | 23140.02 | Bulgur | 01.1.1.9.0 | 115 | 23999.02 | Food preparations, flour, malt extract |
|  |  |  |  | 2805: Rice (Milled Equivalent) | 01.1.1.1.2 | 27 | 113 | Rice, paddy | 01.1.1.1.2 | 32 | 23161.03 | Rice, broken |
|  |  |  |  |  | 01.1.1.1.2 | 28 | 23162 | Rice, husked | 01.1.1.9.0 | 33 | 39130.01 | Gluten, rice |
|  |  |  |  |  | 01.1.1.1.2 | 29 | 23161.01 | Rice, milled/husked | 01.1.1.9.0 | 34 | 23220.03 | Starch, rice |
|  |  |  |  |  | 01.1.1.1.2 | 31 | 23161.02 | ***Rice, milled*** | 01.1.1.9.0 | 35 | 39120.02 | Bran, rice |
|  |  |  |  |  |  | | | | 01.1.1.2.2 | 38 | 23120.01 | Flour, rice |
|  |  |  |  | 2513: Barley and products | 01.1.1.1.4 | 44 | 115 | ***Barley*** | 01.1.1.9.0 | 47 | 39120.03 | Bran, barley |
|  |  |  |  |  | 01.1.1.9.0 | 45 | 23140.04 | Barley, pot | 01.1.1.2.4 | 48 | 23120.02 | Flour, barley and grits |
|  |  |  |  |  | 01.1.1.9.0 | 46 | 23140.05 | Barley, pearled | 01.1.1.9.0 | 49 | 24320 | Malt |
|  |  |  |  |  |  | | | | 01.1.1.9.0 | 50 | 23999.01 | Malt extract |
|  |  |  |  | 2514: Maize and products | 01.1.1.1.6 | 56 | 112 | ***Maize*** | 01.1.1.9.0 | 59 | 39120.04 | Bran, maize |
|  |  |  |  |  | 01.1.1.9.0 | 57 | 23140.06 | Germ, maize | 01.1.1.9.0 | 63 | 39130.02 | Gluten, maize |
|  |  |  |  |  | 01.1.1.2.6 | 58 | 23120.03 | Flour, maize | 01.1.1.9.0 | 64 | 23220.04 | Starch, maize |
|  |  |  |  |  |  | | | | 01.1.1.9.0 | 846 | 39130.04 | Feed and meal, gluten |
|  |  |  |  | 2515: Rye and products | 01.1.1.1.9 | 71 | 116 | ***Rye*** | 01.1.1.2.9 | 72 | 23120.04 | Flour, rye |
|  |  |  |  |  |  | | | | 01.1.1.9.0 | 73 | 39120.05 | Bran, rye |
|  |  |  |  | 2516: Oats | 01.1.1.1.9 | 75 | 117 | ***Oats*** | 01.1.1.9.0 | 76 | 23140.07 | Oats rolled |
|  |  |  |  |  |  | | | | 01.1.1.9.0 | 77 | 39120.06 | Bran, oats |
|  |  |  |  | 2517: Millet and products | 01.1.1.1.5 | 79 | 118 | ***Millet*** | 01.1.1.2.5 | 80 | 23120.05 | Flour, millet |
|  |  |  |  |  |  | | | | 01.1.1.9.0 | 81 | 39120.07 | Bran, millet |
|  |  |  |  | 2518: Sorghum and products | 01.1.1.1.3 | 83 | 114 | ***Sorghum*** | 01.1.1.2.3 | 84 | 23120.06 | Flour, sorghum |
|  |  |  |  |  |  | | | | 01.1.1.9.0 | 85 | 39120.08 | Bran, sorghum |
|  |  |  |  | *2520: Cereals, Other* | 01.1.1.9.0 | 68 | N/A | Popcorn | 01.1.1.2.9 | 98 | 23120.09 | Flour, triticale |
|  |  |  |  |  | 01.1.1.1.9 | 89 | 1192 | ***Buckwheat*** | 01.1.1.9.0 | 99 | 39120.11 | Bran, triticale |
|  |  |  |  |  | 01.1.1.2.9 | 90 | 23120.07 | Flour, buckwheat | 01.1.1.1.9 | 101 | 1195 | **Canary seed** |
|  |  |  |  |  | 01.1.1.9.0 | 91 | 39120.09 | Bran, buckwheat | 01.1.1.1.9 | 103 | 1199.02 | **Grain, mixed** |
|  |  |  |  |  | 01.1.1.1.7 | 92 | 1194 | **Quinoa** | 01.1.1.2.9 | 104 | 23120.1 | Flour, mixed grain |
|  |  |  |  |  | 01.1.1.1.9 | 94 | 1193 | **Fonio** | 01.1.1.9.0 | 105 | 39120.12 | Bran, mixed grains |
|  |  |  |  |  | 01.1.1.2.9 | 95 | 23120.08 | Flour, fonio | 01.1.1.1.9 | 108 | 1199.9 | **Cereals, nes*** |
|  |  |  |  |  | 01.1.1.9.0 | 96 | 39120.1 | Bran, fonio | 01.1.1.2.9 | 111 | 23120.9 | Flour, cereals |
|  |  |  |  |  | 01.1.1.1.9 | 97 | 1191 | **Triticale** | 01.1.1.9.0 | 112 | 39120.13 | Bran, cereals nes |
|  |  |  |  |  |  | | | | 01.1.1.4.0 | 113 | 23140.08 | Cereal preparations, nes |
|  |  |  | 2907: Starchy Roots | 2532: Cassava and products | 01.1.7.5.3 | 125 | 1520.01 | **Cassava** | 01.1.7.9.9 | 127 | 23230.02 | Tapioca, cassava |
|  |  |  |  |  | 01.1.7.9.1 | 126 | 23170.01 | Flour, cassava | 01.1.7.7.0 | 128 | 1520.02 | Cassava dried |
|  |  |  |  |  |  | | | | 01.1.7.9.9 | 129 | 23220.06 | Starch, cassava |
|  |  |  |  | 2531: Potatoes and products |  | 116 | 1510 | **Potatoes** | 01.1.7.8.0 | 118 | 21313 | Potatoes, frozen |
|  |  |  |  |  | 01.1.7.9.1 | 117 | 21392 | Flour, potatoes | 01.1.7.9.9 | 119 | 23220.05 | Starch, potatoes |
|  |  |  |  |  |  | | | | 01.1.7.9.9 | 121 | 23230.01 | Tapioca, potatoes |
|  |  |  |  | 2533: Sweet potatoes | 01.1.7.5.2 | 122 | 1530 | **Sweet potatoes** |  | | | |
|  |  |  |  | 2533: Yams | 01.1.7.5.4 | 137 | 1540 | **Yams** |  | | | |
|  |  |  |  | *2534: Roots, other* | 01.1.7.5.6 | 135 | 1591 | **Yautia (cocoyam)** | 01.1.7.5.9 | 149 | 1599.1 | **Roots and tubers, nes*** |
|  |  |  |  |  | 01.1.7.5.5 | 136 | 1550 | **Taro (cocoyam)** | 01.1.7.9.1 | 150 | 23170.02 | Flour, roots and tubers nes |
|  |  |  |  |  |  | | | | 01.1.7.7.0 | 151 | 1599.2 | Roots and tubers dried |
|  |  | 2: MN-Dense Vegetals | 2911: Pulses | 2546: Beans |  |  |  | Phaseolus spp.: | 01.1.7.6.1 | 176 | 1701 | **black gram, urd (Ph. mungo)** |
|  |  |  |  |  | 01.1.7.6.1 | 176 | 1701 | **kidney, haricot bean (Ph. vulgaris)** | 01.1.7.6.1 | 176 | 1701 | **scarlet runner bean (Ph. coccineus)** |
|  |  |  |  |  | 01.1.7.6.1 | 176 | 1701 | **lima, butter bean (Ph. lunatus)** | 01.1.7.6.1 | 176 | 1701 | **rice bean (Ph. calcaratus)** |
|  |  |  |  |  | 01.1.7.6.1 | 176 | 1701 | **adzuki bean (Ph. angularis)** | 01.1.7.6.1 | 176 | 1701 | **moth bean (Ph. aconitifolius)** |
|  |  |  |  |  | 01.1.7.6.1 | 176 | 1701 | **mungo bean, golden, green gram (Ph. aureus)** | 01.1.7.6.1 | 176 | 1701 | **tepary bean (Ph. acutifolius)** |
|  |  |  |  | 2547: Peas | 01.1.7.6.5 | 187 | 1705 | **Garden pea (Pisum sativum)** | 01.1.7.6.5 | 187 | 1705 | **field pea (P. arvense)** |
|  |  |  |  | *2549: Pulses, Other and products* | 01.1.7.6.2 | 181 | 1702 | **Broad beans, horse beans, dry** | 01.1.7.6.8 | 203 | 1708 | **Bambara beans** |
|  |  |  |  |  | 01.1.7.6.3 | 191 | 1703 | **Chick peas** | 01.1.7.6.9 | 205 | 1709.01 | **Vetches** |
|  |  |  |  |  | 01.1.7.6.6 | 195 | 1706 | **Cow peas, dry** | 01.1.7.6.9 | 210 | 1709.02 | **Lupins** |
|  |  |  |  |  | 01.1.7.6.7 | 197 | 1707 | **Pigeon peas** | 01.1.7.6.9 | 211 | 1709.9 | **Pulses, nes*** |
|  |  |  |  |  | 01.1.7.6.4 | 201 | 1704 | **Lentils** | 01.1.7.9.1 | 212 | 23170.03 | Flour, pulses |
|  |  |  |  |  |  | | | | 01.1.7.9 | 213 | 39120.14 | Bran, pulses |
|  |  |  | 2912: Treenuts | 2551: Nuts and products | 01.1.6.8.7 | 216 | 1377 | **Brazil nuts, with shell** | 01.1.6.8.9 | 226 | 1379.01 | **Areca nuts** |
|  |  |  |  |  | 01.1.6.8.2 | 217 | 1372 | **Cashew nuts, with shell** | 01.1.6.8.7 | 229 | 21429.01 | Brazil nuts, shelled |
|  |  |  |  |  | 01.1.6.8.3 | 220 | 1373 | **Chestnut** | 01.1.6.8.2 | 230 | 21424 | Cashew nuts, shelled |
|  |  |  |  |  | 01.1.6.8.1 | 221 | 1371 | **Almonds, with shell** | 01.1.6.8.1 | 231 | 21422 | Almonds shelled |
|  |  |  |  |  | 01.1.6.8.6 | 222 | 1376 | **Walnuts, with shell** | 01.1.6.8.6 | 232 | 21429.02 | Walnuts, shelled |
|  |  |  |  |  | 01.1.6.8.5 | 223 | 1375 | **Pistachios** | 01.1.6.8.4 | 233 | 21423 | Hazelnuts, shelled |
|  |  |  |  |  | 01.1.6.8.9 | 224 | 1379.02 | **Kola nuts** | 01.1.6.8.9 | 234 | 1379.9 | **Nuts, nes*** |
|  |  |  |  |  | 01.1.6.8.4 | 225 | 1374 | **Hazelnuts, with shell** | 01.1.6.9.4 | 235 | F0235 | Nuts, prepared (exc. groundnuts) |
|  |  |  | 2913: Oilcrops | 2555: Soyabeans | 01.1.7.3.5 | 236 | 141 | **Soybeans** | 01.1.9.3.9 | 240 | 23995.02 | Soya paste |
|  |  |  |  |  | 01.1.9.3.9 | 239 | 23995.01 | **Soya sauce** | 01.1.7.9.5 | 241 | 23999.03 | Soya curd |
|  |  |  |  | 2556: Groundnuts (Shelled Eq) | 01.1.6.8.8 | 242 | 142 | **Groundnuts, with shell** | 01.1.6.9.4 | 246 | 21495.01 | Groundnuts, prepared |
|  |  |  |  |  | 01.1.6.8.8 | 243 | 21421 | **Groundnuts, shelled** | 01.1.8.4.0 | 247 | 21495.02 | Peanut butter |
|  |  |  |  | 2557: Sunflower seed | 01.1.6.9.4 | 267 | 1445 | **Sunflower seed** |  | | | |
|  |  |  |  | 2558: Rape and Mustardseed | 01.1.6.9.4 | 270 | 1443 | **Rapeseed** | 01.1.6.9.4 | 292 | 1442 | **Mustard seed** |
|  |  |  |  |  |  | | | | 01.1.6.9.1 | 295 | 23995.03 | Flour, mustard |
|  |  |  |  | 2559: Cottonseed | 01.1.6.9.4 | 329 | 143 | **Cottonseed** |  |  |  |  |
|  |  |  |  | 2560: Coconuts - Incl Copra | 01.1.6.1.8 | 249 | 1460 | **Coconuts** | 01.1.6.7.9 | 250 | 21429.07 | Coconuts, desiccated |
|  |  |  |  |  |  | | | | 01.1.6.7.9 | 251 | 1492 | Copra |
| 0: No | 0: No | 2: MN-Dense Vegetals | 2913: Oilcrops | 2561: Sesame seed | 01.1.9.4.0 | 289 | 1444 | **Sesame seed** |  | | | |
|  |  |  |  | 2562: Palm kernels | 01.1.5.1.9 | 254 | 1491.01 | Oil, palm fruit | 01.1.6.8.9 | 256 | 1491.02 | **Palm kernels** |
|  |  |  |  | 2563: Olives (incl. pres.) | 01.1.7.4.7 | 260 | 1450 | **Olives** | 01.1.7.9.3 | 262 | F0262 | Olives preserved |
|  |  |  |  | *2570: Oilcrops, Other* | 01.1.6.8.9 | 263 | 1499.01 | **Karite nuts (sheanuts)** | 01.1.6.8.9 | 305 | 1499.04 | **Tallowtree seed** |
|  |  |  |  |  | 01.1.6.8.9 | 265 | 1447 | **Castor oil seed** | 01.1.6.8.9 | 310 | 1499.05 | **Kapok fruit** |
|  |  |  |  |  | 01.1.6.8.9 | 275 | 1499.02 | **Tung nuts** | 01.1.6.8.9 | 311 | 1499.06 | **Kapokseed in shell** |
|  |  |  |  |  | 01.1.6.8.9 | 277 | 1499.03 | **Jojoba seed** | 01.1.6.8.9 | 312 | 1499.07 | Kapokseed shelled |
|  |  |  |  |  | 01.1.6.8.9 | 280 | 1446 | **Safflower seed** | 01.1.6.8.9 | 333 | 1441 | **Linseed** |
|  |  |  |  |  | 01.1.9.4.0 | 296 | 1448 | **Poppy seed** | 01.1.6.8.9 | 336 | 1449.02 | **Hempseed** |
|  |  |  |  |  | 01.1.6.8.9 | 299 | 1449.01 | **Melonseed** | 01.1.6.8.9 | 339 | 1449.9 | **Oilseeds nes*** |
|  |  |  |  |  |  | | | | 01.1.6.9.1 | 343 | 21920 | Flour, oilseeds |
| 1: Yes |  |  | 2918: Vegetables | 2601: Tomatoes and products | 01.1.7.2.4 | 388 | 1234 | **Tomatoes** | 01.2.1.0.0 | 390 | 21321 | Juice, tomato |
|  |  |  |  |  | 01.2.1.0.0 | 389 | N/A | Juice, tomato, concentrated | 01.1.7.9.2 | 391 | 21399.01 | Tomatoes, paste |
|  |  |  |  |  |  | | | | 01.1.7.9.2 | 392 | 21399.02 | Tomatoes, peeled |
|  |  |  |  | 2602: Onions | 01.1.7.4.3 | 403 | 1253.02 | **Onions, dry** |  | | | |
| 1: Yes |  |  | 2918: Vegetables | *2605: Vegetables, Other* | 01.1.7.1.2 | 358 | 1212 | **Cabbages and other brassicas** | 01.1.7.8.0 | 447 | 21319.01 | Sweet corn frozen |
|  |  |  |  |  | 01.1.7.1.6 | 366 | 1216 | **Artichokes** | 01.1.7.9.2 | 448 | 21399.03 | Sweet corn prep or preserved |
|  |  |  |  |  | 01.1.7.1.1 | 367 | 1211 | **Asparagus** | 01.1.7.4.5 | 449 | 1270 | **Mushrooms and truffles** |
|  |  |  |  |  | 01.1.7.1.4 | 372 | 1214 | **Lettuce and chicory** | 01.1.7.7.0 | 450 | 21393.01 | Mushrooms, dried |
|  |  |  |  |  | 01.1.7.1.5 | 373 | 1215 | **Spinach** | 01.1.7.9.2 | 451 | 21397.01 | Mushrooms, canned |
|  |  |  |  |  | 01.1.7.1.7 | 378 | 1219.01 | **Cassava leaves** | 01.1.7.1.4 | 459 | 1691 | **Chicory roots** |
|  |  |  |  |  | 01.1.7.1.3 | 393 | 1213 | **Cauliflowers and broccoli** | 01.1.7.3.9 | 461 | 1356 | **Carobs** |
|  |  |  |  |  | 01.1.7.2.5 | 394 | 1235 | **Pumpkins, squash and gourds** | 01.1.7.4.9 | 463 | 1290.9 | **Vegetables, fresh nes*** |
|  |  |  |  |  | 01.1.7.2.2 | 397 | 1232 | **Cucumbers and gherkins** | 01.1.7.7.0 | 464 | N/A | Vegetables, dried nes* |
|  |  |  |  |  | 01.1.7.2.3 | 399 | 1233 | **Eggplants (aubergines)** | 01.1.7.9.2 | 465 | N/A | Vegetables, canned nes* |
|  |  |  |  |  | 01.1.7.2.1 | 401 | 1231 | **Chillies and peppers, green** | 01.2.1.0.0 | 466 | 21329 | Juice, vegetables nes* |
|  |  |  |  |  | 01.1.7.4.3 | 402 | 1253.01 | **Onions, shallots, green** | 01.1.7.7.0 | 469 | 21393.9 | Vegetables, dehydrated |
|  |  |  |  |  | 01.1.7.4.2 | 406 | 1252 | **Garlic** | 01.1.7.9.2 | 471 | 21340 | Vegetables in vinegar |
|  |  |  |  |  | 01.1.7.4.4 | 407 | 1254 | **Leeks, other alliaceous vegetables** | 01.1.7.9.2 | 472 | F0472 | Vegetables, preserved nes* |
|  |  |  |  |  | 01.1.7.3.1 | 414 | 1243 | **Beans, green** | 01.1.7.8.0 | 473 | F0473 | Vegetables, frozen |
|  |  |  |  |  | 01.1.7.3.3 | 417 | 1242 | **Peas, green** | 01.1.7.9.2 | 474 | 21330.9 | Vegetables, temp. pres. |
|  |  |  |  |  | 01.1.7.3.9 | 420 | 1241.9 | **Vegetables, leguminous nes*** | 01.1.7.8.0 | 475 | F0475 | Vegetables, preserved, frozen |
|  |  |  |  |  | 01.1.7.3.2 | 423 | 1241.01 | **String beans** | 01.1.7.9.9 | 476 | 23991.02 | Vegetables, homog. preps |
|  |  |  |  |  | 01.1.7.4.1 | 426 | 1251 | **Carrots and turnips** | 01.1.6.5.4 | 567 | 1221 | **Watermelons** |
|  |  |  |  |  | 01.1.7.2.6 | 430 | 1239.01 | **Okra** | 01.1.6.5.3 | 568 | 1229 | **Melons, other (inc.cantlpes)** |
|  |  |  |  |  | 01.1.7.4.8 | 446 | 1290.01 | **Maize, green** | 01.2.2.0.2 | 658 | 23912.01 | Coffee, subs. containing coffee |
|  |  |  | 2919: Fruits - Excluding Wine | 2611: Oranges, Mandarines | 01.1.6.2.3 | 490 | 1323 | **Oranges** | 01.2.1.0.0 | 492 | 21431.02 | Juice, orange, concentrated |
|  |  |  |  |  | 01.2.1.0.0 | 491 | 21431.01 | Juice, orange, single strength | 01.1.6.2.4 | 495 | 1324 | **Tangrns, mndrins, clmntines, satsumas** |
|  |  |  |  |  |  | | | | 01.2.1.0.0 | 496 | 21439.01 | Juice, tangerine |
|  |  |  |  | 2612: Lemons, Limes and products | 01.1.6.2.2 | 497 | 1322 | **Lemons and limes** | 01.2.1.0.0 | 498 | 21439.02 | Juice, lemon, single strength |
|  |  |  |  |  |  | | | | 01.2.1.0.0 | 499 | 21439.03 | Juice, lemon, concentrated |
|  |  |  |  | 2613: Grapefruit and products | 01.1.6.2.1 | 507 | 1321 | **Grapefruit (inc. pomelos)** | 01.2.1.0.0 | 509 | 21432 | Juice, grapefruit |
|  |  |  |  |  |  | | | | 01.2.1.0.0 | 510 | 21432.01 | Juice, grapefruit, concentrated |
|  |  |  |  | 2614: Citrus, Other | 01.1.6.2.9 | 512 | 1329 | **Fruit, citrus nes*** | 01.2.1.0.0 | 513 | 21439.04 | Juice, citrus, single strength |
|  |  |  |  |  |  | | | | 01.2.1.0.0 | 514 | 21439.05 | Juice, citrus, concentrated |
|  |  |  |  | 2615: Bananas | 01.1.6.1.2 | 486 | 1312 | **Bananas** |  | | | |
|  |  |  |  | 2616: Plantains | 01.1.7.5.7 | 489 | 1313 | **Plantains** |  | | | |
|  |  |  |  | 2617: Apples and products | 01.1.6.3.1 | 515 | 1341 | **Apples** | 01.2.1.0.0 | 518 | 21435.01 | Juice, apple, single strength |
|  |  |  |  |  |  | | | | 01.2.1.0.0 | 519 | 21435.02 | Juice, apple, concentrated |
|  |  |  |  | 2618: Pineapples and products | 01.1.6.1.7 | 574 | 1318 | **Pineapples** | 01.2.1.0.0 | 576 | 21433 | Juice, pineapple |
|  |  |  |  |  |  | 575 | 21491 | Pineapples canned | 01.2.1.0.0 | 580 | 21433.01 | Juice, pineapple, concentrated |
|  |  |  |  | 2619: Dates | 01.1.6.1.3 | 577 | 1314 | **Dates** |  | | | |
|  |  |  |  | 2620: Grapes and products (excl wine) | 01.1.6.5.1 | 560 | 1330 | **Grapes** | 01.2.1.0.0 | 562 | 21434 | Juice, grape |
|  |  |  |  |  | 01.1.6.7.1 | 561 | 21411 | **Raisins** | 01.2.1.0.0 | 563 | 24212.01 | Grapes, must |
|  |  |  |  | *2625: Fruits, Other* | 01.1.6.3.2 | 521 | 1342.01 | **Pears** | 01.1.6.4.9 | 558 | 1355.9 | **Berries nes*** |
|  |  |  |  |  | 01.1.6.3.2 | 523 | 1342.02 | **Quinces** | 01.1.6.1.4 | 569 | 1315 | **Figs** |
|  |  |  |  |  | 01.2.6.3.3 | 526 | 1343 | **Apricots** | 01.1.6.7.9 | 570 | 21419.02 | Figs dried |
|  |  |  |  |  | 01.1.6.7.9 | 527 | 21419.01 | Apricots, dry | 01.1.6.1.5 | 571 | 1316 | **Mangoes, mangosteens, guavas** |
|  |  |  |  |  | 01.1.6.3.4 | 530 | 1344.01 | **Cherries, sour** | 01.1.6.1.1 | 572 | 1311 | **Avocados** |
|  |  |  |  |  | 01.1.6.3.4 | 531 | 1344.02 | **Cherries** | 01.2.1.0.0 | 583 | 21439.08 | Juice, mango |
|  |  |  |  |  | 01.1.6.3.5 | 534 | 1345 | **Peaches and nectarines** | 01.1.6.5.5 | 587 | 1359.01 | **Persimmons** |
|  |  |  |  |  | 01.1.6.3.6 | 536 | 1346 | **Plums and sloes** | 01.1.6.5.6 | 591 | 1359.02 | **Cashewapple** |
|  |  |  |  |  | 01.1.6.7.9 | 537 | 21412 | Plums dried (prunes) | 01.1.6.5.2 | 592 | 1352 | **Kiwi fruit** |
|  |  |  |  |  | 01.2.1.0.0 | 538 | 21439.06 | Juice, plum, single strength | 01.1.6.1.6 | 600 | 1317 | **Papayas** |
|  |  |  |  |  | 01.2.1.0.0 | 539 | 21439.07 | Juice, plum, concentrated | 01.1.6.1.9 | 603 | 1319 | **Fruit, tropical fresh nes*** |
|  |  |  |  |  | 01.1.6.3.9 | 541 | 1349.2 | **Fruit, stone nes*** | 01.1.6.7.9 | 604 | 21419.91 | Fruit, tropical dried nes* |
|  |  |  |  |  | 01.1.6.3.9 | 542 | 1349.1 | **Fruit, pome nes*** | 01.1.6.5.9 | 619 | 1359.9 | **Fruit, fresh nes*** |
|  |  |  |  |  | 01.1.6.4.5 | 544 | 1354 | **Strawberries** | 01.1.6.7.9 | 620 | 21419.99 | Fruit, dried nes* |
|  |  |  |  |  | 01.1.6.4.3 | 547 | 1353.01 | **Raspberries** | 01.2.1.0.0 | 622 | 21439.9 | Juice, fruit nes* |
|  |  |  |  |  | 01.1.6.4.2 | 549 | 1351.02 | **Gooseberries** | 01.1.6.9.9 | 623 | F0623 | Fruit, prepared nes* |
|  |  |  |  |  | 01.1.6.4.1 | 550 | 1351.01 | **Currants** | 01.1.6.9.1 | 624 | 23170.04 | Flour, fruit |
|  |  |  |  |  | 01.1.6.4.6 | 552 | 1355.01 | **Blueberries** | 01.1.6.9.2 | 625 | 23670.02 | Fruits, nuts, peel, sugar pres. |
|  |  |  |  |  | 01.1.6.4.7 | 554 | 1355.02 | **Cranberries** | 01.1.6.9.3 | 626 | 23991.03 | Fruit, cooked, homog. preps. |
| 0: No |  |  | 2923: Spices | 2640: Pepper | 01.1.9.4.0 | 687 | 1651 | **Pepper (piper spp.)** |  | | | |
|  |  |  |  | 2641: Pimento | 01.1.9.4.0 | 689 | 1652 | **Chillies and peppers, dry** |  | | | |
|  |  |  |  | 2642: Cloves | 01.1.9.4.0 | 698 | 1656 | **Cloves** |  | | | |
|  |  |  |  | *2645: Spices, Other* | 01.1.9.4.0 | 692 | 1658 | **Vanilla** | 01.1.9.4.0 | 711 | 1654 | **Anise, badian, fennel, coriander** |
|  |  |  |  |  | 01.1.9.4.0 | 693 | 1655 | **Cinnamon (canella)** | 01.1.9.4.0 | 720 | 1657 | **Ginger** |
|  |  |  |  |  | 01.1.9.4.0 | 702 | 1653 | **Nutmeg, mace and cardamoms** | 01.1.9.4.0 | 723 | 1699 | **Spices, nes*** |
| 1: Yes |  |  | 2961: Aquatic Products, Other | 2775: Aquatic Plants | 01.1.7.4.6 | 1594 | N/A | Aquatic plants, fresh | 01.1.7.7.0 | 1595 | N/A | Aquatic plants, dried |
|  |  |  |  |  |  | | | | 01.1.7.9.9 | 1596 | N/A | Aquatic plants, other preps |
| 0: No | 1: Yes | 3: Animal Source Foods | 2943: Meat | 2731: Bovine Meat | 01.1.2.2.1 | 867 | 21111.01 | **Meat, cattle** | 01.1.2.5.1 | 874 | 21184.01 | Meat, beef and veal sausages |
|  |  |  |  |  | 01.1.2.2.1 | 870 | 21111.02 | Meat, cattle, boneless (beef & veal) | 01.1.2.5.9 | 875 | F0875 | Meat, beef, preparations |
|  |  |  |  |  | 01.1.2.3.1 | 872 | 21182 | Meat, beef, dried, salted, smoked | 01.1.2.5.2 | 876 | N/A | Meat, beef, canned |
|  |  |  |  |  | 01.1.2.5.9 | 873 | 21185 | Meat, extracts | 01.1.2.5.9 | 877 | 23991.04 | Meat, homog. preps. |
|  |  |  |  |  |  | | | | 01.1.2.2.1 | 947 | 21112 | **Meat, buffalo** |
|  |  |  |  | 2732: Mutton & Goat Meat | 01.1.2.2.3 | 977 | 21115 | **Meat, sheep** | 01.1.2.2.3 | 1017 | 21116 | **Meat, goat** |
|  |  |  |  | 2733: Pigmeat | 01.1.2.2.2 | 1035 | 21113.01 | **Meat, pig** | 01.1.2.3.2 | 1039 | 21181 | Bacon and ham |
|  |  |  |  |  | 01.1.2.2.2 | 1038 | 21113.02 | Meat, pork | 01.1.2.5.1 | 1041 | 21184.02 | Meat, pig sausages |
|  |  |  |  |  |  | | | | 01.1.2.5.9 | 1042 | F1042 | Meat, pig, preparations |
|  |  |  |  | 2734: Poultry Meat | 01.1.2.2.4 | 1058 | 21121 | **Meat, chicken** | 01.1.2.2.4 | 1069 | 21122 | **Meat, duck** |
|  |  |  |  |  | 01.1.2.5.3 | 1060 | 21189.02 | Fat, liver prepared (foie gras) | 01.1.2.2.4 | 1073 | 21123 | **Meat, goose and guinea fowl** |
|  |  |  |  |  | 01.1.2.5.2 | 1061 | F1061 | Meat, chicken, canned | 01.1.2.2.4 | 1080 | 21124 | **Meat, turkey** |
| 0: No | 1: Yes | 3: Animal Source Foods | 2943: Meat | *2735: Meat, Other* | 01.1.2.2.4 | 1089 | 21170.01 | **Meat, bird nes*** | 01.1.2.2.9 | 1163 | 21170.02 | **Meat, game** |
|  |  |  |  |  | 01.1.2.2.6 | 1097 | 21118.01 | **Meat, horse** | 01.1.2.2.9 | 1151 | 21119.01 | **Meat, other rodents** |
|  |  |  |  |  | 01.1.2.2.6 | 1108 | 21118.02 | **Meat, ass** | 01.1.2.2.7 | 1158 | 21117.02 | **Meat, other camelids** |
|  |  |  |  |  | 01.1.2.2.6 | 1111 | 21118.03 | **Meat, mule** | 01.1.2.3.9 | 1164 | 21183 | **Meat, dried nes*** |
|  |  |  |  |  | 01.1.2.2.7 | 1127 | 21117.01 | **Meat, camel** | 01.1.2.2.9 | 1166 | 21170.92 | **Meat, nes*** |
|  |  |  |  |  | 01.1.2.2.5 | 1141 | 21114 | **Meat, rabbit** | 01.1.2.5.9 | 1172 | F1172 | Meat, nes, preparations |
|  |  |  |  |  |  | | | | 01.1.2.2.9 | 1176 | 2920 | **Snails, not sea** |
|  |  |  | 2945: Offals | 2736: Offals, Edible | 01.1.2.4.0 | 868 | 21151 | **Offals, edible, cattle** | 01.1.2.4.0 | 1074 | 21160.02 | **Offals, liver geese** |
|  |  |  |  |  | 01.1.2.5.3 | 878 | 21189.01 | Liver prep. | 01.1.2.4.0 | 1075 | 21160.03 | **Offals, liver duck** |
|  |  |  |  |  | 01.1.2.4.0 | 948 | 21152 | **Offals, edible, buffaloes** | 01.1.2.4.0 | 1081 | 21160.04 | **Offals, liver turkeys** |
|  |  |  |  |  | 01.1.2.4.0 | 978 | 21155 | **Offals, sheep,edible** | 01.1.2.4.0 | 1098 | 21159.01 | **Offals, horses** |
|  |  |  |  |  | 01.1.2.4.0 | 1018 | 21156 | **Offals, edible, goats** | 01.1.2.4.0 | 1128 | 21159.02 | **Offals, edible, camels** |
|  |  |  |  |  | 01.1.2.4.0 | 1036 | 21153 | **Offals, pigs, edible** | 01.1.2.4.0 | 1159 | N/A | Offals, other camelids |
|  |  |  |  |  | 01.1.2.4.0 | 1059 | 21160.01 | **Offals, liver chicken** | 01.1.2.4.0 | 1167 | 21170.93 | **Offals, nes*** |
|  | 0: No |  | 2946: Animal fats | 2740: Butter, Ghee | 01.1.5.2.1 | 886 | 22241.01 | **Butter, cow milk** | 01.1.5.2.9 | 953 | 22242.02 | Ghee, of buffalo milk |
|  |  |  |  |  | 01.1.5.2.9 | 887 | 22241.02 | Ghee, butteroil of cow milk | 01.1.5.2.1 | 983 | 22249.01 | **Butter and ghee, sheep milk** |
|  |  |  |  |  | 01.1.5.2.1 | 952 | 22242.01 | **Butter, buffalo milk** | 01.1.5.2.1 | 1022 | 22249.02 | **Butter of goat mlk** |
|  |  |  | 2946: Animal fats | 2743: Cream | 01.1.4.3.3 | 885 | 22120 | **Cream fresh** |  | | | |
|  |  |  |  | 2737: Fats, Animals, Raw | 01.1.5.9.2 | 869 | 21512 | **Fat, cattle** | 01.1.5.9.2 | 1065 | 21511.03 | **Fat, poultry** |
|  |  |  |  |  | 01.1.5.9.2 | 871 | 21512.01 | **Fat, cattle butcher** | 01.1.5.9.2 | 1066 | 21522 | Fat, poultry, rendered |
|  |  |  |  |  | 01.1.5.9.2 | 949 | 21513 | **Fat, buffaloes** | 01.1.5.9.9 | 1129 | 21519.02 | **Fat, camels** |
|  |  |  |  |  | 01.1.5.9.2 | 979 | 21514 | **Fat, sheep** | 01.1.5.9.9 | 1160 | 21519.03 | **Fat, other camelids** |
|  |  |  |  |  | 01.1.5.9.2 | 994 | F0994 | Grease incl. lanolin wool | 01.1.5.9.9 | 1168 | 21529.03 | **Oils, fats of animal nes*** |
|  |  |  |  |  | 01.1.5.9.2 | 1019 | 21515 | **Fat, goats** | 01.1.5.9.1 | 1221 | 21529.02 | Lard stearine oil |
|  |  |  |  |  | 01.1.5.9.1 | 1037 | 21511.01 | **Fat, pigs** | 01.1.3.3.3 | 1222 | 21932.01 | Degras |
|  |  |  |  |  | 01.1.5.9.1 | 1040 | 21511.02 | **Fat, pig butcher** | 01.1.5.9.2 | 1225 | 21523 | **Tallow** |
|  |  |  |  |  | 01.1.5.9.1 | 1043 | 21521 | **Lard** | 01.1.5.9.9 | 1243 | F1243 | Fat, nes, prepared* |
|  |  |  |  | 2781: Fish, Body Oil | 01.1.5.9.3 | 1509 | N/A | **Frwt Bdy Oil** | 01.1.5.9.4 | 1535 | N/A | **Pelg Bdy Oil** |
|  |  |  |  |  | 01.1.5.9.4 | 1522 | N/A | **Dmrs Bdy Oil** | 01.1.5.9.4 | 1548 | N/A | **Marn Bdy Oil** |
|  |  |  |  |  |  | | | | 01.1.5.9.9 | 1582 | N/A | **Aq M Oils** |
|  |  |  |  | 2782: Fish, Liver Oil | 01.1.5.9.4 | 1510 | N/A | **Frwt Lvr Oil** | 01.1.5.9.4 | 1536 | N/A | **Pelg Lvr Oil** |
|  |  |  |  |  | 01.1.5.9.4 | 1523 | N/A | **Demersal Liver Oils** | 01.1.5.9.4 | 1549 | N/A | **Marine nes Liver Oils** |
|  |  |  | 2949: Eggs | 2744: Eggs | 01.1.4.8.9 | 916 | 23993.01 | Egg albumine | 01.1.4.8.9 | 1063 | 23993.02 | Eggs, liquid |
|  |  |  |  |  | 01.1.4.8.1 | 1062 | 231 | **Eggs, hen, in shell** | 01.1.4.8.9 | 1064 | 23993.03 | Eggs, dried |
|  |  |  |  |  |  | | | | 01.1.4.8.2 | 1091 | 232 | **Eggs, other bird, in shell** |
|  |  |  | 2948: Milk - Excluding Butter | 2848: Milk - Excluding Butter | 01.1.4.1.1 | 882 | 2211 | **Milk, whole fresh cow** | 01.1.4.5.0 | 904 | 22251.02 | Cheese, skimmed cow milk |
|  |  |  |  |  | 01.1.4.2.0 | 888 | 22110.02 | Milk, skimmed cow | 01.1.4.9.0 | 905 | 22251.03 | Whey, cheese |
|  |  |  |  |  | 01.1.4.3.1 | 889 | 22222.01 | Milk, whole condensed | 01.1.4.5.0 | 907 | 22251.04 | Cheese, processed |
|  |  |  |  |  | 01.1.4.9.0 | 890 | 22130.03 | Whey, condensed | 01.1.4.3.9 | 908 | 22110.03 | Milk, reconstituted |
|  |  |  |  |  | 01.1.4.6.0 | 891 | 22230.01 | Yoghurt | 01.1.4.7.0 | 909 | 22290 | Milk, products of natural constituents nes |
|  |  |  |  |  | 01.1.4.6.0 | 892 | 22230.02 | Yoghurt, concentrated or not | 01.1.4.7.0 | 910 | 22270 | Ice cream and edible ice |
|  |  |  |  |  | 01.1.4.6.0 | 893 | 22230.03 | Buttermilk, curdled, acidified milk | 01.1.4.9.0 | 917 | 22260 | Casein |
|  |  |  |  |  | 01.1.4.3.1 | 894 | 22221.01 | Milk, whole evaporated | 01.1.4.1.2 | 951 | 2212 | **Milk, whole fresh buffalo** |
|  |  |  |  |  | 01.1.4.3.1 | 895 | 22221.02 | Milk, skimmed evaporated | 01.1.4.2.0 | 954 | 22110.04 | Milk, skimmed buffalo |
|  |  |  |  |  | 01.1.4.3.1 | 896 | 22222.02 | Milk, skimmed condensed | 01.1.4.5.0 | 955 | 22252 | Cheese, buffalo milk |
|  |  |  |  |  | 01.1.4.3.2 | 897 | 22211 | Milk, whole dried | 01.1.4.1.3 | 982 | 2291 | **Milk, whole fresh sheep** |
|  |  |  |  |  | 01.1.4.3.2 | 898 | 22212 | Milk, skimmed dried | 01.1.4.5.0 | 984 | 22253 | Cheese, sheep milk |
|  |  |  |  |  | 01.1.4.6.0 | 899 | 22230.04 | Milk, dry buttermilk | 01.1.4.2.0 | 985 | 22110.05 | Milk, skimmed sheep |
|  |  |  |  |  | 01.1.4.9.0 | 900 | 22130.02 | Whey, dry | 01.1.4.1.3 | 1020 | 2292 | **Milk, whole fresh goat** |
|  |  |  |  |  | 01.1.4.5.0 | 901 | 22251.01 | Cheese, whole cow milk | 01.1.4.5.0 | 1021 | 22254 | Cheese of goat mlk |
|  |  |  |  |  | 01.1.4.9.0 | 903 | 22130.01 | Whey, fresh | 01.1.4.2.0 | 1023 | 22110.06 | Milk, skimmed goat |
|  |  |  |  |  |  | | | | 01.1.4.1.4 | 1130 | 2293 | **Milk, whole fresh camel** |
|  | 1: Yes |  | 2960: Fish, Seafood | 2761: Freshwater Fish | 01.1.3.1.1 | 1501 | N/A | **Frwtr Diad F** | 01.1.3.2.9 | 1505 | N/A | Frwtr Cured |
|  |  |  |  |  | 01.1.3.1.1 | 1502 | N/A | Frwtr Fz Whl | 01.1.3.3 | 1506 | N/A | Frwtr Canned |
|  |  |  |  |  | 01.1.3.1.1 | 1503 | N/A | Frwtr Fillet | 01.1.3.3 | 1507 | N/A | Frwtr Pr nes |
|  |  |  |  |  | 01.1.3.1.1 | 1504 | N/A | Frwtr Fz Flt | 01.1.3.3 | 1508 | N/A | Frwtr Meals |
|  |  |  |  | 2762: Demersal Fish | 01.1.3.1.3/4 | 1514 | N/A | **Dmrsl Fresh** | 01.1.3.2.2 | 1518 | N/A | Dmrsl Cured |
|  |  |  |  |  | 01.1.3.1.3/4 | 1515 | N/A | Dmrsl Fz Whl | 01.1.3.3 | 1519 | N/A | Dmrsl Canned |
|  |  |  |  |  | 01.1.3.1.3/4 | 1516 | N/A | Dmrsl Fillet | 01.1.3.3 | 1520 | N/A | Dmrsl Pr nes |
|  |  |  |  |  | 01.1.3.1.3/4 | 1517 | N/A | Dmrsl Fz Flt | 01.1.3.3 | 1521 | N/A | Dmrsl Meals |
|  |  |  |  | 2763: Pelagic Fish | 01.1.3.1.2/5/6 | 1527 | N/A | **Pelagic Frsh** | 01.1.3.2.1 | 1531 | N/A | Pelgc Cured |
|  |  |  |  |  | 01.1.3.1.2/5/6 | 1528 | N/A | Pelgc Fz Whl | 01.1.3.3.1/2 | 1532 | N/A | Pelgc Canned |
|  |  |  |  |  | 01.1.3.1.2/5/6 | 1529 | N/A | Pelgc Fillet | 01.1.3.3.1/2 | 1533 | N/A | Pelgc Pr nes |
|  |  |  |  |  | 01.1.3.1.2/5/6 | 1530 | N/A | Pelgc Fz Flt | 01.1.3.3.1/2 | 1534 | N/A | Pelgc Meals |
|  |  |  |  | *2764: Marine Fish, Other* | 01.1.3.1.9 | 1540 | N/A | **Marine nes F** | 01.1.3.2.9 | 1544 | N/A | Marin Cured |
|  |  |  |  |  | 01.1.3.1.9 | 1541 | N/A | Marin Fz Whl | 01.1.3.3 | 1545 | N/A | Marin Canned |
|  |  |  |  |  | 01.1.3.1.9 | 1542 | N/A | Marin Fillet | 01.1.3.3 | 1546 | N/A | Marin Pr nes |
|  |  |  |  |  | 01.1.3.1.9 | 1543 | N/A | Marin Fz Flt | 01.1.3.3 | 1547 | N/A | Marin Meals |
|  |  |  |  | 2765: Crustaceans | 01.1.3.4.1/2 | 1553 | N/A | **Crstaceans F** | 01.1.3.6.1/2 | 1556 | N/A | Crstc Canned |
|  |  |  |  |  | 01.1.3.4.1/2 | 1554 | N/A | Crstc Frozen | 01.1.3.6.1/2 | 1557 | N/A | Crstc Pr nes |
|  |  |  |  |  | 01.1.3.4.1/2 | 1555 | N/A | Crstc Cured | 01.1.3.6.1/2 | 1558 | N/A | Crstc Meals |
|  |  |  |  | 2766: Cephalopods | 01.1.3.4.3/4 | 1570 | N/A | **Cephlp Fresh** | 01.1.3.6.3/4 | 1573 | N/A | Cphlp Canned |
|  |  |  |  |  | 01.1.3.4.3/4 | 1571 | N/A | Cphlp Frozen | 01.1.3.6.3/4 | 1574 | N/A | Cphlp Pr nes |
|  |  |  |  |  | 01.1.3.4.3/4 | 1572 | N/A | Cphlp Cured | 01.1.3.6.3/4 | 1575 | N/A | Cphlp Meals |
|  |  |  |  | *2767: Molluscs, Other* | 01.1.3.4.5 | 1562 | N/A | **Mlluscs Frsh** | 01.1.3.6.5 | 1564 | N/A | Molsc Cured |
|  |  |  |  |  | 01.1.3.4.5 | 1563 | N/A | Molsc Frozen | 01.1.3.6.5 | 1565 | N/A | Molsc Canned |
|  |  |  |  |  |  | | | | 01.1.3.6.5 | 1566 | N/A | Molsc Meals |
|  |  |  | 2961: Aquatic Products, Other | 2768: Meat, Aquatic Mammals | 01.1.3.4.9 | 1580 | N/A | Aq M Meat | 01.1.3.6.9 | 1583 | N/A | Aq M Prep Ns |
|  |  |  |  | *2769: Aquatic Animals, Others* | 01.1.2.2.9 | 1587 | N/A | Aqutc Anim F | 01.1.2.5.1 | 1589 | N/A | Aquatic Animals Meals |
|  |  |  |  |  | 01.1.2.3.9 | 1588 | N/A | Aq A Cured | 01.1.2.5.1 | 1590 | N/A | Aq A Prep Ns |
|  | 0: No | 4: Other | 2908: Sugar Crops | 2236: Sugar cane | 01.1.8.1.1 | 156 | 1802 | **Sugar cane** |  | | | |
|  |  |  |  | 2537: Sugar beet | 01.1.8.1.2 | 157 | 1801 | **Sugar beet** |  | | | |
|  |  |  | 2909: Sugar & Sweeteners | 2541: Sugar non-centrifugal | 01.1.8.1.1 | 163 | 23511.02 | Sugar non-centrifugal |  | | | |
|  |  |  |  | 2542: Sugar (Raw Equivalent) | 01.1.8.1.1 | 158 | 23511.01 | Sugar, cane, raw, centrifugal | 01.1.8.1.1 | 164 | 23520 | Sugar refined |
|  |  |  |  |  | 01.1.8.1.2 | 159 | 23512 | Sugar, beet, raw, centrifugal | 01.1.8.1.1 | 168 | 23670.01 | Sugar confectionery |
|  |  |  |  |  | 01.1.8.1 | 162 | 2351F | **Sugar Raw Centrifugal** | 01.1.8.2.0 | 171 | 23530 | Sugar flavoured |
|  |  |  |  | 2745: Honey | 01.1.8.3.1 | 1182 | 2910 | **Honey, natural** |  | | | |
|  |  |  |  | *2543: Sweeteners, Other* | 01.1.8.2.0 | 154 | 23210.01 | Fructose chemically pure | 01.1.8.2.0 | 166 | 23210.03 | **Fructose and syrup, other** |
|  |  |  |  |  | 01.1.8.2.0 | 155 | 23210.02 | Maltose chemically pure | 01.1.8.2.0 | 167 | 23210.04 | **Sugar, nes*** |
|  |  |  |  |  | 01.1.8.2.0 | 160 | 23530 | **Maple sugar and syrups** | 01.1.8.2.0 | 172 | 23210.05 | Glucose and dextrose |
|  |  |  |  |  | 01.1.8.2.0 | 161 | 1809 | **Sugar crops, nes*** | 01.1.8.2.0 | 173 | 23210.06 | Lactose |
|  |  |  |  |  | 01.1.8.2.0 | 165 | 23540 | **Molasses** | 01.1.8.2.0 | 175 | 23210.08 | Isoglucose |
|  |  |  |  |  |  | | | | 01.2.4.0.0 | 633 | 24490 | Beverages, non alcoholic |
| 0: No | 0: No | 4: Other | 2914: Vegetable Oils | 2571: Soyabean Oil | 01.1.5.1.4 | 237 | 2161 | **Oil, soybean** |  | | | |
|  |  |  |  | 2572: Groundnut Oil | 01.1.5.1.5 | 244 | 2162 | **Oil, groundnut** |  | | | |
|  |  |  |  | 2573: Sunflowerseed Oil | 01.1.5.1.1 | 268 | 21631.01 | **Oil, sunflower** |  | | | |
|  |  |  |  | 2574: Rape and Mustard Oil | 01.1.5.1.9 | 271 | 21641.01 | **Oil, rapeseed** | 01.1.5.1.9 | 293 | 21641.02 | **Oil, mustard** |
|  |  |  |  | 2575: Cottonseed Oil | 01.1.5.1.9 | 331 | 2168 | **Oil, cottonseed** |  | | | |
|  |  |  |  | 2576: Palmkernel Oil | 01.1.5.1.9 | 258 | 21691.14 | **Oil, palm kernel** |  | | | |
|  |  |  |  | 2577: Palm Oil | 01.1.5.1.2 | 257 | 2165 | **Oil, palm** | 01.1.5.1.9 | 1276 | 34120 | Fatty acids |
|  |  |  |  |  |  | | | | 01.1.5.1.9 | 1277 | 21932.02 | Fatty substance residues |
|  |  |  |  | 2578: Coconut Oil | 01.1.5.1.6 | 252 | 2166 | **Oil, coconut (copra)** |  | | | |
|  |  |  |  | 2579: Sesameseed Oil | 01.1.5.1.9 | 290 | 21691.07 | **Oil, sesame** |  | | | |
|  |  |  |  | 2580: Olive Oil | 01.1.5.1.3 | 261 | 2167 | **Oil, olive, virgin** | 01.1.5.1.9 | 274 | 21673 | Oil, olive residues |
|  |  |  |  | 2581: Ricebran Oil | 01.1.5.1.9 | 36 | 21691.01 | **Oil, rice bran** |  | | | |
|  |  |  |  | 2582: Maize Germ Oil | 01.1.5.1.7 | 60 | 21691.02 | **Oil, maize** |  | | | |
|  |  |  |  | *2586: Oilcrops Oil, Other* | 01.1.5.1.9 | 264 | 21691.03 | **Butter of karite nuts** | 01.1.5.1.9 | 334 | 21691.12 | **Oil, linseed** |
|  |  |  |  |  | 01.1.5.1.9 | 266 | 21691.04 | **Oil, castor beans** | 01.1.5.1.9 | 337 | 21691.13 | **Oil, hempseed** |
|  |  |  |  |  | 01.1.5.1.9 | 276 | 21691.05 | **Oil, tung nuts** | 01.1.5.1.9 | 340 | 21691.9 | **Oil, vegetable origin nes*** |
|  |  |  |  |  | 01.1.5.1.9 | 278 | 21691.06 | **Oil, jojoba** | 01.1.8.5.9 | 664 | 23620 | **Cocoa, butter** |
|  |  |  |  |  | 01.1.5.1.9 | 281 | 21631.02 | **Oil, safflower** | 01.1.5.3.0 | 1241 | 21700.01 | **Margarine, liquid** |
|  |  |  |  |  | 01.1.5.1.9 | 297 | 21691.08 | **Oil, poppy** | 01.1.5.3.0 | 1242 | 21700.02 | **Margarine, short** |
|  |  |  |  |  | 01.1.5.1.9 | 306 | 21691.09 | **Vegetable tallow** | 01.1.5.1.9 | 1273 | 21693.02 | **Castor oil, hydrog. (opal wax)** |
|  |  |  |  |  | 01.1.5.1.9 | 307 | 21691.1 | **Oil, stillingia** | 01.1.5.1.9 | 1274 | 34550 | Oil, boiled etc |
|  |  |  |  |  | 01.1.5.1.9 | 313 | 21691.11 | **Oil, kapok** | 01.1.5.1.9 | 1275 | F1275 | **Oil, hydrogenated** |
|  |  |  | 2922: Stimulants | 2630: Coffee and products | 01.2.2.0.1 | 656 | 1610 | **Coffee, green** | 01.2.2.0.1 | 657 | 23911 | Coffee, roasted |
|  |  |  |  |  |  |  |  |  | 01.2.2.0.2 | 659 | 23912.02 | Coffee, extracts |
|  |  |  |  | 2633: Cocoa Beans and products | 01.1.8.5.2 | 661 | 1640 | **Cocoa, beans** | 01.1.8.5.9 | 665 | F0665 | Cocoa, powder & cake |
|  |  |  |  |  | 01.1.8.5.9 | 662 | 23610.01 | Cocoa, paste | 01.1.8.5.9 | 666 | F0666 | Chocolate products nes* |
|  |  |  |  | 2635: Tea (including mate) | 01.2.3.0 | 667 | 1620 | **Tea** | 01.2.3.0.5 | 671 | 1630 | Matcha |
|  |  |  |  |  |  |  |  |  | 01.2.3.0.9 | 672 | 23914 | Tea, mate extracts |
|  |  |  | 2924: Alcoholic Beverages | 2655: Wine | 02.1.2.1 | 564 | 24212.02 | **Wine** | 02.1.2.1 | 565 | 24220 | Vermouths & similar |
|  |  |  |  | 2656: Beer | 02.2.3.0 | 51 | 24310.01 | **Beer of barley** |  | | | |
|  |  |  |  | 2657: Beverages, Fermented | 02.1.9.0 | 26 | 24230.01 | **Beverages, fermented wheat** | 02.2.3.0 | 82 | 24310.03 | **Beer of millet** |
|  |  |  |  |  | 02.1.9.0 | 39 | 24230.02 | **Beverages, fermented rice** | 02.2.3.0 | 86 | 24310.04 | **Beer of sorghum** |
|  |  |  |  |  | 02.2.3.0 | 66 | 24310.02 | **Beer of maize** | 02.1.9.0 | 517 | 24230.03 | **Cider etc** |
|  |  |  |  | 2658: Beverages, Alcoholic | 02.1.1.0 | 634 | 2413 | **Beverages, distilled alcoholic** |  | | | |
|  |  |  |  | 2659: Alcohol, Non-Food |  | 632 | 24110 | Alcohol non food |  | | | |
|  |  |  | 2928: Miscellaneous | 2680: Infant food | 01.1.9.2.1 | 109 | 23991.01 | **Preparations for infant consumption, usually containing some non-cereal ingredients.** |  |  |  |  |
|  |  |  |  | 2899: Miscellaneous | 01.1.9.3.9 | 1232 | F1232 | Food prep nes** |  | | | |

**Supplementary Table 2: NBS food categories and relationships to food products**

The NBS is categorized into variables for fruits and vegetables (FV), heme-source iron (HIS), food group (FG), food subgroup (FSG) and food category (FC). FCs are constructed from 456 Supply and Utilization Accounts (SUAs), here called food products (FPs). For each variable, the NBS and/or FAO code is provided. For each FP, the CPC v2.1 code is provided and the authors’ assumed relationship to a COICOP 2018 code. Within each FC, FPs used as primary commodities (used for FCT match) are shown in bold/italicized font. Within each FSG, food category other (FCO) is shown in italics. The FCO contains multiple primary commodities and an FP deemed ‘Not Elsewhere Specified’ (NES). NES are sets of additional FPs. Fish and seafood categories were informed using the FAO/Coordinating Working Party on Fishery Statistics (CWP) Handbook of Fishery Statistical Standards, Annex S.II: International Standard Statistical Classification of Aquatic Animals and Plants (ISSCAAP). Lists of fish species within these groups were obtained from the FAO software FishStatJ. (*) = NES foods; (**) = Only in 2014-2018 data. FPs that do not have any data in the 2014-2018 SUA data download are shown in gray highlight.
