# Supplementary material for: Tracing global flows of bioactive compounds from farm to fork in Nutrient Balance Sheets can help guide intervention towards healthier food supplies
Source: Nat Food. Author manuscript; Available in PMC 2022 Oct 11. (PMC7613697; doi:10.1038/s43016-022-00585-w)
Supplement: Supplementary Table 3 [file EMS153904-supplement-Supplementary_Table_3.docx]

| **FBS_itemcode** | **FBS_itemname** | **fdc_id** | **ndb_id** | **fdc_name** |  |
| --- | --- | --- | --- | --- | --- |
| 2511 | Wheat and products | 168889 | 20071 | Wheat, hard red spring |  |
| 2511 | Wheat and products | 168890 | 20072 | Wheat, hard red winter |  |
| 2511 | Wheat and products | 168891 | 20073 | Wheat, soft red winter |  |
| 2511 | Wheat and products | 169719 | 20074 | Wheat, hard white |  |
| 2511 | Wheat and products | 169720 | 20075 | Wheat, soft white |  |
| 2511 | Wheat and products | 169721 | 20076 | Wheat, durum |  |
| 2511 | Wheat and products | 169743 | 20138 | Wheat, Kamut khorasan, uncooked |  |
| 2513 | Barley and products | 170283 | 20004 | Barley, hulled |  |
| 2514 | Maize and products | 168920 | 20314 | Corn grain, white |  |
| 2514 | Maize and products | 170288 | 20014 | Corn grain, yellow |  |
| 2515 | Rye and products | 168884 | 20062 | Rye grain |  |
| 2516 | Oats | 169705 | 20038 | Oats |  |
| 2516 | Oats | 173904 | 8120 | Cereals, oats, regular and quick, not fortified, dry |  |
| 2517 | Millet and products | 169702 | 20031 | Millet, raw |  |
| 2518 | Sorghum and products | 169716 | 20067 | Sorghum grain |  |
| 2520 | Cereals, Other | 168874 | 20035 | Quinoa, uncooked |  |
| 2520 | Cereals, Other | 168876 | 20042 | Rice, brown, parboiled, dry, Uncle Ben's |  |
| 2520 | Cereals, Other | 168883 | 20054 | Rice, white, glutinous, unenriched, uncooked |  |
| 2520 | Cereals, Other | 168884 | 20062 | Rye grain |  |
| 2520 | Cereals, Other | 168889 | 20071 | Wheat, hard red spring |  |
| 2520 | Cereals, Other | 168890 | 20072 | Wheat, hard red winter |  |
| 2520 | Cereals, Other | 168891 | 20073 | Wheat, soft red winter |  |
| 2520 | Cereals, Other | 168920 | 20314 | Corn grain, white |  |
| 2520 | Cereals, Other | 168931 | 20452 | Rice, white, short-grain, raw, unenriched |  |
| 2520 | Cereals, Other | 169702 | 20031 | Millet, raw |  |
| 2520 | Cereals, Other | 169703 | 20036 | Rice, brown, long-grain, raw (Includes foods for USDA's Food Distribution Program) |  |
| 2520 | Cereals, Other | 169705 | 20038 | Oats |  |
| 2520 | Cereals, Other | 169706 | 20040 | Rice, brown, medium-grain, raw (Includes foods for USDA's Food Distribution Program) | |
| 2520 | Cereals, Other | 169716 | 20067 | Sorghum grain |  |
| 2520 | Cereals, Other | 169718 | 20069 | Triticale |  |
| 2520 | Cereals, Other | 169719 | 20074 | Wheat, hard white |  |
| 2520 | Cereals, Other | 169720 | 20075 | Wheat, soft white |  |
| 2520 | Cereals, Other | 169721 | 20076 | Wheat, durum |  |
| 2520 | Cereals, Other | 169726 | 20088 | Wild rice, raw |  |
| 2520 | Cereals, Other | 169743 | 20138 | Wheat, Kamut khorasan, uncooked |  |
| 2520 | Cereals, Other | 169756 | 20444 | Rice, white, long-grain, regular, raw, unenriched |  |
| 2520 | Cereals, Other | 169758 | 20446 | Rice, white, long-grain, parboiled, unenriched, dry |  |
| 2520 | Cereals, Other | 169760 | 20450 | Rice, white, medium-grain, raw, unenriched |  |
| 2520 | Cereals, Other | 170283 | 20004 | Barley, hulled |  |
| 2520 | Cereals, Other | 170286 | 20008 | Buckwheat |  |
| 2520 | Cereals, Other | 170288 | 20014 | Corn grain, yellow |  |
| 2520 | Cereals, Other | 173904 | 8120 | Cereals, oats, regular and quick, not fortified, dry |  |
| 2531 | Potatoes and products | 170026 | 11352 | Potatoes, flesh and skin, raw |  |
| 2531 | Potatoes and products | 170028 | 11354 | Potatoes, white, flesh and skin, raw |  |
| 2531 | Potatoes and products | 170029 | 11355 | Potatoes, red, flesh and skin, raw |  |
| 2532 | Cassava and products | 169985 | 11134 | Cassava, raw |  |
| 2533 | Sweet potatoes | 168482 | 11507 | Sweet potato, raw, unprepared (Includes foods for USDA's Food Distribution Program) |  |
| 2534 | Sweet potatoes | WAFCT2019 | 02_049 | Sweet potato, orange flesh, raw |  |
| 2534 | Roots, Other | 168432 | 11258 | Mountain yam, hawaii, raw |  |
| 2534 | Roots, Other | 168482 | 11507 | Sweet potato, raw, unprepared (Includes foods for USDA's Food Distribution Program) |  |
| 2534 | Roots, Other | 168490 | 11697 | Arrowroot, raw |  |
| 2534 | Roots, Other | 169236 | 11226 | Jerusalem-artichokes, raw |  |
| 2534 | Roots, Other | 169308 | 11518 | Taro, raw |  |
| 2534 | Roots, Other | 169310 | 11525 | Taro, tahitian, raw |  |
| 2534 | Roots, Other | 169401 | 11991 | Yautia (tannier), raw |  |
| 2534 | Roots, Other | 169985 | 11134 | Cassava, raw |  |
| 2534 | Roots, Other | 170026 | 11352 | Potatoes, flesh and skin, raw |  |
| 2534 | Roots, Other | 170028 | 11354 | Potatoes, white, flesh and skin, raw |  |
| 2534 | Roots, Other | 170029 | 11355 | Potatoes, red, flesh and skin, raw |  |
| 2534 | Roots, Other | 170071 | 11601 | Yam, raw |  |
| 2534 | Roots, Other | 170073 | 11603 | Yambean (jicama), raw |  |
| 2535 | Yams | 168432 | 11258 | Mountain yam, hawaii, raw |  |
| 2535 | Yams | 170071 | 11601 | Yam, raw |  |
| 2536 | Sugar cane | 170674 | 19908 | Sugar, turbinado |  |
| 2537 | Sugar beet | 170674 | 19908 | Sugar, turbinado |  |
| 2541 | Sugar non-centrifugal | 170674 | 19908 | Sugar, turbinado |  |
| 2542 | Sugar (Raw Equivalent) | 170674 | 19908 | Sugar, turbinado |  |
| 2543 | Sweeteners, Other | 168142 | 44018 | Sweeteners, tabletop, fructose, liquid |  |
| 2543 | Sweeteners, Other | 168820 | 19304 | Molasses |  |
| 2543 | Sweeteners, Other | 168836 | 19349 | Syrups, corn, dark |  |
| 2543 | Sweeteners, Other | 168837 | 19350 | Syrups, corn, light |  |
| 2543 | Sweeteners, Other | 169658 | 19340 | Sugars, maple |  |
| 2543 | Sweeteners, Other | 169659 | 19351 | Syrups, corn, high-fructose |  |
| 2543 | Sweeteners, Other | 169660 | 19352 | Syrups, malt |  |
| 2543 | Sweeteners, Other | 169661 | 19353 | Syrups, maple |  |
| 2543 | Sweeteners, Other | 169662 | 19355 | Syrups, sorghum |  |
| 2543 | Sweeteners, Other | 169896 | 43216 | Sweeteners, tabletop, fructose, dry, powder |  |
| 2543 | Sweeteners, Other | 170276 | 19911 | Syrup, maple, Canadian |  |
| 2543 | Sweeteners, Other | 170277 | 19912 | Sweetener, syrup, agave |  |
| 2546 | Beans | 172425 | 16078 | Mothbeans, mature seeds, raw |  |
| 2546 | Beans | 173727 | 16001 | Beans, adzuki, mature seeds, raw |  |
| 2546 | Beans | 173734 | 16014 | Beans, black, mature seeds, raw |  |
| 2546 | Beans | 173738 | 16022 | Beans, french, mature seeds, raw |  |
| 2546 | Beans | 173742 | 16030 | Beans, kidney, california red, mature seeds, raw |  |
| 2546 | Beans | 173744 | 16032 | Beans, kidney, red, mature seeds, raw |  |
| 2546 | Beans | 173745 | 16037 | Beans, navy, mature seeds, raw |  |
| 2546 | Beans | 173748 | 16040 | Beans, pink, mature seeds, raw |  |
| 2546 | Beans | 173749 | 16045 | Beans, small white, mature seeds, raw |  |
| 2546 | Beans | 173751 | 16047 | Beans, yellow, mature seeds, raw |  |
| 2546 | Beans | 174252 | 16071 | Lima beans, large, mature seeds, raw |  |
| 2546 | Beans | 174255 | 16074 | Lima beans, thin seeded (baby), mature seeds, raw |  |
| 2546 | Beans | 174256 | 16080 | Mung beans, mature seeds, raw |  |
| 2546 | Beans | 174259 | 16083 | Mungo beans, mature seeds, raw |  |
| 2546 | Beans | 175186 | 16016 | Beans, black turtle, mature seeds, raw |  |
| 2546 | Beans | 175189 | 16019 | Beans, cranberry (roman), mature seeds, raw |  |
| 2546 | Beans | 175190 | 16024 | Beans, great northern, mature seeds, raw (Includes foods for USDA's Food Distribution Program) |  |
| 2546 | Beans | 175193 | 16027 | Beans, kidney, all types, mature seeds, raw |  |
| 2546 | Beans | 175196 | 16035 | Beans, kidney, royal red, mature seeds, raw |  |
| 2546 | Beans | 175199 | 16042 | Beans, pinto, mature seeds, raw (Includes foods USDA's food distribution program) |  |
| 2546 | Beans | 175202 | 16049 | Beans, white, mature seeds, raw |  |
| 2547 | Peas | 172428 | 16085 | Peas, green, split, mature seeds, raw |  |
| 2549 | Pulses, Other and products | 172420 | 16069 | Lentils, raw |  |
| 2549 | Pulses, Other and products | 172423 | 16076 | lupins, mature seeds, raw |  |
| 2549 | Pulses, Other and products | 172425 | 16078 | Mothbeans, mature seeds, raw |  |
| 2549 | Pulses, Other and products | 172428 | 16085 | Peas, green, split, mature seeds, raw |  |
| 2549 | Pulses, Other and products | 172436 | 16101 | Pigeon peas, (red gram), mature seeds, raw |  |
| 2549 | Pulses, Other and products | 173727 | 16001 | Beans, adzuki, mature seeds, raw |  |
| 2549 | Pulses, Other and products | 173734 | 16014 | Beans, black, mature seeds, raw |  |
| 2549 | Pulses, Other and products | 173738 | 16022 | Beans, french, mature seeds, raw |  |
| 2549 | Pulses, Other and products | 173742 | 16030 | Beans, kidney, california red, mature seeds, raw |  |
| 2549 | Pulses, Other and products | 173744 | 16032 | Beans, kidney, red, mature seeds, raw |  |
| 2549 | Pulses, Other and products | 173745 | 16037 | Beans, navy, mature seeds, raw |  |
| 2549 | Pulses, Other and products | 173748 | 16040 | Beans, pink, mature seeds, raw |  |
| 2549 | Pulses, Other and products | 173749 | 16045 | Beans, small white, mature seeds, raw |  |
| 2549 | Pulses, Other and products | 173751 | 16047 | Beans, yellow, mature seeds, raw |  |
| 2549 | Pulses, Other and products | 173756 | 16056 | Chickpeas (garbanzo beans, bengal gram), mature seeds, raw |  |
| 2549 | Pulses, Other and products | 173758 | 16062 | Cowpeas, common (blackeyes, crowder, southern), mature seeds, raw |  |
| 2549 | Pulses, Other and products | 174252 | 16071 | Lima beans, large, mature seeds, raw |  |
| 2549 | Pulses, Other and products | 174255 | 16074 | Lima beans, thin seeded (baby), mature seeds, raw |  |
| 2549 | Pulses, Other and products | 174256 | 16080 | Mung beans, mature seeds, raw |  |
| 2549 | Pulses, Other and products | 174259 | 16083 | Mungo beans, mature seeds, raw |  |
| 2549 | Pulses, Other and products | 174281 | 16133 | Yardlong beans, mature seeds, raw |  |
| 2549 | Pulses, Other and products | 174283 | 16135 | Winged beans, mature seeds, raw |  |
| 2549 | Pulses, Other and products | 174284 | 16144 | Lentils, pink or red, raw |  |
| 2549 | Pulses, Other and products | 175186 | 16016 | Beans, black turtle, mature seeds, raw |  |
| 2549 | Pulses, Other and products | 175189 | 16019 | Beans, cranberry (roman), mature seeds, raw |  |
| 2549 | Pulses, Other and products | 175190 | 16024 | Beans, great northern, mature seeds, raw (Includes foods for USDA's Food Distribution Program) |  |
| 2549 | Pulses, Other and products | 175193 | 16027 | Beans, kidney, all types, mature seeds, raw |  |
| 2549 | Pulses, Other and products | 175196 | 16035 | Beans, kidney, royal red, mature seeds, raw |  |
| 2549 | Pulses, Other and products | 175199 | 16042 | Beans, pinto, mature seeds, raw (Includes foods USDA's food distribution program) |  |
| 2549 | Pulses, Other and products | 175202 | 16049 | Beans, white, mature seeds, raw |  |
| 2549 | Pulses, Other and products | 175205 | 16052 | Broadbeans (fava beans), mature seeds, raw |  |
| 2549 | Pulses, Other and products | 175208 | 16060 | Cowpeas, catjang, mature seeds, raw |  |
| 2549 | Pulses, Other and products | 175210 | 16067 | Hyacinth beans, mature seeds, raw |  |
| 2551 | Nuts and products | 169408 | 12175 | Nuts, chestnuts, japanese, dried |  |
| 2551 | Nuts and products | 169413 | 12202 | Nuts, chestnuts, japanese, raw |  |
| 2551 | Nuts and products | 170157 | 12058 | Nuts, acorns, raw |  |
| 2551 | Nuts and products | 170162 | 12087 | Nuts, cashew nuts, raw |  |
| 2551 | Nuts and products | 170164 | 12093 | Nuts, chestnuts, chinese, raw |  |
| 2551 | Nuts and products | 170175 | 12128 | Nuts, ginkgo nuts, dried |  |
| 2551 | Nuts and products | 170177 | 12130 | Nuts, hickorynuts, dried |  |
| 2551 | Nuts and products | 170178 | 12131 | Nuts, macadamia nuts, raw |  |
| 2551 | Nuts and products | 170182 | 12142 | Nuts, pecans |  |
| 2551 | Nuts and products | 170184 | 12151 | Nuts, pistachio nuts, raw |  |
| 2551 | Nuts and products | 170186 | 12154 | Nuts, walnuts, black, dried |  |
| 2551 | Nuts and products | 170187 | 12155 | Nuts, walnuts, english |  |
| 2551 | Nuts and products | 170565 | 12059 | Nuts, acorns, dried |  |
| 2551 | Nuts and products | 170567 | 12061 | Nuts, almonds |  |
| 2551 | Nuts and products | 170569 | 12078 | Nuts, brazilnuts, dried, unblanched |  |
| 2551 | Nuts and products | 170570 | 12084 | Nuts, butternuts, dried |  |
| 2551 | Nuts and products | 170574 | 12097 | Nuts, chestnuts, european, raw, unpeeled |  |
| 2551 | Nuts and products | 170576 | 12099 | Nuts, chestnuts, european, dried, unpeeled |  |
| 2551 | Nuts and products | 170581 | 12120 | Nuts, hazelnuts or filberts |  |
| 2551 | Nuts and products | 170584 | 12127 | Nuts, ginkgo nuts, raw |  |
| 2551 | Nuts and products | 170590 | 12145 | Nuts, pilinuts, dried |  |
| 2551 | Nuts and products | 170591 | 12147 | Nuts, pine nuts, dried |  |
| 2551 | Nuts and products | 170592 | 12149 | Nuts, pine nuts, pinyon, dried |  |
| 2555 | Soyabeans | 174270 | 16108 | Soybeans, mature seeds, raw |  |
| 2556 | Groundnuts (Shelled Eq) | 172430 | 16087 | Peanuts, all types, raw |  |
| 2556 | Groundnuts (Shelled Eq) | 172432 | 16093 | Peanuts, valencia, raw |  |
| 2556 | Groundnuts (Shelled Eq) | 172434 | 16095 | Peanuts, virginia, raw |  |
| 2556 | Groundnuts (Shelled Eq) | 174263 | 16091 | Peanuts, spanish, raw |  |
| 2557 | Sunflower seed | 170562 | 12036 | Seeds, sunflower seed kernels, dried |  |
| 2558 | Rape and Mustardseed | 170929 | 2024 | Spices, mustard seed, ground |  |
| 2559 | Cottonseed | 170596 | 12160 | Seeds, cottonseed kernels, roasted (glandless) |  |
| 2560 | Coconuts - Incl Copra | 170169 | 12104 | Nuts, cocounut meat, raw |  |
| 2561 | Sesame seed | 170150 | 12023 | Seeds, sesame seeds, whole, dried |  |
| 2562 | Palm kernels | WAFCT2019 | 06_029 | Oil palm, nut (kernel), shelled, raw |  |
| 2563 | Olives (including preserved) | 169094 | 9193 | Olives, ripe, canned (small-extra large) |  |
| 2563 | Olives (including preserved) | 169095 | 9194 | Olives, ripe, canned (jumbo-super colossal) |  |
| 2570 | Oilcrops, Other | 169094 | 9193 | Olives, ripe, canned (small-extra large) |  |
| 2570 | Oilcrops, Other | 169095 | 9194 | Olives, ripe, canned (jumbo-super colossal) |  |
| 2570 | Oilcrops, Other | 169414 | 12220 | Seeds, flaxseed |  |
| 2570 | Oilcrops, Other | 170148 | 12012 | Seeds, hemp seed, hulled |  |
| 2570 | Oilcrops, Other | 170150 | 12023 | Seeds, sesame seeds, whole, dried |  |
| 2570 | Oilcrops, Other | 170161 | 12077 | Nuts, beechnuts, dried |  |
| 2570 | Oilcrops, Other | 170169 | 12104 | Nuts, cocounut meat, raw |  |
| 2570 | Oilcrops, Other | 170558 | 12021 | Seeds, safflower seed kernels, dried |  |
| 2570 | Oilcrops, Other | 170562 | 12036 | Seeds, sunflower seed kernels, dried |  |
| 2570 | Oilcrops, Other | 170596 | 12160 | Seeds, cottonseed kernels, roasted (glandless) |  |
| 2570 | Oilcrops, Other | 170929 | 2024 | Spices, mustard seed, ground |  |
| 2570 | Oilcrops, Other | 171330 | 2033 | Spices, poppy seed |  |
| 2570 | Oilcrops, Other | 172430 | 16087 | Peanuts, all types, raw |  |
| 2570 | Oilcrops, Other | 172432 | 16093 | Peanuts, valencia, raw |  |
| 2570 | Oilcrops, Other | 172434 | 16095 | Peanuts, virginia, raw |  |
| 2570 | Oilcrops, Other | 174263 | 16091 | Peanuts, spanish, raw |  |
| 2570 | Oilcrops, Other | 174270 | 16108 | Soybeans, mature seeds, raw |  |
| 2571 | Soyabean Oil | 171012 | 4034 | Oil, soybean, salad or cooking, (partially hydrogenated) |  |
| 2571 | Soyabean Oil | 171411 | 4044 | Oil, soybean, salad or cooking |  |
| 2571 | Soyabean Oil | 171438 | 4699 | Oil, industrial, soy, low linolenic |  |
| 2571 | Soyabean Oil | 171439 | 4700 | Oil, industrial, soy, ultra low linolenic |  |
| 2571 | Soyabean Oil | 171440 | 4701 | Oil, industrial, soy, fully hydrogenated |  |
| 2571 | Soyabean Oil | 172362 | 4652 | Oil, industrial, soy ( partially hydrogenated), all purpose |  |
| 2571 | Soyabean Oil | 172370 | 4669 | Oil, vegetable, soybean, refined |  |
| 2571 | Soyabean Oil | 173598 | 4650 | Oil, industrial, soy, refined, for woks and light frying |  |
| 2572 | Groundnut Oil | 171410 | 4042 | Oil, peanut, salad or cooking |  |
| 2573 | Sunflowerseed Oil | 171017 | 4060 | Oil, sunflower, linoleic (less than 60%) |  |
| 2573 | Sunflowerseed Oil | 171025 | 4506 | Oil, sunflower, linoleic, (approx. 65%) |  |
| 2573 | Sunflowerseed Oil | 172328 | 4545 | Oil, sunflower, linoleic, (partially hydrogenated) |  |
| 2573 | Sunflowerseed Oil | 172338 | 4584 | Oil, sunflower, high oleic (70% and over) |  |
| 2573 | Sunflowerseed Oil | 172357 | 4642 | Oil, industrial, mid-oleic, sunflower |  |
| 2574 | Rape and Mustard Oil | 171033 | 4678 | Oil, vegetable, Natreon canola, high stability, non trans, high oleic (70%) |  |
| 2574 | Rape and Mustard Oil | 171042 | 4698 | Oil, industrial, canola, high oleic |  |
| 2574 | Rape and Mustard Oil | 172336 | 4582 | Oil, canola |  |
| 2574 | Rape and Mustard Oil | 172337 | 4583 | Oil, mustard |  |
| 2574 | Rape and Mustard Oil | 172359 | 4644 | Oil, industrial, canola for salads, woks and light frying |  |
| 2574 | Rape and Mustard Oil | 172360 | 4645 | Oil, industrial, canola (partially hydrogenated) oil for deep fat frying |  |
| 2575 | Cottonseed Oil | 171024 | 4502 | Oil, cottonseed, salad or cooking |  |
| 2575 | Cottonseed Oil | 171441 | 4702 | Oil, industrial, cottonseed, fully hydrogenated |  |
| 2576 | Palmkernel Oil | 171422 | 4513 | Vegetable oil, palm kernel |  |
| 2576 | Palmkernel Oil | 171428 | 4534 | Oil, babassu |  |
| 2576 | Palmkernel Oil | 172366 | 4660 | Oil, industrial, palm kernel (hydrogenated) , used for whipped toppings, non-dairy |  |
| 2576 | Palmkernel Oil | 173600 | 4656 | Oil, industrial, palm kernel, confection fat, uses similar to high quality cocoa butter |  |
| 2576 | Palmkernel Oil | 173601 | 4657 | Oil, industrial, palm kernel (hydrogenated), confection fat, uses similar to 95 degree hard butter |  |
| 2576 | Palmkernel Oil | 173602 | 4658 | Oil, industrial, palm kernel (hydrogenated), confection fat, intermediate grade product |  |
| 2576 | Palmkernel Oil | 173603 | 4663 | Oil, industrial, palm kernel (hydrogenated), filling fat |  |
| 2577 | Palm Oil | 171015 | 4055 | Oil, palm |  |
| 2577 | Palm Oil | WAFCT2019 | 11_004 | Palm oil, red |  |
| 2578 | Coconut Oil | 171412 | 4047 | Oil, coconut |  |
| 2578 | Coconut Oil | 172365 | 4659 | Oil, industrial, coconut, confection fat, typical basis for ice cream coatings |  |
| 2578 | Coconut Oil | 172367 | 4661 | Oil, industrial, coconut (hydrogenated), used for whipped toppings and coffee whiteners |  |
| 2578 | Coconut Oil | 173595 | 4646 | Oil, industrial, coconut, principal uses candy coatings, oil sprays, roasting nuts |  |
| 2579 | Sesameseed Oil | 171016 | 4058 | Oil, sesame, salad or cooking |  |
| 2580 | Olive Oil | 171413 | 4053 | Oil, olive, salad or cooking |  |
| 2581 | Ricebran Oil | 171013 | 4037 | Oil, rice bran |  |
| 2582 | Maize Germ Oil | 171029 | 4518 | Oil, corn, industrial and retail, all purpose salad or cooking |  |
| 2586 | Oilcrops Oil, Other | 167702 | 42231 | Oil, flaxseed, cold pressed |  |
| 2586 | Oilcrops Oil, Other | 171011 | 4031 | Shortening, household, soybean (partially hydrogenated)-cottonseed (partially hydrogenated) |  |
| 2586 | Oilcrops Oil, Other | 171014 | 4038 | Oil, wheat germ |  |
| 2586 | Oilcrops Oil, Other | 171018 | 4073 | Margarine, regular, hard, soybean (hydrogenated) |  |
| 2586 | Oilcrops Oil, Other | 171026 | 4510 | Oil, safflower, salad or cooking, linoleic, (over 70%) |  |
| 2586 | Oilcrops Oil, Other | 171027 | 4511 | Oil, safflower, salad or cooking, high oleic (primary safflower oil of commerce) |  |
| 2586 | Oilcrops Oil, Other | 171028 | 4517 | Oil, grapeseed |  |
| 2586 | Oilcrops Oil, Other | 171030 | 4528 | Oil, walnut |  |
| 2586 | Oilcrops Oil, Other | 171031 | 4529 | Oil, almond |  |
| 2586 | Oilcrops Oil, Other | 171032 | 4530 | Oil, apricot kernel |  |
| 2586 | Oilcrops Oil, Other | 171421 | 4501 | Oil, cocoa butter |  |
| 2586 | Oilcrops Oil, Other | 171423 | 4514 | Oil, poppyseed |  |
| 2586 | Oilcrops Oil, Other | 171424 | 4515 | Oil, tomatoseed |  |
| 2586 | Oilcrops Oil, Other | 171425 | 4516 | Oil, teaseed |  |
| 2586 | Oilcrops Oil, Other | 171427 | 4532 | Oil, hazelnut |  |
| 2586 | Oilcrops Oil, Other | 171429 | 4536 | Oil, sheanut |  |
| 2586 | Oilcrops Oil, Other | 171432 | 4684 | Margarine, 80% fat, tub, CANOLA HARVEST Soft Spread (canola, palm and palm kernel oils) |  |
| 2586 | Oilcrops Oil, Other | 172329 | 4546 | Shortening bread, soybean (hydrogenated) and cottonseed |  |
| 2586 | Oilcrops Oil, Other | 172330 | 4548 | Shortening cake mix, soybean (hydrogenated) and cottonseed (hydrogenated) |  |
| 2586 | Oilcrops Oil, Other | 172331 | 4556 | Shortening frying (heavy duty), palm (hydrogenated) |  |
| 2586 | Oilcrops Oil, Other | 172332 | 4559 | Shortening household soybean (hydrogenated) and palm |  |
| 2586 | Oilcrops Oil, Other | 172333 | 4560 | Shortening frying (heavy duty), soybean (hydrogenated), linoleic (less than 1%) |  |
| 2586 | Oilcrops Oil, Other | 172334 | 4570 | Shortening, confectionery, fractionated palm |  |
| 2586 | Oilcrops Oil, Other | 172335 | 4572 | Oil, nutmeg butter |  |
| 2586 | Oilcrops Oil, Other | 172349 | 4618 | Margarine, regular, 80% fat, composite, tub, without salt |  |
| 2586 | Oilcrops Oil, Other | 172369 | 4668 | Margarine, industrial, soy and partially hydrogenated soy oil, use for baking, sauces and candy |  |
| 2586 | Oilcrops Oil, Other | 173563 | 4541 | Oil, cupu assu |  |
| 2586 | Oilcrops Oil, Other | 173568 | 4551 | Shortening confectionery, coconut (hydrogenated) and or palm kernel (hydrogenated) |  |
| 2586 | Oilcrops Oil, Other | 173569 | 4554 | Shortening industrial, soybean (hydrogenated) and cottonseed |  |
| 2586 | Oilcrops Oil, Other | 173570 | 4573 | Oil, ucuhuba butter |  |
| 2586 | Oilcrops Oil, Other | 173573 | 4581 | Oil, avocado |  |
| 2586 | Oilcrops Oil, Other | 173574 | 4586 | Shortening, special purpose for cakes and frostings, soybean (hydrogenated) |  |
| 2586 | Oilcrops Oil, Other | 173575 | 4587 | Shortening, special purpose for baking, soybean (hydrogenated) palm and cottonseed |  |
| 2586 | Oilcrops Oil, Other | 173579 | 4595 | Shortening, multipurpose, soybean (hydrogenated) and palm (hydrogenated) |  |
| 2586 | Oilcrops Oil, Other | 173584 | 4615 | Shortening, vegetable, household, composite |  |
| 2586 | Oilcrops Oil, Other | 173585 | 4617 | Margarine, regular, 80% fat, composite, stick, without salt |  |
| 2586 | Oilcrops Oil, Other | 173587 | 4628 | Margarine, 80% fat, stick, includes regular and hydrogenated corn and soybean oils |  |
| 2586 | Oilcrops Oil, Other | 173599 | 4655 | Margarine-like shortening, industrial, soy (partially hydrogenated), cottonseed, and soy, principal use flaky pastries |  |
| 2586 | Oilcrops Oil, Other | 173605 | 4665 | Margarine, industrial, non-dairy, cottonseed, soy oil (partially hydrogenated ), for flaky pastries |  |
| 2586 | Oilcrops Oil, Other | 173606 | 4666 | Shortening, industrial, soy (partially hydrogenated ) and corn for frying |  |
| 2586 | Oilcrops Oil, Other | 173607 | 4667 | Shortening, industrial, soy (partially hydrogenated ) for baking and confections |  |
| 2601 | Tomatoes and products | 170096 | 11696 | Tomatoes, yellow, raw |  |
| 2601 | Tomatoes and products | 170456 | 11527 | Tomatoes, green, raw |  |
| 2601 | Tomatoes and products | 170457 | 11529 | Tomatoes, red, ripe, raw, year round average |  |
| 2601 | Tomatoes and products | 170502 | 11695 | Tomatoes, orange, raw |  |
| 2601 | Tomatoes and products | 321360 | 100147 | Tomatoes, grape, raw |  |
| 2602 | Onions | 170000 | 11282 | Onions, raw |  |
| 2602 | Onions | 170008 | 11294 | Onions, sweet, raw |  |
| 2605 | Vegetables, Other | 167758 | 9307 | Rhubarb, raw |  |
| 2605 | Vegetables, Other | 167765 | 9326 | Watermelon, raw |  |
| 2605 | Vegetables, Other | 168389 | 11011 | Asparagus, raw |  |
| 2605 | Vegetables, Other | 168396 | 11031 | Lima beans, immature seeds, raw |  |
| 2605 | Vegetables, Other | 168405 | 11197 | Cowpeas, young pods with seeds, raw |  |
| 2605 | Vegetables, Other | 168407 | 11203 | Cress, garden, raw |  |
| 2605 | Vegetables, Other | 168409 | 11205 | Cucumber, with peel, raw |  |
| 2605 | Vegetables, Other | 168412 | 11213 | Endive, raw |  |
| 2605 | Vegetables, Other | 168421 | 11233 | Kale, raw |  |
| 2605 | Vegetables, Other | 168422 | 11239 | Mushrooms, Chanterelle, raw |  |
| 2605 | Vegetables, Other | 168423 | 11240 | Mushrooms, morel, raw |  |
| 2605 | Vegetables, Other | 168424 | 11241 | Kohlrabi, raw |  |
| 2605 | Vegetables, Other | 168429 | 11250 | Lettuce, butterhead (includes boston and bibb types), raw |  |
| 2605 | Vegetables, Other | 168431 | 11257 | Lettuce, red leaf, raw |  |
| 2605 | Vegetables, Other | 168434 | 11266 | Mushrooms, brown, italian, or crimini, raw |  |
| 2605 | Vegetables, Other | 168438 | 11274 | Mustard spinach, (tendergreen), raw |  |
| 2605 | Vegetables, Other | 168440 | 11276 | New Zealand spinach, raw |  |
| 2605 | Vegetables, Other | 168448 | 11422 | Pumpkin, raw |  |
| 2605 | Vegetables, Other | 168451 | 11430 | Radishes, oriental, raw |  |
| 2605 | Vegetables, Other | 168454 | 11435 | Rutabagas, raw |  |
| 2605 | Vegetables, Other | 168462 | 11457 | Spinach, raw |  |
| 2605 | Vegetables, Other | 168538 | 11900 | Corn, sweet, white, raw |  |
| 2605 | Vegetables, Other | 168565 | 11953 | Squash, zucchini, baby, raw |  |
| 2605 | Vegetables, Other | 168568 | 11960 | Carrots, baby, raw |  |
| 2605 | Vegetables, Other | 168574 | 11973 | Beans, fava, in pod, raw |  |
| 2605 | Vegetables, Other | 168576 | 11979 | Peppers, jalapeno, raw |  |
| 2605 | Vegetables, Other | 168580 | 11987 | Mushrooms, oyster, raw |  |
| 2605 | Vegetables, Other | 169092 | 9181 | Melons, cantaloupe, raw |  |
| 2605 | Vegetables, Other | 169093 | 9183 | Melons, casaba, raw |  |
| 2605 | Vegetables, Other | 169145 | 11080 | Beets, raw |  |
| 2605 | Vegetables, Other | 169205 | 11007 | Artichokes, (globe or french), raw |  |
| 2605 | Vegetables, Other | 169210 | 11026 | Bamboo shoots, raw |  |
| 2605 | Vegetables, Other | 169220 | 11191 | Cowpeas (blackeyes), immature seeds, raw |  |
| 2605 | Vegetables, Other | 169222 | 11199 | Yardlong bean, raw |  |
| 2605 | Vegetables, Other | 169228 | 11209 | Eggplant, raw |  |
| 2605 | Vegetables, Other | 169230 | 11215 | Garlic, raw |  |
| 2605 | Vegetables, Other | 169242 | 11238 | Mushrooms, shiitake, raw |  |
| 2605 | Vegetables, Other | 169246 | 11246 | Leeks, (bulb and lower leaf-portion), raw |  |
| 2605 | Vegetables, Other | 169247 | 11251 | Lettuce, cos or romaine, raw |  |
| 2605 | Vegetables, Other | 169248 | 11252 | Lettuce, iceberg (includes crisphead types), raw |  |
| 2605 | Vegetables, Other | 169249 | 11253 | Lettuce, green leaf, raw |  |
| 2605 | Vegetables, Other | 169251 | 11260 | Mushrooms, white, raw |  |
| 2605 | Vegetables, Other | 169255 | 11265 | Mushrooms, portabella, raw |  |
| 2605 | Vegetables, Other | 169256 | 11270 | Mustard greens, raw |  |
| 2605 | Vegetables, Other | 169260 | 11278 | Okra, raw |  |
| 2605 | Vegetables, Other | 169276 | 11429 | Radishes, raw |  |
| 2605 | Vegetables, Other | 169277 | 11437 | Salsify, (vegetable oyster), raw |  |
| 2605 | Vegetables, Other | 169320 | 11722 | Beans, snap, yellow, raw |  |
| 2605 | Vegetables, Other | 169335 | 11749 | Cabbage, common (danish, domestic, and pointed types), freshly harvest, raw |  |
| 2605 | Vegetables, Other | 169382 | 11950 | Mushrooms, enoki, raw |  |
| 2605 | Vegetables, Other | 169383 | 11951 | Peppers, sweet, yellow, raw |  |
| 2605 | Vegetables, Other | 169385 | 11957 | Fennel, bulb, raw |  |
| 2605 | Vegetables, Other | 169389 | 11965 | Cauliflower, green, raw |  |
| 2605 | Vegetables, Other | 169394 | 11976 | Pepper, banana, raw |  |
| 2605 | Vegetables, Other | 169395 | 11977 | Peppers, serrano, raw |  |
| 2605 | Vegetables, Other | 169403 | 11993 | Mushrooms, maitake, raw |  |
| 2605 | Vegetables, Other | 169404 | 11994 | Broccoli, chinese, raw |  |
| 2605 | Vegetables, Other | 169911 | 9184 | Melons, honeydew, raw |  |
| 2605 | Vegetables, Other | 169961 | 11052 | Beans, snap, green, raw |  |
| 2605 | Vegetables, Other | 169975 | 11109 | Cabbage, raw |  |
| 2605 | Vegetables, Other | 169977 | 11112 | Cabbage, red, raw |  |
| 2605 | Vegetables, Other | 169979 | 11119 | Cabbage, chinese (pe-tsai), raw |  |
| 2605 | Vegetables, Other | 169981 | 11122 | Cardoon, raw |  |
| 2605 | Vegetables, Other | 169986 | 11135 | Cauliflower, raw |  |
| 2605 | Vegetables, Other | 169988 | 11143 | Celery, raw |  |
| 2605 | Vegetables, Other | 169990 | 11145 | Celtuce, raw |  |
| 2605 | Vegetables, Other | 169991 | 11147 | Chard, swiss, raw |  |
| 2605 | Vegetables, Other | 169992 | 11152 | Chicory greens, raw |  |
| 2605 | Vegetables, Other | 169993 | 11154 | Chicory roots, raw |  |
| 2605 | Vegetables, Other | 169994 | 11156 | Chives, raw |  |
| 2605 | Vegetables, Other | 169998 | 11167 | Corn, sweet, yellow, raw |  |
| 2605 | Vegetables, Other | 170000 | 11282 | Onions, raw |  |
| 2605 | Vegetables, Other | 170005 | 11291 | Onions, spring or scallions (includes tops and bulb), raw |  |
| 2605 | Vegetables, Other | 170006 | 11292 | Onions, young green, tops only |  |
| 2605 | Vegetables, Other | 170007 | 11293 | Onions, welsh, raw |  |
| 2605 | Vegetables, Other | 170008 | 11294 | Onions, sweet, raw |  |
| 2605 | Vegetables, Other | 170010 | 11300 | Peas, edible-podded, raw |  |
| 2605 | Vegetables, Other | 170061 | 11568 | Turnip greens, raw |  |
| 2605 | Vegetables, Other | 170068 | 11591 | Watercress, raw |  |
| 2605 | Vegetables, Other | 170081 | 11637 | Radishes, white icicle, raw |  |
| 2605 | Vegetables, Other | 170096 | 11696 | Tomatoes, yellow, raw |  |
| 2605 | Vegetables, Other | 170375 | 11086 | Beet greens, raw |  |
| 2605 | Vegetables, Other | 170377 | 11088 | Broadbeans, immature seeds, raw |  |
| 2605 | Vegetables, Other | 170379 | 11090 | Broccoli, raw |  |
| 2605 | Vegetables, Other | 170383 | 11098 | Brussels sprouts, raw |  |
| 2605 | Vegetables, Other | 170388 | 11114 | Cabbage, savoy, raw |  |
| 2605 | Vegetables, Other | 170390 | 11116 | Cabbage, chinese (pak-choi), raw |  |
| 2605 | Vegetables, Other | 170392 | 11118 | Cabbage, kimchi |  |
| 2605 | Vegetables, Other | 170393 | 11124 | Carrots, raw |  |
| 2605 | Vegetables, Other | 170400 | 11141 | Celeriac, raw |  |
| 2605 | Vegetables, Other | 170404 | 11151 | Chicory, witloof, raw |  |
| 2605 | Vegetables, Other | 170406 | 11161 | Collards, raw |  |
| 2605 | Vegetables, Other | 170416 | 11297 | Parsley, fresh |  |
| 2605 | Vegetables, Other | 170417 | 11298 | Parsnips, raw |  |
| 2605 | Vegetables, Other | 170419 | 11304 | Peas, green, raw |  |
| 2605 | Vegetables, Other | 170427 | 11333 | Peppers, sweet, green, raw |  |
| 2605 | Vegetables, Other | 170456 | 11527 | Tomatoes, green, raw |  |
| 2605 | Vegetables, Other | 170457 | 11529 | Tomatoes, red, ripe, raw, year round average |  |
| 2605 | Vegetables, Other | 170465 | 11564 | Turnips, raw |  |
| 2605 | Vegetables, Other | 170487 | 11641 | Squash, summer, all varieties, raw |  |
| 2605 | Vegetables, Other | 170489 | 11643 | Squash, winter, all varieties, raw |  |
| 2605 | Vegetables, Other | 170497 | 11670 | Peppers, hot chili, green, raw |  |
| 2605 | Vegetables, Other | 170499 | 11677 | Shallots, raw |  |
| 2605 | Vegetables, Other | 170502 | 11695 | Tomatoes, orange, raw |  |
| 2605 | Vegetables, Other | 172238 | 2054 | Capers, canned |  |
| 2605 | Vegetables, Other | 321360 | 100147 | Tomatoes, grape, raw |  |
| 2611 | Oranges, Mandarines | 168195 | 9433 | Clementines, raw |  |
| 2611 | Oranges, Mandarines | 169097 | 9200 | Oranges, raw, all commercial varieties |  |
| 2611 | Oranges, Mandarines | 169105 | 9218 | Tangerines, (mandarin oranges), raw |  |
| 2612 | Lemons, Limes and products | 167746 | 9150 | Lemons, raw, without peel |  |
| 2612 | Lemons, Limes and products | 168155 | 9159 | Limes, raw |  |
| 2613 | Grapefruit and products | 167754 | 9295 | Pummelo, raw |  |
| 2613 | Grapefruit and products | 173033 | 9111 | Grapefruit, raw, pink and red and white, all areas |  |
| 2614 | Citrus, Other | 168154 | 9149 | Kumquats, raw |  |
| 2615 | Bananas | 173944 | 9040 | Bananas, raw |  |
| 2616 | Plantains | 168215 | 9542 | Plantains, green, raw |  |
| 2616 | Plantains | 169130 | 9277 | Plantains, yellow, raw |  |
| 2617 | Apples and products | 171688 | 9003 | Apples, raw, with skin (Includes foods for USDA's Food Distribution Program) |  |
| 2618 | Pineapples and products | 169124 | 9266 | Pineapple, raw, all varieties |  |
| 2619 | Dates | 168191 | 9421 | Dates, medjool |  |
| 2619 | Dates | 171726 | 9087 | Dates, deglet noor |  |
| 2620 | Grapes and products (excl wine) | 173040 | 9129 | Grapes, muscadine, raw |  |
| 2620 | Grapes and products (excl wine) | 174682 | 9131 | Grapes, american type (slip skin), raw |  |
| 2620 | Grapes and products (excl wine) | 174683 | 9132 | Grapes, red or green (European type, such as Thompson seedless), raw |  |
| 2625 | Fruits, Other | 167640 | 35155 | Blueberries, wild, raw (Alaska Native) |  |
| 2625 | Fruits, Other | 167746 | 9150 | Lemons, raw, without peel |  |
| 2625 | Fruits, Other | 167750 | 9287 | Prickly pears, raw |  |
| 2625 | Fruits, Other | 167754 | 9295 | Pummelo, raw |  |
| 2625 | Fruits, Other | 167755 | 9302 | Raspberries, raw |  |
| 2625 | Fruits, Other | 167759 | 9313 | Sapodilla, raw |  |
| 2625 | Fruits, Other | 167760 | 9314 | Sapote, mamey, raw |  |
| 2625 | Fruits, Other | 167762 | 9316 | Strawberries, raw |  |
| 2625 | Fruits, Other | 167763 | 9322 | Tamarinds, raw |  |
| 2625 | Fruits, Other | 167790 | 9450 | Naranjilla (lulo) pulp, frozen, unsweetened |  |
| 2625 | Fruits, Other | 168153 | 9148 | Kiwifruit, green, raw |  |
| 2625 | Fruits, Other | 168154 | 9149 | Kumquats, raw |  |
| 2625 | Fruits, Other | 168155 | 9159 | Limes, raw |  |
| 2625 | Fruits, Other | 168163 | 9296 | Quinces, raw |  |
| 2625 | Fruits, Other | 168176 | 9334 | Feijoa, raw |  |
| 2625 | Fruits, Other | 168191 | 9421 | Dates, medjool |  |
| 2625 | Fruits, Other | 168192 | 9422 | Durian, raw or frozen |  |
| 2625 | Fruits, Other | 168195 | 9433 | Clementines, raw |  |
| 2625 | Fruits, Other | 168211 | 9520 | Kiwifruit, ZESPRI SunGold, raw |  |
| 2625 | Fruits, Other | 168215 | 9542 | Plantains, green, raw |  |
| 2625 | Fruits, Other | 168982 | 35015 | Blackberries, wild, raw (Alaska Native) |  |
| 2625 | Fruits, Other | 168998 | 35203 | Rose Hips, wild (Northern Plains Indians) |  |
| 2625 | Fruits, Other | 169086 | 9164 | Litchis, raw |  |
| 2625 | Fruits, Other | 169088 | 9167 | Loganberries, frozen |  |
| 2625 | Fruits, Other | 169089 | 9172 | Longans, raw |  |
| 2625 | Fruits, Other | 169097 | 9200 | Oranges, raw, all commercial varieties |  |
| 2625 | Fruits, Other | 169105 | 9218 | Tangerines, (mandarin oranges), raw |  |
| 2625 | Fruits, Other | 169108 | 9231 | Passion-fruit, (granadilla), purple, raw |  |
| 2625 | Fruits, Other | 169118 | 9252 | Pears, raw |  |
| 2625 | Fruits, Other | 169124 | 9266 | Pineapple, raw, all varieties |  |
| 2625 | Fruits, Other | 169130 | 9277 | Plantains, yellow, raw |  |
| 2625 | Fruits, Other | 169134 | 9286 | Pomegranates, raw |  |
| 2625 | Fruits, Other | 169805 | 35030 | Cranberry, low bush or lingenberry, raw (Alaska Native) |  |
| 2625 | Fruits, Other | 169808 | 35043 | Huckleberries, raw (Alaska Native) |  |
| 2625 | Fruits, Other | 169908 | 9174 | Loquats, raw |  |
| 2625 | Fruits, Other | 169909 | 9175 | Mammy-apple, (mamey), raw |  |
| 2625 | Fruits, Other | 169910 | 9176 | Mangos, raw |  |
| 2625 | Fruits, Other | 169913 | 9190 | Mulberries, raw |  |
| 2625 | Fruits, Other | 169914 | 9191 | Nectarines, raw |  |
| 2625 | Fruits, Other | 169915 | 9192 | Oheloberries, raw |  |
| 2625 | Fruits, Other | 169926 | 9226 | Papayas, raw |  |
| 2625 | Fruits, Other | 169928 | 9236 | Peaches, yellow, raw |  |
| 2625 | Fruits, Other | 169941 | 9263 | Persimmons, japanese, raw |  |
| 2625 | Fruits, Other | 169943 | 9265 | Persimmons, native, raw |  |
| 2625 | Fruits, Other | 169949 | 9279 | Plums, raw |  |
| 2625 | Fruits, Other | 171688 | 9003 | Apples, raw, with skin (Includes foods for USDA's Food Distribution Program) |  |
| 2625 | Fruits, Other | 171697 | 9021 | Apricots, raw |  |
| 2625 | Fruits, Other | 171705 | 9037 | Avocados, raw, all commercial varieties |  |
| 2625 | Fruits, Other | 171711 | 9050 | Blueberries, raw |  |
| 2625 | Fruits, Other | 171714 | 9059 | Breadfruit, raw |  |
| 2625 | Fruits, Other | 171715 | 9060 | Carambola, (starfruit), raw |  |
| 2625 | Fruits, Other | 171719 | 9070 | Cherries, sweet, raw |  |
| 2625 | Fruits, Other | 171722 | 9078 | Cranberries, raw |  |
| 2625 | Fruits, Other | 171725 | 9086 | Custard-apple, (bullock's-heart), raw |  |
| 2625 | Fruits, Other | 171726 | 9087 | Dates, deglet noor |  |
| 2625 | Fruits, Other | 171727 | 9088 | Elderberries, raw |  |
| 2625 | Fruits, Other | 173021 | 9089 | Figs, raw |  |
| 2625 | Fruits, Other | 173030 | 9107 | Gooseberries, raw |  |
| 2625 | Fruits, Other | 173033 | 9111 | Grapefruit, raw, pink and red and white, all areas |  |
| 2625 | Fruits, Other | 173040 | 9129 | Grapes, muscadine, raw |  |
| 2625 | Fruits, Other | 173044 | 9139 | Guavas, common, raw |  |
| 2625 | Fruits, Other | 173944 | 9040 | Bananas, raw |  |
| 2625 | Fruits, Other | 173946 | 9042 | Blackberries, raw |  |
| 2625 | Fruits, Other | 173953 | 9062 | Cherimoya, raw |  |
| 2625 | Fruits, Other | 173954 | 9063 | Cherries, sour, red, raw |  |
| 2625 | Fruits, Other | 173963 | 9083 | Currants, european black, raw |  |
| 2625 | Fruits, Other | 173964 | 9084 | Currants, red and white, raw |  |
| 2625 | Fruits, Other | 174682 | 9131 | Grapes, american type (slip skin), raw |  |
| 2625 | Fruits, Other | 174683 | 9132 | Grapes, red or green (European type, such as Thompson seedless), raw |  |
| 2625 | Fruits, Other | 174687 | 9144 | Jackfruit, raw |  |
| 2630 | Coffee and products | 171890 | 14209 | Beverages, coffee, brewed, prepared with tap water |  |
| 2633 | Cocoa Beans and products | 169593 | 19165 | Cocoa, dry powder, unsweetened |  |
| 2633 | Cocoa Beans and products | 171421 | 4501 | Oil, cocoa butter |  |
| 2635 | Tea (including mate) | 171917 | 14278 | Beverages, tea, green, brewed, regular |  |
| 2635 | Tea (including mate) | 174120 | 14185 | Beverages, tea, Oolong, brewed |  |
| 2635 | Tea (including mate) | 174155 | 14544 | Beverages, tea, black, brewed, prepared with distilled water |  |
| 2640 | Pepper | 170931 | 2030 | Spices, pepper, black |  |
| 2640 | Pepper | 170933 | 2032 | Spices, pepper, white |  |
| 2641 | Pimento | 170932 | 2031 | Spices, pepper, red or cayenne |  |
| 2641 | Pimento | 171315 | 2001 | Spices, allspice, ground |  |
| 2641 | Pimento | 171319 | 2009 | Spices, chili powder |  |
| 2641 | Pimento | 171329 | 2028 | Spices, paprika |  |
| 2642 | Cloves | 171321 | 2011 | Spices, cloves, ground |  |
| 2645 | Spices, Other | 169231 | 11216 | Ginger root, raw |  |
| 2645 | Spices, Other | 170917 | 2004 | Spices, bay leaf |  |
| 2645 | Spices, Other | 170918 | 2005 | Spices, caraway seed |  |
| 2645 | Spices, Other | 170919 | 2006 | Spices, cardamom |  |
| 2645 | Spices, Other | 170920 | 2007 | Spices, celery seed |  |
| 2645 | Spices, Other | 170921 | 2012 | Spices, coriander leaf, dried |  |
| 2645 | Spices, Other | 170922 | 2013 | Spices, coriander seed |  |
| 2645 | Spices, Other | 170923 | 2014 | Spices, cumin seed |  |
| 2645 | Spices, Other | 170924 | 2015 | Spices, curry powder |  |
| 2645 | Spices, Other | 170925 | 2016 | Spices, dill seed |  |
| 2645 | Spices, Other | 170926 | 2021 | Spices, ginger, ground |  |
| 2645 | Spices, Other | 170927 | 2022 | Spices, mace, ground |  |
| 2645 | Spices, Other | 170928 | 2023 | Spices, marjoram, dried |  |
| 2645 | Spices, Other | 170930 | 2029 | Spices, parsley, dried |  |
| 2645 | Spices, Other | 170931 | 2030 | Spices, pepper, black |  |
| 2645 | Spices, Other | 170932 | 2031 | Spices, pepper, red or cayenne |  |
| 2645 | Spices, Other | 170933 | 2032 | Spices, pepper, white |  |
| 2645 | Spices, Other | 170934 | 2037 | Spices, saffron |  |
| 2645 | Spices, Other | 170935 | 2038 | Spices, sage, ground |  |
| 2645 | Spices, Other | 170936 | 2039 | Spices, savory, ground |  |
| 2645 | Spices, Other | 170937 | 2041 | Spices, tarragon, dried |  |
| 2645 | Spices, Other | 170938 | 2042 | Spices, thyme, dried |  |
| 2645 | Spices, Other | 171315 | 2001 | Spices, allspice, ground |  |
| 2645 | Spices, Other | 171316 | 2002 | Spices, anise seed |  |
| 2645 | Spices, Other | 171317 | 2003 | Spices, basil, dried |  |
| 2645 | Spices, Other | 171318 | 2008 | Spices, chervil, dried |  |
| 2645 | Spices, Other | 171319 | 2009 | Spices, chili powder |  |
| 2645 | Spices, Other | 171320 | 2010 | Spices, cinnamon, ground |  |
| 2645 | Spices, Other | 171321 | 2011 | Spices, cloves, ground |  |
| 2645 | Spices, Other | 171322 | 2017 | Spices, dill weed, dried |  |
| 2645 | Spices, Other | 171323 | 2018 | Spices, fennel seed |  |
| 2645 | Spices, Other | 171324 | 2019 | Spices, fenugreek seed |  |
| 2645 | Spices, Other | 171325 | 2020 | Spices, garlic powder |  |
| 2645 | Spices, Other | 171326 | 2025 | Spices, nutmeg, ground |  |
| 2645 | Spices, Other | 171327 | 2026 | Spices, onion powder |  |
| 2645 | Spices, Other | 171328 | 2027 | Spices, oregano, dried |  |
| 2645 | Spices, Other | 171329 | 2028 | Spices, paprika |  |
| 2645 | Spices, Other | 171331 | 2034 | Spices, poultry seasoning |  |
| 2645 | Spices, Other | 171332 | 2035 | Spices, pumpkin pie spice |  |
| 2645 | Spices, Other | 171333 | 2036 | Spices, rosemary, dried |  |
| 2645 | Spices, Other | 172231 | 2043 | Spices, turmeric, ground |  |
| 2655 | Wine | 173176 | 14057 | Alcoholic beverage, wine, dessert, sweet |  |
| 2655 | Wine | 173185 | 14084 | Alcoholic beverage, wine, table, all |  |
| 2655 | Wine | 174157 | 14553 | Beverages, Wine, non-alcoholic |  |
| 2655 | Wine | 175112 | 14536 | Alcoholic beverage, wine, dessert, dry |  |
| 2656 | Beer | 168746 | 14003 | Alcoholic beverage, beer, regular, all |  |
| 2656 | Beer | 168749 | 14006 | Alcoholic beverage, beer, light |  |
| 2656 | Beer | 169575 | 14013 | Alcoholic beverage, beer, light, low carb |  |
| 2656 | Beer | 171906 | 14251 | Alcoholic beverages, beer, higher alcohol |  |
| 2656 | Beer | 171907 | 14252 | Beverages, Malt liquor beverage |  |
| 2656 | Beer | 174141 | 14239 | Alcoholic beverage, malt beer, hard lemonade |  |
| 2656 | Beer | 174145 | 14248 | Alcoholic beverage, beer, light, higher alcohol |  |
| 2656 | Beer | 174863 | 14305 | Malt beverage, includes non-alcoholic beer |  |
| 2657 | Beverages, Fermented | 167723 | 43479 | Alcoholic beverage, rice (sake) |  |
| 2657 | Beverages, Fermented | 174146 | 14250 | Beverages, AMBER, hard cider |  |
| 2658 | Beverages, Alcoholic | 171919 | 14550 | Alcoholic beverage, distilled, all (gin, rum, vodka, whiskey) 86 proof |  |
| 2658 | Beverages, Alcoholic | 171920 | 14551 | Alcoholic beverage, distilled, all (gin, rum, vodka, whiskey) 90 proof |  |
| 2658 | Beverages, Alcoholic | 173168 | 14034 | Alcoholic beverage, creme de menthe, 72 proof |  |
| 2658 | Beverages, Alcoholic | 173663 | 14532 | Alcoholic beverage, distilled, all (gin, rum, vodka, whiskey) 94 proof |  |
| 2658 | Beverages, Alcoholic | 173664 | 14533 | Alcoholic beverage, distilled, all (gin, rum, vodka, whiskey) 100 proof |  |
| 2658 | Beverages, Alcoholic | 174815 | 14037 | Alcoholic beverage, distilled, all (gin, rum, vodka, whiskey) 80 proof |  |
| 2680 | Infant food | 168968 | 33864 | Infant Formula, MEAD JOHNSON, ENFAMIL, Premium LIPIL, Infant, Liquid concentrate, not reconstituted |  |
| 2680 | Infant food | 168969 | 33865 | Infant Formula, MEAD JOHNSON, ENFAMIL, Premium, Infant, Liquid concentrate, not reconstituted |  |
| 2680 | Infant food | 168974 | 33874 | Infant formula, MEAD JOHNSON, ENFAMIL, Premature, 20 calories ready-to-feed Low iron |  |
| 2680 | Infant food | 169789 | 33875 | Infant formula, MEAD JOHNSON, ENFAMIL, Premature, 24 calories ready-to-feed Low iron |  |
| 2680 | Infant food | 169790 | 33876 | Infant Formula, MEAD JOHNSON, ENFAMIL, Premium, Infant, ready-to-feed |  |
| 2680 | Infant food | 170986 | 3955 | Infant Formula, MEAD JOHNSON, ENFAMIL, ENFACARE, ready-to-feed, with ARA and DHA |  |
| 2680 | Infant food | 170991 | 3966 | Infant formula, NESTLE, GOOD START SOY, with DHA and ARA, liquid concentrate |  |
| 2680 | Infant food | 170993 | 3968 | Toddler formula, MEAD JOHNSON, ENFAGROW PREMIUM (formerly ENFAMIL, LIPIL, NEXT STEP), ready-to-feed |  |
| 2680 | Infant food | 170994 | 3986 | Infant Formula, MEAD JOHNSON, ENFAMIL, Newborn, with DHA and ARA, ready-to-feed |  |
| 2680 | Infant food | 170995 | 3987 | Infant formula, GERBER, GOOD START 2 Soy, with iron, ready-to-feed |  |
| 2680 | Infant food | 170996 | 3988 | Infant formula, GERBER, GOOD START, PROTECT PLUS, ready-to-feed |  |
| 2680 | Infant food | 170997 | 3989 | Infant Formula, GERBER GOOD START 2, GENTLE PLUS, ready-to-feed |  |
| 2680 | Infant food | 171371 | 3216 | Babyfood, GERBER, GRADUATES Lil Biscuits Vanilla Wheat |  |
| 2680 | Infant food | 171382 | 3952 | Infant formula, ABBOTT NUTRITION, SIMILAC, ISOMIL, ADVANCE with iron, liquid concentrate |  |
| 2680 | Infant food | 171383 | 3953 | Infant formula, ABBOTT NUTRITION, SIMILAC, ISOMIL, ADVANCE with iron, ready-to-feed |  |
| 2680 | Infant food | 171385 | 3960 | Infant formula, NESTLE, GOOD START SUPREME, with iron, DHA and ARA, ready-to-feed |  |
| 2680 | Infant food | 171386 | 3961 | Infant formula, NESTLE, GOOD START SUPREME, with iron, DHA and ARA, prepared from liquid concentrate |  |
| 2680 | Infant food | 171390 | 3982 | Infant formula, MEAD JOHNSON, ENFAMIL, Enfagrow, Soy, Toddler ready-to-feed |  |
| 2680 | Infant food | 171391 | 3983 | Infant formula, MEAD JOHNSON, ENFAMIL, NUTRAMIGEN AA, ready-to-feed |  |
| 2680 | Infant food | 171392 | 3984 | Infant formula, MEAD JOHNSON, ENFAMIL, Premature, with iron, 20 calories, ready-to-feed |  |
| 2680 | Infant food | 171393 | 3985 | Infant formula, MEAD JOHNSON, ENFAMIL, Premature, with iron, 24 calories, ready-to-feed |  |
| 2680 | Infant food | 171394 | 3990 | Infant formula, GERBER, GOOD START 2, PROTECT PLUS, ready-to-feed |  |
| 2680 | Infant food | 171395 | 3991 | Infant formula, ABBOTT NUTRITION, SIMILAC, GO AND GROW, ready-to-feed, with ARA and DHA |  |
| 2680 | Infant food | 171396 | 3992 | Infant formula, ABBOTT NUTRITION, SIMILAC, Expert Care, Diarrhea, ready- to- feed with ARA and DHA |  |
| 2680 | Infant food | 171397 | 3993 | Infant formula, ABBOTT NUTRITION, SIMILAC, For Spit Up, ready-to-feed, with ARA and DHA |  |
| 2680 | Infant food | 172289 | 3382 | Infant formula, MEAD JOHNSON, Enfamil Premature High Protein 24 Calories, ready to feed, with ARA and DHA |  |
| 2680 | Infant food | 172290 | 3384 | Infant formula, MEAD JOHNSON, Enfamil Premature 30 Calories, ready to feed, with ARA and DHA | |
| 2680 | Infant food | 172293 | 3391 | Infant formula, MEAD JOHNSON, Pregestimil 24 Calories, ready to feed, with ARA and DHA |  |
| 2680 | Infant food | 172295 | 3393 | Infant formula, MEAD JOHNSON, Enfamil for Supplementing, ready to feed, with ARA and DHA |  |
| 2680 | Infant food | 172303 | 3812 | Infant formula, MEAD JOHNSON, ENFAMIL, Infant, with iron, liquid concentrate, with ARA and DHA, reconstituted |  |
| 2680 | Infant food | 172304 | 3815 | Infant formula, MEAD JOHNSON, ENFAMIL LIPIL, with iron, ready-to-feed, with ARA and DHA |  |
| 2680 | Infant food | 172305 | 3825 | Infant formula, MEAD JOHNSON, ENFAMIL, LIPIL, low iron, ready to feed, with ARA and DHA |  |
| 2680 | Infant food | 172306 | 3832 | Infant formula, MEAD JOHNSON, ENFAMIL, Infant, ready-to-feed, with ARA and DHA |  |
| 2680 | Infant food | 172309 | 3845 | Infant formula, MEAD JOHNSON, ENFAMIL, NUTRAMIGEN, with iron, ready-to-feed, with ARA and DHA |  |
| 2680 | Infant food | 172310 | 3846 | Infant formula, ABBOTT NUTRITION, SIMILAC, ALIMENTUM, with iron, ready-to-feed |  |
| 2680 | Infant food | 172312 | 3851 | Infant formula, ABBOTT NUTRITION, SIMILAC, with iron, liquid concentrate, not reconstituted |  |
| 2680 | Infant food | 172313 | 3860 | Child formula, ABBOTT NUTRITION, PEDIASURE, ready-to-feed |  |
| 2680 | Infant food | 172315 | 3864 | Infant formula, MEAD JOHNSON, NEXT STEP, PROSOBEE, LIPIL, ready to feed, with ARA and DHA |  |
| 2680 | Infant food | 172318 | 3935 | Infant formula, ABBOTT NUTRITION, SIMILAC, ALIMENTUM, ADVANCE, ready-to-feed, with ARA and DHA |  |
| 2680 | Infant food | 172319 | 3936 | Infant formula, PBM PRODUCTS, store brand, ready-to-feed |  |
| 2680 | Infant food | 172320 | 3937 | Infant formula, PBM PRODUCTS, store brand, liquid concentrate, not reconstituted |  |
| 2680 | Infant food | 172321 | 3942 | Infant formula, MEAD JOHNSON, ENFAMIL, AR, ready-to-feed, with ARA and DHA |  |
| 2680 | Infant food | 172323 | 3944 | Infant formula, ABBOTT NUTRITION, SIMILAC NEOSURE, ready-to-feed, with ARA and DHA |  |
| 2680 | Infant food | 172325 | 3946 | Infant formula, ABBOTT NUTRITION, SIMILAC, SENSITIVE (LACTOSE FREE) ready-to-feed, with ARA and DHA |  |
| 2680 | Infant food | 172326 | 3951 | Infant formula, ABBOTT NUTRITION, SIMILAC, ADVANCE, with iron, liquid concentrate, not reconstituted |  |
| 2680 | Infant food | 173522 | 3376 | Infant formula, MEAD JOHNSON, Enfamil 24, ready to feed, with ARA and DHA |  |
| 2680 | Infant food | 173524 | 3387 | Infant formula, MEAD JOHNSON, Enfamil Reguline, ready to feed, with ARA and DHA |  |
| 2680 | Infant food | 173525 | 3388 | Infant formula, MEAD JOHNSON, Gentlease, ready to feed, with ARA and DHA |  |
| 2680 | Infant food | 173527 | 3390 | Infant formula, MEAD JOHNSON, Pregestimil 20 Calories, ready to feed, with ARA and DHA |  |
| 2680 | Infant food | 173535 | 3801 | Infant formula, NESTLE, GOOD START SUPREME, with iron, liquid concentrate, not reconstituted |  |
| 2680 | Infant food | 173537 | 3818 | Infant formula, MEAD JOHNSON, ENFAMIL, LIPIL, low iron, liquid concentrate, with ARA and DHA |  |
| 2680 | Infant food | 173540 | 3823 | Infant formula, MEAD JOHNSON, PROSOBEE, with iron, ready-to-feed |  |
| 2680 | Infant food | 173541 | 3841 | Infant formula, ABBOTT NUTRITION, SIMILAC, ISOMIL, with iron, ready-to-feed |  |
| 2680 | Infant food | 173542 | 3842 | Infant formula, ABBOTT NUTRITION, SIMILAC, ISOMIL, with iron, liquid concentrate |  |
| 2680 | Infant food | 173544 | 3844 | Infant formula, MEAD JOHNSON, ENFAMIL, NUTRAMIGEN, with iron, liquid concentrate not reconstituted, with ARA and DHA |  |
| 2680 | Infant food | 173547 | 3854 | Infant formula, MEAD JOHNSON, ENFAMIL, PROSOBEE, liquid concentrate, reconstituted, with ARA and DHA |  |
| 2680 | Infant food | 173548 | 3857 | Infant formula, MEAD JOHNSON, PROSOBEE, with iron, ready to feed, with ARA and DHA |  |
| 2680 | Infant food | 173549 | 3859 | Infant formula, NESTLE, GOOD START SOY, with DHA and ARA, ready-to-feed |  |
| 2680 | Infant food | 173550 | 3870 | Child formula, ABBOTT NUTRITION, PEDIASURE, ready-to-feed, with iron and fiber |  |
| 2680 | Infant food | 173555 | 3939 | Infant formula, PBM PRODUCTS, store brand, soy, ready-to-feed |  |
| 2680 | Infant food | 173556 | 3940 | Infant formula, PBM PRODUCTS, store brand, soy, liquid concentrate, not reconstituted |  |
| 2680 | Infant food | 173558 | 3947 | Infant formula, ABBOTT NUTRITION, SIMILAC, SENSITIVE, (LACTOSE FREE), liquid concentrate, with ARA and DHA |  |
| 2680 | Infant food | 173560 | 3949 | Infant formula, ABBOTT NUTRITION, SIMILAC, ADVANCE, with iron, ready-to-feed |  |
| 2731 | Bovine Meat | 169483 | 13795 | Beef, composite of trimmed retail cuts, separable lean and fat, trimmed to 1/8in fat, all grades, raw |  |
| 2731 | Bovine Meat | 173815 | 17088 | Veal, composite of trimmed retail cuts, separable lean and fat, raw |  |
| 2731 | Bovine Meat | 174757 | 23482 | Beef, composite of trimmed retail cuts, separable lean and fat, trimmed to 0in fat, all grades, raw |  |
| 2732 | Mutton & Goat Meat | 172479 | 17001 | Lamb, composite of trimmed retail cuts, separable lean and fat, trimmed to 1/4in fat, choice, raw |  |
| 2732 | Mutton & Goat Meat | 172545 | 17226 | Lamb, composite of trimmed retail cuts, separable lean and fat, trimmed to 1/8in fat, choice, raw |  |
| 2732 | Mutton & Goat Meat | 175303 | 17168 | Game meat, goat, raw |  |
| 2733 | Pigmeat | 167888 | 10187 | Pork, fresh, composite of trimmed retail cuts (leg, loin, shoulder, and spareribs), separable lean and fat, raw |  |
| 2734 | Poultry Meat | 171081 | 5165 | Turkey, whole, meat and skin, raw |  |
| 2734 | Poultry Meat | 171447 | 5006 | Chicken, broilers or fryers, meat and skin, raw |  |
| 2734 | Poultry Meat | 172408 | 5139 | Duck, domesticated, meat and skin, raw |  |
| 2734 | Poultry Meat | 172416 | 5151 | Guinea hen, meat and skin, raw |  |
| 2734 | Poultry Meat | 174470 | 5146 | Goose, domesticated, meat and skin, raw |  |
| 2735 | Meat, Other | 167607 | 35049 | Moose, meat, raw (Alaska Native) |  |
| 2735 | Meat, Other | 167610 | 35056 | Seal, bearded (Oogruk), meat, raw (Alaska Native) |  |
| 2735 | Meat, Other | 167622 | 35080 | Deer (venison), sitka, raw (Alaska Native) |  |
| 2735 | Meat, Other | 167745 | 93600 | Turtle, green, raw |  |
| 2735 | Meat, Other | 167888 | 10187 | Pork, fresh, composite of trimmed retail cuts (leg, loin, shoulder, and spareribs), separable lean and fat, raw |  |
| 2735 | Meat, Other | 168025 | 35071 | Seal, ringed, meat (Alaska Native) |  |
| 2735 | Meat, Other | 168029 | 35081 | Walrus, meat, raw (Alaska Native) |  |
| 2735 | Meat, Other | 168148 | 80200 | Frog legs, raw |  |
| 2735 | Meat, Other | 168987 | 35034 | Fish, devilfish, meat (Alaska Native) |  |
| 2735 | Meat, Other | 169003 | 35229 | Sea lion, Steller, meat (Alaska Native) |  |
| 2735 | Meat, Other | 169483 | 13795 | Beef, composite of trimmed retail cuts, separable lean and fat, trimmed to 1/8in fat, all grades, raw |  |
| 2735 | Meat, Other | 169793 | 35007 | Bear, black, meat (Alaska Native) |  |
| 2735 | Meat, Other | 169794 | 35008 | Bear, polar, meat, raw (Alaska Native) |  |
| 2735 | Meat, Other | 169797 | 35011 | Whale, beluga, meat, raw (Alaska Native) |  |
| 2735 | Meat, Other | 171081 | 5165 | Turkey, whole, meat and skin, raw |  |
| 2735 | Meat, Other | 171447 | 5006 | Chicken, broilers or fryers, meat and skin, raw |  |
| 2735 | Meat, Other | 172408 | 5139 | Duck, domesticated, meat and skin, raw |  |
| 2735 | Meat, Other | 172416 | 5151 | Guinea hen, meat and skin, raw |  |
| 2735 | Meat, Other | 172418 | 5157 | Quail, meat and skin, raw |  |
| 2735 | Meat, Other | 172479 | 17001 | Lamb, composite of trimmed retail cuts, separable lean and fat, trimmed to 1/4in fat, choice, raw |  |
| 2735 | Meat, Other | 172518 | 17174 | Game meat, muskrat, raw |  |
| 2735 | Meat, Other | 172521 | 17177 | Game meat, rabbit, domesticated, composite of cuts, raw |  |
| 2735 | Meat, Other | 172523 | 17183 | Game meat, squirrel, raw |  |
| 2735 | Meat, Other | 172545 | 17226 | Lamb, composite of trimmed retail cuts, separable lean and fat, trimmed to 1/8in fat, choice, raw |  |
| 2735 | Meat, Other | 172832 | 5621 | Emu, ground, raw |  |
| 2735 | Meat, Other | 173815 | 17088 | Veal, composite of trimmed retail cuts, separable lean and fat, raw |  |
| 2735 | Meat, Other | 173845 | 17146 | Game meat, bear, raw |  |
| 2735 | Meat, Other | 173851 | 17156 | Game meat, bison, separable lean only, raw |  |
| 2735 | Meat, Other | 173853 | 17162 | Game meat, caribou, raw |  |
| 2735 | Meat, Other | 173855 | 17164 | Game meat, deer, raw |  |
| 2735 | Meat, Other | 174344 | 17172 | Game meat, moose, raw |  |
| 2735 | Meat, Other | 174347 | 17180 | Game meat, rabbit, wild, raw |  |
| 2735 | Meat, Other | 174470 | 5146 | Goose, domesticated, meat and skin, raw |  |
| 2735 | Meat, Other | 174472 | 5153 | Pheasant, raw, meat and skin |  |
| 2735 | Meat, Other | 174475 | 5160 | Squab, (pigeon), meat and skin, raw |  |
| 2735 | Meat, Other | 174481 | 5641 | Ostrich, ground, raw |  |
| 2735 | Meat, Other | 174757 | 23482 | Beef, composite of trimmed retail cuts, separable lean and fat, trimmed to 0in fat, all grades, raw |  |
| 2735 | Meat, Other | 175086 | 17170 | Game meat, horse, raw |  |
| 2735 | Meat, Other | 175292 | 17144 | Game meat, antelope, raw |  |
| 2735 | Meat, Other | 175293 | 17149 | Bison, ground, grass-fed, raw |  |
| 2735 | Meat, Other | 175294 | 17150 | Game meat, beaver, raw |  |
| 2735 | Meat, Other | 175296 | 17152 | Game meat, beefalo, composite of cuts, raw |  |
| 2735 | Meat, Other | 175297 | 17158 | Game meat, boar, wild, raw |  |
| 2735 | Meat, Other | 175299 | 17160 | Game meat, buffalo, water, raw |  |
| 2735 | Meat, Other | 175301 | 17166 | Game meat, elk, raw |  |
| 2735 | Meat, Other | 175303 | 17168 | Game meat, goat, raw |  |
| 2736 | Offals, Edible | 167857 | 10100 | Pork, fresh, variety meats and by-products, ears, frozen, raw |  |
| 2736 | Offals, Edible | 167859 | 10102 | Pork, fresh, variety meats and by-products, feet, raw |  |
| 2736 | Offals, Edible | 167862 | 10110 | Pork, fresh, variety meats and by-products, liver, raw |  |
| 2736 | Offals, Edible | 167864 | 10112 | Pork, fresh, variety meats and by-products, lungs, raw |  |
| 2736 | Offals, Edible | 167865 | 10117 | Pork, fresh, variety meats and by-products, spleen, raw |  |
| 2736 | Offals, Edible | 167867 | 10119 | Pork, fresh, variety meats and by-products, stomach, raw |  |
| 2736 | Offals, Edible | 168026 | 35072 | Seal, ringed, liver (Alaska Native) |  |
| 2736 | Offals, Edible | 168031 | 35083 | Walrus, liver, raw (Alaska Native) |  |
| 2736 | Offals, Edible | 168264 | 10096 | Pork, fresh, variety meats and by-products, brain, raw |  |
| 2736 | Offals, Edible | 168266 | 10098 | Pork, fresh, variety meats and by-products, chitterlings, raw |  |
| 2736 | Offals, Edible | 168267 | 10103 | Pork, fresh, variety meats and by-products, heart, raw |  |
| 2736 | Offals, Edible | 168269 | 10105 | Pork, fresh, variety meats and by-products, jowl, raw |  |
| 2736 | Offals, Edible | 168270 | 10106 | Pork, fresh, variety meats and by-products, kidneys, raw |  |
| 2736 | Offals, Edible | 168273 | 10115 | Pork, fresh, variety meats and by-products, pancreas, raw |  |
| 2736 | Offals, Edible | 168275 | 10121 | Pork, fresh, variety meats and by-products, tongue, raw |  |
| 2736 | Offals, Edible | 168291 | 10174 | Pork, fresh, variety meats and by-products, tail, raw |  |
| 2736 | Offals, Edible | 168622 | 13318 | Beef, variety meats and by-products, brain, raw |  |
| 2736 | Offals, Edible | 168625 | 13321 | Beef, variety meats and by-products, heart, raw |  |
| 2736 | Offals, Edible | 168628 | 13328 | Beef, variety meats and by-products, lungs, raw |  |
| 2736 | Offals, Edible | 168980 | 35013 | Whale, beluga, liver, raw (Alaska Native) |  |
| 2736 | Offals, Edible | 168983 | 35023 | Caribou, liver, raw (Alaska Native) |  |
| 2736 | Offals, Edible | 168985 | 35025 | Caribou, tongue, raw (Alaska Native) |  |
| 2736 | Offals, Edible | 169000 | 35226 | Sea lion, Steller, liver (Alaska Native) |  |
| 2736 | Offals, Edible | 169002 | 35228 | Sea lion, Steller, heart (Alaska Native) |  |
| 2736 | Offals, Edible | 169449 | 13323 | Beef, variety meats and by-products, kidneys, raw |  |
| 2736 | Offals, Edible | 169451 | 13325 | Beef, variety meats and by-products, liver, raw |  |
| 2736 | Offals, Edible | 169452 | 13331 | Beef, variety meats and by-products, pancreas, raw |  |
| 2736 | Offals, Edible | 169454 | 13333 | Beef, variety meats and by-products, spleen, raw |  |
| 2736 | Offals, Edible | 170194 | 13337 | Beef, variety meats and by-products, thymus, raw |  |
| 2736 | Offals, Edible | 170196 | 13339 | Beef, variety meats and by-products, tongue, raw |  |
| 2736 | Offals, Edible | 170599 | 13341 | Beef, variety meats and by-products, tripe, raw |  |
| 2736 | Offals, Edible | 171060 | 5027 | Chicken, liver, all classes, raw |  |
| 2736 | Offals, Edible | 171458 | 5025 | Chicken, heart, all classes, raw |  |
| 2736 | Offals, Edible | 171484 | 5175 | Turkey, all classes, heart, raw |  |
| 2736 | Offals, Edible | 171486 | 5177 | Turkey, all classes, liver, raw |  |
| 2736 | Offals, Edible | 172415 | 5150 | Goose, liver, raw |  |
| 2736 | Offals, Edible | 172525 | 17185 | Lamb, variety meats and by-products, brain, raw |  |
| 2736 | Offals, Edible | 172527 | 17191 | Lamb, variety meats and by-products, heart, raw |  |
| 2736 | Offals, Edible | 172529 | 17193 | Veal, variety meats and by-products, heart, raw |  |
| 2736 | Offals, Edible | 172531 | 17199 | Lamb, variety meats and by-products, liver, raw |  |
| 2736 | Offals, Edible | 172534 | 17202 | Veal, variety meats and by-products, liver, raw |  |
| 2736 | Offals, Edible | 172538 | 17210 | Lamb, variety meats and by-products, pancreas, raw |  |
| 2736 | Offals, Edible | 172540 | 17216 | Veal, variety meats and by-products, spleen, raw |  |
| 2736 | Offals, Edible | 172542 | 17218 | Veal, variety meats and by-products, thymus, raw |  |
| 2736 | Offals, Edible | 172615 | 17369 | Lamb, New Zealand, imported, liver, raw |  |
| 2736 | Offals, Edible | 172621 | 17379 | Lamb, New Zealand, imported, tongue - swiss cut, raw |  |
| 2736 | Offals, Edible | 173096 | 23445 | Beef, New Zealand, imported, variety meats and by-products, tripe uncooked, raw |  |
| 2736 | Offals, Edible | 174351 | 17188 | Veal, variety meats and by-products, brain, raw |  |
| 2736 | Offals, Edible | 174354 | 17195 | Lamb, variety meats and by-products, kidneys, raw |  |
| 2736 | Offals, Edible | 174356 | 17197 | Veal, variety meats and by-products, kidneys, raw |  |
| 2736 | Offals, Edible | 174359 | 17205 | Lamb, variety meats and by-products, lungs, raw |  |
| 2736 | Offals, Edible | 174361 | 17207 | Veal, variety meats and by-products, lungs, raw |  |
| 2736 | Offals, Edible | 174362 | 17212 | Veal, variety meats and by-products, pancreas, raw |  |
| 2736 | Offals, Edible | 174364 | 17214 | Lamb, variety meats and by-products, spleen, raw |  |
| 2736 | Offals, Edible | 174366 | 17220 | Lamb, variety meats and by-products, tongue, raw |  |
| 2736 | Offals, Edible | 174368 | 17222 | Veal, variety meats and by-products, tongue, raw |  |
| 2736 | Offals, Edible | 174437 | 17358 | Lamb, New Zealand, imported, brains, raw |  |
| 2736 | Offals, Edible | 174442 | 17367 | Lamb, New Zealand, imported, kidney, raw |  |
| 2736 | Offals, Edible | 174444 | 17373 | Lamb, New Zealand, imported, heart, raw |  |
| 2736 | Offals, Edible | 174467 | 5143 | Duck, domesticated, liver, raw |  |
| 2736 | Offals, Edible | 174723 | 23415 | Beef, New Zealand, imported, variety meats and by-products, heart, raw |  |
| 2736 | Offals, Edible | 174727 | 23423 | Beef, New Zealand, imported, variety meats and by-products, kidney, raw |  |
| 2736 | Offals, Edible | 174729 | 23425 | Beef, New Zealand, imported, variety meats and by-products, liver, raw |  |
| 2736 | Offals, Edible | 174738 | 23443 | Beef, New Zealand, imported, variety meats and by-products, tongue, raw |  |
| 2737 | Fats, Animals, Raw | 167861 | 10109 | Pork, fresh, variety meats and by-products, leaf fat, raw |  |
| 2737 | Fats, Animals, Raw | 170193 | 13335 | Beef, variety meats and by-products, suet, raw |  |
| 2737 | Fats, Animals, Raw | 171400 | 4001 | Fat, beef tallow |  |
| 2737 | Fats, Animals, Raw | 171401 | 4002 | Lard |  |
| 2737 | Fats, Animals, Raw | 173564 | 4542 | Fat, chicken |  |
| 2737 | Fats, Animals, Raw | 173571 | 4575 | Fat, turkey |  |
| 2737 | Fats, Animals, Raw | 173572 | 4576 | Fat, goose |  |
| 2740 | Butter, Ghee | 173430 | 1145 | Butter, without salt |  |
| 2743 | Cream | 170859 | 1053 | Cream, fluid, heavy whipping |  |
| 2744 | Eggs | 171287 | 1123 | Egg, whole, raw, fresh |  |
| 2744 | Eggs | 172189 | 1138 | Egg, duck, whole, fresh, raw |  |
| 2744 | Eggs | 172190 | 1139 | Egg, goose, whole, fresh, raw |  |
| 2744 | Eggs | 172191 | 1140 | Egg, quail, whole, fresh, raw |  |
| 2744 | Eggs | 172192 | 1141 | Egg, turkey, whole, fresh, raw |  |
| 2745 | Honey | 169640 | 19296 | Honey |  |
| 2761 | Freshwater Fish | 167639 | 35150 | Fish, salmon, coho (silver), raw (Alaska Native) |  |
| 2761 | Freshwater Fish | 167648 | 35169 | Fish, sheefish, raw (Alaska Native) |  |
| 2761 | Freshwater Fish | 168033 | 35089 | Fish, whitefish, mixed species, raw (Alaska Native) |  |
| 2761 | Freshwater Fish | 168045 | 35151 | Fish, salmon, sockeye (red), raw (Alaska Native) |  |
| 2761 | Freshwater Fish | 168046 | 35152 | Fish, Salmon, Chum, raw (Alaska Native) |  |
| 2761 | Freshwater Fish | 168047 | 35153 | Fish, salmon, king (chinook), raw (Alaska Native) |  |
| 2761 | Freshwater Fish | 171948 | 15004 | Fish, bass, striped, raw |  |
| 2761 | Freshwater Fish | 171950 | 15006 | Fish, burbot, raw |  |
| 2761 | Freshwater Fish | 171952 | 15008 | Fish, carp, raw |  |
| 2761 | Freshwater Fish | 171953 | 15013 | Fish, cisco, raw |  |
| 2761 | Freshwater Fish | 171960 | 15024 | Fish, drum, freshwater, raw |  |
| 2761 | Freshwater Fish | 173675 | 15053 | Fish, milkfish, raw |  |
| 2761 | Freshwater Fish | 173678 | 15060 | Fish, perch, mixed species, raw |  |
| 2761 | Freshwater Fish | 173680 | 15062 | Fish, pike, northern, raw |  |
| 2761 | Freshwater Fish | 173686 | 15076 | Fish, salmon, Atlantic, wild, raw |  |
| 2761 | Freshwater Fish | 173688 | 15078 | Fish, salmon, chinook, raw |  |
| 2761 | Freshwater Fish | 173691 | 15085 | Fish, salmon, sockeye, raw |  |
| 2761 | Freshwater Fish | 173695 | 15093 | Fish, seatrout, mixed species, raw |  |
| 2761 | Freshwater Fish | 173696 | 15094 | Fish, shad, american, raw |  |
| 2761 | Freshwater Fish | 173701 | 15104 | Fish, sturgeon, mixed species, raw |  |
| 2761 | Freshwater Fish | 173711 | 15130 | Fish, whitefish, mixed species, raw |  |
| 2761 | Freshwater Fish | 173715 | 15238 | Fish, salmon, coho, farmed, raw |  |
| 2761 | Freshwater Fish | 173717 | 15240 | Fish, trout, rainbow, farmed, raw |  |
| 2761 | Freshwater Fish | 174184 | 15003 | Fish, bass, fresh water, mixed species, raw |  |
| 2761 | Freshwater Fish | 174186 | 15010 | Fish, catfish, channel, wild, raw |  |
| 2761 | Freshwater Fish | 174193 | 15025 | Fish, eel, mixed species, raw |  |
| 2761 | Freshwater Fish | 175128 | 15064 | Fish, pike, walleye, raw |  |
| 2761 | Freshwater Fish | 175136 | 15081 | Fish, salmon, coho, wild, raw |  |
| 2761 | Freshwater Fish | 175138 | 15083 | Fish, salmon, pink, raw |  |
| 2761 | Freshwater Fish | 175146 | 15099 | Fish, smelt, rainbow, raw |  |
| 2761 | Freshwater Fish | 175150 | 15107 | Fish, sucker, white, raw |  |
| 2761 | Freshwater Fish | 175153 | 15114 | Fish, trout, mixed species, raw |  |
| 2761 | Freshwater Fish | 175154 | 15115 | Fish, trout, rainbow, wild, raw |  |
| 2761 | Freshwater Fish | 175165 | 15234 | Fish, catfish, channel, farmed, raw |  |
| 2761 | Freshwater Fish | 175167 | 15236 | Fish, salmon, Atlantic, farmed, raw |  |
| 2761 | Freshwater Fish | 175176 | 15261 | Fish, tilapia, raw |  |
| 2761 | Freshwater Fish | 175181 | 15274 | Fish, trout, brook, raw, New York State |  |
| 2762 | Demersal Fish | 167638 | 35149 | Fish, halibut, raw, with skin (Alaska Native) |  |
| 2762 | Demersal Fish | 169809 | 35046 | Fish, lingcod, meat, raw (Alaska Native) |  |
| 2762 | Demersal Fish | 171955 | 15015 | Fish, cod, Atlantic, raw |  |
| 2762 | Demersal Fish | 171958 | 15022 | Fish, cusk, raw |  |
| 2762 | Demersal Fish | 171962 | 15031 | Fish, grouper, mixed species, raw |  |
| 2762 | Demersal Fish | 171964 | 15033 | Fish, haddock, raw |  |
| 2762 | Demersal Fish | 171965 | 15038 | Fish, halibut, Greenland, raw |  |
| 2762 | Demersal Fish | 173670 | 15044 | Fish, ling, raw |  |
| 2762 | Demersal Fish | 173671 | 15045 | Fish, lingcod, raw |  |
| 2762 | Demersal Fish | 173676 | 15054 | Fish, monkfish, raw |  |
| 2762 | Demersal Fish | 173677 | 15059 | Fish, pout, ocean, raw |  |
| 2762 | Demersal Fish | 173684 | 15070 | Fish, rockfish, Pacific, mixed species, raw |  |
| 2762 | Demersal Fish | 173697 | 15095 | Fish, shark, mixed species, raw |  |
| 2762 | Demersal Fish | 173698 | 15101 | Fish, snapper, mixed species, raw |  |
| 2762 | Demersal Fish | 173700 | 15103 | Fish, spot, raw |  |
| 2762 | Demersal Fish | 173705 | 15112 | Fish, tilefish, raw |  |
| 2762 | Demersal Fish | 173710 | 15129 | Fish, turbot, european, raw |  |
| 2762 | Demersal Fish | 173713 | 15132 | Fish, whiting, mixed species, raw |  |
| 2762 | Demersal Fish | 173725 | 15266 | Fish, pollock, Alaska, raw |  |
| 2762 | Demersal Fish | 174191 | 15019 | Fish, cod, Pacific, raw (may have been previously frozen) |  |
| 2762 | Demersal Fish | 174192 | 15020 | Fish, croaker, Atlantic, raw |  |
| 2762 | Demersal Fish | 174196 | 15028 | Fish, flatfish (flounder and sole species), raw |  |
| 2762 | Demersal Fish | 174200 | 15036 | Fish, halibut, Atlantic and Pacific, raw |  |
| 2762 | Demersal Fish | 175123 | 15055 | Fish, mullet, striped, raw |  |
| 2762 | Demersal Fish | 175125 | 15057 | Fish, ocean perch, Atlantic, raw |  |
| 2762 | Demersal Fish | 175129 | 15065 | Fish, pollock, Atlantic, raw |  |
| 2762 | Demersal Fish | 175130 | 15066 | Fish, pollock, Alaska, raw (may contain additives to retain moisture) |  |
| 2762 | Demersal Fish | 175133 | 15073 | Fish, roughy, orange, raw |  |
| 2762 | Demersal Fish | 175134 | 15074 | Fish, sablefish, raw |  |
| 2762 | Demersal Fish | 175141 | 15090 | Fish, scup, raw |  |
| 2762 | Demersal Fish | 175142 | 15091 | Fish, sea bass, mixed species, raw |  |
| 2762 | Demersal Fish | 175144 | 15097 | Fish, sheepshead, raw |  |
| 2762 | Demersal Fish | 175162 | 15134 | Fish, wolffish, Atlantic, raw |  |
| 2762 | Demersal Fish | 175163 | 15135 | Fish, yellowtail, mixed species, raw |  |
| 2762 | Demersal Fish | 333476 | 15066 | Fish, pollock, raw |  |
| 2763 | Pelagic Fish | 171949 | 15005 | Fish, bluefish, raw |  |
| 2763 | Pelagic Fish | 171951 | 15007 | Fish, butterfish, raw |  |
| 2763 | Pelagic Fish | 171959 | 15023 | Fish, mahimahi, raw |  |
| 2763 | Pelagic Fish | 173669 | 15043 | Fish, herring, Pacific, raw |  |
| 2763 | Pelagic Fish | 173672 | 15050 | Fish, mackerel, Pacific and jack, mixed species, raw |  |
| 2763 | Pelagic Fish | 173673 | 15051 | Fish, mackerel, spanish, raw |  |
| 2763 | Pelagic Fish | 173682 | 15068 | Fish, pompano, florida, raw |  |
| 2763 | Pelagic Fish | 173703 | 15110 | Fish, swordfish, raw |  |
| 2763 | Pelagic Fish | 173706 | 15117 | Fish, tuna, fresh, bluefin, raw |  |
| 2763 | Pelagic Fish | 174182 | 15001 | Fish, anchovy, european, raw |  |
| 2763 | Pelagic Fish | 175116 | 15039 | Fish, herring, Atlantic, raw |  |
| 2763 | Pelagic Fish | 175119 | 15046 | Fish, mackerel, Atlantic, raw |  |
| 2763 | Pelagic Fish | 175122 | 15049 | Fish, mackerel, king, raw |  |
| 2763 | Pelagic Fish | 175151 | 15108 | Fish, sunfish, pumpkin seed, raw |  |
| 2763 | Pelagic Fish | 175156 | 15123 | Fish, tuna, fresh, skipjack, raw |  |
| 2763 | Pelagic Fish | 175159 | 15127 | Fish, tuna, fresh, yellowfin, raw |  |
| 2764 | Marine Fish, Other | 167638 | 35149 | Fish, halibut, raw, with skin (Alaska Native) |  |
| 2764 | Marine Fish, Other | 167639 | 35150 | Fish, salmon, coho (silver), raw (Alaska Native) |  |
| 2764 | Marine Fish, Other | 167648 | 35169 | Fish, sheefish, raw (Alaska Native) |  |
| 2764 | Marine Fish, Other | 168033 | 35089 | Fish, whitefish, mixed species, raw (Alaska Native) |  |
| 2764 | Marine Fish, Other | 168045 | 35151 | Fish, salmon, sockeye (red), raw (Alaska Native) |  |
| 2764 | Marine Fish, Other | 168046 | 35152 | Fish, Salmon, Chum, raw (Alaska Native) |  |
| 2764 | Marine Fish, Other | 168047 | 35153 | Fish, salmon, king (chinook), raw (Alaska Native) |  |
| 2764 | Marine Fish, Other | 169809 | 35046 | Fish, lingcod, meat, raw (Alaska Native) |  |
| 2764 | Marine Fish, Other | 171948 | 15004 | Fish, bass, striped, raw |  |
| 2764 | Marine Fish, Other | 171949 | 15005 | Fish, bluefish, raw |  |
| 2764 | Marine Fish, Other | 171950 | 15006 | Fish, burbot, raw |  |
| 2764 | Marine Fish, Other | 171951 | 15007 | Fish, butterfish, raw |  |
| 2764 | Marine Fish, Other | 171952 | 15008 | Fish, carp, raw |  |
| 2764 | Marine Fish, Other | 171953 | 15013 | Fish, cisco, raw |  |
| 2764 | Marine Fish, Other | 171955 | 15015 | Fish, cod, Atlantic, raw |  |
| 2764 | Marine Fish, Other | 171958 | 15022 | Fish, cusk, raw |  |
| 2764 | Marine Fish, Other | 171959 | 15023 | Fish, mahimahi, raw |  |
| 2764 | Marine Fish, Other | 171960 | 15024 | Fish, drum, freshwater, raw |  |
| 2764 | Marine Fish, Other | 171962 | 15031 | Fish, grouper, mixed species, raw |  |
| 2764 | Marine Fish, Other | 171964 | 15033 | Fish, haddock, raw |  |
| 2764 | Marine Fish, Other | 171965 | 15038 | Fish, halibut, Greenland, raw |  |
| 2764 | Marine Fish, Other | 173669 | 15043 | Fish, herring, Pacific, raw |  |
| 2764 | Marine Fish, Other | 173670 | 15044 | Fish, ling, raw |  |
| 2764 | Marine Fish, Other | 173671 | 15045 | Fish, lingcod, raw |  |
| 2764 | Marine Fish, Other | 173672 | 15050 | Fish, mackerel, Pacific and jack, mixed species, raw |  |
| 2764 | Marine Fish, Other | 173673 | 15051 | Fish, mackerel, spanish, raw |  |
| 2764 | Marine Fish, Other | 173675 | 15053 | Fish, milkfish, raw |  |
| 2764 | Marine Fish, Other | 173676 | 15054 | Fish, monkfish, raw |  |
| 2764 | Marine Fish, Other | 173677 | 15059 | Fish, pout, ocean, raw |  |
| 2764 | Marine Fish, Other | 173678 | 15060 | Fish, perch, mixed species, raw |  |
| 2764 | Marine Fish, Other | 173680 | 15062 | Fish, pike, northern, raw |  |
| 2764 | Marine Fish, Other | 173682 | 15068 | Fish, pompano, florida, raw |  |
| 2764 | Marine Fish, Other | 173684 | 15070 | Fish, rockfish, Pacific, mixed species, raw |  |
| 2764 | Marine Fish, Other | 173686 | 15076 | Fish, salmon, Atlantic, wild, raw |  |
| 2764 | Marine Fish, Other | 173688 | 15078 | Fish, salmon, chinook, raw |  |
| 2764 | Marine Fish, Other | 173691 | 15085 | Fish, salmon, sockeye, raw |  |
| 2764 | Marine Fish, Other | 173695 | 15093 | Fish, seatrout, mixed species, raw |  |
| 2764 | Marine Fish, Other | 173696 | 15094 | Fish, shad, american, raw |  |
| 2764 | Marine Fish, Other | 173697 | 15095 | Fish, shark, mixed species, raw |  |
| 2764 | Marine Fish, Other | 173698 | 15101 | Fish, snapper, mixed species, raw |  |
| 2764 | Marine Fish, Other | 173700 | 15103 | Fish, spot, raw |  |
| 2764 | Marine Fish, Other | 173701 | 15104 | Fish, sturgeon, mixed species, raw |  |
| 2764 | Marine Fish, Other | 173703 | 15110 | Fish, swordfish, raw |  |
| 2764 | Marine Fish, Other | 173705 | 15112 | Fish, tilefish, raw |  |
| 2764 | Marine Fish, Other | 173706 | 15117 | Fish, tuna, fresh, bluefin, raw |  |
| 2764 | Marine Fish, Other | 173710 | 15129 | Fish, turbot, european, raw |  |
| 2764 | Marine Fish, Other | 173711 | 15130 | Fish, whitefish, mixed species, raw |  |
| 2764 | Marine Fish, Other | 173713 | 15132 | Fish, whiting, mixed species, raw |  |
| 2764 | Marine Fish, Other | 173715 | 15238 | Fish, salmon, coho, farmed, raw |  |
| 2764 | Marine Fish, Other | 173717 | 15240 | Fish, trout, rainbow, farmed, raw |  |
| 2764 | Marine Fish, Other | 173725 | 15266 | Fish, pollock, Alaska, raw |  |
| 2764 | Marine Fish, Other | 174182 | 15001 | Fish, anchovy, european, raw |  |
| 2764 | Marine Fish, Other | 174184 | 15003 | Fish, bass, fresh water, mixed species, raw |  |
| 2764 | Marine Fish, Other | 174186 | 15010 | Fish, catfish, channel, wild, raw |  |
| 2764 | Marine Fish, Other | 174191 | 15019 | Fish, cod, Pacific, raw (may have been previously frozen) |  |
| 2764 | Marine Fish, Other | 174192 | 15020 | Fish, croaker, Atlantic, raw |  |
| 2764 | Marine Fish, Other | 174193 | 15025 | Fish, eel, mixed species, raw |  |
| 2764 | Marine Fish, Other | 174196 | 15028 | Fish, flatfish (flounder and sole species), raw |  |
| 2764 | Marine Fish, Other | 174200 | 15036 | Fish, halibut, Atlantic and Pacific, raw |  |
| 2764 | Marine Fish, Other | 175116 | 15039 | Fish, herring, Atlantic, raw |  |
| 2764 | Marine Fish, Other | 175119 | 15046 | Fish, mackerel, Atlantic, raw |  |
| 2764 | Marine Fish, Other | 175122 | 15049 | Fish, mackerel, king, raw |  |
| 2764 | Marine Fish, Other | 175123 | 15055 | Fish, mullet, striped, raw |  |
| 2764 | Marine Fish, Other | 175125 | 15057 | Fish, ocean perch, Atlantic, raw |  |
| 2764 | Marine Fish, Other | 175128 | 15064 | Fish, pike, walleye, raw |  |
| 2764 | Marine Fish, Other | 175129 | 15065 | Fish, pollock, Atlantic, raw |  |
| 2764 | Marine Fish, Other | 175130 | 15066 | Fish, pollock, Alaska, raw (may contain additives to retain moisture) |  |
| 2764 | Marine Fish, Other | 175133 | 15073 | Fish, roughy, orange, raw |  |
| 2764 | Marine Fish, Other | 175134 | 15074 | Fish, sablefish, raw |  |
| 2764 | Marine Fish, Other | 175136 | 15081 | Fish, salmon, coho, wild, raw |  |
| 2764 | Marine Fish, Other | 175138 | 15083 | Fish, salmon, pink, raw |  |
| 2764 | Marine Fish, Other | 175141 | 15090 | Fish, scup, raw |  |
| 2764 | Marine Fish, Other | 175142 | 15091 | Fish, sea bass, mixed species, raw |  |
| 2764 | Marine Fish, Other | 175144 | 15097 | Fish, sheepshead, raw |  |
| 2764 | Marine Fish, Other | 175146 | 15099 | Fish, smelt, rainbow, raw |  |
| 2764 | Marine Fish, Other | 175150 | 15107 | Fish, sucker, white, raw |  |
| 2764 | Marine Fish, Other | 175151 | 15108 | Fish, sunfish, pumpkin seed, raw |  |
| 2764 | Marine Fish, Other | 175153 | 15114 | Fish, trout, mixed species, raw |  |
| 2764 | Marine Fish, Other | 175154 | 15115 | Fish, trout, rainbow, wild, raw |  |
| 2764 | Marine Fish, Other | 175156 | 15123 | Fish, tuna, fresh, skipjack, raw |  |
| 2764 | Marine Fish, Other | 175159 | 15127 | Fish, tuna, fresh, yellowfin, raw |  |
| 2764 | Marine Fish, Other | 175162 | 15134 | Fish, wolffish, Atlantic, raw |  |
| 2764 | Marine Fish, Other | 175163 | 15135 | Fish, yellowtail, mixed species, raw |  |
| 2764 | Marine Fish, Other | 175165 | 15234 | Fish, catfish, channel, farmed, raw |  |
| 2764 | Marine Fish, Other | 175167 | 15236 | Fish, salmon, Atlantic, farmed, raw |  |
| 2764 | Marine Fish, Other | 175176 | 15261 | Fish, tilapia, raw |  |
| 2764 | Marine Fish, Other | 175181 | 15274 | Fish, trout, brook, raw, New York State |  |
| 2764 | Marine Fish, Other | 333476 | 15066 | Fish, pollock, raw |  |
| 2765 | Crustaceans | 171968 | 15143 | Crustaceans, crab, dungeness, raw |  |
| 2765 | Crustaceans | 171969 | 15144 | Crustaceans, crab, queen, raw |  |
| 2765 | Crustaceans | 174204 | 15139 | Crustaceans, crab, blue, raw |  |
| 2765 | Crustaceans | 174206 | 15145 | Crustaceans, crayfish, mixed species, wild, raw |  |
| 2765 | Crustaceans | 174208 | 15147 | Crustaceans, lobster, northern, raw |  |
| 2765 | Crustaceans | 174211 | 15154 | Crustaceans, spiny lobster, mixed species, raw |  |
| 2765 | Crustaceans | 175164 | 15136 | Crustaceans, crab, alaska king, raw |  |
| 2765 | Crustaceans | 175169 | 15242 | Crustaceans, crayfish, mixed species, farmed, raw |  |
| 2765 | Crustaceans | 175179 | 15270 | Crustaceans, shrimp, raw |  |
| 2766 | Cephalopods | 168019 | 35054 | Octopus (Alaska Native) |  |
| 2766 | Cephalopods | 174215 | 15163 | Mollusks, cuttlefish, mixed species, raw |  |
| 2766 | Cephalopods | 174218 | 15166 | Mollusks, octopus, common, raw |  |
| 2766 | Cephalopods | 174223 | 15175 | Mollusks, squid, mixed species, raw |  |
| 2767 | Molluscs, Other | 167744 | 90560 | Mollusks, snail, raw |  |
| 2767 | Molluscs, Other | 169803 | 35028 | Cockles, raw (Alaska Native) |  |
| 2767 | Molluscs, Other | 171978 | 15167 | Mollusks, oyster, eastern, wild, raw |  |
| 2767 | Molluscs, Other | 171983 | 15177 | Mollusks, whelk, unspecified, raw |  |
| 2767 | Molluscs, Other | 174212 | 15155 | Mollusks, abalone, mixed species, raw |  |
| 2767 | Molluscs, Other | 174214 | 15157 | Mollusks, clam, mixed species, raw |  |
| 2767 | Molluscs, Other | 174216 | 15164 | Mollusks, mussel, blue, raw |  |
| 2767 | Molluscs, Other | 174219 | 15171 | Mollusks, oyster, Pacific, raw |  |
| 2767 | Molluscs, Other | 174220 | 15172 | Mollusks, scallop, mixed species, raw |  |
| 2767 | Molluscs, Other | 175172 | 15245 | Mollusks, oyster, eastern, farmed, raw |  |
| 2768 | Meat, Aquatic Mammals | 167610 | 35056 | Seal, bearded (Oogruk), meat, raw (Alaska Native) |  |
| 2768 | Meat, Aquatic Mammals | 168025 | 35071 | Seal, ringed, meat (Alaska Native) |  |
| 2768 | Meat, Aquatic Mammals | 168029 | 35081 | Walrus, meat, raw (Alaska Native) |  |
| 2768 | Meat, Aquatic Mammals | 168987 | 35034 | Fish, devilfish, meat (Alaska Native) |  |
| 2768 | Meat, Aquatic Mammals | 169003 | 35229 | Sea lion, Steller, meat (Alaska Native) |  |
| 2768 | Meat, Aquatic Mammals | 169797 | 35011 | Whale, beluga, meat, raw (Alaska Native) |  |
| 2769 | Aquatic Animals, Others | 167612 | 35058 | Oopah (tunicate), whole animal (Alaska Native) |  |
| 2769 | Aquatic Animals, Others | 167618 | 35070 | Sea cucumber, yane (Alaska Native) |  |
| 2769 | Aquatic Animals, Others | 167745 | 93600 | Turtle, green, raw |  |
| 2769 | Aquatic Animals, Others | 168148 | 80200 | Frog legs, raw |  |
| 2769 | Aquatic Animals, Others | 168978 | 35004 | Ascidians (tunughnak) (Alaska Native) |  |
| 2775 | Aquatic Plants | 168456 | 11444 | Seaweed, irishmoss, raw |  |
| 2775 | Aquatic Plants | 168457 | 11445 | Seaweed, kelp, raw |  |
| 2775 | Aquatic Plants | 169250 | 11254 | Lotus root, raw |  |
| 2775 | Aquatic Plants | 169280 | 11442 | Seaweed, agar, raw |  |
| 2775 | Aquatic Plants | 169301 | 11503 | Water convolvulus,raw |  |
| 2775 | Aquatic Plants | 170091 | 11666 | Seaweed, spirulina, raw |  |
| 2775 | Aquatic Plants | 170496 | 11669 | Seaweed, wakame, raw |  |
| 2781 | Fish, Body Oil | 172340 | 4590 | Fish oil, herring |  |
| 2781 | Fish, Body Oil | 172341 | 4591 | Fish oil, menhaden |  |
| 2781 | Fish, Body Oil | 172343 | 4593 | Fish oil, salmon |  |
| 2781 | Fish, Liver Oil | 173577 | 4589 | Fish oil, cod liver |  |
| 2781 | Fish, Body Oil | 173578 | 4594 | Fish oil, sardine |  |
| 2805 | Rice (Milled Equivalent) | 168876 | 20042 | Rice, brown, parboiled, dry, Uncle Ben's |  |
| 2805 | Rice (Milled Equivalent) | 168883 | 20054 | Rice, white, glutinous, unenriched, uncooked |  |
| 2805 | Rice (Milled Equivalent) | 168931 | 20452 | Rice, white, short-grain, raw, unenriched |  |
| 2805 | Rice (Milled Equivalent) | 169703 | 20036 | Rice, brown, long-grain, raw (Includes foods for USDA's Food Distribution Program) |  |
| 2805 | Rice (Milled Equivalent) | 169706 | 20040 | Rice, brown, medium-grain, raw (Includes foods for USDA's Food Distribution Program) |  |
| 2805 | Rice (Milled Equivalent) | 169756 | 20444 | Rice, white, long-grain, regular, raw, unenriched |  |
| 2805 | Rice (Milled Equivalent) | 169758 | 20446 | Rice, white, long-grain, parboiled, unenriched, dry |  |
| 2805 | Rice (Milled Equivalent) | 169760 | 20450 | Rice, white, medium-grain, raw, unenriched |  |
| 2848 | Milk - Excluding Butter | 170882 | 1109 | Milk, sheep, fluid |  |
| 2848 | Milk - Excluding Butter | 171280 | 1108 | Milk, indian buffalo, fluid |  |
| 2848 | Milk - Excluding Butter | 172217 | 1211 | Milk, whole, 3.25% milkfat, without added vitamin A and vitamin D |  |
| **Flour** |  |  |  |  |  |
| *Whole* |  |  |  |  |  |
| 2511 | Wheat and products | 168944 | 20649 | Wheat flour, whole-grain, soft wheat |  |
| *Refined* |  |  |  |  |  |
| 2511 | Wheat and products | 168913 | 20129 | Wheat flours, bread, unenriched |  |
| 2511 | Wheat and products | 168940 | 20635 | Wheat flour, white (industrial), 11.5% protein, bleached, unenriched |  |
| 2511 | Wheat and products | 169761 | 20481 | Wheat flour, white, all-purpose, unenriched |  |
| 2511 | Wheat and products | 172017 | 20624 | Wheat flour, white (industrial), 9% protein, bleached, unenriched |  |
| 2511 | Wheat and products | 172019 | 20629 | Wheat flour, white (industrial), 10% protein, bleached, unenriched |  |
| 2511 | Wheat and products | 172020 | 20641 | Wheat flour, white (industrial), 13% protein, bleached, unenriched |  |
| 2511 | Wheat and products | 172022 | 20646 | Wheat flour, white (industrial), 15% protein, bleached, unenriched |  |
| *Whole/refined* |  |  |  |  |  |
| 2514 | Maize and products | 168929 | 20422 | Cornmeal, degermed, unenriched, yellow |  |
| 2514 | Maize and products | 172015 | 20522 | Cornmeal, degermed, unenriched, white |  |
| *Masa* |  |  |  |  |  |
| 2514 | Maize and products | 169696 | 20019 | Corn flour, masa, unenriched, white |  |
| *Whole/refined* |  |  |  |  |  |
| 2517 | Millet and products | 169702 | 20031 | Millet, raw |  |
| 2517 | Millet and products | 169702 | 20031 | millet, refined |  |
| *Whole/refined* |  |  |  |  |  |
| 2518 | Sorghum and products | 168943 | 20648 | Sorghum flour, whole-grain |  |
| 2518 | Sorghum and products | 173262 | 20650 | Sorghum flour, refined, unenriched |  |

**Supplementary Table 3: NBS food composition matches**

Food composition data come from the Nutrient Database for Standard Reference Legacy database (NDB) available at Food Data Central (FDC) (https://fdc.nal.usda.gov), supplemented by the West Africa Food Composition Table, 2019 (https://www.fao.org/infoods/infoods/tables-and-databases/faoinfoods-databases/en) shown in yellow highlight. Matches were based on definitions for FAO Food Balance Sheet (FBS) categories, using food items in primary commodity form or processed and cooked as specified in the FDC item name.
