# Supplementary material for: Tracing global flows of bioactive compounds from farm to fork in Nutrient Balance Sheets can help guide intervention towards healthier food supplies
Source: Nat Food. Author manuscript; Available in PMC 2022 Oct 11. (PMC7613697; doi:10.1038/s43016-022-00585-w)
Supplement: Supplementary Table 4 [file EMS153904-supplement-Supplementary_Table_4.docx]

| **FBS_itemcode** | | **FBS_itemname** | | | **PhyFoodComp1.0 food_item_id** | | | **food_name_english** | | |  |  |
| --- | --- | --- | --- | --- | --- | --- | --- | --- | --- | --- | --- | --- |
| 2511 | Wheat and products | | | 1030220 | | | Wheat, Bobwhite, MON 71800 (glyphosate tolerant), raw | | | | | |
| 2511 | Wheat and products | | | 1030221 | | | Wheat, Bobwhite, MON 71800 (glyphosate tolerant), raw | | | | | |
| 2511 | Wheat and products | | | 1030222 | | | Wheat, Bobwhite, raw | | | | | |
| 2511 | Wheat and products | | | 1030223 | | | Wheat, Bobwhite, raw | | | | | |
| 2511 | Wheat and products | | | 1030224 | | | Wheat, C-306, grown organic, raw | | | | | |
| 2511 | Wheat and products | | | 1030227 | | | Wheat, grown non-organic, raw | | | | | |
| 2511 | Wheat and products | | | 1030228 | | | Wheat, raw | | | | | |
| 2511 | Wheat and products | | | 1030230 | | | Wheat, raw | | | | | |
| 2511 | Wheat and products | | | 1030234 | | | Wheat, whole grain, raw | | | | | |
| 2511 | Wheat and products | | | 1030235 | | | Wheat, whole grain, raw | | | | | |
| 2511 | Wheat and products | | | 1030237 | | | Wheat, with gluten, raw | | | | | |
| 2513 | Barley and products | | | 1060036 | | | Barley, dried | | | | | |
| 2513 | Barley and products | | | 1060038 | | | Barley, grain, raw | | | | | |
| 2513 | Barley and products | | | 1060045 | | | Barley, raw | | | | | |
| 2513 | Barley and products | | | 1060046 | | | Barley, Sunnita, raw, 25 kGy irradiated | | | | | |
| 2513 | Barley and products | | | 1060050 | | | Barley, Sunnita, raw | | | | | |
| 2513 | Barley and products | | | 1060053 | | | Barley, whole grain, dried | | | | | |
| 2514 | Maize and products | | | 1020022 | | | Corn, 113, raw | | | | | |
| 2514 | Maize and products | | | 1020023 | | | Corn, 12, raw | | | | | |
| 2514 | Maize and products | | | 1020024 | | | Corn, 14, raw | | | | | |
| 2514 | Maize and products | | | 1020025 | | | Corn, 22, raw | | | | | |
| 2514 | Maize and products | | | 1020026 | | | Corn, 35, raw | | | | | |
| 2514 | Maize and products | | | 1020027 | | | Corn, 36, raw | | | | | |
| 2514 | Maize and products | | | 1020028 | | | Corn, 37, raw | | | | | |
| 2514 | Maize and products | | | 1020029 | | | Corn, 41, raw | | | | | |
| 2514 | Maize and products | | | 1020030 | | | Corn, 42, raw | | | | | |
| 2514 | Maize and products | | | 1020031 | | | Corn, 43, raw | | | | | |
| 2514 | Maize and products | | | 1020032 | | | Corn, 91, raw | | | | | |
| 2514 | Maize and products | | | 1020037 | | | Corn, LH82xB73, raw | | | | | |
| 2514 | Maize and products | | | 1020038 | | | Corn, LH82xB73, raw | | | | | |
| 2514 | Maize and products | | | 1020041 | | | Corn, Mugtama 45, raw | | | | | |
| 2514 | Maize and products | | | 1020042 | | | Corn, NK603xB73 (glyphosate tolerant), raw | | | | | |
| 2514 | Maize and products | | | 1020043 | | | Corn, NK603xB73 (glyphosate tolerant), raw | | | | | |
| 2514 | Maize and products | | | 1020045 | | | Corn, raw | | | | | |
| 2514 | Maize and products | | | 1020048 | | | Corn, Texas 17W, raw | | | | | |
| 2514 | Maize and products | | | 1020065 | | | Maize, 1F5924, dried | | | | | |
| 2514 | Maize and products | | | 1020066 | | | Maize, 2E4794, dried | | | | | |
| 2514 | Maize and products | | | 1020067 | | | Maize, 2E5305, dried | | | | | |
| 2514 | Maize and products | | | 1020068 | | | Maize, 3E4824, dried | | | | | |
| 2514 | Maize and products | | | 1020073 | | | Maize, Bc 183 (1985), full grain maturity, raw | | | | | |
| 2514 | Maize and products | | | 1020074 | | | Maize, Bc 183 (1985), full grain maturity, raw | | | | | |
| 2514 | Maize and products | | | 1020080 | | | Maize, Bc 183 (1986), full grain maturity, raw | | | | | |
| 2514 | Maize and products | | | 1020081 | | | Maize, Bc 183 (1986), full grain maturity, raw | | | | | |
| 2514 | Maize and products | | | 1020087 | | | Maize, Bc 196 (1985), full grain maturity, raw | | | | | |
| 2514 | Maize and products | | | 1020088 | | | Maize, Bc 196 (1985), full grain maturity, raw | | | | | |
| 2514 | Maize and products | | | 1020094 | | | Maize, Bc 196 (1986), full grain maturity, raw | | | | | |
| 2514 | Maize and products | | | 1020095 | | | Maize, Bc 196 (1986), full grain maturity, raw | | | | | |
| 2514 | Maize and products | | | 1020101 | | | Maize, Bc 488 (1985), full grain maturity, raw | | | | | |
| 2514 | Maize and products | | | 1020102 | | | Maize, Bc 488 (1985), full grain maturity, raw | | | | | |
| 2514 | Maize and products | | | 1020108 | | | Maize, Bc 488 (1986), full grain maturity, raw | | | | | |
| 2514 | Maize and products | | | 1020109 | | | Maize, Bc 488 (1986), full grain maturity, raw | | | | | |
| 2514 | Maize and products | | | 1020117 | | | Maize, dried | | | | | |
| 2514 | Maize and products | | | 1020118 | | | Maize, grain, raw | | | | | |
| 2514 | Maize and products | | | 1020119 | | | Maize, grain, raw | | | | | |
| 2514 | Maize and products | | | 1020126 | | | Maize, T1A1 (hybrid), transgenic, negative trait expression (NK603: herbicide tolerance), raw | | |  |  |  |
| 2514 | Maize and products | | | 1020127 | | | Maize, T1A1 (hybrid), transgenic, positive trait expression (NK603: herbicide tolerance), raw | | |  |  |  |
| 2514 | Maize and products | | | 1020128 | | | Maize, T1A2 (hybrid), transgenic, negative trait expression (NK603: herbicide tolerance), raw | | |  |  |  |
| 2514 | Maize and products | | | 1020129 | | | Maize, T1A2 (hybrid), transgenic, positive trait expression (NK603: herbicide tolerance), raw | | |  |  |  |
| 2514 | Maize and products | | | 1020130 | | | Maize, T1B1 (hybrid), transgenic, negative trait expression (NK603: herbicide tolerance), raw | | |  |  |  |
| 2514 | Maize and products | | | 1020131 | | | Maize, T1B1 (hybrid), transgenic, positive trait expression (NK603: herbicide tolerance), raw | | |  |  |  |
| 2514 | Maize and products | | | 1020132 | | | Maize, T1B2 (hybrid), transgenic, negative trait expression (NK603: herbicide tolerance), raw | | |  |  |  |
| 2514 | Maize and products | | | 1020133 | | | Maize, T1B2 (hybrid), transgenic, positive trait expression (NK603: herbicide tolerance), raw | | |  |  |  |
| 2514 | Maize and products | | | 1020134 | | | Maize, tender, raw | | |  |  |  |
| 2514 | Maize and products | | | 1020135 | | | Maize, tender, sweet, raw | | | | | |
| 2514 | Maize and products | | | 1020137 | | | Maize, white, whole dried kernels | | | | | |
| 2514 | Maize and products | | | 1020138 | | | Maize, white, whole grain, dried | | | | | |
| 2514 | Maize and products | | | 1020139 | | | Maize, white, whole grain, fresh, raw | | | | | |
| 2514 | Maize and products | | | 1020140 | | | Maize, white, whole grain, raw | | | | | |
| 2515 | Rye and products | | | 1060226 | | | Rye, grain, raw | | | | | |
| 2516 | Oats | | | 1060192 | | | Oats, raw | | |  |  |  |
| 2517 | Millet and products | | | 1050075 | | | Bajra, raw | | |  |  |  |
| 2517 | Millet and products | | | 1050112 | | | Millet, bulrush, grain, raw | | |  |  |  |
| 2517 | Millet and products | | | 1050116 | | | Millet, raw | | |  |  |  |
| 2517 | Millet and products | | | 1050117 | | | Millet, raw | | |  |  |  |
| 2517 | Millet and products | | | 1050121 | | | Millet, whole grain, raw | | |  |  |  |
| 2517 | Millet and products | | | 1050183 | | | Pearl millet, B 1, yellowish, raw | | |  |  |  |
| 2517 | Millet and products | | | 1050184 | | | Pearl millet, B 2, gray, raw | | |  |  |  |
| 2517 | Millet and products | | | 1050185 | | | Pearl millet, Gampela, whole, raw | | |  |  |  |
| 2517 | Millet and products | | | 1050186 | | | Pearl millet, Gampela, yellow, raw, hand-decorticated | | |  |  |  |
| 2517 | Millet and products | | | 1050187 | | | Pearl millet, Gampela, yellow, raw, mechanically-decorticated | | |  |  |  |
| 2517 | Millet and products | | | 1050188 | | | Pearl millet, Gampela, yellow, raw | | |  |  |  |
| 2517 | Millet and products | | | 1050189 | | | Pearl millet, IKMP 1, yellowish, raw | | |  |  |  |
| 2517 | Millet and products | | | 1050190 | | | Pearl millet, IKMP 2, gray yellow, raw | | |  |  |  |
| 2517 | Millet and products | | | 1050191 | | | Pearl millet, IKMP 3, gray, raw | | |  |  |  |
| 2517 | Millet and products | | | 1050192 | | | Pearl millet, IKMP 5, gray light, raw | | |  |  |  |
| 2517 | Millet and products | | | 1050193 | | | Pearl millet, IKMP-5, whole, raw | | |  |  |  |
| 2517 | Millet and products | | | 1050194 | | | Pearl millet, IKMV 8201, gray yellow, raw | | |  |  |  |
| 2517 | Millet and products | | | 1050195 | | | Pearl millet, KM, gray, raw | | |  |  |  |
| 2517 | Millet and products | | | 1050196 | | | Pearl millet, L. Nahartenga, yellow blade, raw | | |  |  |  |
| 2517 | Millet and products | | | 1050197 | | | Pearl millet, L. Zatiib, gray yellow, raw | | |  |  |  |
| 2517 | Millet and products | | | 1050200 | | | Pearl millet, raw | | |  |  |  |
| 2517 | Millet and products | | | 1050201 | | | Pearl millet, raw | | |  |  |  |
| 2517 | Millet and products | | | 1050202 | | | Pearl millet, SG, gray, raw | | |  |  |  |
| 2517 | Millet and products | | | 1050203 | | | Pearl millet, SOSATC 88, gray yellow, raw | | |  |  |  |
| 2517 | Millet and products | | | 1050204 | | | Pearl millet, TK, yellow, raw | | |  |  |  |
| 2517 | Millet and products | | | 1050206 | | | Pearl millet, XX, gray, raw | | |  |  |  |
| 2517 | Millet and products | | | 1050210 | | | Proso millet, TNAU-145, brown | | |  |  |  |
| 2517 | Millet and products | | | 1050211 | | | Proso millet, TNAU-145, polished | | |  |  |  |
| 2517 | Millet and products | | | 1050212 | | | Proso millet, TNAU-145, whole | | |  |  |  |
| 2517 | Millet and products | | | 1050213 | | | Ragi, raw | | |  |  |  |
| 2517 | Millet and products | | | 1050214 | | | Samai, raw | | |  |  |  |
| 2517 | Millet and products | | | 1050215 | | | Varagu, raw | | |  |  |  |
| 2518 | Sorghum and products | | | 1040007 | | | Jowar, raw | | |  |  |  |
| 2518 | Sorghum and products | | | 1040065 | | | Sorghum, Fibmigou, white, raw, hand-decorticated | | |  |  |  |
| 2518 | Sorghum and products | | | 1040066 | | | Sorghum, Fibmigou, white, raw | | |  |  |  |
| 2518 | Sorghum and products | | | 1040067 | | | Sorghum, grain, white, raw | | |  |  |  |
| 2518 | Sorghum and products | | | 1040068 | | | Sorghum, raw | | |  |  |  |
| 2518 | Sorghum and products | | | 1040069 | | | Sorghum, red, whole grain, dried | | |  |  |  |
| 2518 | Sorghum and products | | | 1040070 | | | Sorghum, white, steamed | | |  |  |  |
| 2518 | Sorghum and products | | | 1040071 | | | Sorghum, white, steamed, dried | | |  |  |  |
| 2518 | Sorghum and products | | | 1040072 | | | Sorghum, white, whole grain, dried | | |  |  |  |
| 2518 | Sorghum and products | | | 1040073 | | | Sorghum, whole grain, red, raw | | |  |  |  |
| 2518 | Sorghum and products | | | 1040074 | | | Sorghum, whole grain, white, raw | | |  |  |  |
| 2520 | Cereals, Other | | | 1030133 | | | Triticale, grain, raw | | |  |  |  |
| 2520 | Cereals, Other | | | 1050077 | | | Fonio, Dieni, ready-to-cook, raw, dehulled | | |  |  |  |
| 2520 | Cereals, Other | | | 1050078 | | | Fonio, Finiba, ready-to-cook, raw, dehulled | | |  |  |  |
| 2520 | Cereals, Other | | | 1050079 | | | Fonio, Finiba/Kassangara, ready-to-cook, raw, dehulled | | |  |  |  |
| 2520 | Cereals, Other | | | 1050080 | | | Fonio, Kassangara, ready-to-cook, raw, dehulled | | |  |  |  |
| 2520 | Cereals, Other | | | 1050081 | | | Fonio, Peazo, ready-to-cook, raw, dehulled | | |  |  |  |
| 2520 | Cereals, Other | | | 1050082 | | | Fonio, Peazo, ready-to-cook, raw, dehulled | | |  |  |  |
| 2520 | Cereals, Other | | | 1050083 | | | Fonio, Petama, ready-to-cook, raw, dehulled | | |  |  |  |
| 2520 | Cereals, Other | | | 1050084 | | | Fonio, Peye, ready-to-cook, raw, dehulled | | |  |  |  |
| 2520 | Cereals, Other | | | 1050085 | | | Fonio, Tama, ready-to-cook, raw, dehulled | | |  |  |  |
| 2520 | Cereals, Other | | | 1050086 | | | Fonio, Tamabe, ready-to-cook,raw, dehulled | | |  |  |  |
| 2520 | Cereals, Other | | | 1050087 | | | Fonio, Tamatioi, ready-to-cook, raw, dehulled | | |  |  |  |
| 2520 | Cereals, Other | | | 1050088 | | | Fonio, Tioi, ready-to-cook, raw, dehulled | | |  |  |  |
| 2520 | Cereals, Other | | | 1060002 | | | Amaranth, brown, grain, dried | | |  |  |  |
| 2520 | Cereals, Other | | | 1060004 | | | Amaranth, Centenario, grain, raw | | |  |  |  |
| 2520 | Cereals, Other | | | 1060006 | | | Amaranth, Oscar Blanco, grain, raw | | |  |  |  |
| 2520 | Cereals, Other | | | 1060007 | | | Amaranth, red, grain, dried | | |  |  |  |
| 2520 | Cereals, Other | | | 1060008 | | | Amaranth, white, grain, dried | | |  |  |  |
| 2520 | Cereals, Other | | | 1060112 | | | Buckwheat, broken whole grain, dried | | |  |  |  |
| 2520 | Cereals, Other | | | 1060116 | | | Buckwheat, Kora, raw | | |  |  |  |
| 2520 | Cereals, Other | | | 1060117 | | | Buckwheat, whole grain, dried | | |  |  |  |
| 2520 | Cereals, Other | | | 1060222 | | | Quinoa, raw | | |  |  |  |
| 2520 | Cereals, Other | | | 1060230 | | | Teff, mixed red and white, whole grain, raw | | |  |  |  |
| 2520 | Cereals, Other | | | 1060231 | | | Teff, red, whole grain, raw | | |  |  |  |
| 2520 | Cereals, Other | | | 1060232 | | | Teff, white, whole grain, raw | | |  |  |  |
| 2520 | Cereals, Other | | | 1060233 | | | Teff, whole grain, dried | | |  |  |  |
| 2520 | Cereals, Other | | | 4010007 | | | Amaranth globe, ABS-38-AWKA, raw | | |  |  |  |
| 2520 | Cereals, Other | | | 4010009 | | | Amaranth globe, AKS-33-EKPENE EDIENE x ABS-38-AWKA, raw | | |  |  |  |
| 2520 | Cereals, Other | | | 4010011 | | | Amaranth globe, AKS-33-EKPENE EDIENE, raw | | |  |  |  |
| 2520 | Cereals, Other | | | 4010013 | | | Amaranth globe, EBS-15-NKALAGU x ABS-38-AKWA, raw | | |  |  |  |
| 2520 | Cereals, Other | | | 4010015 | | | Amaranth globe, EBS-15-NKALAGU x AKS-33-EKPENE EDIENE, raw | | |  |  |  |
| 2520 | Cereals, Other | | | 4010017 | | | Amaranth globe, EBS-15-NKALAGU x IMS-20-NJIABA, raw | | |  |  |  |
| 2520 | Cereals, Other | | | 4010019 | | | Amaranth globe, EBS-15-NKALAGU, raw | | |  |  |  |
| 2520 | Cereals, Other | | | 4010021 | | | Amaranth globe, ENS-08-MBU x ABS-38-AWKA, raw | | |  |  |  |
| 2520 | Cereals, Other | | | 4010023 | | | Amaranth globe, ENS-08-MBU x AKS-33-EKPENE EDIENE, raw | | |  |  |  |
| 2520 | Cereals, Other | | | 4010025 | | | Amaranth globe, ENS-08-MBU x EBS-15-NKALAGU, raw | | |  |  |  |
| 2520 | Cereals, Other | | | 4010027 | | | Amaranth globe, ENS-08-MBU x IMS-20-NJIABA, raw | | |  |  |  |
| 2520 | Cereals, Other | | | 4010029 | | | Amaranth globe, ENS-08-MBU, raw | | |  |  |  |
| 2520 | Cereals, Other | | | 4010031 | | | Amaranth globe, IMS-20-NJIABA x ABS-38-AWKA, raw | | |  |  |  |
| 2520 | Cereals, Other | | | 4010033 | | | Amaranth globe, IMS-20-NJIABA x AKS-33-EKPENE EDIENE, raw | | |  |  |  |
| 2520 | Cereals, Other | | | 4010035 | | | Amaranth globe, IMS-20-NJIABA, raw | | |  |  |  |
| 2531 | Potatoes and products | | | 2010003 | | | Potato, baby red skin, raw | | |  |  |  |
| 2531 | Potatoes and products | | | 2010005 | | | Potato, brown skin, big, raw | | |  |  |  |
| 2531 | Potatoes and products | | | 2010006 | | | Potato, brown skin, small, raw | | |  |  |  |
| 2531 | Potatoes and products | | | 2010015 | | | Potato, Irish potato, raw | | |  |  |  |
| 2531 | Potatoes and products | | | 2010016 | | | Potato, Kennebec, raw, unpeeled | | |  |  |  |
| 2531 | Potatoes and products | | | 2010019 | | | Potato, Norland, raw, unpeeled | | |  |  |  |
| 2531 | Potatoes and products | | | 2010020 | | | Potato, raw | | |  |  |  |
| 2531 | Potatoes and products | | | 2010021 | | | Potato, Red LaSoda, raw, unpeeled | | |  |  |  |
| 2531 | Potatoes and products | | | 2010022 | | | Potato, red skin, raw | | |  |  |  |
| 2531 | Potatoes and products | | | 2010023 | | | Potato, red skin, raw | | |  |  |  |
| 2531 | Potatoes and products | | | 2010025 | | | Potato, Russet Burbank, raw, unpeeled | | |  |  |  |
| 2531 | Potatoes and products | | | 2010026 | | | Potato, Russet Norkotah, raw, unpeeled | | |  |  |  |
| 2531 | Potatoes and products | | | 2010027 | | | Potato, Russet, raw | | |  |  |  |
| 2531 | Potatoes and products | | | 2010029 | | | Potato, Superior, raw, unpeeled | | |  |  |  |
| 2531 | Potatoes and products | | | 2010035 | | | Potato, Yellow Finn, raw, unpeeled | | |  |  |  |
| 2532 | Cassava and products | | | 2020016 | | | Cassava, Kello 44/72, roots, raw | | |  |  |  |
| 2532 | Cassava and products | | | 2020020 | | | Cassava, Quelle 104/72, roots, raw | | |  |  |  |
| 2532 | Cassava and products | | | 2020021 | | | Cassava, raw | | |  |  |  |
| 2533 | Sweet potatoes | | | 2010044 | | | Sweet potato, brown skin, raw | | |  |  |  |
| 2533 | Sweet potatoes | | | 2010049 | | | Sweet potato, pink skin, raw | | |  |  |  |
| 2533 | Sweet potatoes | | | 2010050 | | | Sweet potato, raw | | |  |  |  |
| 2533 | Sweet potatoes | | | 2010051 | | | Sweet potato, raw | | |  |  |  |
| 2533 | Sweet potatoes | | | 2010053 | | | Sweet potato, white flesh, raw | | |  |  |  |
| 2534 | Roots, Other | | | 2020027 | | | Cocoyam, raw | | |  |  |  |
| 2534 | Roots, Other | | | 2030015 | | | Taro, raw | | |  |  |  |
| 2534 | Roots, Other | | | 2040001 | | | Taro (eddoe), raw | | |  |  |  |
| 2534 | Roots, Other | | | 2050001 | | | American yam bean root, IRNAS NÂ° 11 | | |  |  |  |
| 2534 | Roots, Other | | | 2050002 | | | American yam bean root, IRNAS NÂ° 4 | | |  |  |  |
| 2534 | Roots, Other | | | 2050003 | | | American yam bean root, IRNAS NÂ° 5 | | |  |  |  |
| 2534 | Roots, Other | | | 2050004 | | | American yam bean root, IRNAS NÂ° 9 | | |  |  |  |
| 2534 | Roots, Other | | | 2050005 | | | American yam bean root, Local | | |  |  |  |
| 2534 | Roots, Other | | | 2050007 | | | Anchote, roots, whole, raw | | |  |  |  |
| 2534 | Roots, Other | | | 2050016 | | | Jerusalem artichoke, raw | | |  |  |  |
| 2535 | Yams | | | 2040006 | | | Yam, Elephant, raw | | |  |  |  |
| 2535 | Yams | | | 2040008 | | | Yam, Hingurala, raw | | |  |  |  |
| 2535 | Yams | | | 2040009 | | | Yam, Ini ata, raw | | |  |  |  |
| 2535 | Yams | | | 2040010 | | | Yam, Kahata ala, raw | | |  |  |  |
| 2535 | Yams | | | 2040011 | | | Yam, Kombuwalli, raw | | |  |  |  |
| 2535 | Yams | | | 2040012 | | | Yam, ordinary, raw | | |  |  |  |
| 2535 | Yams | | | 2040013 | | | Yam, Raja aIa, raw | | |  |  |  |
| 2535 | Yams | | | 2040014 | | | Yam, Rata ala, raw | | |  |  |  |
| 2535 | Yams | | | 2040015 | | | Yam, raw | | |  |  |  |
| 2535 | Yams | | | 2040017 | | | Yam, Thambala, raw | | |  |  |  |
| 2535 | Yams | | | 2040018 | | | Yam, tuber, raw | | |  |  |  |
| 2535 | Yams | | | 2040020 | | | Yam, wild, raw | | |  |  |  |
| 2536 | Sugar cane | | | 14050002 | | | Sugar | | |  |  |  |
| 2537 | Sugar beet | | | 14050002 | | | Sugar | | |  |  |  |
| 2541 | Sugar non-centrifugal | | | 14050001 | | | Jaggery | | |  |  |  |
| 2542 | Sugar (Raw Equivalent) | | | 14050002 | | | Sugar | | |  |  |  |
| 2546 | Beans | | | 3010046 | | | Bean, black turtle, dried | | |  |  |  |
| 2546 | Beans | | | 3010047 | | | Bean, black, split, raw | | |  |  |  |
| 2546 | Beans | | | 3010048 | | | Bean, brown, dried | | |  |  |  |
| 2546 | Beans | | | 3010049 | | | Bean, butter, dried | | |  |  |  |
| 2546 | Beans | | | 3010051 | | | Bean, great northern, dried | | |  |  |  |
| 2546 | Beans | | | 3010053 | | | Bean, Haricot kidney, raw | | |  |  |  |
| 2546 | Beans | | | 3010054 | | | Bean, pink, dried | | |  |  |  |
| 2546 | Beans | | | 3010056 | | | Bean, pinto and red, dried | | |  |  |  |
| 2546 | Beans | | | 3010057 | | | Bean, pinto, dried | | |  |  |  |
| 2546 | Beans | | | 3010064 | | | Bean, 'Vigna aconitifolia', seed, mature, sun-dried | | |  |  |  |
| 2546 | Beans | | | 3010065 | | | Bean, 'Vigna aconitifolia', seed, mature, sun-dried | | |  |  |  |
| 2546 | Beans | | | 3010066 | | | Bean, 'Vigna ambacensis', TVnu 306, Cream, seed, raw | | |  |  |  |
| 2546 | Beans | | | 3010067 | | | Bean, 'Vigna bourneae', seed, mature, sun-dried | | |  |  |  |
| 2546 | Beans | | | 3010068 | | | Bean, 'Vigna luteola', TVnu 24, Brown, seed, raw | | |  |  |  |
| 2546 | Beans | | | 3010069 | | | Bean, 'Vigna luteola', TVnu 29, Brown, seed, raw | | |  |  |  |
| 2546 | Beans | | | 3010070 | | | Bean, 'Vigna oblongifolia', TVnu 38, Brown-black, seed, raw | | |  |  |  |
| 2546 | Beans | | | 3010073 | | | Bean, 'Vigna racemosa', raw | | |  |  |  |
| 2546 | Beans | | | 3010074 | | | Bean, 'Vigna racemosa', TVnu 105, Brown mottled, seed, raw | | |  |  |  |
| 2546 | Beans | | | 3010075 | | | Bean, 'Vigna radiata', var. sublota, seed, mature, sun-dried | | |  |  |  |
| 2546 | Beans | | | 3010076 | | | Bean, 'Vigna reticulata', TVnu 225, Light Brown, seed, raw | | |  |  |  |
| 2546 | Beans | | | 3010077 | | | Bean, 'Vigna trilobata', seed, mature, sun-dried | | |  |  |  |
| 2546 | Beans | | | 3010078 | | | Bean, 'Vigna umbellata', seed, mature, sun-dried | | |  |  |  |
| 2546 | Beans | | | 3010079 | | | Bean, 'Vigna unguiculata dekindtiana', TVnu 278, Brown, seed, raw | | |  |  |  |
| 2546 | Beans | | | 3010080 | | | Bean, 'Vigna unguiculata', Black, seed, mature, sun-dried | | |  |  |  |
| 2546 | Beans | | | 3010081 | | | Bean, 'Vigna unguiculata', Maroon, seed, mature, sun-dried | | |  |  |  |
| 2546 | Beans | | | 3010082 | | | Bean, 'Vigna unguiculata', seed, mature, raw | | |  |  |  |
| 2546 | Beans | | | 3010083 | | | Bean, 'Vigna unguiculata', seed, mature, sun-dried | | |  |  |  |
| 2546 | Beans | | | 3010084 | | | Bean, 'Vigna vexillata macrosperma', TVnu 72, Green-brown, seed, raw | | |  |  |  |
| 2546 | Beans | | | 3010085 | | | Bean, 'Vigna vexillata macrosperma', TVnu 73A, Black, seed, raw | | |  |  |  |
| 2546 | Beans | | | 3010086 | | | Bean, 'Vigna vexillata', seed, mature, sun-dried | | |  |  |  |
| 2546 | Beans | | | 3010087 | | | Bean, 'Vigna vexillata', TVnu 71, Brown, seed, raw | | |  |  |  |
| 2546 | Beans | | | 3010124 | | | Black bean, raw | | |  |  |  |
| 2546 | Beans | | | 3010125 | | | Black gram, dal | | |  |  |  |
| 2546 | Beans | | | 3010127 | | | Black gram, whole, raw | | |  |  |  |
| 2546 | Beans | | | 3010191 | | | Common bean, A321, raw | | |  |  |  |
| 2546 | Beans | | | 3010192 | | | Common bean, A321, raw | | |  |  |  |
| 2546 | Beans | | | 3010193 | | | Common bean, A321, raw | | |  |  |  |
| 2546 | Beans | | | 3010194 | | | Common bean, A321, raw | | |  |  |  |
| 2546 | Beans | | | 3010195 | | | Common bean, A410, raw | | |  |  |  |
| 2546 | Beans | | | 3010196 | | | Common bean, A410, raw | | |  |  |  |
| 2546 | Beans | | | 3010197 | | | Common bean, A410, raw | | |  |  |  |
| 2546 | Beans | | | 3010198 | | | Common bean, Baki wake, seed, raw | | |  |  |  |
| 2546 | Beans | | | 3010203 | | | Common bean, Cal1, raw | | |  |  |  |
| 2546 | Beans | | | 3010204 | | | Common bean, Calima, raw | | |  |  |  |
| 2546 | Beans | | | 3010205 | | | Common bean, Calima, raw | | |  |  |  |
| 2546 | Beans | | | 3010206 | | | Common bean, Calima, raw | | |  |  |  |
| 2546 | Beans | | | 3010207 | | | Common bean, Calima, raw | | |  |  |  |
| 2546 | Beans | | | 3010208 | | | Common bean, Dermaso, raw | | |  |  |  |
| 2546 | Beans | | | 3010209 | | | Common bean, Diamante Negro, seed, raw | | |  |  |  |
| 2546 | Beans | | | 3010212 | | | Common bean, Dore, raw | | |  |  |  |
| 2546 | Beans | | | 3010213 | | | Common bean, Dore, raw | | |  |  |  |
| 2546 | Beans | | | 3010214 | | | Common bean, Horoz, raw | | |  |  |  |
| 2546 | Beans | | | 3010215 | | | Common bean, Inta Linea 628-08, bright red, raw | | |  |  |  |
| 2546 | Beans | | | 3010216 | | | Common bean, Inta Linea 628-09, dark red, raw | | |  |  |  |
| 2546 | Beans | | | 3010217 | | | Common bean, Inta Rojo, non-uniform red, raw | | |  |  |  |
| 2546 | Beans | | | 3010218 | | | Common bean, Kidney, 10kGy irradiated, raw | | |  |  |  |
| 2546 | Beans | | | 3010219 | | | Common bean, Kidney, 5kGy irradiated, raw | | |  |  |  |
| 2546 | Beans | | | 3010220 | | | Common bean, Kidney, 7.5kGy irradiated, raw | | |  |  |  |
| 2546 | Beans | | | 3010222 | | | Common bean, Kidney, fresh, raw | | |  |  |  |
| 2546 | Beans | | | 3010223 | | | Common bean, Kidney, raw | | |  |  |  |
| 2546 | Beans | | | 3010224 | | | Common bean, Kidney, raw | | |  |  |  |
| 2546 | Beans | | | 3010225 | | | Common bean, Kidney, red, raw | | |  |  |  |
| 2546 | Beans | | | 3010226 | | | Common bean, Kidney, red, whole, sun-dried | | |  |  |  |
| 2546 | Beans | | | 3010228 | | | Common bean, Kwakiul, seed, raw | | |  |  |  |
| 2546 | Beans | | | 3010230 | | | Common bean, Ouro branco, seed, raw | | |  |  |  |
| 2546 | Beans | | | 3010234 | | | Common bean, Perola, seed, raw | | |  |  |  |
| 2546 | Beans | | | 3010237 | | | Common bean, Pink-mottled cream, seed, raw | | |  |  |  |
| 2546 | Beans | | | 3010241 | | | Common bean, Pinto, raw | | |  |  |  |
| 2546 | Beans | | | 3010242 | | | Common bean, raw | | |  |  |  |
| 2546 | Beans | | | 3010243 | | | Common bean, Seker, raw | | |  |  |  |
| 2546 | Beans | | | 3010244 | | | Common bean, Sen46, black, raw | | |  |  |  |
| 2546 | Beans | | | 3010246 | | | Common bean, TalismÃ£, seed, raw | | |  |  |  |
| 2546 | Beans | | | 3010249 | | | Common bean, var. A321, seed, raw | | |  |  |  |
| 2546 | Beans | | | 3010250 | | | Common bean, var. A321, seed, raw | | |  |  |  |
| 2546 | Beans | | | 3010251 | | | Common bean, var. A321, seed, raw | | |  |  |  |
| 2546 | Beans | | | 3010252 | | | Common bean, var. A321, seed, raw | | |  |  |  |
| 2546 | Beans | | | 3010253 | | | Common bean, var. A410, seed, raw | | |  |  |  |
| 2546 | Beans | | | 3010254 | | | Common bean, var. A410, seed, raw | | |  |  |  |
| 2546 | Beans | | | 3010255 | | | Common bean, var. A410, seed, raw | | |  |  |  |
| 2546 | Beans | | | 3010256 | | | Common bean, var. Calima, seed, raw | | |  |  |  |
| 2546 | Beans | | | 3010257 | | | Common bean, var. Calima, seed, raw | | |  |  |  |
| 2546 | Beans | | | 3010258 | | | Common bean, var. Calima, seed, raw | | |  |  |  |
| 2546 | Beans | | | 3010259 | | | Common bean, var. Calima, seed, raw | | |  |  |  |
| 2546 | Beans | | | 3010260 | | | Common bean, var. Dore de Kirundo, seed, raw | | |  |  |  |
| 2546 | Beans | | | 3010261 | | | Common bean, var. Dore de Kirundo, seed, raw | | |  |  |  |
| 2546 | Beans | | | 3010262 | | | Common bean, White, seed, raw | | |  |  |  |
| 2546 | Beans | | | 3010266 | | | Common bean, white, whole, raw | | |  |  |  |
| 2546 | Beans | | | 3010405 | | | Faba bean, Qidou 2, raw | | |  |  |  |
| 2546 | Beans | | | 3010406 | | | Faba bean, Qidou 2, raw | | |  |  |  |
| 2546 | Beans | | | 3010437 | | | Field bean, black, raw | | |  |  |  |
| 2546 | Beans | | | 3010438 | | | Field bean, brown, raw | | |  |  |  |
| 2546 | Beans | | | 3010439 | | | Field bean, white, raw | | |  |  |  |
| 2546 | Beans | | | 3010443 | | | Green gram, dal | | |  |  |  |
| 2546 | Beans | | | 3010444 | | | Green gram, raw | | |  |  |  |
| 2546 | Beans | | | 3010445 | | | Green gram, split, raw | | |  |  |  |
| 2546 | Beans | | | 3010450 | | | Green gram, whole, raw | | |  |  |  |
| 2546 | Beans | | | 3010457 | | | Haricot bean, white, whole grain, sun-dried | | |  |  |  |
| 2546 | Beans | | | 3010504 | | | Lima bean, fresh, raw | | |  |  |  |
| 2546 | Beans | | | 3010506 | | | Lima bean, dried | | |  |  |  |
| 2546 | Beans | | | 3010507 | | | Lima bean, raw | | |  |  |  |
| 2546 | Beans | | | 3010545 | | | Moth bean, raw | | |  |  |  |
| 2546 | Beans | | | 3010555 | | | Mung bean, Asha, raw | | |  |  |  |
| 2546 | Beans | | | 3010557 | | | Mung bean, MH 124, raw | | |  |  |  |
| 2546 | Beans | | | 3010558 | | | Mung bean, MH 125, raw | | |  |  |  |
| 2546 | Beans | | | 3010559 | | | Mung bean, MH 318, raw | | |  |  |  |
| 2546 | Beans | | | 3010560 | | | Mung bean, MH 421, raw | | |  |  |  |
| 2546 | Beans | | | 3010561 | | | Mung bean, MH 539, raw | | |  |  |  |
| 2546 | Beans | | | 3010562 | | | Mung bean, MH 560, raw | | |  |  |  |
| 2546 | Beans | | | 3010563 | | | Mung bean, MH 564, raw | | |  |  |  |
| 2546 | Beans | | | 3010572 | | | Mung bean, Muskan, raw | | |  |  |  |
| 2546 | Beans | | | 3010581 | | | Mung bean, raw | | |  |  |  |
| 2546 | Beans | | | 3010582 | | | Mung bean, raw | | |  |  |  |
| 2546 | Beans | | | 3010583 | | | Mung bean, Satya, raw | | |  |  |  |
| 2546 | Beans | | | 3010590 | | | Mung bean, var. Giza-1, seed, whole, raw | | |  |  |  |
| 2546 | Beans | | | 3010632 | | | Rajmah, black, raw | | |  |  |  |
| 2546 | Beans | | | 3010633 | | | Rajmah, brown, raw | | |  |  |  |
| 2546 | Beans | | | 3010634 | | | Rajmah, red, raw | | |  |  |  |
| 2546 | Beans | | | 3010641 | | | Rice bean, Beziamah, seeds, light brown, raw | | |  |  |  |
| 2546 | Beans | | | 3010642 | | | Rice bean, Beziarnah, oven-dried | | |  |  |  |
| 2546 | Beans | | | 3010645 | | | Rice bean, raw | | |  |  |  |
| 2546 | Beans | | | 3010646 | | | Rice bean, RBL-121, oven-dried | | |  |  |  |
| 2546 | Beans | | | 3010651 | | | Rice bean, RBL-121, seeds, greenish brown, raw | | |  |  |  |
| 2546 | Beans | | | 3010652 | | | Rice bean, RBL-4, oven-dried | | |  |  |  |
| 2546 | Beans | | | 3010657 | | | Rice bean, RBL-4, seed, brown, raw | | |  |  |  |
| 2546 | Beans | | | 3010658 | | | Rice bean, RBL-6, oven-dried | | |  |  |  |
| 2546 | Beans | | | 3010662 | | | Rice bean, RBL-6, seed, light brown, raw | | |  |  |  |
| 2546 | Beans | | | 3010693 | | | Tepary bean, Domestic brown, raw | | |  |  |  |
| 2546 | Beans | | | 3010694 | | | Tepary bean, Domestic brown, raw | | |  |  |  |
| 2546 | Beans | | | 3010695 | | | Tepary bean, Domestic white, raw | | |  |  |  |
| 2546 | Beans | | | 3010696 | | | Tepary bean, Domestic white, raw | | |  |  |  |
| 2546 | Beans | | | 3010697 | | | Tepary bean, wild, raw | | |  |  |  |
| 2547 | Peas | | | 3010052 | | | Bean, green, split, raw | | |  |  |  |
| 2547 | Peas | | | 3010441 | | | Grass pea, split, dried | | |  |  |  |
| 2547 | Peas | | | 3010442 | | | Grass pea, split, dried | | |  |  |  |
| 2547 | Peas | | | 3010451 | | | Grass pea, dried | | |  |  |  |
| 2547 | Peas | | | 3010592 | | | Pea, 10kGy irradiated, dried | | |  |  |  |
| 2547 | Peas | | | 3010593 | | | Pea, 5kGy irradiated, dried | | |  |  |  |
| 2547 | Peas | | | 3010594 | | | Pea, 7.5kGy irradiated, dried | | |  |  |  |
| 2547 | Peas | | | 3010595 | | | Pea, Agat, dried | | |  |  |  |
| 2547 | Peas | | | 3010596 | | | Pea, Agra, dried | | |  |  |  |
| 2547 | Peas | | | 3010597 | | | Pea, Albatros, dried | | |  |  |  |
| 2547 | Peas | | | 3010598 | | | Pea, Ametyst, dried | | |  |  |  |
| 2547 | Peas | | | 3010599 | | | Pea, Diament, dried | | |  |  |  |
| 2547 | Peas | | | 3010600 | | | Pea, dried | | |  |  |  |
| 2547 | Peas | | | 3010601 | | | Pea, dried | | |  |  |  |
| 2547 | Peas | | | 3010602 | | | Pea, dried | | |  |  |  |
| 2547 | Peas | | | 3010603 | | | Pea, dried | | |  |  |  |
| 2547 | Peas | | | 3010607 | | | Pea, Ergo, dried | | |  |  |  |
| 2547 | Peas | | | 3010608 | | | Pea, field, whole grain, sun-dried | | |  |  |  |
| 2547 | Peas | | | 3010609 | | | Pea, Finale, whole seed, dried | | |  |  |  |
| 2547 | Peas | | | 3010610 | | | Pea, Hermes, dried | | |  |  |  |
| 2547 | Peas | | | 3010611 | | | Pea, Imposant, whole seed, dried | | |  |  |  |
| 2547 | Peas | | | 3010612 | | | Pea, Karat, dried | | |  |  |  |
| 2547 | Peas | | | 3010613 | | | Pea, Koral, dried | | |  |  |  |
| 2547 | Peas | | | 3010614 | | | Pea, Kwestor, dried | | |  |  |  |
| 2547 | Peas | | | 3010615 | | | Pea, Piast, dried | | |  |  |  |
| 2547 | Peas | | | 3010616 | | | Pea, Rodan, dried | | |  |  |  |
| 2547 | Peas | | | 3010617 | | | Pea, Rondo, whole seed, dried | | |  |  |  |
| 2547 | Peas | | | 3010618 | | | Pea, Rubin, dried | | |  |  |  |
| 2547 | Peas | | | 3010619 | | | Pea, split, dried | | |  |  |  |
| 2547 | Peas | | | 3010620 | | | Pea, split, dried | | |  |  |  |
| 2547 | Peas | | | 3010621 | | | Pea, Szafir, dried | | |  |  |  |
| 2547 | Peas | | | 3010622 | | | Pea, Tegma, dried | | |  |  |  |
| 2549 | Pulses, Other and products | | | 3010002 | | | African faba bean, seed, raw | | |  |  |  |
| 2549 | Pulses, Other and products | | | 3010006 | | | African locust bean, Boki, mature seeds, raw | | |  |  |  |
| 2549 | Pulses, Other and products | | | 3010009 | | | African locust bean, Obanliku, mature seeds, raw | | |  |  |  |
| 2549 | Pulses, Other and products | | | 3010012 | | | African locust bean, Obudu, mature seeds, raw | | |  |  |  |
| 2549 | Pulses, Other and products | | | 3010015 | | | African locust bean, raw | | |  |  |  |
| 2549 | Pulses, Other and products | | | 3010017 | | | African oil bean, raw | | |  |  |  |
| 2549 | Pulses, Other and products | | | 3010019 | | | African oil bean, seed, raw | | |  |  |  |
| 2549 | Pulses, Other and products | | | 3010023 | | | African yam bean, seed, raw | | |  |  |  |
| 2549 | Pulses, Other and products | | | 3010031 | | | Bambara groundnut, Ci12, raw | | |  |  |  |
| 2549 | Pulses, Other and products | | | 3010032 | | | Bambara groundnut, raw | | |  |  |  |
| 2549 | Pulses, Other and products | | | 3010033 | | | Bambara groundnut, raw | | |  |  |  |
| 2549 | Pulses, Other and products | | | 3010039 | | | Bambara groundnut, seed, raw | | |  |  |  |
| 2549 | Pulses, Other and products | | | 3010040 | | | Bambara groundnut, seed, red, raw | | |  |  |  |
| 2549 | Pulses, Other and products | | | 3010041 | | | Bambara groundnut, seed, white, raw | | |  |  |  |
| 2549 | Pulses, Other and products | | | 3010044 | | | Bambara groundnut, seed, raw | | |  |  |  |
| 2549 | Pulses, Other and products | | | 3010050 | | | Bean, 'Cassia laevigata', seed, raw | | |  |  |  |
| 2549 | Pulses, Other and products | | | 3010090 | | | Bean, White, California small white, raw | | |  |  |  |
| 2549 | Pulses, Other and products | | | 3010091 | | | Bean, 'Xylia xylocarpa', seed, sun-dried | | |  |  |  |
| 2549 | Pulses, Other and products | | | 3010100 | | | Bengal gram, brown, whole grain, raw | | |  |  |  |
| 2549 | Pulses, Other and products | | | 3010101 | | | Bengal gram, brown, whole grain, raw | | |  |  |  |
| 2549 | Pulses, Other and products | | | 3010102 | | | Bengal gram, brown, whole grain, raw | | |  |  |  |
| 2549 | Pulses, Other and products | | | 3010111 | | | Bengal gram, dal | | |  |  |  |
| 2549 | Pulses, Other and products | | | 3010117 | | | Bengal gram, white, whole grain, raw | | |  |  |  |
| 2549 | Pulses, Other and products | | | 3010118 | | | Bengal gram, white, whole grain, raw | | |  |  |  |
| 2549 | Pulses, Other and products | | | 3010122 | | | Bengal gram, whole, raw | | |  |  |  |
| 2549 | Pulses, Other and products | | | 3010123 | | | Bengal gram, whole, raw | | |  |  |  |
| 2549 | Pulses, Other and products | | | 3010132 | | | Broad bean, Giza 716, raw | | |  |  |  |
| 2549 | Pulses, Other and products | | | 3010133 | | | Broad bean, whole, raw | | |  |  |  |
| 2549 | Pulses, Other and products | | | 3010140 | | | Chickpea, C-235, grown organic, raw | | |  |  |  |
| 2549 | Pulses, Other and products | | | 3010142 | | | Chickpea, Desi, seed, raw | | |  |  |  |
| 2549 | Pulses, Other and products | | | 3010143 | | | Chickpea, grown inorganic, raw | | |  |  |  |
| 2549 | Pulses, Other and products | | | 3010146 | | | Chickpea, Kabuli, Giza 1, whole, dried | | |  |  |  |
| 2549 | Pulses, Other and products | | | 3010149 | | | Chickpea, Kabuli, Giza 2-L, whole, dried | | |  |  |  |
| 2549 | Pulses, Other and products | | | 3010152 | | | Chickpea, Kabuli, Giza 2-U, whole, dried | | |  |  |  |
| 2549 | Pulses, Other and products | | | 3010153 | | | Chickpea, Kabuli, seed, raw | | |  |  |  |
| 2549 | Pulses, Other and products | | | 3010162 | | | Chickpea, raw | | |  |  |  |
| 2549 | Pulses, Other and products | | | 3010163 | | | Chickpea, raw | | |  |  |  |
| 2549 | Pulses, Other and products | | | 3010164 | | | Chickpea, raw | | |  |  |  |
| 2549 | Pulses, Other and products | | | 3010165 | | | Chickpea, raw | | |  |  |  |
| 2549 | Pulses, Other and products | | | 3010166 | | | Chickpea, raw | | |  |  |  |
| 2549 | Pulses, Other and products | | | 3010182 | | | Chickpea, white, raw | | |  |  |  |
| 2549 | Pulses, Other and products | | | 3010183 | | | Chickpea, whole, raw | | |  |  |  |
| 2549 | Pulses, Other and products | | | 3010184 | | | Chickpea, whole, raw | | |  |  |  |
| 2549 | Pulses, Other and products | | | 3010365 | | | Cowpea, white, raw | | |  |  |  |
| 2549 | Pulses, Other and products | | | 3010367 | | | Faba bean, 1 irrigation/week, raw | | |  |  |  |
| 2549 | Pulses, Other and products | | | 3010368 | | | Faba bean, 2 irrigation/week, raw | | |  |  |  |
| 2549 | Pulses, Other and products | | | 3010370 | | | Faba bean, Aguadulce, whole seed, raw | | |  |  |  |
| 2549 | Pulses, Other and products | | | 3010373 | | | Faba bean, Big Qinpi, raw | | |  |  |  |
| 2549 | Pulses, Other and products | | | 3010377 | | | Faba bean, Giza 2, whole, raw | | |  |  |  |
| 2549 | Pulses, Other and products | | | 3010380 | | | Faba bean, Manfredini, whole seed, raw | | |  |  |  |
| 2549 | Pulses, Other and products | | | 3010382 | | | Faba bean, plant fertilized by 200 kg/ha P2O5 superphosphate and with 50 kg/ha sulphur, 1 irrigation/week, raw | | |  |  |  |
| 2549 | Pulses, Other and products | | | 3010383 | | | Faba bean, plant fertilized by 200 kg/ha P2O5 superphosphate and with 50 kg/ha sulphur, 2 irrigation/week, raw | | |  |  |  |
| 2549 | Pulses, Other and products | | | 3010384 | | | Faba bean, plant fertilized by 200 kg/ha P2O5 superphosphate, 1 irrigation/week, raw | | |  |  |  |
| 2549 | Pulses, Other and products | | | 3010385 | | | Faba bean, plant fertilized by 200 kg/ha P2O5 superphosphate, 2 irrigation/week, raw | | |  |  |  |
| 2549 | Pulses, Other and products | | | 3010386 | | | Faba bean, plant fertilized with 50 kg/ha sulphur, 1 irrigation/week, raw | | |  |  |  |
| 2549 | Pulses, Other and products | | | 3010387 | | | Faba bean, plant fertilized with 50 kg/ha sulphur, 2 irrigation/week, raw | | |  |  |  |
| 2549 | Pulses, Other and products | | | 3010388 | | | Faba bean, plant inoculated with mycorrhiza and fertilized by 200 kg/ha P2O5 superphosphate and with 50 kg/ha sulphur, 1 irrigation/week, raw | | |  |  |  |
| 2549 | Pulses, Other and products | | | 3010389 | | | Faba bean, plant inoculated with mycorrhiza and fertilized by 200 kg/ha P2O5 superphosphate and with 50 kg/ha sulphur, 2 irrigation/week, raw | | |  |  |  |
| 2549 | Pulses, Other and products | | | 3010390 | | | Faba bean, plant inoculated with mycorrhiza and fertilized by 200 kg/ha P2O5 superphosphate, 1 irrigation/week, raw | | |  |  |  |
| 2549 | Pulses, Other and products | | | 3010391 | | | Faba bean, plant inoculated with mycorrhiza and fertilized by 200 kg/ha P2O5 superphosphate, 2 irrigation/week, raw | | |  |  |  |
| 2549 | Pulses, Other and products | | | 3010392 | | | Faba bean, plant inoculated with mycorrhiza and fertilized with 50 kg/ha sulphur, 1 irrigation/week, raw | | |  |  |  |
| 2549 | Pulses, Other and products | | | 3010393 | | | Faba bean, plant inoculated with mycorrhiza and fertilized with 50 kg/ha sulphur, 2 irrigation/week, raw | | |  |  |  |
| 2549 | Pulses, Other and products | | | 3010394 | | | Faba bean, plant inoculated with mycorrhiza, 1 irrigation/week, raw | | |  |  |  |
| 2549 | Pulses, Other and products | | | 3010395 | | | Faba bean, plant inoculated with mycorrhiza, 2 irrigation/week, raw | | |  |  |  |
| 2549 | Pulses, Other and products | | | 3010396 | | | Faba bean, Polo, whole seed, raw | | |  |  |  |
| 2549 | Pulses, Other and products | | | 3010416 | | | Faba bean, raw | | |  |  |  |
| 2549 | Pulses, Other and products | | | 3010417 | | | Faba bean, raw | | |  |  |  |
| 2549 | Pulses, Other and products | | | 3010421 | | | Faba bean, Vesuvio, whole seed, raw | | |  |  |  |
| 2549 | Pulses, Other and products | | | 3010436 | | | Feathertree bean, raw | | |  |  |  |
| 2549 | Pulses, Other and products | | | 3010458 | | | Horse gram, whole, raw | | |  |  |  |
| 2549 | Pulses, Other and products | | | 3010463 | | | Itching bean, var. pruriens, Aliyar, raw | | |  |  |  |
| 2549 | Pulses, Other and products | | | 3010464 | | | Itching bean, var. pruriens, Anaikatti, raw | | |  |  |  |
| 2549 | Pulses, Other and products | | | 3010465 | | | Itching bean, var. pruriens, Ayyanarkoil, raw | | |  |  |  |
| 2549 | Pulses, Other and products | | | 3010466 | | | Itching bean, var. pruriens, Seithur, raw | | |  |  |  |
| 2549 | Pulses, Other and products | | | 3010467 | | | Itching bean, var. pruriens, Sivagiri, raw | | |  |  |  |
| 2549 | Pulses, Other and products | | | 3010468 | | | Jackbean, raw | | |  |  |  |
| 2549 | Pulses, Other and products | | | 3010470 | | | Lablab bean, raw | | |  |  |  |
| 2549 | Pulses, Other and products | | | 3010472 | | | Lentil, raw, 10kGy irradiated | | |  |  |  |
| 2549 | Pulses, Other and products | | | 3010473 | | | Lentil, raw, 5kGy irradiated | | |  |  |  |
| 2549 | Pulses, Other and products | | | 3010474 | | | Lentil, raw, 7.5kGy irradiated | | |  |  |  |
| 2549 | Pulses, Other and products | | | 3010476 | | | Lentil, Brown, raw | | |  |  |  |
| 2549 | Pulses, Other and products | | | 3010477 | | | Lentil, Brown, seed, raw | | |  |  |  |
| 2549 | Pulses, Other and products | | | 3010483 | | | Lentil, dal | | |  |  |  |
| 2549 | Pulses, Other and products | | | 3010484 | | | Lentil, Pardina, seed, raw | | |  |  |  |
| 2549 | Pulses, Other and products | | | 3010488 | | | Lentil, raw | | |  |  |  |
| 2549 | Pulses, Other and products | | | 3010489 | | | Lentil, raw | | |  |  |  |
| 2549 | Pulses, Other and products | | | 3010490 | | | Lentil, raw | | |  |  |  |
| 2549 | Pulses, Other and products | | | 3010491 | | | Lentil, raw | | |  |  |  |
| 2549 | Pulses, Other and products | | | 3010492 | | | Lentil, raw | | |  |  |  |
| 2549 | Pulses, Other and products | | | 3010493 | | | Lentil, raw | | |  |  |  |
| 2549 | Pulses, Other and products | | | 3010494 | | | Lentil, raw | | |  |  |  |
| 2549 | Pulses, Other and products | | | 3010498 | | | Lentil, whole grain, sun-dried | | |  |  |  |
| 2549 | Pulses, Other and products | | | 3010499 | | | Lentil, whole, brown, raw | | |  |  |  |
| 2549 | Pulses, Other and products | | | 3010500 | | | Lentil, whole, yellowish, raw | | |  |  |  |
| 2549 | Pulses, Other and products | | | 3010518 | | | Locust bean, seed, raw | | |  |  |  |
| 2549 | Pulses, Other and products | | | 3010520 | | | Longleaf Milkpea, dried | | |  |  |  |
| 2549 | Pulses, Other and products | | | 3010536 | | | Mexican palo verde bean, raw | | |  |  |  |
| 2549 | Pulses, Other and products | | | 3010537 | | | Mexican palo verde bean, raw | | |  |  |  |
| 2549 | Pulses, Other and products | | | 3010538 | | | Mexican palo verde bean, raw | | |  |  |  |
| 2549 | Pulses, Other and products | | | 3010546 | | | Mucuna, Mottle, seed, mature, air-dried, raw | | |  |  |  |
| 2549 | Pulses, Other and products | | | 3010547 | | | Mucuna, seed, mature, air-dried, raw | | |  |  |  |
| 2549 | Pulses, Other and products | | | 3010548 | | | Mucuna, seed, mature, air-dried, raw | | |  |  |  |
| 2549 | Pulses, Other and products | | | 3010549 | | | Mucuna, seed, mature, air-dried, raw | | |  |  |  |
| 2549 | Pulses, Other and products | | | 3010550 | | | Mucuna, seed, mature, air-dried, raw | | |  |  |  |
| 2549 | Pulses, Other and products | | | 3010551 | | | Mucuna, var. utilis, seed, mature, sun-dried, raw | | |  |  |  |
| 2549 | Pulses, Other and products | | | 3010553 | | | Mucuna, White, seed, mature, air-dried, raw | | |  |  |  |
| 2549 | Pulses, Other and products | | | 3010627 | | | Pigeon pea, raw | | |  |  |  |
| 2549 | Pulses, Other and products | | | 3010628 | | | Pigeon pea, raw | | |  |  |  |
| 2549 | Pulses, Other and products | | | 3010635 | | | Red gram, dal | | |  |  |  |
| 2549 | Pulses, Other and products | | | 3010636 | | | Red gram, raw | | |  |  |  |
| 2549 | Pulses, Other and products | | | 3010638 | | | Red gram, whole, raw | | |  |  |  |
| 2549 | Pulses, Other and products | | | 3010664 | | | Sesbania, raw | | |  |  |  |
| 2549 | Pulses, Other and products | | | 3010665 | | | Sesbania, raw | | |  |  |  |
| 2549 | Pulses, Other and products | | | 3010666 | | | Sesbania, raw | | |  |  |  |
| 2549 | Pulses, Other and products | | | 3010684 | | | Stink bean, dry heated | | |  |  |  |
| 2549 | Pulses, Other and products | | | 3010688 | | | Stink bean, raw | | |  |  |  |
| 2549 | Pulses, Other and products | | | 3010698 | | | Velvet bean, black coloured seed coat, raw | | |  |  |  |
| 2549 | Pulses, Other and products | | | 3010700 | | | Velvet bean, Black, whole seed, raw | | |  |  |  |
| 2549 | Pulses, Other and products | | | 3010701 | | | Velvet bean, Cream, seed, sun-dried | | |  |  |  |
| 2549 | Pulses, Other and products | | | 3010702 | | | Velvet bean, Maroon, seed, sun-dried | | |  |  |  |
| 2549 | Pulses, Other and products | | | 3010703 | | | Velvet bean, Mottled, seed, sun-dried | | |  |  |  |
| 2549 | Pulses, Other and products | | | 3010704 | | | Velvet bean, Mottled, seed, sun-dried | | |  |  |  |
| 2549 | Pulses, Other and products | | | 3010705 | | | Velvet bean, seed, mature, sun-dried | | |  |  |  |
| 2549 | Pulses, Other and products | | | 3010706 | | | Velvet bean, seed, sun-dried | | |  |  |  |
| 2549 | Pulses, Other and products | | | 3010707 | | | Velvet bean, seed, sun-dried | | |  |  |  |
| 2549 | Pulses, Other and products | | | 3010708 | | | Velvet bean, underutilized type, raw | | |  |  |  |
| 2549 | Pulses, Other and products | | | 3010709 | | | Velvet bean, white coloured seed coat, raw | | |  |  |  |
| 2549 | Pulses, Other and products | | | 3010712 | | | Velvet bean, White, whole seed, raw | | |  |  |  |
| 2549 | Pulses, Other and products | | | 3010713 | | | Velvet bean, White, whole seed, raw | | |  |  |  |
| 2549 | Pulses, Other and products | | | 3010714 | | | Winged bean, 034(b), raw | | |  |  |  |
| 2549 | Pulses, Other and products | | | 3010715 | | | Winged bean, 039(a)a, raw | | |  |  |  |
| 2549 | Pulses, Other and products | | | 3010716 | | | Winged bean, 042(a), raw | | |  |  |  |
| 2549 | Pulses, Other and products | | | 3010717 | | | Winged bean, 044(a), raw | | |  |  |  |
| 2549 | Pulses, Other and products | | | 3010718 | | | Winged bean, 046(b)/ba, raw | | |  |  |  |
| 2549 | Pulses, Other and products | | | 3010719 | | | Winged bean, 048(a), raw | | |  |  |  |
| 2549 | Pulses, Other and products | | | 3010720 | | | Winged bean, 050(a), raw | | |  |  |  |
| 2549 | Pulses, Other and products | | | 3010721 | | | Winged bean, 051(b), raw | | |  |  |  |
| 2549 | Pulses, Other and products | | | 3010722 | | | Winged bean, 079(c)/b(a), raw | | |  |  |  |
| 2549 | Pulses, Other and products | | | 3010723 | | | Winged bean, 157(d), raw | | |  |  |  |
| 2549 | Pulses, Other and products | | | 3010724 | | | Winged bean, 181(b), raw | | |  |  |  |
| 2549 | Pulses, Other and products | | | 3010725 | | | Winged bean, 184(a)(b), raw | | |  |  |  |
| 2549 | Pulses, Other and products | | | 3010726 | | | Winged bean, 188(d), raw | | |  |  |  |
| 2549 | Pulses, Other and products | | | 3010727 | | | Winged bean, 195(b), raw | | |  |  |  |
| 2549 | Pulses, Other and products | | | 3010728 | | | Winged bean, 201(a), raw | | |  |  |  |
| 2549 | Pulses, Other and products | | | 3010729 | | | Winged bean, 207, raw | | |  |  |  |
| 2549 | Pulses, Other and products | | | 4030054 | | | Grass pea, fresh, raw | | |  |  |  |
| 2549 | Pulses, Other and products | | | 4030055 | | | Grass pea, fresh, raw | | |  |  |  |
| 2549 | Pulses, Other and products | | | 4030062 | | | Grass pea, Kwestor, raw | | |  |  |  |
| 2551 | Nuts and products | | | 6010016 | | | Almond, raw | | |  |  |  |
| 2551 | Nuts and products | | | 6010019 | | | Arecanut, brown, dried | | |  |  |  |
| 2551 | Nuts and products | | | 6010020 | | | Arecanut, fresh, raw | | |  |  |  |
| 2551 | Nuts and products | | | 6010021 | | | Arecanut, red, dried | | |  |  |  |
| 2551 | Nuts and products | | | 6010032 | | | Brazil nut, raw | | |  |  |  |
| 2551 | Nuts and products | | | 6010036 | | | Cashew nut, raw | | |  |  |  |
| 2551 | Nuts and products | | | 6010037 | | | Cashew nut, raw | | |  |  |  |
| 2551 | Nuts and products | | | 6010038 | | | Cashew nut, raw | | |  |  |  |
| 2551 | Nuts and products | | | 6010079 | | | Hazelnut, raw | | |  |  |  |
| 2551 | Nuts and products | | | 6010098 | | | Macadamia nut, raw | | |  |  |  |
| 2551 | Nuts and products | | | 6010099 | | | Macadamia nuts, raw | | |  |  |  |
| 2551 | Nuts and products | | | 6010146 | | | Pecan nut, Desirable, raw | | |  |  |  |
| 2551 | Nuts and products | | | 6010150 | | | Pine nut, raw | | |  |  |  |
| 2551 | Nuts and products | | | 6010151 | | | Pine nut, raw | | |  |  |  |
| 2551 | Nuts and products | | | 6010154 | | | Pistachio, raw | | |  |  |  |
| 2551 | Nuts and products | | | 6010182 | | | Walnut, raw | | |  |  |  |
| 2551 | Nuts and products | | | 6010183 | | | Walnut, raw | | |  |  |  |
| 2551 | Nuts and products | | | 6010201 | | | Wonderful kola, seed, raw | | |  |  |  |
| 2555 | Soyabeans | | | 3020034 | | | Soybean, Asmara, mature seed (6 weeks), raw | | |  |  |  |
| 2555 | Soyabeans | | | 3020035 | | | Soybean, brown, raw | | |  |  |  |
| 2555 | Soyabeans | | | 3020036 | | | Soybean, C & W brand | | |  |  |  |
| 2555 | Soyabeans | | | 3020038 | | | Soybean, Crawford, whole, raw | | |  |  |  |
| 2555 | Soyabeans | | | 3020043 | | | Soybean, Daewon, dried | | |  |  |  |
| 2555 | Soyabeans | | | 3020044 | | | Soybean, Dekabig, raw | | |  |  |  |
| 2555 | Soyabeans | | | 3020045 | | | Soybean, dried | | |  |  |  |
| 2555 | Soyabeans | | | 3020046 | | | Soybean, dried | | |  |  |  |
| 2555 | Soyabeans | | | 3020047 | | | Soybean, dried | | |  |  |  |
| 2555 | Soyabeans | | | 3020050 | | | Soybean, Giza 82, raw | | |  |  |  |
| 2555 | Soyabeans | | | 3020051 | | | Soybean, Glyphosate-Tolerant Soybean 40-3-2, raw | | |  |  |  |
| 2555 | Soyabeans | | | 3020052 | | | Soybean, Jinpum n.2, dried | | |  |  |  |
| 2555 | Soyabeans | | | 3020057 | | | Soybean, Mooncake, mature seed (6 weeks), raw | | |  |  |  |
| 2555 | Soyabeans | | | 3020059 | | | Soybean, prennial, dried | | |  |  |  |
| 2555 | Soyabeans | | | 3020060 | | | Soybean, Puleun, dried | | |  |  |  |
| 2555 | Soyabeans | | | 3020061 | | | Soybean, raw | | |  |  |  |
| 2555 | Soyabeans | | | 3020062 | | | Soybean, raw | | |  |  |  |
| 2555 | Soyabeans | | | 3020063 | | | Soybean, raw | | |  |  |  |
| 2555 | Soyabeans | | | 3020064 | | | Soybean, Red Mill brand, raw | | |  |  |  |
| 2555 | Soyabeans | | | 3020065 | | | Soybean, Safeway brand, sweet | | |  |  |  |
| 2555 | Soyabeans | | | 3020066 | | | Soybean, Seonheuk, dried | | |  |  |  |
| 2555 | Soyabeans | | | 3020068 | | | Soybean, Sinpaldal n.2, dried | | |  |  |  |
| 2555 | Soyabeans | | | 3020069 | | | Soybean, Somyeong, dried | | |  |  |  |
| 2555 | Soyabeans | | | 3020070 | | | Soybean, Taewang, dried | | |  |  |  |
| 2555 | Soyabeans | | | 3020071 | | | Soybean, Taiwan 75-M, dried, 150Gy irradiated | | |  |  |  |
| 2555 | Soyabeans | | | 3020072 | | | Soybean, Taiwan 75-M, dried, 150Gy irradiated | | |  |  |  |
| 2555 | Soyabeans | | | 3020073 | | | Soybean, Taiwan 75-P, dried, 150Gy irradiated | | |  |  |  |
| 2555 | Soyabeans | | | 3020074 | | | Soybean, Taiwan 75-P, dried, 150Gy irradiated | | |  |  |  |
| 2555 | Soyabeans | | | 3020077 | | | Soybean, white, raw | | |  |  |  |
| 2555 | Soyabeans | | | 3020079 | | | Soybean, ZC3-M, dried, 150Gy irradiated | | |  |  |  |
| 2555 | Soyabeans | | | 3020080 | | | Soybean, ZC3-M, dried, 150Gy irradiated | | |  |  |  |
| 2555 | Soyabeans | | | 3020081 | | | Soybean, ZC3-P, dried, 150Gy irradiated | | |  |  |  |
| 2555 | Soyabeans | | | 3020082 | | | Soybean, ZC3-P, dried, 150Gy irradiated | | |  |  |  |
| 2555 | Soyabeans | | | 3020083 | | | Soybeans, Bragg, 112kg of P2O5/ha, raw | | |  |  |  |
| 2555 | Soyabeans | | | 3020084 | | | Soybeans, Bragg, 56kg of P2O5/ha, raw | | |  |  |  |
| 2555 | Soyabeans | | | 3020085 | | | Soybeans, Bragg, raw | | |  |  |  |
| 2555 | Soyabeans | | | 3020086 | | | Soybeans, Punja-1, 112kg of P2O5/ha, raw | | |  |  |  |
| 2555 | Soyabeans | | | 3020087 | | | Soybeans, Punja-1, 56kg of P2O5/ha, raw | | |  |  |  |
| 2555 | Soyabeans | | | 3020088 | | | Soybeans, Punja-1, raw | | |  |  |  |
| 2556 | Groundnuts (Shelled Eq) | | | 6010069 | | | Ground nut, raw | | |  |  |  |
| 2556 | Groundnuts (Shelled Eq) | | | 6010073 | | | Groundnut, fresh, raw | | |  |  |  |
| 2556 | Groundnuts (Shelled Eq) | | | 6010074 | | | Groundnut, mature, raw | | |  |  |  |
| 2556 | Groundnuts (Shelled Eq) | | | 6010127 | | | Peanut, raw | | |  |  |  |
| 2556 | Groundnuts (Shelled Eq) | | | 6010139 | | | Peanut, raw | | |  |  |  |
| 2556 | Groundnuts (Shelled Eq) | | | 6010140 | | | Peanut, raw | | |  |  |  |
| 2556 | Groundnuts (Shelled Eq) | | | 6010141 | | | Peanut, raw | | |  |  |  |
| 2557 | Sunflower seed | | | 6010173 | | | Sunflower, seed, raw | | |  |  |  |
| 2558 | Rape and Mustardseed | | | 6010120 | | | Mustard, seed, raw | | |  |  |  |
| 2559 | Cottonseed | | | 6010049 | | | Cottonseed, seed flour, ISA BC4, dehulled, defatted | | |  |  |  |
| 2560 | Coconuts - Incl Copra | | | 5020021 | | | Coconut, kernel, fresh, raw | | |  |  |  |
| 2561 | Sesame seed | | | 6010161 | | | Sesame seed, grown inorganic, raw | | |  |  |  |
| 2561 | Sesame seed | | | 6010162 | | | Sesame seed, HT-1, grown organic, raw | | |  |  |  |
| 2561 | Sesame seed | | | 6010163 | | | Sesame seed, raw | | |  |  |  |
| 2561 | Sesame seed | | | 6010164 | | | Sesame seed, whole, raw | | |  |  |  |
| 2562 | Palm kernels | | | Wessels et al (2012) | | | Palm kernels | | |  |  |  |
| 2570 | Oilcrops, Other | | | 6010089 | | | Linseed, raw | | |  |  |  |
| 2570 | Oilcrops, Other | | | 6010155 | | | Poppy seed, McCormick | | |  |  |  |
| 2570 | Oilcrops, Other | | | 6010160 | | | Safflower, seed, raw | | |  |  |  |
| 2572 | Groundnut Oil | | | 12010003 | | | Groundnut oil | | |  |  |  |
| 2577 | Palm Oil | | | 12020001 | | | Palm oil, orange | | |  |  |  |
| 2601 | Tomatoes and products | | | 4030107 | | | Tomato, bitter, raw | | |  |  |  |
| 2601 | Tomatoes and products | | | 4030108 | | | Tomato, green, raw | | |  |  |  |
| 2601 | Tomatoes and products | | | 4030109 | | | Tomato, raw | | |  |  |  |
| 2601 | Tomatoes and products | | | 4030110 | | | Tomato, ripe, hybrid, raw | | |  |  |  |
| 2601 | Tomatoes and products | | | 4030111 | | | Tomato, ripe, raw | | |  |  |  |
| 2602 | Onions | | | 4030087 | | | Onion, big, raw | | |  |  |  |
| 2602 | Onions | | | 4030088 | | | Onion, raw | | |  |  |  |
| 2602 | Onions | | | 4030089 | | | Onion, raw | | |  |  |  |
| 2602 | Onions | | | 4030090 | | | Onion, small, raw | | |  |  |  |
| 2605 | Vegetables, Other | | | 2050008 | | | Beet root, raw | | |  |  |  |
| 2605 | Vegetables, Other | | | 2050009 | | | Beet, red beet, raw | | |  |  |  |
| 2605 | Vegetables, Other | | | 2050014 | | | Fluted pumpkin root, mature plant, raw | | |  |  |  |
| 2605 | Vegetables, Other | | | 2050021 | | | Parsnip, raw | | |  |  |  |
| 2605 | Vegetables, Other | | | 2050022 | | | Radish, raw | | |  |  |  |
| 2605 | Vegetables, Other | | | 2050029 | | | Turnip, raw | | |  |  |  |
| 2605 | Vegetables, Other | | | 2050030 | | | Turnip, raw | | |  |  |  |
| 2605 | Vegetables, Other | | | 4010002 | | | African lettuce, leaves, raw | | |  |  |  |
| 2605 | Vegetables, Other | | | 4010006 | | | Amaranth globe, ABS-38-AWKA, dried | | |  |  |  |
| 2605 | Vegetables, Other | | | 4010008 | | | Amaranth globe, AKS-33-EKPENE EDIENE x ABS-38-AWKA, dried | | |  |  |  |
| 2605 | Vegetables, Other | | | 4010010 | | | Amaranth globe, AKS-33-EKPENE EDIENE, dried | | |  |  |  |
| 2605 | Vegetables, Other | | | 4010012 | | | Amaranth globe, EBS-15-NKALAGU x ABS-38-AKWA, dried | | |  |  |  |
| 2605 | Vegetables, Other | | | 4010014 | | | Amaranth globe, EBS-15-NKALAGU x AKS-33-EKPENE EDIENE, dried | | |  |  |  |
| 2605 | Vegetables, Other | | | 4010016 | | | Amaranth globe, EBS-15-NKALAGU x IMS-20-NJIABA, dried | | |  |  |  |
| 2605 | Vegetables, Other | | | 4010018 | | | Amaranth globe, EBS-15-NKALAGU, dried | | |  |  |  |
| 2605 | Vegetables, Other | | | 4010020 | | | Amaranth globe, ENS-08-MBU x ABS-38-AWKA, dried | | |  |  |  |
| 2605 | Vegetables, Other | | | 4010022 | | | Amaranth globe, ENS-08-MBU x AKS-33-EKPENE EDIENE, dried | | |  |  |  |
| 2605 | Vegetables, Other | | | 4010024 | | | Amaranth globe, ENS-08-MBU x EBS-15-NKALAGU, dried | | |  |  |  |
| 2605 | Vegetables, Other | | | 4010026 | | | Amaranth globe, ENS-08-MBU x IMS-20-NJIABA, dried | | |  |  |  |
| 2605 | Vegetables, Other | | | 4010028 | | | Amaranth globe, ENS-08-MBU, dried | | |  |  |  |
| 2605 | Vegetables, Other | | | 4010030 | | | Amaranth globe, IMS-20-NJIABA x ABS-38-AWKA, dried | | |  |  |  |
| 2605 | Vegetables, Other | | | 4010032 | | | Amaranth globe, IMS-20-NJIABA x AKS-33-EKPENE EDIENE, dried | | |  |  |  |
| 2605 | Vegetables, Other | | | 4010034 | | | Amaranth globe, IMS-20-NJIABA, dried | | |  |  |  |
| 2605 | Vegetables, Other | | | 4010055 | | | Beet greens, raw | | |  |  |  |
| 2605 | Vegetables, Other | | | 4010078 | | | Brussels sprouts, raw | | |  |  |  |
| 2605 | Vegetables, Other | | | 4010080 | | | Buffalo spinach, leaves, raw | | |  |  |  |
| 2605 | Vegetables, Other | | | 4010086 | | | Cabbage, Chinese, raw | | |  |  |  |
| 2605 | Vegetables, Other | | | 4010088 | | | Cabbage, collard greens, raw | | |  |  |  |
| 2605 | Vegetables, Other | | | 4010089 | | | Cabbage, green, raw | | |  |  |  |
| 2605 | Vegetables, Other | | | 4010093 | | | Cabbage, raw | | |  |  |  |
| 2605 | Vegetables, Other | | | 4010094 | | | Cabbage, violet, raw | | |  |  |  |
| 2605 | Vegetables, Other | | | 4010099 | | | Cassava, leaves, destalked, raw | | |  |  |  |
| 2605 | Vegetables, Other | | | 4010100 | | | Cassava, leaves, raw | | |  |  |  |
| 2605 | Vegetables, Other | | | 4010101 | | | Cassava, leaves, raw | | |  |  |  |
| 2605 | Vegetables, Other | | | 4010102 | | | Cassava, leaves, raw | | |  |  |  |
| 2605 | Vegetables, Other | | | 4010103 | | | Cassava, mature leaves, raw | | |  |  |  |
| 2605 | Vegetables, Other | | | 4010104 | | | Cassava, very young leaves, raw | | |  |  |  |
| 2605 | Vegetables, Other | | | 4010105 | | | Cassava, young leaves, raw | | |  |  |  |
| 2605 | Vegetables, Other | | | 4010108 | | | Cauliflower, raw | | |  |  |  |
| 2605 | Vegetables, Other | | | 4010109 | | | Cauliflower, raw | | |  |  |  |
| 2605 | Vegetables, Other | | | 4010113 | | | Chinese violet, leaves, raw | | |  |  |  |
| 2605 | Vegetables, Other | | | 4010123 | | | Collard, raw | | |  |  |  |
| 2605 | Vegetables, Other | | | 4010126 | | | Common nettle, leaves, wild, raw | | |  |  |  |
| 2605 | Vegetables, Other | | | 4010148 | | | Garden cress, raw | | |  |  |  |
| 2605 | Vegetables, Other | | | 4010170 | | | Lettuce, raw | | |  |  |  |
| 2605 | Vegetables, Other | | | 4010178 | | | Mustard, leaves, raw | | |  |  |  |
| 2605 | Vegetables, Other | | | 4010179 | | | Mustard, leaves, raw | | |  |  |  |
| 2605 | Vegetables, Other | | | 4010196 | | | Pak Choi, leaves, raw | | |  |  |  |
| 2605 | Vegetables, Other | | | 4010227 | | | Sorrell, leaves, raw | | |  |  |  |
| 2605 | Vegetables, Other | | | 4010229 | | | Spinach, raw | | |  |  |  |
| 2605 | Vegetables, Other | | | 4010248 | | | Water cress, leaves, raw | | |  |  |  |
| 2605 | Vegetables, Other | | | 4010254 | | | Waterleaf leaves, raw | | |  |  |  |
| 2605 | Vegetables, Other | | | 4010255 | | | Waterleaf leaves, wild, raw | | |  |  |  |
| 2605 | Vegetables, Other | | | 4010258 | | | Yellow sawah lettuce, leaves, raw | | |  |  |  |
| 2605 | Vegetables, Other | | | 4020001 | | | Babycorn, raw | | |  |  |  |
| 2605 | Vegetables, Other | | | 4020002 | | | Carrot, orange, raw | | |  |  |  |
| 2605 | Vegetables, Other | | | 4020003 | | | Carrot, raw | | |  |  |  |
| 2605 | Vegetables, Other | | | 4020004 | | | Carrot, raw | | |  |  |  |
| 2605 | Vegetables, Other | | | 4020005 | | | Carrot, red, raw | | |  |  |  |
| 2605 | Vegetables, Other | | | 4020006 | | | Cucumber, orange, round, raw | | |  |  |  |
| 2605 | Vegetables, Other | | | 4020007 | | | Pumpkin, orange, round, raw | | |  |  |  |
| 2605 | Vegetables, Other | | | 4020008 | | | Sweet pepper, yellow, raw | | |  |  |  |
| 2605 | Vegetables, Other | | | 4020009 | | | Zucchini, yellow, raw | | |  |  |  |
| 2605 | Vegetables, Other | | | 4030001 | | | Ash gourd, raw | | |  |  |  |
| 2605 | Vegetables, Other | | | 4030002 | | | Avocado fruit, raw | | |  |  |  |
| 2605 | Vegetables, Other | | | 4030003 | | | Bamboo shoot, tender, raw | | |  |  |  |
| 2605 | Vegetables, Other | | | 4030004 | | | Bitter gourd, jagged, smooth ridges, elongate, raw | | |  |  |  |
| 2605 | Vegetables, Other | | | 4030005 | | | Bitter gourd, jagged, teeth ridges, elongate, raw | | |  |  |  |
| 2605 | Vegetables, Other | | | 4030006 | | | Bitter gourd, jagged, teeth ridges, short, raw | | |  |  |  |
| 2605 | Vegetables, Other | | | 4030008 | | | Bottle gourd, elongate, dark green, raw | | |  |  |  |
| 2605 | Vegetables, Other | | | 4030009 | | | Bottle gourd, elongate, pale green, raw | | |  |  |  |
| 2605 | Vegetables, Other | | | 4030010 | | | Bottle gourd, raw | | |  |  |  |
| 2605 | Vegetables, Other | | | 4030011 | | | Bottle gourd, round, pale green, raw | | |  |  |  |
| 2605 | Vegetables, Other | | | 4030012 | | | Capsicum, green, raw | | |  |  |  |
| 2605 | Vegetables, Other | | | 4030013 | | | Capsicum, red, raw | | |  |  |  |
| 2605 | Vegetables, Other | | | 4030014 | | | Celery stalk, raw | | |  |  |  |
| 2605 | Vegetables, Other | | | 4030015 | | | Cho-cho-marrow, raw | | |  |  |  |
| 2605 | Vegetables, Other | | | 4030018 | | | Cowslip creeper, raw | | |  |  |  |
| 2605 | Vegetables, Other | | | 4030022 | | | Cucumber, bitter, raw | | |  |  |  |
| 2605 | Vegetables, Other | | | 4030024 | | | Cucumber, green, elongate, raw | | |  |  |  |
| 2605 | Vegetables, Other | | | 4030025 | | | Cucumber, green, short, raw | | |  |  |  |
| 2605 | Vegetables, Other | | | 4030027 | | | Eggplant, Brinjal-1, raw | | |  |  |  |
| 2605 | Vegetables, Other | | | 4030028 | | | Eggplant, Brinjal-10, raw | | |  |  |  |
| 2605 | Vegetables, Other | | | 4030029 | | | Eggplant, Brinjal-11, raw | | |  |  |  |
| 2605 | Vegetables, Other | | | 4030030 | | | Eggplant, Brinjal-12, raw | | |  |  |  |
| 2605 | Vegetables, Other | | | 4030031 | | | Eggplant, Brinjal-13, raw | | |  |  |  |
| 2605 | Vegetables, Other | | | 4030032 | | | Eggplant, Brinjal-14, raw | | |  |  |  |
| 2605 | Vegetables, Other | | | 4030033 | | | Eggplant, Brinjal-15, raw | | |  |  |  |
| 2605 | Vegetables, Other | | | 4030034 | | | Eggplant, Brinjal-16, raw | | |  |  |  |
| 2605 | Vegetables, Other | | | 4030035 | | | Eggplant, Brinjal-17, raw | | |  |  |  |
| 2605 | Vegetables, Other | | | 4030036 | | | Eggplant, Brinjal-18, raw | | |  |  |  |
| 2605 | Vegetables, Other | | | 4030037 | | | Eggplant, Brinjal-19, raw | | |  |  |  |
| 2605 | Vegetables, Other | | | 4030038 | | | Eggplant, Brinjal-2, raw | | |  |  |  |
| 2605 | Vegetables, Other | | | 4030039 | | | Eggplant, Brinjal-20, raw | | |  |  |  |
| 2605 | Vegetables, Other | | | 4030040 | | | Eggplant, Brinjal-21, raw | | |  |  |  |
| 2605 | Vegetables, Other | | | 4030041 | | | Eggplant, Brinjal-3, raw | | |  |  |  |
| 2605 | Vegetables, Other | | | 4030042 | | | Eggplant, Brinjal-4, raw | | |  |  |  |
| 2605 | Vegetables, Other | | | 4030043 | | | Eggplant, Brinjal-5, raw | | |  |  |  |
| 2605 | Vegetables, Other | | | 4030044 | | | Eggplant, Brinjal-6, raw | | |  |  |  |
| 2605 | Vegetables, Other | | | 4030045 | | | Eggplant, Brinjal-7, raw | | |  |  |  |
| 2605 | Vegetables, Other | | | 4030046 | | | Eggplant, Brinjal-8, raw | | |  |  |  |
| 2605 | Vegetables, Other | | | 4030047 | | | Eggplant, Brinjal-9, raw | | |  |  |  |
| 2605 | Vegetables, Other | | | 4030048 | | | Eggplant, raw | | |  |  |  |
| 2605 | Vegetables, Other | | | 4030063 | | | Kovai, big, raw | | |  |  |  |
| 2605 | Vegetables, Other | | | 4030064 | | | Kovai, small, raw | | |  |  |  |
| 2605 | Vegetables, Other | | | 4030065 | | | Mushroom, 'Agaricus bisporus', raw | | |  |  |  |
| 2605 | Vegetables, Other | | | 4030066 | | | Mushroom, 'Auricularia auricula', dried | | |  |  |  |
| 2605 | Vegetables, Other | | | 4030067 | | | Mushroom, 'Calvatia cyathiformis', dried | | |  |  |  |
| 2605 | Vegetables, Other | | | 4030068 | | | Mushroom, 'Hirneola auriculajudae', fresh, raw | | |  |  |  |
| 2605 | Vegetables, Other | | | 4030069 | | | Mushroom, 'Lentinus brunneofloccosus', fresh, raw | | |  |  |  |
| 2605 | Vegetables, Other | | | 4030070 | | | Mushroom, 'Lentinus subnudud', dried | | |  |  |  |
| 2605 | Vegetables, Other | | | 4030071 | | | Mushroom, 'Pleurotus ostreatus', fresh, raw | | |  |  |  |
| 2605 | Vegetables, Other | | | 4030072 | | | Mushroom, 'Pleurotus sajor caju', raw | | |  |  |  |
| 2605 | Vegetables, Other | | | 4030073 | | | Mushroom, 'Psathyrella atroumbonata', dried | | |  |  |  |
| 2605 | Vegetables, Other | | | 4030074 | | | Mushroom, 'Psathyrella tuberculata', fresh, raw | | |  |  |  |
| 2605 | Vegetables, Other | | | 4030075 | | | Mushroom, 'Schizophyllum commune', dried | | |  |  |  |
| 2605 | Vegetables, Other | | | 4030076 | | | Mushroom, 'Termitomyces letestui', fresh, raw | | |  |  |  |
| 2605 | Vegetables, Other | | | 4030077 | | | Mushroom, 'Termitomyces microcarpus', dried | | |  |  |  |
| 2605 | Vegetables, Other | | | 4030078 | | | Mushroom, 'Termitomyces robustus', dried | | |  |  |  |
| 2605 | Vegetables, Other | | | 4030079 | | | Mushroom, 'Volvariella volvacea', fresh, raw | | |  |  |  |
| 2605 | Vegetables, Other | | | 4030081 | | | Okra, fruit, washed, dried | | |  |  |  |
| 2605 | Vegetables, Other | | | 4030084 | | | Okra, raw | | |  |  |  |
| 2605 | Vegetables, Other | | | 4030085 | | | Okra, raw | | |  |  |  |
| 2605 | Vegetables, Other | | | 4030086 | | | Okra, raw | | |  |  |  |
| 2605 | Vegetables, Other | | | 4030092 | | | Parwar, raw | | |  |  |  |
| 2605 | Vegetables, Other | | | 4030094 | | | Pumpkin, green, cylindrical, raw | | |  |  |  |
| 2605 | Vegetables, Other | | | 4030095 | | | Radish, elongate, red skin, raw | | |  |  |  |
| 2605 | Vegetables, Other | | | 4030096 | | | Radish, elongate, white skin, raw | | |  |  |  |
| 2605 | Vegetables, Other | | | 4030097 | | | Radish, round, red skin, raw | | |  |  |  |
| 2605 | Vegetables, Other | | | 4030098 | | | Radish, round, white skin, raw | | |  |  |  |
| 2605 | Vegetables, Other | | | 4030099 | | | Ridge gourd, raw | | |  |  |  |
| 2605 | Vegetables, Other | | | 4030100 | | | Ridge gourd, smooth skin, raw | | |  |  |  |
| 2605 | Vegetables, Other | | | 4030101 | | | Snake gourd, long, dark green, raw | | |  |  |  |
| 2605 | Vegetables, Other | | | 4030102 | | | Snake gourd, long, pale green, raw | | |  |  |  |
| 2605 | Vegetables, Other | | | 4030103 | | | Snake gourd, short, raw | | |  |  |  |
| 2605 | Vegetables, Other | | | 4030106 | | | Tinda, tender, raw | | |  |  |  |
| 2605 | Vegetables, Other | | | 4030112 | | | Zucchini, green, raw | | |  |  |  |
| 2605 | Vegetables, Other | | | 15010021 | | | Chili pepper, Green, raw | | |  |  |  |
| 2605 | Vegetables, Other | | | 15010022 | | | Chili pepper, Green-1, raw | | |  |  |  |
| 2605 | Vegetables, Other | | | 15010023 | | | Chili pepper, Green-2, raw | | |  |  |  |
| 2605 | Vegetables, Other | | | 15010024 | | | Chili pepper, Green-3, raw | | |  |  |  |
| 2605 | Vegetables, Other | | | 15010025 | | | Chili pepper, Green-4, raw | | |  |  |  |
| 2605 | Vegetables, Other | | | 15010026 | | | Chili pepper, Green-5, raw | | |  |  |  |
| 2605 | Vegetables, Other | | | 15010027 | | | Chili pepper, Green-6, raw | | |  |  |  |
| 2605 | Vegetables, Other | | | 15010028 | | | Chili pepper, Green-7, raw | | |  |  |  |
| 2605 | Vegetables, Other | | | 15010029 | | | Chili pepper, raw | | |  |  |  |
| 2605 | Vegetables, Other | | | 15010055 | | | Garlic, big clove, raw | | |  |  |  |
| 2605 | Vegetables, Other | | | 15010058 | | | Garlic, Kashmir, single clove, raw | | |  |  |  |
| 2605 | Vegetables, Other | | | 15010059 | | | Garlic, small clove, raw | | |  |  |  |
| 2605 | Vegetables, Other | | | 15010075 | | | Parsley, raw | | |  |  |  |
| 2611 | Oranges, Mandarines | | | 5010030 | | | Orange, raw | | |  |  |  |
| 2614 | Citrus, Other | | | 5010012 | | | Citron, fresh whole fruit, raw | | |  |  |  |
| 2615 | Bananas | | | 5010006 | | | Banana, Montham, ripe, raw | | |  |  |  |
| 2615 | Bananas | | | 5010007 | | | Banana, Poovam, ripe, raw | | |  |  |  |
| 2615 | Bananas | | | 5010008 | | | Banana, raw | | |  |  |  |
| 2615 | Bananas | | | 5010010 | | | Banana, red, ripe, raw | | |  |  |  |
| 2615 | Bananas | | | 5010011 | | | Banana, Robusta, ripe, raw | | |  |  |  |
| 2616 | Plantains | | | 2060010 | | | Plantain, green, raw | | |  |  |  |
| 2617 | Apples and products | | | 5020004 | | | Apple, big, raw | | |  |  |  |
| 2617 | Apples and products | | | 5020005 | | | Apple, green, raw | | |  |  |  |
| 2617 | Apples and products | | | 5020006 | | | Apple, Kashmir, small, raw | | |  |  |  |
| 2617 | Apples and products | | | 5020007 | | | Apple, small, raw | | |  |  |  |
| 2618 | Pineapples and products | | | 5010042 | | | Pineapple, raw | | |  |  |  |
| 2619 | Dates | | | 5030003 | | | Dates, dried, dark brown | | |  |  |  |
| 2619 | Dates | | | 5030004 | | | Dates, dried, pale brown | | |  |  |  |
| 2620 | Grapes and products (excl wine) | | | 5020032 | | | Grapes, fresh fruit, raw | | |  |  |  |
| 2620 | Grapes and products (excl wine) | | | 5020033 | | | Grapes, seeded, round, black, raw | | |  |  |  |
| 2620 | Grapes and products (excl wine) | | | 5020034 | | | Grapes, seeded, round, green, raw | | |  |  |  |
| 2620 | Grapes and products (excl wine) | | | 5020035 | | | Grapes, seeded, round, red, raw | | |  |  |  |
| 2620 | Grapes and products (excl wine) | | | 5020036 | | | Grapes, seedless, oval, black, raw | | |  |  |  |
| 2620 | Grapes and products (excl wine) | | | 5020037 | | | Grapes, seedless, round, black, raw | | |  |  |  |
| 2620 | Grapes and products (excl wine) | | | 5020038 | | | Grapes, seedless, round, green, raw | | |  |  |  |
| 2625 | Fruits, Other | | | 5010013 | | | Mango, Banganapalli, ripe, raw | | |  |  |  |
| 2625 | Fruits, Other | | | 5010014 | | | Mango, Gulabkhas, ripe, raw | | |  |  |  |
| 2625 | Fruits, Other | | | 5010015 | | | Mango, Himsagar, ripe, raw | | |  |  |  |
| 2625 | Fruits, Other | | | 5010016 | | | Mango, Kesar, ripe, raw | | |  |  |  |
| 2625 | Fruits, Other | | | 5010017 | | | Mango, Neelam, ripe, raw | | |  |  |  |
| 2625 | Fruits, Other | | | 5010018 | | | Mango, Paheri, ripe, raw | | |  |  |  |
| 2625 | Fruits, Other | | | 5010019 | | | Mango, ripe, raw | | |  |  |  |
| 2625 | Fruits, Other | | | 5010020 | | | Mango, ripe, raw | | |  |  |  |
| 2625 | Fruits, Other | | | 5010021 | | | Mango, Totapari, ripe, raw | | |  |  |  |
| 2625 | Fruits, Other | | | 5010022 | | | Mangosteen, raw | | |  |  |  |
| 2625 | Fruits, Other | | | 5010023 | | | Melon, orange flesh, ripe, raw | | |  |  |  |
| 2625 | Fruits, Other | | | 5010025 | | | Monkey-jack, yellowish-orange flesh, raw | | |  |  |  |
| 2625 | Fruits, Other | | | 5010026 | | | Musk melon, light orange flesh, ripe, raw | | |  |  |  |
| 2625 | Fruits, Other | | | 5010027 | | | Musk melon, orange flesh, raw | | |  |  |  |
| 2625 | Fruits, Other | | | 5010028 | | | Musk melon, yellow flesh, raw | | |  |  |  |
| 2625 | Fruits, Other | | | 5010031 | | | Papaya, raw | | |  |  |  |
| 2625 | Fruits, Other | | | 5010032 | | | Papaya, ripe, raw | | |  |  |  |
| 2625 | Fruits, Other | | | 5010033 | | | Papaya, ripe, raw | | |  |  |  |
| 2625 | Fruits, Other | | | 5010034 | | | Passion fruit, fresh pulp, raw | | |  |  |  |
| 2625 | Fruits, Other | | | 5010039 | | | Pawpaw, ripe, yellow, waxed, raw | | |  |  |  |
| 2625 | Fruits, Other | | | 5010040 | | | Pawpaw, ripe, yellow, waxed, raw | | |  |  |  |
| 2625 | Fruits, Other | | | 5010041 | | | Peach, raw | | |  |  |  |
| 2625 | Fruits, Other | | | 5010044 | | | Pomelo, raw | | |  |  |  |
| 2625 | Fruits, Other | | | 5010045 | | | Star fruit, raw | | |  |  |  |
| 2625 | Fruits, Other | | | 5020008 | | | Avocado pear, raw | | |  |  |  |
| 2625 | Fruits, Other | | | 5020009 | | | Bael fruit, raw | | |  |  |  |
| 2625 | Fruits, Other | | | 5020017 | | | Blackberry, raw | | |  |  |  |
| 2625 | Fruits, Other | | | 5020019 | | | Breadfruit, raw | | |  |  |  |
| 2625 | Fruits, Other | | | 5020020 | | | Cherries, red, raw | | |  |  |  |
| 2625 | Fruits, Other | | | 5020022 | | | Currants, black, raw | | |  |  |  |
| 2625 | Fruits, Other | | | 5020023 | | | Custard apple, fresh whole, fruit without seeds | | |  |  |  |
| 2625 | Fruits, Other | | | 5020024 | | | Custard apple, raw | | |  |  |  |
| 2625 | Fruits, Other | | | 5020025 | | | Emblic, raw | | |  |  |  |
| 2625 | Fruits, Other | | | 5020026 | | | Fig, green, raw | | |  |  |  |
| 2625 | Fruits, Other | | | 5020027 | | | Fig, raw | | |  |  |  |
| 2625 | Fruits, Other | | | 5020028 | | | Fig, ripe, raw | | |  |  |  |
| 2625 | Fruits, Other | | | 5020031 | | | Gooseberry, raw | | |  |  |  |
| 2625 | Fruits, Other | | | 5020039 | | | Guava, fresh whole fruit, raw | | |  |  |  |
| 2625 | Fruits, Other | | | 5020040 | | | Guava, pink flesh, raw | | |  |  |  |
| 2625 | Fruits, Other | | | 5020041 | | | Guava, white flesh, raw | | |  |  |  |
| 2625 | Fruits, Other | | | 5020043 | | | Indian cucumber, whole fruit, raw | | |  |  |  |
| 2625 | Fruits, Other | | | 5020044 | | | Indian fig tree, whole fruit, raw | | |  |  |  |
| 2625 | Fruits, Other | | | 5020045 | | | Jackfruit, raw | | |  |  |  |
| 2625 | Fruits, Other | | | 5020046 | | | Jackfruit, ripe, raw | | |  |  |  |
| 2625 | Fruits, Other | | | 5020047 | | | Jambolan, raw | | |  |  |  |
| 2625 | Fruits, Other | | | 5020048 | | | Jambu fruit, ripe, raw | | |  |  |  |
| 2625 | Fruits, Other | | | 5020049 | | | Jujube, wihout seeds, raw | | |  |  |  |
| 2625 | Fruits, Other | | | 5020051 | | | Karonda fruit, raw | | |  |  |  |
| 2625 | Fruits, Other | | | 5020052 | | | Litchi, raw | | |  |  |  |
| 2625 | Fruits, Other | | | 5020053 | | | Mango, green, raw | | |  |  |  |
| 2625 | Fruits, Other | | | 5020054 | | | Mango, unripe, raw | | |  |  |  |
| 2625 | Fruits, Other | | | 5020055 | | | Manila tamarind, raw | | |  |  |  |
| 2625 | Fruits, Other | | | 5020056 | | | Mello, fresh whole fruit, raw | | |  |  |  |
| 2625 | Fruits, Other | | | 5020057 | | | Pear, raw | | |  |  |  |
| 2625 | Fruits, Other | | | 5020059 | | | Plum, raw | | |  |  |  |
| 2625 | Fruits, Other | | | 5020060 | | | Pomegranate, maroon seeds, raw | | |  |  |  |
| 2625 | Fruits, Other | | | 5020061 | | | Prickly pear, fresh flesh | | |  |  |  |
| 2625 | Fruits, Other | | | 5020062 | | | Quince, OHM13, fruit, raw | | |  |  |  |
| 2625 | Fruits, Other | | | 5020063 | | | Quince, OHM14, fruit, raw | | |  |  |  |
| 2625 | Fruits, Other | | | 5020064 | | | Quince, OHM2, fruit, raw | | |  |  |  |
| 2625 | Fruits, Other | | | 5020065 | | | Quince, PUM, fruit, raw | | |  |  |  |
| 2625 | Fruits, Other | | | 5020066 | | | Quince, ZM6, fruit, raw | | |  |  |  |
| 2625 | Fruits, Other | | | 5020067 | | | Quince, ZM9, fruit, raw | | |  |  |  |
| 2625 | Fruits, Other | | | 5020068 | | | Rambutan, raw | | |  |  |  |
| 2625 | Fruits, Other | | | 5020072 | | | Strawberry, raw | | |  |  |  |
| 2625 | Fruits, Other | | | 5020077 | | | Watermelon (tinda), raw | | |  |  |  |
| 2625 | Fruits, Other | | | 5020078 | | | Watermelon, dark green, raw | | |  |  |  |
| 2625 | Fruits, Other | | | 5020079 | | | Watermelon, pale green, raw | | |  |  |  |
| 2625 | Fruits, Other | | | 5020080 | | | Watermelon, ripe, raw | | |  |  |  |
| 2625 | Fruits, Other | | | 5020083 | | | Zizyphus, raw | | |  |  |  |
| 2630 | Coffee and products | | | 13030007 | | | Coffee for brewing, dried, Maxwell House, Master Blend | | |  |  |  |
| 2633 | Cocoa Beans and products | | | 13030002 | | | Cocoa powder, industrial | | |  |  |  |
| 2633 | Cocoa Beans and products | | | 13030003 | | | Cocoa powder, industrial | | |  |  |  |
| 2633 | Cocoa Beans and products | | | 13030004 | | | Cocoa powder, industrial | | |  |  |  |
| 2633 | Cocoa Beans and products | | | 13030005 | | | Cocoa powder, industrial | | |  |  |  |
| 2633 | Cocoa Beans and products | | | 13030006 | | | Cocoa powder, industrial | | |  |  |  |
| 2635 | Tea (including mate) | | | 13030020 | | | Tea for brewing, dried, Lipton, Decaffeinated | | |  |  |  |
| 2635 | Tea (including mate) | | | 13030021 | | | Tea, instant, Nestea | | |  |  |  |
| 2640 | Pepper | | | 15010008 | | | African pepper seed, dried | | |  |  |  |
| 2640 | Pepper | | | 15010014 | | | Black pepper, dried | | |  |  |  |
| 2640 | Pepper | | | 15010015 | | | Black pepper, dried, powdered | | |  |  |  |
| 2640 | Pepper | | | 15010066 | | | Lalot pepper, leaves, raw | | |  |  |  |
| 2640 | Pepper | | | 15010078 | | | Pippali, dried | | |  |  |  |
| 2641 | Pimento | | | 15010020 | | | Chili pepper, dried | | |  |  |  |
| 2641 | Pimento | | | 15010030 | | | Chili pepper, red, dried | | |  |  |  |
| 2641 | Pimento | | | 15010080 | | | Red chilies, dried, powdered | | |  |  |  |
| 2641 | Pimento | | | 15010083 | | | Sweet pepper, seed kernel | | |  |  |  |
| 2642 | Cloves | | | 15010032 | | | Clove, dried | | |  |  |  |
| 2642 | Cloves | | | 15010034 | | | Clove, seed, dried | | |  |  |  |
| 2645 | Spices, Other | | | 4010106 | | | Cassia, leaves, fresh, raw | | |  |  |  |
| 2645 | Spices, Other | | | 6010008 | | | African nutmeg, seed, dried | | |  |  |  |
| 2645 | Spices, Other | | | 6010009 | | | African nutmeg, seed, dried | | |  |  |  |
| 2645 | Spices, Other | | | 15010001 | | | African basil (scent leaves), dried | | |  |  |  |
| 2645 | Spices, Other | | | 15010002 | | | African basil (scent leaves), raw | | |  |  |  |
| 2645 | Spices, Other | | | 15010003 | | | African basil, dried | | |  |  |  |
| 2645 | Spices, Other | | | 15010004 | | | African basil, leaves, raw | | |  |  |  |
| 2645 | Spices, Other | | | 15010005 | | | African basil, raw | | |  |  |  |
| 2645 | Spices, Other | | | 15010009 | | | African spice, 'Cochlospermum spp.', rootâs powder | | |  |  |  |
| 2645 | Spices, Other | | | 15010010 | | | Ajowan, dried, powdered | | |  |  |  |
| 2645 | Spices, Other | | | 15010018 | | | Cardamom, black, dried | | |  |  |  |
| 2645 | Spices, Other | | | 15010019 | | | Cardamom, green, dried | | |  |  |  |
| 2645 | Spices, Other | | | 15010031 | | | Cinnamon, leaves, dried | | |  |  |  |
| 2645 | Spices, Other | | | 15010036 | | | Coriander, dried, powdered | | |  |  |  |
| 2645 | Spices, Other | | | 15010037 | | | Coriander, leaves, raw | | |  |  |  |
| 2645 | Spices, Other | | | 15010038 | | | Coriander, seeds, dried | | |  |  |  |
| 2645 | Spices, Other | | | 15010039 | | | Culantro, leaves, raw | | |  |  |  |
| 2645 | Spices, Other | | | 15010040 | | | Culantro, leaves, raw | | |  |  |  |
| 2645 | Spices, Other | | | 15010041 | | | Cumin, seeds, dried | | |  |  |  |
| 2645 | Spices, Other | | | 15010042 | | | Cumin, seeds, dried, powdered | | |  |  |  |
| 2645 | Spices, Other | | | 15010043 | | | Curry leaves, raw | | |  |  |  |
| 2645 | Spices, Other | | | 15010044 | | | Curry tree, leaves, raw | | |  |  |  |
| 2645 | Spices, Other | | | 15010049 | | | Fenugreek seed, dried | | |  |  |  |
| 2645 | Spices, Other | | | 15010050 | | | Fenugreek seed, Pusa, dried | | |  |  |  |
| 2645 | Spices, Other | | | 15010053 | | | Fenugreek seed, raw | | |  |  |  |
| 2645 | Spices, Other | | | 15010054 | | | Fenugreek seed, white, roasted, powdered | | |  |  |  |
| 2645 | Spices, Other | | | 15010060 | | | Ginger, dried, powdered | | |  |  |  |
| 2645 | Spices, Other | | | 15010061 | | | Ginger, fresh, raw | | |  |  |  |
| 2645 | Spices, Other | | | 15010067 | | | Mace, dried | | |  |  |  |
| 2645 | Spices, Other | | | 15010068 | | | Mango ginger, raw | | |  |  |  |
| 2645 | Spices, Other | | | 15010069 | | | Mentha, leaves, raw | | |  |  |  |
| 2645 | Spices, Other | | | 15010070 | | | Mexican mint, leaves, water-washed, raw | | |  |  |  |
| 2645 | Spices, Other | | | 15010071 | | | Mint, leaves, raw | | |  |  |  |
| 2645 | Spices, Other | | | 15010072 | | | Nutmeg, seed, dried | | |  |  |  |
| 2645 | Spices, Other | | | 15010073 | | | Nutmeg, seed, dried | | |  |  |  |
| 2645 | Spices, Other | | | 15010074 | | | Omum, dried | | |  |  |  |
| 2645 | Spices, Other | | | 15010076 | | | Pepper, Alligator, big variety, seed, dried | | |  |  |  |
| 2645 | Spices, Other | | | 15010077 | | | Pepper, Alligator, small variety, seed, dried | | |  |  |  |
| 2645 | Spices, Other | | | 15010079 | | | Poppy seeds, dried | | |  |  |  |
| 2645 | Spices, Other | | | 15010084 | | | Turmeric, dried, powdered | | |  |  |  |
| 2761 | Freshwater Fish | | | 10010001 | | | Tilapia fish, boiled | | |  |  |  |
| 2761 | Freshwater Fish | | | 10070003 | | | Tilapia fish, dried | | |  |  |  |
| 2763 | Pelagic Fish | | | 10020001 | | | Herring (west african herring), boiled | | |  |  |  |
| 2763 | Pelagic Fish | | | 10030001 | | | Spanish fish, large | | |  |  |  |
| 2763 | Pelagic Fish | | | 10070001 | | | Herring (west african herring), dried | | |  |  |  |
| 2763 | Pelagic Fish | | | 10070002 | | | Spanish fish, dried | | |  |  |  |
| 2764 | Marine Fish, Other | | | 10010001 | | | Tilapia fish, boiled | | |  |  |  |
| 2764 | Marine Fish, Other | | | 10020001 | | | Herring (west african herring), boiled | | |  |  |  |
| 2764 | Marine Fish, Other | | | 10030001 | | | Spanish fish, large | | |  |  |  |
| 2764 | Marine Fish, Other | | | 10070001 | | | Herring (west african herring), dried | | |  |  |  |
| 2764 | Marine Fish, Other | | | 10070002 | | | Spanish fish, dried | | |  |  |  |
| 2764 | Marine Fish, Other | | | 10070003 | | | Tilapia fish, dried | | |  |  |  |
| 2775 | Aquatic Plants | | | 2050018 | | | Lotus root, raw | | |  |  |  |
| 2775 | Aquatic Plants | | | 4010247 | | | Water chestnut, leaves, raw | | |  |  |  |
| 2775 | Aquatic Plants | | | 4010249 | | | Water hyacinth, leaves, raw | | |  |  |  |
| 2775 | Aquatic Plants | | | 4010250 | | | Water spinach, leaves, raw | | |  |  |  |
| 2775 | Aquatic Plants | | | 4010251 | | | Water spinach, leaves, raw | | |  |  |  |
| 2775 | Aquatic Plants | | | 4010252 | | | Water spinach, leaves, raw | | |  |  |  |
| 2775 | Aquatic Plants | | | 4010253 | | | Water spinach, raw | | |  |  |  |
| 2805 | Rice (Milled Equivalent) | | | 1010063 | | | Rice, Agb0101, brown, genetically modified, raw | | |  |  |  |
| 2805 | Rice (Milled Equivalent) | | | 1010064 | | | Rice, Anjung, conventional, brown, raw | | |  |  |  |
| 2805 | Rice (Milled Equivalent) | | | 1010065 | | | Rice, Bar68-1, brown, genetically modified, raw, milled | | |  |  |  |
| 2805 | Rice (Milled Equivalent) | | | 1010066 | | | Rice, Bar68-1, brown, genetically modified, raw | | |  |  |  |
| 2805 | Rice (Milled Equivalent) | | | 1010067 | | | Rice, Basmati, parboiled | | |  |  |  |
| 2805 | Rice (Milled Equivalent) | | | 1010068 | | | Rice, Bengal, rough, nontransgenic, conventional herbicide system, raw | | |  |  |  |
| 2805 | Rice (Milled Equivalent) | | | 1010069 | | | Rice, Bengal, rough, transgenic, conventional herbicide system, raw | | |  |  |  |
| 2805 | Rice (Milled Equivalent) | | | 1010070 | | | Rice, Bengal, rough, transgenic, liberty herbicide system, raw | | |  |  |  |
| 2805 | Rice (Milled Equivalent) | | | 1010071 | | | Rice, Bg 352/Bg300, parboiled | | |  |  |  |
| 2805 | Rice (Milled Equivalent) | | | 1010072 | | | Rice, Bg 352/Bg300, parboiled | | |  |  |  |
| 2805 | Rice (Milled Equivalent) | | | 1010073 | | | Rice, Bg 352/Bg300, parboiled | | |  |  |  |
| 2805 | Rice (Milled Equivalent) | | | 1010074 | | | Rice, Bg 352/Bg300, parboiled | | |  |  |  |
| 2805 | Rice (Milled Equivalent) | | | 1010075 | | | Rice, Bg 352/Bg300, raw | | |  |  |  |
| 2805 | Rice (Milled Equivalent) | | | 1010076 | | | Rice, Bg 352/Bg300, raw | | |  |  |  |
| 2805 | Rice (Milled Equivalent) | | | 1010077 | | | Rice, Bg 352/Bg300, raw | | |  |  |  |
| 2805 | Rice (Milled Equivalent) | | | 1010078 | | | Rice, Bg 352/Bg300, raw | | |  |  |  |
| 2805 | Rice (Milled Equivalent) | | | 1010079 | | | Rice, Bg 379-2, parboiled | | |  |  |  |
| 2805 | Rice (Milled Equivalent) | | | 1010080 | | | Rice, Bg 379-2, parboiled | | |  |  |  |
| 2805 | Rice (Milled Equivalent) | | | 1010081 | | | Rice, Bg 379-2, raw | | |  |  |  |
| 2805 | Rice (Milled Equivalent) | | | 1010082 | | | Rice, Bg 379-2, raw | | |  |  |  |
| 2805 | Rice (Milled Equivalent) | | | 1010083 | | | Rice, Bg403, raw | | |  |  |  |
| 2805 | Rice (Milled Equivalent) | | | 1010084 | | | Rice, Bg94-1/At354, parboiled | | |  |  |  |
| 2805 | Rice (Milled Equivalent) | | | 1010085 | | | Rice, Bg94-1/At354, raw | | |  |  |  |
| 2805 | Rice (Milled Equivalent) | | | 1010086 | | | Rice, Bg94-1/At354, raw | | |  |  |  |
| 2805 | Rice (Milled Equivalent) | | | 1010087 | | | Rice, black, raw | | |  |  |  |
| 2805 | Rice (Milled Equivalent) | | | 1010091 | | | Rice, BR-28, parboiled, milled | | |  |  |  |
| 2805 | Rice (Milled Equivalent) | | | 1010092 | | | Rice, brown, dried, Manischewitz (kosher) | | |  |  |  |
| 2805 | Rice (Milled Equivalent) | | | 1010094 | | | Rice, brown, raw | | |  |  |  |
| 2805 | Rice (Milled Equivalent) | | | 1010095 | | | Rice, brown, raw | | |  |  |  |
| 2805 | Rice (Milled Equivalent) | | | 1010096 | | | Rice, brown, raw | | |  |  |  |
| 2805 | Rice (Milled Equivalent) | | | 1010097 | | | Rice, brown, raw | | |  |  |  |
| 2805 | Rice (Milled Equivalent) | | | 1010099 | | | Rice, Calrose, brown, raw | | |  |  |  |
| 2805 | Rice (Milled Equivalent) | | | 1010102 | | | Rice, D68, brown, raw, milled | | |  |  |  |
| 2805 | Rice (Milled Equivalent) | | | 1010103 | | | Rice, D68, brown, raw | | |  |  |  |
| 2805 | Rice (Milled Equivalent) | | | 1010104 | | | Rice, Dongjin, conventional, brown, raw | | |  |  |  |
| 2805 | Rice (Milled Equivalent) | | | 1010107 | | | Rice, Giant embryonic, raw | | |  |  |  |
| 2805 | Rice (Milled Equivalent) | | | 1010108 | | | Rice, Goami, raw | | |  |  |  |
| 2805 | Rice (Milled Equivalent) | | | 1010109 | | | Rice, Green, raw | | |  |  |  |
| 2805 | Rice (Milled Equivalent) | | | 1010110 | | | Rice, H-4, parboiled | | |  |  |  |
| 2805 | Rice (Milled Equivalent) | | | 1010111 | | | Rice, H-4, raw | | |  |  |  |
| 2805 | Rice (Milled Equivalent) | | | 1010112 | | | Rice, Heilongjiang, raw | | |  |  |  |
| 2805 | Rice (Milled Equivalent) | | | 1010113 | | | Rice, Iksan483, genetically modified, brown, raw | | |  |  |  |
| 2805 | Rice (Milled Equivalent) | | | 1010114 | | | Rice, Indica, brown, raw | | |  |  |  |
| 2805 | Rice (Milled Equivalent) | | | 1010115 | | | Rice, Indica, dehulled, raw, milled | | |  |  |  |
| 2805 | Rice (Milled Equivalent) | | | 1010116 | | | Rice, Indica, Liangyou 2186, brown, raw | | |  |  |  |
| 2805 | Rice (Milled Equivalent) | | | 1010117 | | | Rice, Indica, Liangyou 2186, milled, raw | | |  |  |  |
| 2805 | Rice (Milled Equivalent) | | | 1010118 | | | Rice, Indica, Liangyou Kefeng No. 6, brown, genetically modified, raw | | |  |  |  |
| 2805 | Rice (Milled Equivalent) | | | 1010119 | | | Rice, Indica, Liangyou Kefeng No. 6, genetically modified, milled, raw | | |  |  |  |
| 2805 | Rice (Milled Equivalent) | | | 1010130 | | | Rice, IRR, brown, raw | | |  |  |  |
| 2805 | Rice (Milled Equivalent) | | | 1010131 | | | Rice, IRR, dehulled, milled, raw | | |  |  |  |
| 2805 | Rice (Milled Equivalent) | | | 1010132 | | | Rice, Japonica, Huai269, dehulled, dried, high N fertilization | | |  |  |  |
| 2805 | Rice (Milled Equivalent) | | | 1010133 | | | Rice, Japonica, Huai269, dehulled, dried, high N fertilization | | |  |  |  |
| 2805 | Rice (Milled Equivalent) | | | 1010134 | | | Rice, Japonica, Huai269, dehulled, dried, moderate N fertilization | | |  |  |  |
| 2805 | Rice (Milled Equivalent) | | | 1010135 | | | Rice, Japonica, Huai269, dehulled, dried, moderate N fertilization | | |  |  |  |
| 2805 | Rice (Milled Equivalent) | | | 1010136 | | | Rice, Japonica, Huai269, dehulled, dried, without N fertilization | | |  |  |  |
| 2805 | Rice (Milled Equivalent) | | | 1010137 | | | Rice, Japonica, Huaidao6, dehulled, dried, high N fertilization | | |  |  |  |
| 2805 | Rice (Milled Equivalent) | | | 1010138 | | | Rice, Japonica, Huaidao6, dehulled, dried, high N fertilization | | |  |  |  |
| 2805 | Rice (Milled Equivalent) | | | 1010139 | | | Rice, Japonica, Huaidao6, dehulled, dried, moderate N fertilization | | |  |  |  |
| 2805 | Rice (Milled Equivalent) | | | 1010140 | | | Rice, Japonica, Huaidao6, dehulled, dried, moderate N fertilization | | |  |  |  |
| 2805 | Rice (Milled Equivalent) | | | 1010141 | | | Rice, Japonica, Huaidao6, dehulled, dried, without N fertilization | | |  |  |  |
| 2805 | Rice (Milled Equivalent) | | | 1010142 | | | Rice, Japonica, Huaidao7, dehulled, dried, high N fertilization | | |  |  |  |
| 2805 | Rice (Milled Equivalent) | | | 1010143 | | | Rice, Japonica, Huaidao7, dehulled, dried, high N fertilization | | |  |  |  |
| 2805 | Rice (Milled Equivalent) | | | 1010144 | | | Rice, Japonica, Huaidao7, dehulled, dried, moderate N fertilization | | |  |  |  |
| 2805 | Rice (Milled Equivalent) | | | 1010145 | | | Rice, Japonica, Huaidao7, dehulled, dried, moderate N fertilization | | |  |  |  |
| 2805 | Rice (Milled Equivalent) | | | 1010146 | | | Rice, Japonica, Huaidao7, dehulled, dried, without N fertilization | | |  |  |  |
| 2805 | Rice (Milled Equivalent) | | | 1010147 | | | Rice, Japonica, Huaidao9, dehulled, dried, high N fertilization | | |  |  |  |
| 2805 | Rice (Milled Equivalent) | | | 1010148 | | | Rice, Japonica, Huaidao9, dehulled, dried, high N fertilization | | |  |  |  |
| 2805 | Rice (Milled Equivalent) | | | 1010149 | | | Rice, Japonica, Huaidao9, dehulled, dried, moderate N fertilization | | |  |  |  |
| 2805 | Rice (Milled Equivalent) | | | 1010150 | | | Rice, Japonica, Huaidao9, dehulled, dried, moderate N fertilization | | |  |  |  |
| 2805 | Rice (Milled Equivalent) | | | 1010151 | | | Rice, Japonica, Huaidao9, dehulled, dried, without N fertilization | | |  |  |  |
| 2805 | Rice (Milled Equivalent) | | | 1010152 | | | Rice, Japonica, Huajing6, dehulled, dried, high N fertilization | | |  |  |  |
| 2805 | Rice (Milled Equivalent) | | | 1010153 | | | Rice, Japonica, Huajing6, dehulled, dried, high N fertilization | | |  |  |  |
| 2805 | Rice (Milled Equivalent) | | | 1010154 | | | Rice, Japonica, Huajing6, dehulled, dried, moderate N fertilization | | |  |  |  |
| 2805 | Rice (Milled Equivalent) | | | 1010155 | | | Rice, Japonica, Huajing6, dehulled, dried, moderate N fertilization | | |  |  |  |
| 2805 | Rice (Milled Equivalent) | | | 1010156 | | | Rice, Japonica, Huajing6, dehulled, dried, without N fertilization | | |  |  |  |
| 2805 | Rice (Milled Equivalent) | | | 1010157 | | | Rice, Japonica, Nanjing43, dehulled, dried, high N fertilization | | |  |  |  |
| 2805 | Rice (Milled Equivalent) | | | 1010158 | | | Rice, Japonica, Nanjing43, dehulled, dried, high N fertilization | | |  |  |  |
| 2805 | Rice (Milled Equivalent) | | | 1010159 | | | Rice, Japonica, Nanjing43, dehulled, dried, moderate N fertilization | | |  |  |  |
| 2805 | Rice (Milled Equivalent) | | | 1010160 | | | Rice, Japonica, Nanjing43, dehulled, dried, moderate N fertilization | | |  |  |  |
| 2805 | Rice (Milled Equivalent) | | | 1010161 | | | Rice, Japonica, Nanjing43, dehulled, dried, without N fertilization | | |  |  |  |
| 2805 | Rice (Milled Equivalent) | | | 1010162 | | | Rice, Japonica, Ningjing1, dehulled, dried, high N fertilization | | |  |  |  |
| 2805 | Rice (Milled Equivalent) | | | 1010163 | | | Rice, Japonica, Ningjing1, dehulled, dried, high N fertilization | | |  |  |  |
| 2805 | Rice (Milled Equivalent) | | | 1010164 | | | Rice, Japonica, Ningjing1, dehulled, dried, moderate N fertilization | | |  |  |  |
| 2805 | Rice (Milled Equivalent) | | | 1010165 | | | Rice, Japonica, Ningjing1, dehulled, dried, moderate N fertilization | | |  |  |  |
| 2805 | Rice (Milled Equivalent) | | | 1010166 | | | Rice, Japonica, Ningjing1, dehulled, dried, without N fertilization | | |  |  |  |
| 2805 | Rice (Milled Equivalent) | | | 1010167 | | | Rice, Japonica, Ningjing2, dehulled, dried, high N fertilization | | |  |  |  |
| 2805 | Rice (Milled Equivalent) | | | 1010168 | | | Rice, Japonica, Ningjing2, dehulled, dried, high N fertilization | | |  |  |  |
| 2805 | Rice (Milled Equivalent) | | | 1010169 | | | Rice, Japonica, Ningjing2, dehulled, dried, moderate N fertilization | | |  |  |  |
| 2805 | Rice (Milled Equivalent) | | | 1010170 | | | Rice, Japonica, Ningjing2, dehulled, dried, moderate N fertilization | | |  |  |  |
| 2805 | Rice (Milled Equivalent) | | | 1010171 | | | Rice, Japonica, Ningjing2, dehulled, dried, without N fertilization | | |  |  |  |
| 2805 | Rice (Milled Equivalent) | | | 1010172 | | | Rice, Japonica, Ningjing3, dehulled, dried, high N fertilization | | |  |  |  |
| 2805 | Rice (Milled Equivalent) | | | 1010173 | | | Rice, Japonica, Ningjing3, dehulled, dried, high N fertilization | | |  |  |  |
| 2805 | Rice (Milled Equivalent) | | | 1010174 | | | Rice, Japonica, Ningjing3, dehulled, dried, moderate N fertilization | | |  |  |  |
| 2805 | Rice (Milled Equivalent) | | | 1010175 | | | Rice, Japonica, Ningjing3, dehulled, dried, moderate N fertilization | | |  |  |  |
| 2805 | Rice (Milled Equivalent) | | | 1010176 | | | Rice, Japonica, Ningjing3, dehulled, dried, without N fertilization | | |  |  |  |
| 2805 | Rice (Milled Equivalent) | | | 1010177 | | | Rice, Japonica, Sidao10, dehulled, dried, high N fertilization | | |  |  |  |
| 2805 | Rice (Milled Equivalent) | | | 1010178 | | | Rice, Japonica, Sidao10, dehulled, dried, high N fertilization | | |  |  |  |
| 2805 | Rice (Milled Equivalent) | | | 1010179 | | | Rice, Japonica, Sidao10, dehulled, dried, moderate N fertilization | | |  |  |  |
| 2805 | Rice (Milled Equivalent) | | | 1010180 | | | Rice, Japonica, Sidao10, dehulled, dried, moderate N fertilization | | |  |  |  |
| 2805 | Rice (Milled Equivalent) | | | 1010181 | | | Rice, Japonica, Sidao10, dehulled, dried, without N fertilization | | |  |  |  |
| 2805 | Rice (Milled Equivalent) | | | 1010182 | | | Rice, Japonica, Sujing45, dehulled, dried, high N fertilization | | |  |  |  |
| 2805 | Rice (Milled Equivalent) | | | 1010183 | | | Rice, Japonica, Sujing45, dehulled, dried, high N fertilization | | |  |  |  |
| 2805 | Rice (Milled Equivalent) | | | 1010184 | | | Rice, Japonica, Sujing45, dehulled, dried, moderate N fertilization | | |  |  |  |
| 2805 | Rice (Milled Equivalent) | | | 1010185 | | | Rice, Japonica, Sujing45, dehulled, dried, moderate N fertilization | | |  |  |  |
| 2805 | Rice (Milled Equivalent) | | | 1010186 | | | Rice, Japonica, Sujing45, dehulled, dried, without N fertilization | | |  |  |  |
| 2805 | Rice (Milled Equivalent) | | | 1010187 | | | Rice, Japonica, W3668, dehulled, dried, high N fertilization | | |  |  |  |
| 2805 | Rice (Milled Equivalent) | | | 1010188 | | | Rice, Japonica, W3668, dehulled, dried, high N fertilization | | |  |  |  |
| 2805 | Rice (Milled Equivalent) | | | 1010189 | | | Rice, Japonica, W3668, dehulled, dried, moderate N fertilization | | |  |  |  |
| 2805 | Rice (Milled Equivalent) | | | 1010190 | | | Rice, Japonica, W3668, dehulled, dried, moderate N fertilization | | |  |  |  |
| 2805 | Rice (Milled Equivalent) | | | 1010191 | | | Rice, Japonica, W3668, dehulled, dried, without N fertilization | | |  |  |  |
| 2805 | Rice (Milled Equivalent) | | | 1010192 | | | Rice, Japonica, Wu9516, dehulled, dried, high N fertilization | | |  |  |  |
| 2805 | Rice (Milled Equivalent) | | | 1010193 | | | Rice, Japonica, Wu9516, dehulled, dried, high N fertilization | | |  |  |  |
| 2805 | Rice (Milled Equivalent) | | | 1010194 | | | Rice, Japonica, Wu9516, dehulled, dried, moderate N fertilization | | |  |  |  |
| 2805 | Rice (Milled Equivalent) | | | 1010195 | | | Rice, Japonica, Wu9516, dehulled, dried, moderate N fertilization | | |  |  |  |
| 2805 | Rice (Milled Equivalent) | | | 1010196 | | | Rice, Japonica, Wu9516, dehulled, dried, without N fertilization | | |  |  |  |
| 2805 | Rice (Milled Equivalent) | | | 1010197 | | | Rice, Japonica, Wu9520, dehulled, dried, high N fertilization | | |  |  |  |
| 2805 | Rice (Milled Equivalent) | | | 1010198 | | | Rice, Japonica, Wu9520, dehulled, dried, high N fertilization | | |  |  |  |
| 2805 | Rice (Milled Equivalent) | | | 1010199 | | | Rice, Japonica, Wu9520, dehulled, dried, moderate N fertilization | | |  |  |  |
| 2805 | Rice (Milled Equivalent) | | | 1010200 | | | Rice, Japonica, Wu9520, dehulled, dried, moderate N fertilization | | |  |  |  |
| 2805 | Rice (Milled Equivalent) | | | 1010201 | | | Rice, Japonica, Wu9520, dehulled, dried, without N fertilization | | |  |  |  |
| 2805 | Rice (Milled Equivalent) | | | 1010202 | | | Rice, Japonica, Wu9522, dehulled, dried, high N fertilization | | |  |  |  |
| 2805 | Rice (Milled Equivalent) | | | 1010203 | | | Rice, Japonica, Wu9522, dehulled, dried, high N fertilization | | |  |  |  |
| 2805 | Rice (Milled Equivalent) | | | 1010204 | | | Rice, Japonica, Wu9522, dehulled, dried, moderate N fertilization | | |  |  |  |
| 2805 | Rice (Milled Equivalent) | | | 1010205 | | | Rice, Japonica, Wu9522, dehulled, dried, moderate N fertilization | | |  |  |  |
| 2805 | Rice (Milled Equivalent) | | | 1010206 | | | Rice, Japonica, Wu9522, dehulled, dried, without N fertilization | | |  |  |  |
| 2805 | Rice (Milled Equivalent) | | | 1010207 | | | Rice, Japonica, Wu9746, dehulled, dried, high N fertilization | | |  |  |  |
| 2805 | Rice (Milled Equivalent) | | | 1010208 | | | Rice, Japonica, Wu9746, dehulled, dried, high N fertilization | | |  |  |  |
| 2805 | Rice (Milled Equivalent) | | | 1010209 | | | Rice, Japonica, Wu9746, dehulled, dried, moderate N fertilization | | |  |  |  |
| 2805 | Rice (Milled Equivalent) | | | 1010210 | | | Rice, Japonica, Wu9746, dehulled, dried, moderate N fertilization | | |  |  |  |
| 2805 | Rice (Milled Equivalent) | | | 1010211 | | | Rice, Japonica, Wu9746, dehulled, dried, without N fertilization | | |  |  |  |
| 2805 | Rice (Milled Equivalent) | | | 1010212 | | | Rice, Japonica, Wujing15, dehulled, dried, high N fertilization | | |  |  |  |
| 2805 | Rice (Milled Equivalent) | | | 1010213 | | | Rice, Japonica, Wujing15, dehulled, dried, high N fertilization | | |  |  |  |
| 2805 | Rice (Milled Equivalent) | | | 1010214 | | | Rice, Japonica, Wujing15, dehulled, dried, moderate N fertilization | | |  |  |  |
| 2805 | Rice (Milled Equivalent) | | | 1010215 | | | Rice, Japonica, Wujing15, dehulled, dried, moderate N fertilization | | |  |  |  |
| 2805 | Rice (Milled Equivalent) | | | 1010216 | | | Rice, Japonica, Wujing15, dehulled, dried, without N fertilization | | |  |  |  |
| 2805 | Rice (Milled Equivalent) | | | 1010217 | | | Rice, Japonica, Wuyujing3, dehulled, dried, high N fertilization | | |  |  |  |
| 2805 | Rice (Milled Equivalent) | | | 1010218 | | | Rice, Japonica, Wuyujing3, dehulled, dried, high N fertilization | | |  |  |  |
| 2805 | Rice (Milled Equivalent) | | | 1010219 | | | Rice, Japonica, Wuyujing3, dehulled, dried, moderate N fertilization | | |  |  |  |
| 2805 | Rice (Milled Equivalent) | | | 1010220 | | | Rice, Japonica, Wuyujing3, dehulled, dried, moderate N fertilization | | |  |  |  |
| 2805 | Rice (Milled Equivalent) | | | 1010221 | | | Rice, Japonica, Wuyujing3, dehulled, dried, without N fertilization | | |  |  |  |
| 2805 | Rice (Milled Equivalent) | | | 1010222 | | | Rice, Japonica, Xiushui09, dehulled, dried, high N fertilization | | |  |  |  |
| 2805 | Rice (Milled Equivalent) | | | 1010223 | | | Rice, Japonica, Xiushui09, dehulled, dried, high N fertilization | | |  |  |  |
| 2805 | Rice (Milled Equivalent) | | | 1010224 | | | Rice, Japonica, Xiushui09, dehulled, dried, moderate N fertilization | | |  |  |  |
| 2805 | Rice (Milled Equivalent) | | | 1010225 | | | Rice, Japonica, Xiushui09, dehulled, dried, moderate N fertilization | | |  |  |  |
| 2805 | Rice (Milled Equivalent) | | | 1010226 | | | Rice, Japonica, Xiushui09, dehulled, dried, without N fertilization | | |  |  |  |
| 2805 | Rice (Milled Equivalent) | | | 1010227 | | | Rice, Japonica, Xiushui11, dehulled, dried, high N fertilization | | |  |  |  |
| 2805 | Rice (Milled Equivalent) | | | 1010228 | | | Rice, Japonica, Xiushui11, dehulled, dried, high N fertilization | | |  |  |  |
| 2805 | Rice (Milled Equivalent) | | | 1010229 | | | Rice, Japonica, Xiushui11, dehulled, dried, moderate N fertilization | | |  |  |  |
| 2805 | Rice (Milled Equivalent) | | | 1010230 | | | Rice, Japonica, Xiushui11, dehulled, dried, moderate N fertilization | | |  |  |  |
| 2805 | Rice (Milled Equivalent) | | | 1010231 | | | Rice, Japonica, Xiushui11, dehulled, dried, without N fertilization | | |  |  |  |
| 2805 | Rice (Milled Equivalent) | | | 1010232 | | | Rice, Japonica, Xiushui63, dehulled, dried, high N fertilization | | |  |  |  |
| 2805 | Rice (Milled Equivalent) | | | 1010233 | | | Rice, Japonica, Xiushui63, dehulled, dried, high N fertilization | | |  |  |  |
| 2805 | Rice (Milled Equivalent) | | | 1010234 | | | Rice, Japonica, Xiushui63, dehulled, dried, moderate N fertilization | | |  |  |  |
| 2805 | Rice (Milled Equivalent) | | | 1010235 | | | Rice, Japonica, Xiushui63, dehulled, dried, moderate N fertilization | | |  |  |  |
| 2805 | Rice (Milled Equivalent) | | | 1010236 | | | Rice, Japonica, Xiushui63, dehulled, dried, without N fertilization | | |  |  |  |
| 2805 | Rice (Milled Equivalent) | | | 1010237 | | | Rice, Japonica, Xudao3, dehulled, dried, high N fertilization | | |  |  |  |
| 2805 | Rice (Milled Equivalent) | | | 1010238 | | | Rice, Japonica, Xudao3, dehulled, dried, high N fertilization | | |  |  |  |
| 2805 | Rice (Milled Equivalent) | | | 1010239 | | | Rice, Japonica, Xudao3, dehulled, dried, moderate N fertilization | | |  |  |  |
| 2805 | Rice (Milled Equivalent) | | | 1010240 | | | Rice, Japonica, Xudao3, dehulled, dried, moderate N fertilization | | |  |  |  |
| 2805 | Rice (Milled Equivalent) | | | 1010241 | | | Rice, Japonica, Xudao3, dehulled, dried, without N fertilization | | |  |  |  |
| 2805 | Rice (Milled Equivalent) | | | 1010242 | | | Rice, Japonica, Xudao4, dehulled, dried, high N fertilization | | |  |  |  |
| 2805 | Rice (Milled Equivalent) | | | 1010243 | | | Rice, Japonica, Xudao4, dehulled, dried, high N fertilization | | |  |  |  |
| 2805 | Rice (Milled Equivalent) | | | 1010244 | | | Rice, Japonica, Xudao4, dehulled, dried, moderate N fertilization | | |  |  |  |
| 2805 | Rice (Milled Equivalent) | | | 1010245 | | | Rice, Japonica, Xudao4, dehulled, dried, moderate N fertilization | | |  |  |  |
| 2805 | Rice (Milled Equivalent) | | | 1010246 | | | Rice, Japonica, Xudao4, dehulled, dried, without N fertilization | | |  |  |  |
| 2805 | Rice (Milled Equivalent) | | | 1010247 | | | Rice, Japonica, Xudao5, dehulled, dried, high N fertilization | | |  |  |  |
| 2805 | Rice (Milled Equivalent) | | | 1010248 | | | Rice, Japonica, Xudao5, dehulled, dried, high N fertilization | | |  |  |  |
| 2805 | Rice (Milled Equivalent) | | | 1010249 | | | Rice, Japonica, Xudao5, dehulled, dried, moderate N fertilization | | |  |  |  |
| 2805 | Rice (Milled Equivalent) | | | 1010250 | | | Rice, Japonica, Xudao5, dehulled, dried, moderate N fertilization | | |  |  |  |
| 2805 | Rice (Milled Equivalent) | | | 1010251 | | | Rice, Japonica, Xudao5, dehulled, dried, without N fertilization | | |  |  |  |
| 2805 | Rice (Milled Equivalent) | | | 1010252 | | | Rice, Japonica, Yanjing2, dehulled, dried, high N fertilization | | |  |  |  |
| 2805 | Rice (Milled Equivalent) | | | 1010253 | | | Rice, Japonica, Yanjing2, dehulled, dried, high N fertilization | | |  |  |  |
| 2805 | Rice (Milled Equivalent) | | | 1010254 | | | Rice, Japonica, Yanjing2, dehulled, dried, moderate N fertilization | | |  |  |  |
| 2805 | Rice (Milled Equivalent) | | | 1010255 | | | Rice, Japonica, Yanjing2, dehulled, dried, moderate N fertilization | | |  |  |  |
| 2805 | Rice (Milled Equivalent) | | | 1010256 | | | Rice, Japonica, Yanjing2, dehulled, dried, without N fertilization | | |  |  |  |
| 2805 | Rice (Milled Equivalent) | | | 1010257 | | | Rice, Japonica, Yanjing5, dehulled, dried, high N fertilization | | |  |  |  |
| 2805 | Rice (Milled Equivalent) | | | 1010258 | | | Rice, Japonica, Yanjing5, dehulled, dried, high N fertilization | | |  |  |  |
| 2805 | Rice (Milled Equivalent) | | | 1010259 | | | Rice, Japonica, Yanjing5, dehulled, dried, moderate N fertilization | | |  |  |  |
| 2805 | Rice (Milled Equivalent) | | | 1010260 | | | Rice, Japonica, Yanjing5, dehulled, dried, moderate N fertilization | | |  |  |  |
| 2805 | Rice (Milled Equivalent) | | | 1010261 | | | Rice, Japonica, Yanjing5, dehulled, dried, without N fertilization | | |  |  |  |
| 2805 | Rice (Milled Equivalent) | | | 1010262 | | | Rice, Japonica, Yanjing9, dehulled, dried, high N fertilization | | |  |  |  |
| 2805 | Rice (Milled Equivalent) | | | 1010263 | | | Rice, Japonica, Yanjing9, dehulled, dried, high N fertilization | | |  |  |  |
| 2805 | Rice (Milled Equivalent) | | | 1010264 | | | Rice, Japonica, Yanjing9, dehulled, dried, moderate N fertilization | | |  |  |  |
| 2805 | Rice (Milled Equivalent) | | | 1010265 | | | Rice, Japonica, Yanjing9, dehulled, dried, moderate N fertilization | | |  |  |  |
| 2805 | Rice (Milled Equivalent) | | | 1010266 | | | Rice, Japonica, Yanjing9, dehulled, dried, without N fertilization | | |  |  |  |
| 2805 | Rice (Milled Equivalent) | | | 1010267 | | | Rice, Japonica, Zaofeng9, dehulled, dried, high N fertilization | | |  |  |  |
| 2805 | Rice (Milled Equivalent) | | | 1010268 | | | Rice, Japonica, Zaofeng9, dehulled, dried, high N fertilization | | |  |  |  |
| 2805 | Rice (Milled Equivalent) | | | 1010269 | | | Rice, Japonica, Zaofeng9, dehulled, dried, moderate N fertilization | | |  |  |  |
| 2805 | Rice (Milled Equivalent) | | | 1010270 | | | Rice, Japonica, Zaofeng9, dehulled, dried, moderate N fertilization | | |  |  |  |
| 2805 | Rice (Milled Equivalent) | | | 1010271 | | | Rice, Japonica, Zaofeng9, dehulled, dried, without N fertilization | | |  |  |  |
| 2805 | Rice (Milled Equivalent) | | | 1010272 | | | Rice, Japonica, Zhendao10, dehulled, dried, high N fertilization | | |  |  |  |
| 2805 | Rice (Milled Equivalent) | | | 1010273 | | | Rice, Japonica, Zhendao10, dehulled, dried, high N fertilization | | |  |  |  |
| 2805 | Rice (Milled Equivalent) | | | 1010274 | | | Rice, Japonica, Zhendao10, dehulled, dried, moderate N fertilization | | |  |  |  |
| 2805 | Rice (Milled Equivalent) | | | 1010275 | | | Rice, Japonica, Zhendao10, dehulled, dried, moderate N fertilization | | |  |  |  |
| 2805 | Rice (Milled Equivalent) | | | 1010276 | | | Rice, Japonica, Zhendao10, dehulled, dried, without N fertilization | | |  |  |  |
| 2805 | Rice (Milled Equivalent) | | | 1010277 | | | Rice, Japonica, Zhendao88, dehulled, dried, high N fertilization | | |  |  |  |
| 2805 | Rice (Milled Equivalent) | | | 1010278 | | | Rice, Japonica, Zhendao88, dehulled, dried, high N fertilization | | |  |  |  |
| 2805 | Rice (Milled Equivalent) | | | 1010279 | | | Rice, Japonica, Zhendao88, dehulled, dried, moderate N fertilization | | |  |  |  |
| 2805 | Rice (Milled Equivalent) | | | 1010280 | | | Rice, Japonica, Zhendao88, dehulled, dried, moderate N fertilization | | |  |  |  |
| 2805 | Rice (Milled Equivalent) | | | 1010281 | | | Rice, Japonica, Zhendao88, dehulled, dried, without N fertilization | | |  |  |  |
| 2805 | Rice (Milled Equivalent) | | | 1010282 | | | Rice, Japonica, Zhendao99, dehulled, dried, high N fertilization | | |  |  |  |
| 2805 | Rice (Milled Equivalent) | | | 1010283 | | | Rice, Japonica, Zhendao99, dehulled, dried, high N fertilization | | |  |  |  |
| 2805 | Rice (Milled Equivalent) | | | 1010284 | | | Rice, Japonica, Zhendao99, dehulled, dried, moderate N fertilization | | |  |  |  |
| 2805 | Rice (Milled Equivalent) | | | 1010285 | | | Rice, Japonica, Zhendao99, dehulled, dried, moderate N fertilization | | |  |  |  |
| 2805 | Rice (Milled Equivalent) | | | 1010286 | | | Rice, Japonica, Zhendao99, dehulled, dried, without N fertilization | | |  |  |  |
| 2805 | Rice (Milled Equivalent) | | | 1010287 | | | Rice, Junam, conventional, brown, raw | | |  |  |  |
| 2805 | Rice (Milled Equivalent) | | | 1010288 | | | Rice, Ld 355/Bg450, parboiled | | |  |  |  |
| 2805 | Rice (Milled Equivalent) | | | 1010289 | | | Rice, Ld 355/Bg450, raw | | |  |  |  |
| 2805 | Rice (Milled Equivalent) | | | 1010292 | | | Rice, milled, raw | | |  |  |  |
| 2805 | Rice (Milled Equivalent) | | | 1010293 | | | Rice, milled, raw | | |  |  |  |
| 2805 | Rice (Milled Equivalent) | | | 1010294 | | | Rice, milled, raw | | |  |  |  |
| 2805 | Rice (Milled Equivalent) | | | 1010295 | | | Rice, milled, raw | | |  |  |  |
| 2805 | Rice (Milled Equivalent) | | | 1010296 | | | Rice, milled, raw | | |  |  |  |
| 2805 | Rice (Milled Equivalent) | | | 1010297 | | | Rice, milled, raw | | |  |  |  |
| 2805 | Rice (Milled Equivalent) | | | 1010298 | | | Rice, milled, raw | | |  |  |  |
| 2805 | Rice (Milled Equivalent) | | | 1010299 | | | Rice, milled, raw | | |  |  |  |
| 2805 | Rice (Milled Equivalent) | | | 1010304 | | | Rice, Milyang204, genetically modified, brown, raw | | |  |  |  |
| 2805 | Rice (Milled Equivalent) | | | 1010305 | | | Rice, Nakdongbyeo, brown, raw | | |  |  |  |
| 2805 | Rice (Milled Equivalent) | | | 1010308 | | | Rice, Panjin Pearl, raw | | |  |  |  |
| 2805 | Rice (Milled Equivalent) | | | 1010312 | | | Rice, parboiled, milled | | |  |  |  |
| 2805 | Rice (Milled Equivalent) | | | 1010313 | | | Rice, polished, raw, milled | | |  |  |  |
| 2805 | Rice (Milled Equivalent) | | | 1010314 | | | Rice, polished, raw | | |  |  |  |
| 2805 | Rice (Milled Equivalent) | | | 1010317 | | | Rice, raw | | |  |  |  |
| 2805 | Rice (Milled Equivalent) | | | 1010318 | | | Rice, raw | | |  |  |  |
| 2805 | Rice (Milled Equivalent) | | | 1010319 | | | Rice, raw | | |  |  |  |
| 2805 | Rice (Milled Equivalent) | | | 1010320 | | | Rice, RD-6, raw | | |  |  |  |
| 2805 | Rice (Milled Equivalent) | | | 1010322 | | | Rice, RD-8, brown, raw, dehulled | | |  |  |  |
| 2805 | Rice (Milled Equivalent) | | | 1010328 | | | Rice, red, raw | | |  |  |  |
| 2805 | Rice (Milled Equivalent) | | | 1010332 | | | Rice, Thailand, raw | | |  |  |  |
| 2805 | Rice (Milled Equivalent) | | | 1010333 | | | Rice, Tianjin Xiao Zhan, raw | | |  |  |  |
| 2805 | Rice (Milled Equivalent) | | | 1010334 | | | Rice, unpolished, raw | | |  |  |  |
| 2805 | Rice (Milled Equivalent) | | | 1010337 | | | Rice, white, long grains, raw | | |  |  |  |
| 2805 | Rice (Milled Equivalent) | | | 1010338 | | | Rice, white, Manischewitz's brand (kosher), raw | | |  |  |  |
| 2805 | Rice (Milled Equivalent) | | | 1010339 | | | Rice, Calrose, white, raw | | |  |  |  |
| 2805 | Rice (Milled Equivalent) | | | 1010340 | | | Rice, white, raw | | |  |  |  |
| 2805 | Rice (Milled Equivalent) | | | 1010341 | | | Rice, white, short grains, raw | | |  |  |  |
| 2805 | Rice (Milled Equivalent) | | | 1010342 | | | Rice, white, sunned, aromatic | | |  |  |  |
| 2805 | Rice (Milled Equivalent) | | | 1010343 | | | Rice, white, sunned, polished, milled | | |  |  |  |
| 2805 | Rice (Milled Equivalent) | | | 1010345 | | | Rice, whole grain, raw | | |  |  |  |
| 2848 | Milk - Excluding Butter | | | 11010002 | | | Milk, cow, raw | | |  |  |  |
| **Flour** |  | | |  | | |  | | |  |  |  |
| *Whole* |  | | |  | | |  | | |  |  |  |
| 2511 | Wheat and products | | | 1030185 | | | Wheat flour, atta | | |  |  |  |
| 2511 | Wheat and products | | | 1030209 | | | Wheat flour, whole grain | | |  |  |  |
| 2511 | Wheat and products | | | 1030210 | | | Wheat flour, whole grain | | |  |  |  |
| 2511 | Wheat and products | | | 1030211 | | | Wheat flour, whole grain | | |  |  |  |
| 2511 | Wheat and products | | | 1030212 | | | Wheat flour, whole grain | | |  |  |  |
| 2511 | Wheat and products | | | 1030213 | | | Wheat flour, whole grain | | |  |  |  |
| 2511 | Wheat and products | | | 1030214 | | | Wheat flour, whole grain | | |  |  |  |
| 2511 | Wheat and products | | | 1030215 | | | Wheat flour, whole grain | | |  |  |  |
| 2511 | Wheat and products | | | 1030216 | | | Wheat flour, whole grain, Pillsbury | | |  |  |  |
| 2511 | Wheat and products | | | 1030217 | | | Wheat flour, whole meal | | |  |  |  |
| *Refined* |  | | |  | | |  | | |  |  |  |
| 2511 | Wheat and products | | | 1030168 | | | Wheat flour | | |  |  |  |
| 2511 | Wheat and products | | | 1030169 | | | Wheat flour | | |  |  |  |
| 2511 | Wheat and products | | | 1030170 | | | Wheat flour | | |  |  |  |
| 2511 | Wheat and products | | | 1030171 | | | Wheat flour | | |  |  |  |
| 2511 | Wheat and products | | | 1030172 | | | Wheat flour | | |  |  |  |
| 2511 | Wheat and products | | | 1030173 | | | Wheat flour | | |  |  |  |
| 2511 | Wheat and products | | | 1030174 | | | Wheat flour | | |  |  |  |
| 2511 | Wheat and products | | | 1030175 | | | Wheat flour | | |  |  |  |
| 2511 | Wheat and products | | | 1030176 | | | Wheat flour | | |  |  |  |
| 2511 | Wheat and products | | | 1030181 | | | Wheat flour, 50% extraction | | |  |  |  |
| 2511 | Wheat and products | | | 1030182 | | | Wheat flour, 75% extraction | | |  |  |  |
| 2511 | Wheat and products | | | 1030183 | | | Wheat flour, 85% extraction | | |  |  |  |
| 2511 | Wheat and products | | | 1030184 | | | Wheat flour, all-purpose, General Mills | | |  |  |  |
| 2511 | Wheat and products | | | 1030186 | | | Wheat flour, Back Cross of Roshan | | |  |  |  |
| 2511 | Wheat and products | | | 1030187 | | | Wheat flour, Back Cross of Roshan, hydrothermaled | | |  |  |  |
| 2511 | Wheat and products | | | 1030191 | | | Wheat flour, Tajan | | |  |  |  |
| 2511 | Wheat and products | | | 1030192 | | | Wheat flour, Tajan, hydrothermaled | | |  |  |  |
| 2511 | Wheat and products | | | 1030193 | | | Wheat flour, type 500-1 | | |  |  |  |
| 2511 | Wheat and products | | | 1030194 | | | Wheat flour, type 500-2 | | |  |  |  |
| 2511 | Wheat and products | | | 1030196 | | | Wheat flour, type for leavened pastry | | |  |  |  |
| 2511 | Wheat and products | | | 1030201 | | | Wheat flour, white | | |  |  |  |
| 2511 | Wheat and products | | | 1030202 | | | Wheat flour, white | | |  |  |  |
| 2511 | Wheat and products | | | 1030203 | | | Wheat flour, white | | |  |  |  |
| 2511 | Wheat and products | | | 1030204 | | | Wheat flour, white | | |  |  |  |
| 2511 | Wheat and products | | | 1030205 | | | Wheat flour, white | | |  |  |  |
| 2511 | Wheat and products | | | 1030206 | | | Wheat flour, white | | |  |  |  |
| 2511 | Wheat and products | | | 1030207 | | | Wheat flour, white, packaged | | |  |  |  |
| 2511 | Wheat and products | | | 1030208 | | | Wheat flour, white, refined | | |  |  |  |
| 2511 | Wheat and products | | | 1030219 | | | Wheat, baking flour | | |  |  |  |
| *Whole/refined* |  | | |  | | |  | | |  |  |  |
| 2514 | Maize and products | | | 1020060 | | | Maize flour, 65% extraction | | |  |  |  |
| *Whole/refined* |  | | |  | | |  | | |  |  |  |
| 2517 | Millet and products | | | 1050076 | | | Finger millet flour | | |  |  |  |
| 2517 | Millet and products | | | 1050100 | | | Foxtail millet flour | | |  |  |  |
| 2517 | Millet and products | | | 1050101 | | | Foxtail millet flour | | |  |  |  |
| 2517 | Millet and products | | | 1050123 | | | Pearl millet flour, Ashana, dehulled seed | | |  |  |  |
| 2517 | Millet and products | | | 1050124 | | | Pearl millet flour, Ashana, dehulled seed, 30 days storage | | |  |  |  |
| 2517 | Millet and products | | | 1050125 | | | Pearl millet flour, Ashana, dehulled seed, 60 days storage | | |  |  |  |
| 2517 | Millet and products | | | 1050129 | | | Pearl millet flour, Ashana, dehulled seed, irradiated | | |  |  |  |
| 2517 | Millet and products | | | 1050130 | | | Pearl millet flour, Ashana, dehulled seed, irradiated, 30 days storage | | |  |  |  |
| 2517 | Millet and products | | | 1050131 | | | Pearl millet flour, Ashana, dehulled seed, irradiated, 60 days storage | | |  |  |  |
| 2517 | Millet and products | | | 1050135 | | | Pearl millet flour, Ashana, whole seed | | |  |  |  |
| 2517 | Millet and products | | | 1050136 | | | Pearl millet flour, Ashana, whole seed, 30 days storage | | |  |  |  |
| 2517 | Millet and products | | | 1050137 | | | Pearl millet flour, Ashana, whole seed, 60 days storage | | |  |  |  |
| 2517 | Millet and products | | | 1050141 | | | Pearl millet flour, Ashana, whole seed, raw, irradiated | | |  |  |  |
| 2517 | Millet and products | | | 1050142 | | | Pearl millet flour, Ashana, whole seed, irradiated, 30 days storage | | |  |  |  |
| 2517 | Millet and products | | | 1050143 | | | Pearl millet flour, Ashana, whole seed, irradiated, 60 days storage | | |  |  |  |
| 2517 | Millet and products | | | 1050147 | | | Pearl millet flour, Dembi, dehulled seed | | |  |  |  |
| 2517 | Millet and products | | | 1050148 | | | Pearl millet flour, Dembi, dehulled seed, 30 days storage | | |  |  |  |
| 2517 | Millet and products | | | 1050149 | | | Pearl millet flour, Dembi, dehulled seed, 60 days storage | | |  |  |  |
| 2517 | Millet and products | | | 1050153 | | | Pearl millet flour, Dembi, dehulled seed, irradiated | | |  |  |  |
| 2517 | Millet and products | | | 1050154 | | | Pearl millet flour, Dembi, dehulled seed, irradiated, 60 days storage | | |  |  |  |
| 2517 | Millet and products | | | 1050158 | | | Pearl millet flour, Dembi, dehulled seed,irradiated, 30 days storage | | |  |  |  |
| 2517 | Millet and products | | | 1050159 | | | Pearl millet flour, Dembi, whole seed | | |  |  |  |
| 2517 | Millet and products | | | 1050160 | | | Pearl millet flour, Dembi, whole seed, 30 days storage | | |  |  |  |
| 2517 | Millet and products | | | 1050161 | | | Pearl millet flour, Dembi, whole seed, 60 days storage | | |  |  |  |
| 2517 | Millet and products | | | 1050165 | | | Pearl millet flour, Dembi, whole seed, raw, irradiated | | |  |  |  |
| 2517 | Millet and products | | | 1050166 | | | Pearl millet flour, Dembi, whole seed, irradiated, 30 days storage | | |  |  |  |
| 2517 | Millet and products | | | 1050167 | | | Pearl millet flour, Dembi, whole seed, irradiated, 60 days storage | | |  |  |  |
| 2517 | Millet and products | | | 1050171 | | | Pearl millet flour, HHB-67 | | |  |  |  |
| 2517 | Millet and products | | | 1050172 | | | Pearl millet flour, IS 833 | | |  |  |  |
| 2517 | Millet and products | | | 1050173 | | | Pearl millet flour, IS 843 | | |  |  |  |
| 2517 | Millet and products | | | 1050174 | | | Pearl millet flour, IS 880004 | | |  |  |  |
| 2517 | Millet and products | | | 1050175 | | | Pearl millet flour, IS 89111 | | |  |  |  |
| 2517 | Millet and products | | | 1050176 | | | Pearl millet flour, IS 91333 | | |  |  |  |
| 2517 | Millet and products | | | 1050177 | | | Pearl millet flour, IS 91666 | | |  |  |  |
| 2517 | Millet and products | | | 1050178 | | | Pearl millet flour, IS 91777 | | |  |  |  |
| 2517 | Millet and products | | | 1050179 | | | Pearl millet flour, Kabti | | |  |  |  |
| 2517 | Millet and products | | | 1050180 | | | Pearl millet flour, Tihama | | |  |  |  |
| 2517 | Millet and products | | | 1050181 | | | Pearl millet flour, YD-X3 | | |  |  |  |
| *Whole/refined* | | |  | | |  | | |  | | |  |
| 2518 | Sorghum and products | | | 1040013 | | | Sorghum flour | | |  |  |  |
| 2518 | Sorghum and products | | | 1040015 | | | Sorghum flour, Dabar, whole grain, 10KGy irradiated, dried | | |  |  |  |
| 2518 | Sorghum and products | | | 1040019 | | | Sorghum flour, Dabar, whole grain, 15KGy irradiated, dried | | |  |  |  |
| 2518 | Sorghum and products | | | 1040023 | | | Sorghum flour, Dabar, whole grain, 5KGy irradiated, dried | | |  |  |  |
| 2518 | Sorghum and products | | | 1040027 | | | Sorghum flour, Dabar, whole grain, dried | | |  |  |  |
| 2518 | Sorghum and products | | | 1040030 | | | Sorghum flour, ground-dried | | |  |  |  |
| 2518 | Sorghum and products | | | 1040032 | | | Sorghum flour, Karamaka, whole grain, 10KGy irradiated, dried | | |  |  |  |
| 2518 | Sorghum and products | | | 1040036 | | | Sorghum flour, Karamaka, whole grain, 15KGy irradiated, dried | | |  |  |  |
| 2518 | Sorghum and products | | | 1040039 | | | Sorghum flour, Karamaka, whole grain, 5KGy irradiated, dried | | |  |  |  |
| 2518 | Sorghum and products | | | 1040044 | | | Sorghum flour, Karamaka, whole grain, dried | | |  |  |  |
| 2518 | Sorghum and products | | | 1040047 | | | Sorghum flour, Tabat | | |  |  |  |
| 2518 | Sorghum and products | | | 1040049 | | | Sorghum flour, Wad Ahmed, whole grain, 10KGy irradiated, dried | | |  |  |  |
| 2518 | Sorghum and products | | | 1040053 | | | Sorghum flour, Wad Ahmed, whole grain, 15KGy irradiated, dried | | |  |  |  |
| 2518 | Sorghum and products | | | 1040057 | | | Sorghum flour, Wad Ahmed, whole grain, 5KGy irradiated, dried | | |  |  |  |
| 2518 | Sorghum and products | | | 1040061 | | | Sorghum flour, Wad Ahmed, whole grain, dried | | |  |  |  |
| *No matches- assumed to be 0* | | | |  | | |  | | |  |  |  |
| 2543 | Sweeteners, Other | | |  | | |  | | |  |  |  |
| 2563 | Olives (including preserved) | | | | | |  | | |  |  |  |
| 2571 | Soyabean Oil | | |  | | |  | | |  |  |  |
| 2573 | Sunflowerseed Oil | | |  | | |  | | |  |  |  |
| 2574 | Rape and Mustard Oil | | |  | | |  | | |  |  |  |
| 2575 | Cottonseed Oil | | |  | | |  | | |  |  |  |
| 2576 | Palmkernel Oil | | |  | | |  | | |  |  |  |
| 2578 | Coconut Oil | | |  | | |  | | |  |  |  |
| 2579 | Sesameseed Oil | | |  | | |  | | |  |  |  |
| 2580 | Olive Oil | | |  | | |  | | |  |  |  |
| 2581 | Ricebran Oil | | |  | | |  | | |  |  |  |
| 2582 | Maize Germ Oil | | |  | | |  | | |  |  |  |
| 2586 | Oilcrops Oil, Other | | |  | | |  | | |  |  |  |
| 2612 | Lemons, Limes and products | | | | | |  | | |  |  |  |
| 2613 | Grapefruit and products | | |  | | |  | | |  |  |  |
| 2655 | Wine | | |  | | |  | | |  |  |  |
| 2656 | Beer | | |  | | |  | | |  |  |  |
| 2657 | Beverages, Fermented | | |  | | |  | | |  |  |  |
| 2658 | Beverages, Alcoholic | | |  | | |  | | |  |  |  |
| 2659 | Alcohol, Non-Food | | |  | | |  | | |  |  |  |
| 2680 | Infant food | | |  | | |  | | |  |  |  |
| 2731 | Bovine Meat | | |  | | |  | | |  |  |  |
| 2732 | Mutton & Goat Meat | | |  | | |  | | |  |  |  |
| 2733 | Pigmeat | | |  | | |  | | |  |  |  |
| 2734 | Poultry Meat | | |  | | |  | | |  |  |  |
| 2735 | Meat, Other | | |  | | |  | | |  |  |  |
| 2736 | Offals, Edible | | |  | | |  | | |  |  |  |
| 2737 | Fats, Animals, Raw | | |  | | |  | | |  |  |  |
| 2740 | Butter, Ghee | | |  | | |  | | |  |  |  |
| 2743 | Cream | | |  | | |  | | |  |  |  |
| 2744 | Eggs | | |  | | |  | | |  |  |  |
| 2745 | Honey | | |  | | |  | | |  |  |  |
| 2762 | Demersal Fish | | |  | | |  | | |  |  |  |
| 2765 | Crustaceans | | |  | | |  | | |  |  |  |
| 2766 | Cephalopods | | |  | | |  | | |  |  |  |
| 2767 | Molluscs, Other | | |  | | |  | | |  |  |  |
| 2768 | Meat, Aquatic Mammals | | |  | | |  | | |  |  |  |
| 2769 | Aquatic Animals, Others | | |  | | |  | | |  |  |  |
| 2781 | Fish, Body Oil | | |  | | |  | | |  |  |  |
| 2782 | Fish, Liver Oil | | |  | | |  | | |  |  |  |

**Supplementary Table 4: NBS food matches for phytate**

Food composition data come from PhyFoodComp1.0 available at the International Network of Food Data Systems (INFOODS) (https://www.fao.org/infoods/infoods/tables-and-databases/faoinfoods-databases/en/). Matches were based on definitions for FAO Food Balance Sheet (FBS) categories, using food items in primary commodity form or processed and cooked as specified in the FDC item name.
